# Direct Synthesis of Polyesterether from Ethylene Glycol

**DOI:** 10.1021/acssuschemeng.5c00886

**Published:** 2025-04-08

**Authors:** Garima Saini, Pavel Kulyabin, Angus McLuskie, Niklas von Wolff, Amit Kumar

**Affiliations:** † EaStCHEM, School of Chemistry, University of St. Andrews, North Haugh, St. Andrews KY16 9ST, U.K.; ‡ Sorbonne Université, Institut Parisien de Chimie Moléculaire, IPCM, F-75005 Paris, France

**Keywords:** polyesterether, dehydrogenation, ethylene glycol, hydrogen-borrowing, ruthenium

## Abstract

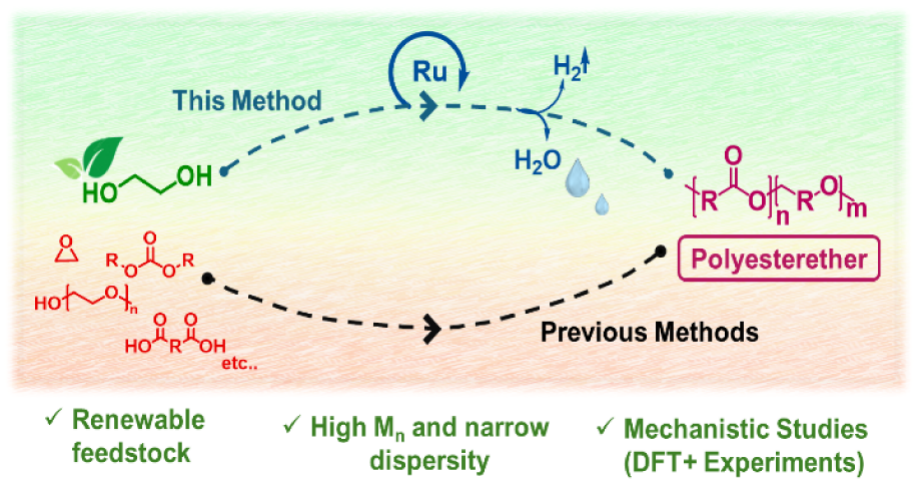

We report here a method for making polyesterether from ethylene glycol. The reaction is catalyzed by a ruthenium complex and liberates H_2_ gas and H_2_O as byproducts. Mechanistic studies conducted through experiments and DFT computations suggest that the chain growth of the polymerization process involves both dehydrogenation and dehydration pathways stemming from a hemiacetal intermediate, leading to the formation of esters and ethers, respectively. Investigations into the polymerization of other diols have also been conducted, showing that diols with a lower number of carbons between the alcohol groups (propylene glycol, glycerol, and 1,3-propanediol) lead to the formation of polyesterether whereas α,ω-diols containing a higher number of carbons (1,6-hexanediol and 1,10-decanediol) lead to the formation of polyester.

## Abstract 2

We report here a method for making polyesterethers directly from ethylene glycol, forming water and H_2_ gas as the only byproducts.

## Introduction

**Figure 1:**
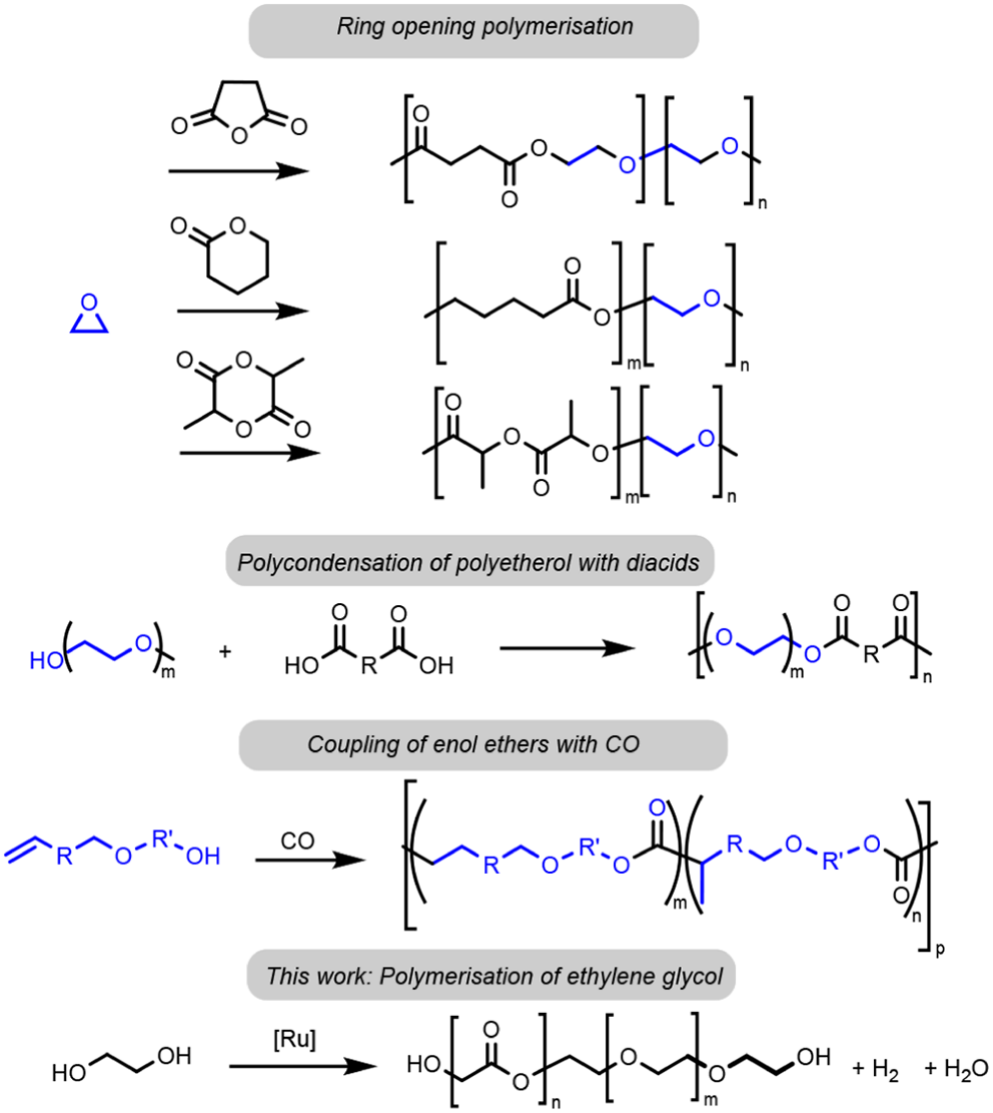
Known methods for the synthesis of polyesterethers and the method disclosed herein.

Aliphatic copolyesterethers have attracted significant interest recently due to their excellent biocompatibility, biodegradability and applications in areas such as tissue engineering and packaging.^1,2^ The conventional methods for their synthesis involve ring-opening polymerization of epoxides with lactones,^3,4^ lactides,^5−8^ or anhydrides (Figure 1).^9,10^ However, these methods often involve multiple steps of polymerization and purification, which make their large-scale application challenging. A few other methods based on melt polymerization of polyethylene glycol with diacids or diesters,^11,12^ hydroesterificative polymerization (coupling of enol ethers with CO),^13^ and reduction of polyesters^14^ have also been reported in the past (Figure 1). Most of these processes involve monomers that require multiple steps to be synthesized and are derived from fossil fuels. As such, there is a need to develop new methods for the synthesis of aliphatic copolyesterethers from renewable, inexpensive, and commercially available feedstock.

Catalytic dehydrogenation is an atom-economic approach for the synthesis of organic compounds, especially those containing carbonyl compounds such as aldehydes, ketones, esters, and amides.^15^ The concept of acceptorless dehydrogenative catalysis has also been utilized for the synthesis of various polymers.^15^ For example, Milstein^16^ reported the synthesis of polyamides from the dehydrogenative coupling of diols and diamines. Recently, we,^17−19^ Robertson,^20^ and Liu^21^ have independently reported the synthesis of polyureas from the dehydrogenative coupling of diamines and methanol/diformamides. We have also reported the synthesis of polyethylenimines a from the coupling of diols and diamines using the hydrogen-borrowing approach.^22^ Along this line, Robertson reported the synthesis of high molar mass polyesters from the dehydrogenative coupling of diols with an alkyl spacer of six carbons or more (e.g., hexane diol) using Milstein’s RuPNN catalyst.^23^ Smaller diols, such as ethylene glycol or propylene glycol, did not result in the formation of polyester, presumably due to the formation of a metallacycle with ruthenium that could block the active site. Recently, Milstein has reported the use of ethylene glycol as a liquid organic hydrogen carrier, where ethylene glycol was dehydrogenated to form a “polyester,” which could be hydrogenated back to ethylene glycol.^24^ However, limited emphasis was given to the isolation and chemical and physical characterization of the formed polymers.

1,2-Diols, such as ethylene glycol, propylene glycol, and glycerol, can be sourced from biomass and are attractive feedstocks for making renewable polymers.^25−28^ We envisioned that their dehydrogenative/dehydrative coupling could lead to the formation of polyester, polyether, or polyesterether. To test this hypothesis, we studied the polymerization of ethylene glycol using bifunctional ruthenium catalysts known for the dehydrogenation of alcohols and hydrogenation of carbonyl compounds, as reported herein.

## Experimental Section

### General Method for Polymerization of Ethylene Glycol under Closed Conditions

A 100 mL ampule equipped with a J. Young’s valve was charged with precatalyst (e.g., **Ru-5**; 8.3 mg, 0.01 mmol, 1 mol %) and base (e.g., KO^t^Bu, 2.2 mg, 0.02 mmol, 2 mol %). THF (2 mL) and ethylene glycol (0.11 mL, 2.0 mmol) were added, and the flask was sealed under an argon atmosphere before heating to the desired temperature (e.g., 150 °C) for the desired length of time (e.g., 24 h) with stirring. After this period, the reaction vessel was allowed to cool to room temperature, and the amount of gas evolved (presumably H_2_) during the reaction was measured by syringe and analyzed by GC-TCD (Gas Chromatography-Thermal Conductivity Detector). The solvent was removed by rotary evaporator. Water (10 mL) was then added to the residue, followed by vacuum filtration to remove any solid as the desired product is soluble in water. The aqueous solution containing the product was concentrated using a rotary evaporator to remove the water. It was then analyzed by different characterization techniques like NMR and IR spectroscopy, GPC, TGA, and DSC, further details of which are given in the Supporting Information.

## Results and Discussion

**Figure 2:**
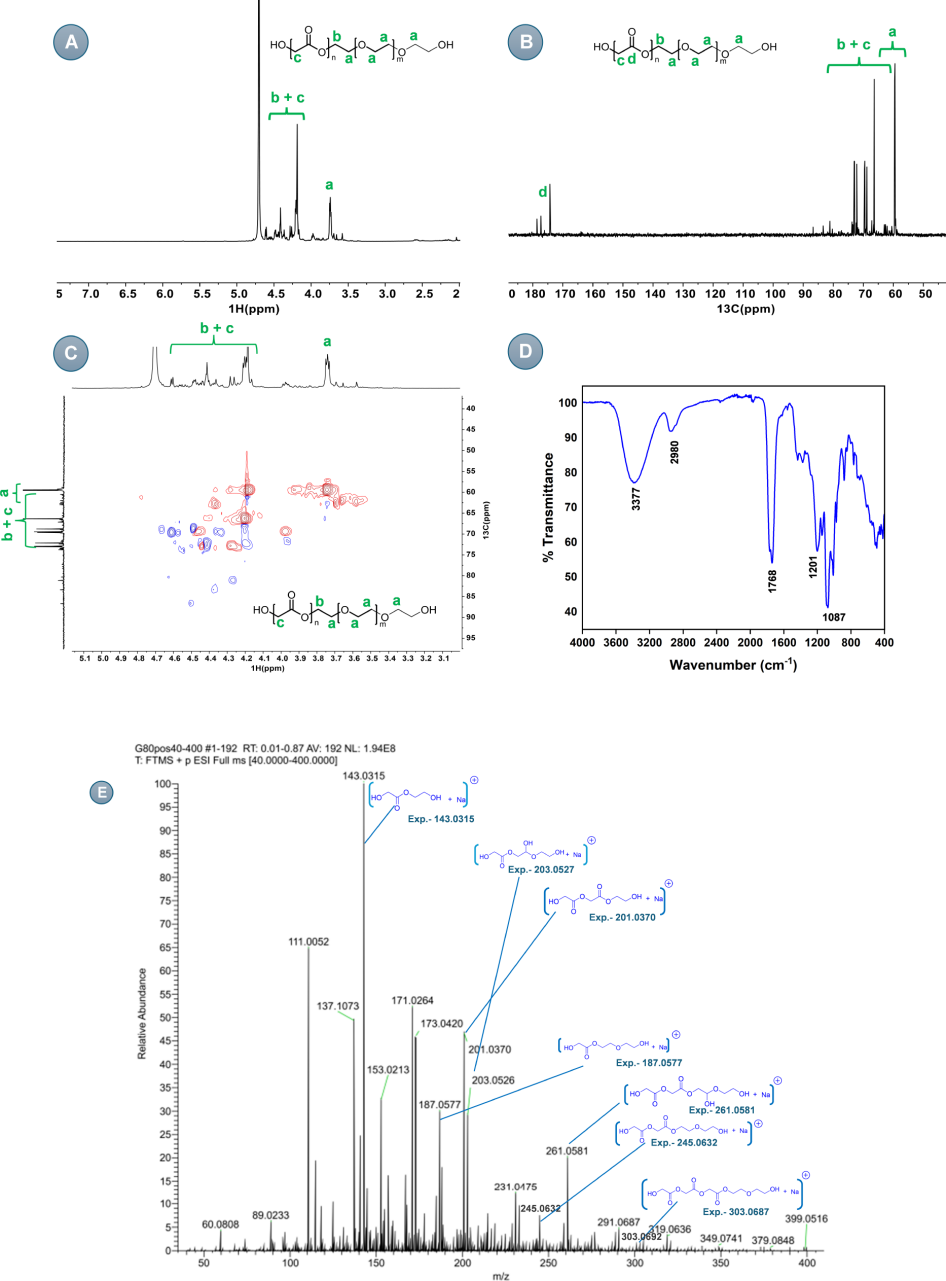
^1^H NMR (A), ^13^C{^1^H} NMR (B), HSQC NMR (C), and IR(D) spectra, as well as ESI-MS data (E) for the polyesterether.

We began our investigation by studying the self-coupling of ethylene glycol in the presence of various ruthenium catalysts known for their catalytic dehydrogenation of alcohols. In a pilot experiment, 2 mmol of ethylene glycol was refluxed in 2 mL of THF in a sealed system in the presence of 1 mol % Ru-MACHO complex (**Ru-1**) and 2 mol % KO^t^Bu at 150 °C for 24 h (Table 1, entry 1). At the end of the reaction, 24 mL of gas was collected in the overhead space of the Young’s flask. Analysis of the gas by GC-TCD (Gas Chromatography-Thermal Conductivity Detector) confirmed that the released gas was mainly H_2_ with a small component of CO (<1%). Conversion of ethylene glycol was found to be 63%, as determined by ^1^H NMR spectroscopy of the crude reaction mixture in D_2_O using ethylene carbonate as an internal standard. The product was isolated by extraction in water and characterized by NMR, IR spectroscopy, and GPC (Gel Permeation Chromatography). The GPC analysis confirmed the product to be a polymer with a molar mass *M*_n_ = 38,020 Da and a dispersity of 1.3. Analysis by NMR spectroscopy revealed that the polymer contains both ester and ether linkages in a ratio of 3.6:1, suggesting that the polymer belongs to the class of polyesterethers (Figure 2A,B). This was further confirmed by IR spectroscopy analysis, which showed the presence of ester (1768 cm^–1^) and ether linkages (1087–1201 cm^–1^) in addition to the presence of the O–H (3377 cm^–1^) and aliphatic C–H (2980 cm^–1^) stretches (Figure 2D). The HSQC (multiplicity-edited) spectrum (Figure 2C) shows the correlation between the hydrogens and carbons of the polymer and also suggested (see Figure S9) the presence of a hemiacetal-type intermediate, which was further confirmed by the ESI-MS study (Figure 2E) that showed the presence of various random oligoesterethers with molar masses up to 400 Da.

**Table 1:**
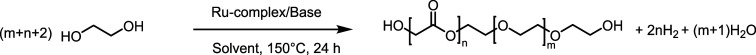
Optimization of Catalytic Conditions for the Coupling of Ethylene Glycol^i^

Entry no.	Precatalyst	Base	Solvent	Time (h)	Gas released (mL)^a^	Conversion (%)^b^	Molar mass, *M*_n_	*Đ*	Ester:Ether^c^
1	Ru-1	KO^t^Bu	THF	24	24	63	38,020	1.3	3.6:1
2	Ru-2	KO^t^Bu	THF	24	19	37	34,640	1.3	4.2:1
3	Ru-3	KO^t^Bu	THF	24	3	14	-	-	1.8:1
4	Ru-4	KO^t^Bu	THF	24	10	22	-	-	-
5	Ru-5	KO^t^Bu	THF	24	49	89	34,450	1.3	4.6:1
6^d^	Ru-5	KO^t^Bu	THF	24	10	20	-	-	-
7^e^	Ru-5	KO^t^Bu	THF	24	22	47	15,640	1.5	3.8:1
8	Ru-5	KO^t^Bu	H_2_O	24	4	10	-	-	-
9	Ru-5	KO^t^Bu	Diglyme	24	45	83	31,200	1.3	4.2:1
10	Ru-5	KO^t^Bu	DME	24	56	91	11,770	2.6	5.6:1
11	Ru-5	KO^t^Bu	DME	72	66	95	24,320	1.5	4.8:1
12^f^	Ru-5	KO^t^Bu	Diglyme	24	-	81	32,680	1.4	4.2:1
13	Ru-5	KO^t^Bu	THF	48	60	94	48,940	1.4	4.2:1
14^g^	Ru-5	KO^t^Bu	THF	24	43	79	24,010	1.6	3.6:1
15		KO^t^Bu	THF	24	1	1	-	-	-
16	Ru-5	K_2_CO_3_	THF	24	39	87	29,130	1.5	3.0:1
17	Ru-5	KOH	THF	24	34	84	33,370	1.4	2.8:1
18	Ru-5	KBH_4_	THF	24	21	55	17,370	1.6	3.4:1
19	Ru-5	-	THF	24	3	7	-	-	-
20^h^	Ru-5	KO^t^Bu	THF	24	46	83	55,830	1.2	5.6:1

a
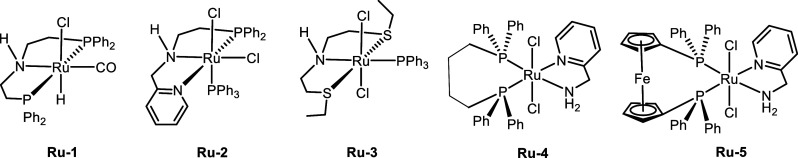
In all cases, released gas was identified to be mainly H_2_ with ≤1% of CO using GC-TCD analysis.

b Conversion was determined by ^1^H NMR spectroscopy of the crude reaction mixture, using ethylene carbonate as an internal standard and D_2_O as solvent.

c Ester:ether ratio was estimated by ^1^H NMR spectroscopy (see Supporting Information for more details).

d Reaction conducted at 100 °C.

e 0.5 mol % catalyst was used.

f Reaction performed under open flow of argon.

g 10 mol % KO^t^Bu was used.

h Reaction with non-anhydrous ethylene glycol.

i Reaction conditions: ethylene glycol: 2 mmol, solvent: 2 mL, catalyst (1 mol %), base (2 mol %).

To achieve a higher conversion of ethylene glycol, we studied various ruthenium catalysts (**Ru-2**–**Ru-5**) under identical reaction conditions. Complexes **Ru-2**, **Ru-3**, and **Ru-4** resulted in lower conversion rates of ethylene glycol, whereas a higher conversion (89%) was obtained with **Ru-5** complex (Table 1, entries 2–5). The polymer obtained in this case was characterized as a polyesterether with a molar mass of 34,450 Da and an ester:ether ratio of 4.6:1 (entry 5). Using **Ru-5**, we further varied reaction conditions (temperature, catalytic loading, solvent, and time) to achieve a higher conversion of ethylene glycol. Performing the reaction at a lower temperature (100 °C) and with lower complex loading (0.5 mol %) resulted in lower conversion of ethylene glycol (20%, and 47%, respectively), as mentioned in Table 1 (entries 6 and 7). Conducting the reaction in water led to a very low conversion of ethylene glycol (10%, entry 8), whereas performing the reaction in diglyme resulted in a higher conversion of ethylene glycol (83%, entry 9). The polyesterether formed in diglyme was found to have a molar mass of 31,200 Da and an ester:ether ratio of 4.2:1. A higher conversion of 91% was achieved when DME (dimethoxyethane) was used as solvent, although the molar mass of the resulting polyesterether was found to be lower (11,770 Da, entry 10) compared to that obtained in diglyme. Increasing the reaction time to 72 h slightly increased the conversion (95%) and the molar mass (24,320 Da, entry 11). Performing the reaction under an open flow of argon in diglyme solvent did not have much effect on the conversion of ethylene glycol or the characteristics of the formed polyesterether in terms of molar mass or ester:ether ratio (entries 9 and 12). Extending the reaction for a longer time (48 h) in THF led to a higher conversion of ethylene glycol (94%) and the formation of a polyesterether with a higher molar mass (48,940 Da, entry 13) compared to the reaction conducted for 24 h (ethylene glycol conversion: 89%, polymer molar mass: 34,450 Da, entry 5). Using a higher base loading (10 mol %) led to the formation of a polyesterether with a relatively lower molar mass (24,010 Da, entry 14) compared to the example where 2 mol % base was used (entry 5). The use of different bases K_2_CO_3_, KOH, and KBH_4_ led to the formation of polyesterethers with lower molar masses (entries 16–18) compared to those produced using KO^t^Bu base (entry 5). Performing the reaction solely in the presence of KO^t^Bu (2 mol %) without using any metal complex (entry 15) or solely in the presence of complex **5** without using any base (entry 19) did not lead to the formation of any polymer, suggesting the crucial role of both complex **5** and the base. Additionally, conducting the reaction with non-anhydrous ethylene glycol led to the formation of less ether compared to ester, indicating that the presence of water is unfavorable for ether formation (entry 20). Similarly, when the reaction was conducted in the presence of molecular sieves (see the Supporting Information), the ester:ether ratio changed to 3.6:1 from 4.6:1 (entry 5), suggesting that the removal of water can promote the etherification process.

To get an idea of the ether chain length, the saponification of a formed polyesterether (*M*_n_ = 35,950 Da, *Đ* = 1.2) was carried out using KOH as the base and water as the solvent at 150 °C for 24 h. After the completion of the reaction, the resulting reaction mixture was extracted with diethyl ether. Analysis of the reaction mixture revealed a combination of polyethylene glycol (confirmed by NMR spectroscopy) with *M*_n_ = 1550 Da (*Đ* = 2.1) and *M*_n_ = 22,660 Da (*Đ* = 1.5). Additionally, ethylene glycol and a higher molecular weight polyethylene glycol [*M*_n_ = 55,580 Da (*Đ* = 1.2)] were also observed (see Section S1.17).

**Figure 3:**
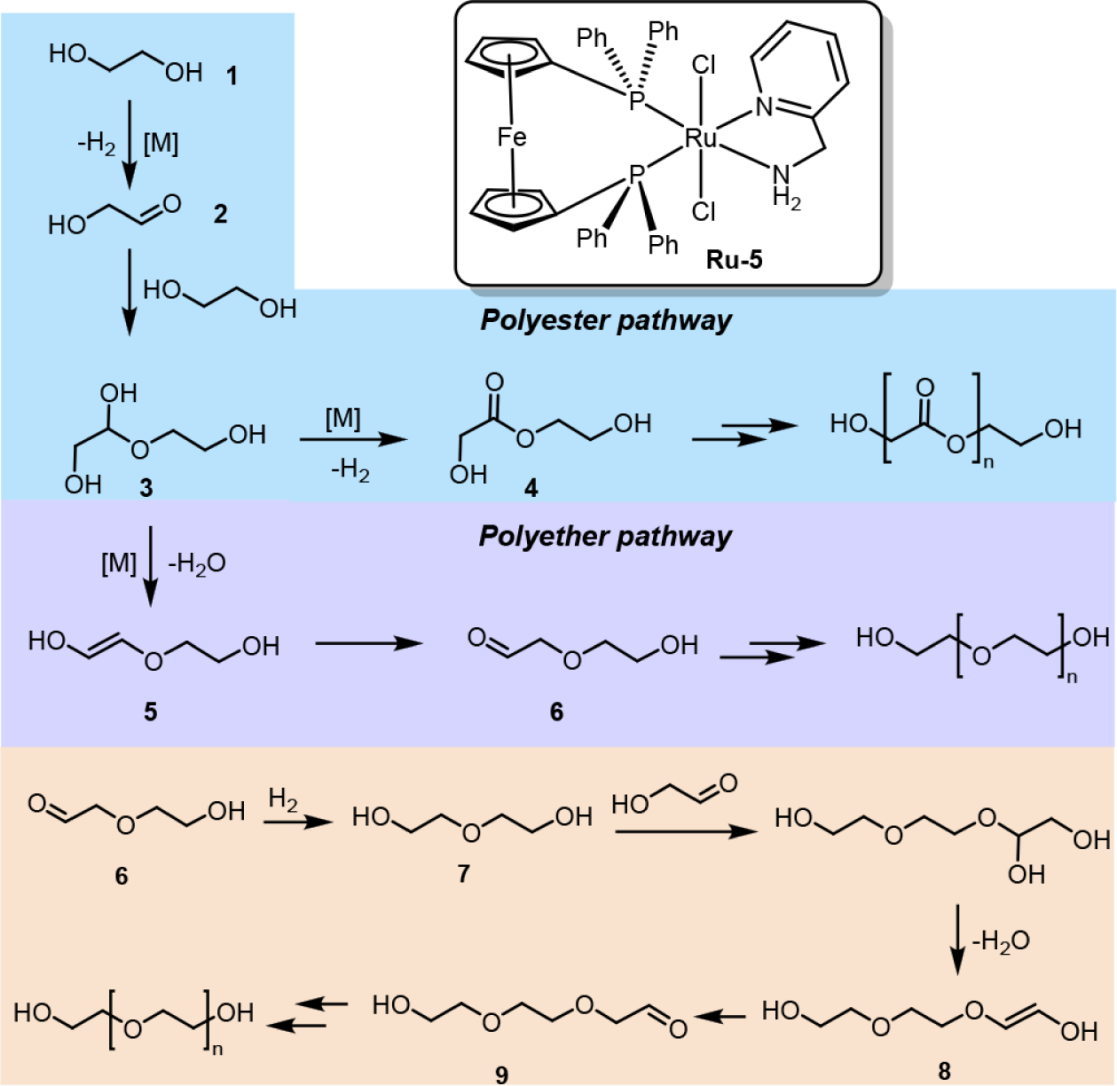
Proposed pathway for the synthesis of polyester and polyether from ethylene glycol.

Having developed the catalytic conditions for the formation of polyesterethers, we turned our attention to understanding the mechanism of the process. The dehydrogenative coupling of alcohols to esters is well studied for a number of ruthenium complexes, including **Ru-1**–**Ru-5**.^29−32^ Based on the reported mechanisms, we suggest that the process begins with the catalytic dehydrogenation of ethylene glycol (**1**) to glycolaldehyde (**2**) which reacts with another molecule of ethylene glycol to form a hemiacetal (**3**) that subsequently undergoes dehydrogenation in the presence of the ruthenium catalyst to form an ester (**4**, Figure 3). However, the formation of ethers from alcohols using bifunctional transition-metal complexes that operate via Noyori-type metal–ligand cooperativity^33^ (e.g., **Ru-1**–**Ru-5**) is intriguing and has not been reported before. In general, alcohol-to-ether transformation is achieved using acid-based catalysts.^34^ We suggest, in this case, that a possible pathway could operate via the dehydration of hemiacetal **3** to form an enol ether (**5**), which could tautomerize to the aldehyde ether (**6**). Conversion of a hemiacetal intermediate to an ether has also been proposed earlier by Beller during the hydrogenation of esters to ethers catalyzed by a ruthenium/Triphos-based catalyst.^35^ We propose that the formation of a polyether sequence from the aldehyde ether intermediate (**6**) could proceed via the hydrogenation of the aldehyde group to form diol intermediate **7** that could then react with glycolaldehyde **2** to form intermediate **8** upon dehydration (Figure 3). The enol intermediate **8** could tautomerize to form the aldehyde intermediate **9,** and the continuation of this process could lead to the formation of polyether.

**Figure 4:**
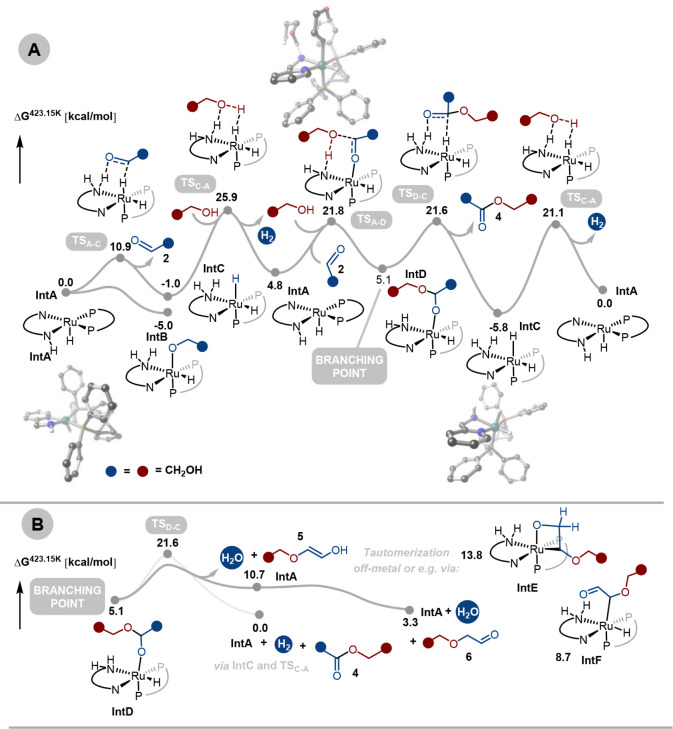
(A) Proposed reaction mechanism for ethylene glycol dehydrogenation to ester promoted by precatalyst **Ru-5**. (B) Possible bifurcation of reaction pathway toward ether intermediate **6**. Free energies were calculated at 423.15 K and at 1 M concentration of solutes in kcal/mol (see the Supporting Information for details).

To further investigate this, we also performed DFT calculations (see the Supporting Information for more information and Figure 4). Under the basic dehydrogenative conditions, precatalyst **Ru-5** could *in situ* generate amido-complex **IntA**. We consider the most stable isomer of **IntA** (square pyramidal with one hydride in the apical position; see the Supporting Information) as a reference point (Δ*G* = 0.0 kcal/mol at 423.15 K). The reaction with ethylene glycol follows the commonly observed pattern for ruthenium-based alcohol dehydrogenation catalysis, with the alkoxide as off-cycle intermediate (**IntB**, −5.0 kcal/mol) and H_2_-elimination as the rate-limiting transition state (**TS**_**A-C**_ = 25.9 kcal/mol, see Figure 4). Importantly, the dual bis-dentate ligand system allows for isomerization, and while **IntA** is most stable in *trans-*N-P configuration, the most stable intermediate (TDI) is the *cis*-dihydride species **IntC** (−5.8 kcal/mol, after the second H_2_ elimination step). This is also corroborated by experiments, showing a *cis*-dihydride as a resting state during dehydrogenative coupling (see discussion below and Section S1.16). Hence, in contrast to the Ru-acridine-ligand system known for ethylene glycol dehydrogenation,^24^ where an open coordination site is available at ruthenium, in the present case, a hydride *trans* to the N—N bidentate ligand remains bound along the catalytic cycle, as observed for other pincer-ruthenium systems.^36,37^ After C—O bond formation between ethylene glycol **1** and glycol aldehyde **2** (**TS**_**C-A**_ = 21.8 kcal/mol), the hemiacetal-bound species **IntD** is formed (+5.1 kcal/mol). Thus, **IntD** represents the branching point for (poly)ester vs (polyester)ether formation (Figure 4B). While acetal dehydrogenation and H_2_ elimination provide free ester **4** (0.0 kcal/mol), **IntD** could also undergo dehydration to release enol **5** together with **IntA** (10.7 kcal/mol). While we were not able to locate a transition state for this step, metal-assisted C—O bond breakage during repeated optimization attempts is consistent with a kinetically feasible, relatively flat PES^38^ (see Section S1.16). Nevertheless, the formation of **5** from **IntD** is 10.7 kcal/mol less favored than ester formation. Tautomerization of **5** to aldehydic ether **6** (+3.3 kcal/mol), e.g., via sigma-complexes **IntE** (13.8 kcal/mol) or **IntF** (8.7 kcal/mol), commonly found in ruthenium-catalyzed C—C double bond isomerization^39−42^ could serve as the driving force for (polyester)ether formation. Indeed, isomerization of alkenes via chain walking and olefin transposition mechanisms has been reported in the past using similar types of bifunctional catalysts that are also known for the dehydrogenation of alcohols.^39−42^

**Figure 5:**
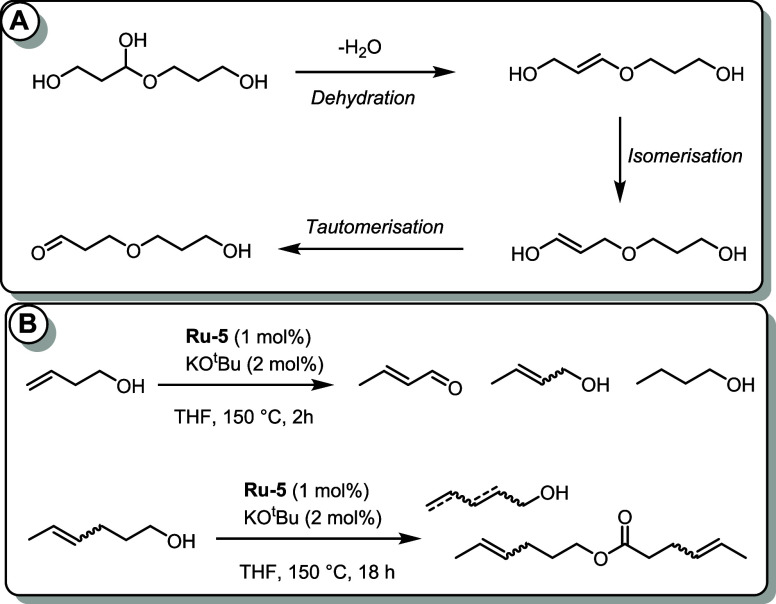
Isomerization and tautomerization steps in the case of 1,3-propanediol (A), and reactivity of alkenols using the **Ru-5** precatalyst (B).

Thus, the relative propensity of ether bond formation vs ester formation will depend on the free energy of H_2_-release vs H_2_O-formation (affected, e.g., by temperature, solubility, mass transfer between the liquid and gas phase^29^ and the isomerization of the C—C double bond for possible enol tautomerization to the aldehyde). With a simple statistical redistribution of the C—C double bond, longer-chain diols are thus expected to give lower yields of (polyester)ether. Indeed, while 1,2-diols and 1,3-diols led to the formation of polyesterethers (Table 2, entries 1–4), higher diols such as 1,6-hexanediol and 1,10-decanediol led to the formation of a polyester (Table 2, entries 5 and 6, see the Supporting Information). Additionally, 1,4-cyclohexanediol under the reaction conditions of Table 2 (entry 1) led to the formation of 4-hydroxycyclohexan-1-one, and the formation of a polymer was not observed in this case (entry 7). Similarly, performing the dehydrogenation of 1-phenylethanol under the reaction conditions of Table 2 (entry 1) led to the formation of acetophenone as the only product with a 45% yield. The lack of ether formation in this case further confirms the hypothesis of the need for an enol-ether type intermediate to form ether. To further confirm this hypothesis of C=C double bond isomerization, we studied the reactivity of 3-butenol and 4-hexenol in the presence of **Ru-5** (1 mol %) precatalyst and KO^t^Bu (2 mol %; Figure 5B). Interestingly, alcohols, aldehydes, and esters with varying positions of C=C were observed, suggesting the possibility of alkene isomerization as hypothesized in Figure 5A. The crucial role of the tautomerization step (only possible with diols) was also addressed by control experiments with n-hexanol or n-propanol instead of ethylene glycol. Under the catalytic conditions described in Table 1 (entry 5), n-hexanol/n-propanol led to the formation of hexanal/propanal and hexyl hexanoate/propyl propanoate, and no ether was formed in this case. This is further supported by the lack of observation of any alkene groups in the NMR spectra of the formed polyesterethers (Table 1 and see Section S1.9) as the enol-ether intermediate (**5**) can easily tautomerize to form the aldehyde-ether intermediate (**6**). To probe whether an aldehyde group could be hydrogenated under the reaction conditions, we performed the polymerization of ethylene glycol (as per the conditions of Table 1, entry 5) in the presence of hexanal (1 mmol). Interestingly, 59% of hexanal was hydrogenated to hexanol in addition to the formation of polyesterether, confirming our hypothesis that the aldehyde group can be hydrogenated to alcohol under these reaction conditions.

**Table 2:**
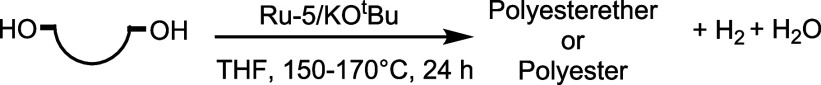

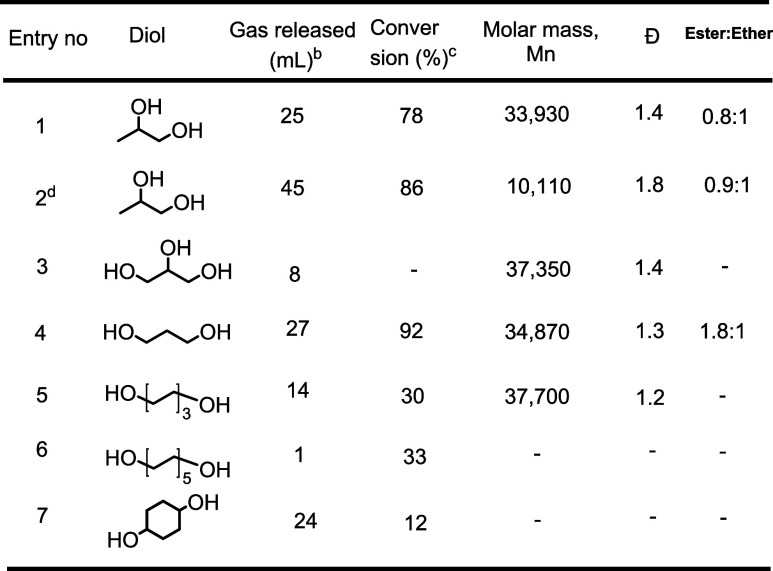
Polymerization with Propylene Glycol, Glycerol, and α,ω-Diols

a Reaction conditions: diol: 2 mmol, THF: 2 mL, **Ru-5** (1 mol %), KO^t^Bu (2 mol %), 150 °C, 24 h.

b In all cases, released gas was identified to be mainly H_2_ with ≤1% of CO using GC-TCD analysis.

c Conversion was determined by ^1^H NMR spectroscopy of the crude reaction mixture, using ethylene carbonate as an internal standard and D_2_O as a solvent.

d Reaction conducted at 170 °C. Molecular weight in the case of 1,10-decanediol could not be estimated due to solubility issues.

**Figure 6:**
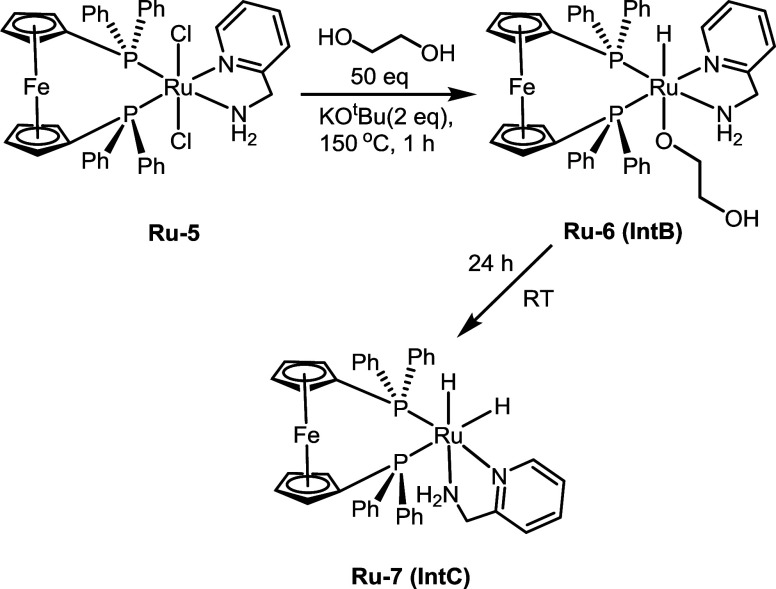
Observation of ruthenium hydride species under catalytic conditions.

To understand the nature of the catalytically active species, we monitored the catalytic reaction by NMR spectroscopy. A ^1^H NMR spectrum taken after 1 h of reaction time under the catalytic conditions described in Table 1, entry 5 (1 mol % complex **5**, 2 mol % KO^t^Bu, THF, 150 °C) showed a doublet of doublets centered at δ −6.47 ppm, suggestive of a hydride species that is both *cis* (^2^J_PH_ = 27.7 Hz) and *trans* (^2^J_PH_ = 120.5 Hz) to phosphorus (see Figure S114). The ^31^P{^1^H} NMR spectrum (see Figure S115) showed the presence of two ruthenium *bis*-phosphine complexes with both phosphorus atoms being *cis* to each other (δ 45.1, 37.3, ^2^J_PP_ = 25.2 Hz; and δ 54.5, 21.8, ^2^J_PP_ = 14.0 Hz). Based on this, we speculate the formation of a ruthenium hydrido alkoxide complex **Ru-6** (**IntB,**Figure 6). Interestingly, leaving the reaction mixture for a further 24 h at room temperature resulted in the disappearance of these signals in ^1^the H and ^31^P{^1^H} NMR spectra. Instead, two hydride signals centered at δ −6.48 [dt: ^2^J_PH_ = 23.3 Hz (t) and ^2^J_HH_ = 5.5 Hz (d)] and −7.9 [ddd: ^2^J_PH_ = 34.3 Hz (coupling with cis phosphorus), 74.4 Hz (coupling with trans phosphorus), and ^2^J_HH_ = 5.9 Hz] ppm were observed. These hydride signals are suggestive of a *cis*-dihydride species complex **Ru-7** (**IntC,**Figure 6). This was further confirmed by the observation of *m*/*z* = 767.06 Da in the ESI-MS, which corresponds to the dihydride complex **Ru-7** (expected *m*/*z*: 767.09 Da).

## Conclusion

In conclusion, we report a new method for the synthesis of an aliphatic polyesterether via ruthenium-catalyzed dehydrative/dehydrogenative polymerization of ethylene glycol. Under our optimized catalytic conditions (1 mol % ruthenium precatalyst, 2 mol % base), we successfully synthesized an aliphatic polyesterether with a reasonable molar mass (34,450 Da) and dispersity (1.3). H_2_ gas and H_2_O are eliminated as the only byproducts in this process. We propose that the reaction occurs via the dehydrogenation of ethylene glycol to glycolaldehyde, which reacts with another molecule of ethylene glycol to form a hemiacetal intermediate. This intermediate can undergo either dehydrogenation to produce an ester or dehydration to form an enol intermediate, which can tautomerize to yield an aldehyde intermediate ready for further chain propagation (Figure 3). The DFT computations suggest that both dehydration and dehydrogenation pathways are facile for ethylene glycol, leading to the formation of polyesterether (Figure 4). The ability of enol ether to tautomerize into an aldehyde is an important step from a thermodynamic perspective, enabling the formation of ether in the case of ethylene glycol but not in the case of propanol. Similarly, both dehydration and dehydrogenation pathways are possible for propylene glycol, glycerol, and 1,3-propanediol, leading to the formation of both ester and ether functionalities (Table 2). In the case of 1,3-propanediol, we suggest that the alkene formed upon dehydration can undergo a chain-walking process to produce an enol intermediate, which can tautomerize to form an aldehyde (Figure 5A). However, we do not observe the formation of ethers in the cases of 1,6-hexanediol and 1,10-decanediol, likely due to the low concentration of the appropriate enol intermediate that could tautomerize to form an aldehyde (Figure 5). Considering that some of these diols (ethylene glycol, propylene glycol, and glycerol) are sourced from renewable feedstocks, the reported methodology presents a useful route for synthesizing renewable polyesterethers.

## Supporting Information Available

Catalytic studies, characterization details, and DFT details (PDF)
The raw research data supporting this publication can be accessed at https://doi.org/10.17630/15c7c187-a8d6-42db-abea-c5df539e14e6.
