# Supplementary material for: Direct Synthesis of Polyesterether from Ethylene Glycol
Source: ACS Sustain Chem Eng. 2025 Apr 8;13(15):5703–11. doi: 10.1021/acssuschemeng.5c00886 (PMC12015959; doi:10.1021/acssuschemeng.5c00886)
Supplement: Supplementary file 1 — sc5c00886_si_001.pdf [file sc5c00886_si_001.pdf]

Electronic Supporting Information for Manuscript:

Direct Synthesis of Polyesterether from Ethylene glycol

Garima Saini,† Pavel Kulyabin,† Angus McLuskie,† Niklas von Wolff*,δ and Amit Kumar*†

† EaStCHEM, School of Chemistry, University of St. Andrews, North Haugh, St. Andrews, KY16 9ST, UK.

δ eMOCA, Institut Parisien de Chimie Moléculaire, Sorbonne Université, CNRS, 4 Place Jussieu, 75005 Paris, France.

Email: ak366@st-andrews.ac.uk, niklas.von-wolff@cnrs.fr

Number of Pages: 83

Number of Figures:133

Number of Tables: 3

Table of Contents

1. Experimental Details 3
  1.1 General Considerations 3
  1.2 Procedure for polymerisation of ethylene glycol 4
  1.3 Method to calculate the conversion of ethylene glycol 4
  1.4 Method to calculate the ester:ether ratio 5
  1.5 Method for product isolation 6
  1.6 Characterisation of Ethylene Glycol 6
  1.7 GC-TCD analysis of the reaction product 8
  1.8 Characterisation of isolated product 8
  1.9 Optimisation of Catalytic Conditions 14
  1.10 Reaction with n-Propanol and n-Hexanol 49
  1.11 Substrate scope 53
  1.12 Reaction with Alkenols 69
  1.13 Reaction of Ethylene Glycol and Hexanal 69
  1.14 Catalyst stability study during reaction 70
  1.15 Study of organometallic species during catalysis 73
  1.16 DFT Studies 76
  1.17 Hydrolysis of Polyesterether 78
  1.18 Reaction with 1-Phenylethanol 80
  1.19 References 82

1. Experimental Details

1.1 General Considerations

All manipulations, unless otherwise stated, were performed under an argon atmosphere using standard Schlenk line and glove-box techniques. Glassware was oven-dried at 130 °C overnight and flamed under vacuum prior to use. THF was dried using a Grubbs-type solvent purification system (Innovative Technologies SPS) equipped with a degasser. Ethylene glycol (anhydrous), diglyme (anhydrous), and ruthenium complex **Ru-1** was purchased from Sigma-Aldrich and TCI and used as received. DME was purchased from Sigma-Aldrich and degassed using freeze-pump-thaw prior to use. Water was degassed by purging argon through it. Complexes **Ru-2**, **Ru-3**, **Ru-4**, and **Ru-5** were donated by Johnson Matthey.

KO^tBu, KOH, K_2CO_3, and KBH_4 were purchased from Sigma-Aldrich, and TCI and stored at 80 °C and dried before use. NMR solvents were purchased from Sigma-Aldrich and used as received.

NMR spectra were recorded on a Bruker AVIII-HD 500 MHz NMR spectrometer at 298 K unless otherwise specified. Residual proton solvent was used as reference for ^1H spectra in deuterated solvent samples. All chemical shifts (δ) are quoted in ppm and coupling constants (*J*) in Hz.

Gel permeation chromatography (GPC) was performed on an Agilent 1260 InfinityLab II GPC fitted with a refractive index (RI) detector (35 °C). The single (plus guard column) Agilent PolarGel column setup was contained within an oven (35 °C). H_2O was used as the eluent at a flow rate of 1.0 mL min^{-1}. Samples were dissolved in the eluent (2.0 mg mL^{-1}), filtered (0.2 µm pore size) and run immediately. The calibration was conducted using a series of monodisperse poly(ethylene glycol) (M_n = 194–20,000 g mol^{-1}) and poly(ethylene oxide) (M_n = 30,000–50,000 g mol^{-1}) standards obtained from Agilent Technologies.

GC-TCD analysis were done with Agilent 8860 GC system. Infrared spectra (ATR-FTIR) were collected using a Shimadzu IRAffinity-1. TGA was performed using an Stanton Redcroft STA-780 Series Thermal Analyser between 20–600°C at a heating rate of 10 °C/min under a flow of nitrogen gas (25 mL/min). DSC analyses were performed using a Netzsch DSC204 between -50–250°C at a heating rate of 10 °C/min under a flow of nitrogen gas (20 mL/min) after an initial heat/cool cycle (25–120 °C at 10 °C/min with a 20 minute isothermal at 120 °C) to remove the thermal history of the sample.

1.2 Procedure for polymerisation of ethylene glycol

General method for polymerisation of ethylene glycol under closed conditions:

A 100 mL ampoule equipped with a J-Young's valve was charged with pre-catalyst (*e.g.* **Ru-1**; 6.1 mg, 0.01 mmol, 1 mol%, 0.005 eq.) and base (*e.g.* KO^{t}Bu, 2.2 mg, 0.02 mmol, 2 mol%, 0.01 eq.). THF (2 mL) and ethylene glycol (0.11 mL, 2.0 mmol, 1 eq.) were added, and the flask was sealed under an argon atmosphere before heating to the desired temperature (*e.g.* 150 °C) for the desired length of time (*e.g.* 24 hours) with stirring. After this period, the reaction vessel was allowed to cool to room temperature, and the amount of gas evolved (presumably H_2) during the reaction was measured by syringe and analysed by GC-TCD (Gas Chromatography-Thermal Conductivity Detector). Methods to analyse the reaction mixture by the NMR spectroscopy and to isolate the product has been described in Sections 1.3-1.5.

Method for polymerisation of ethylene glycol under open conditions:

A 100 mL round bottomed flask equipped with a condenser was charged with pre-catalyst **5** (8.3 mg, 0.01 mmol, 1 mol%, 0.005 eq.) and base (*e.g.* KO^{t}Bu, 2.2 mg, 0.02 mmol, 2 mol%, 0.01 eq.). Diglyme (2 mL) and ethylene glycol (0.11 mL, 2.0 mmol, 1 eq.) were added and the reaction flask was heated to reflux at 150 °C under a continuous flow of argon for 24 hours with stirring. After this period, the flask was allowed to cool to room temperature. Any volatile components from the resulting mixture were removed under reduced pressure to isolate the product that was analysed by NMR and IR spectroscopies.

1.3 Method to calculate the conversion of ethylene glycol

At the end of the reaction, an internal standard ethylene carbonate was added to the crude reaction mixture. An aliquot was taken from the crude reaction mixture and added to D_2O (0.5 mL) and the sample was analysed by ^{1}H NMR spectroscopy. As shown in the following figure (that corresponds to Table S1, entry 1), the signal at δ3.6 ppm was assigned to ethylene glycol (CH_2) and the signal at δ4.5 ppm was assigned to ethylene carbonate (CH_2). The conversion was estimated by the integration of these two signals.

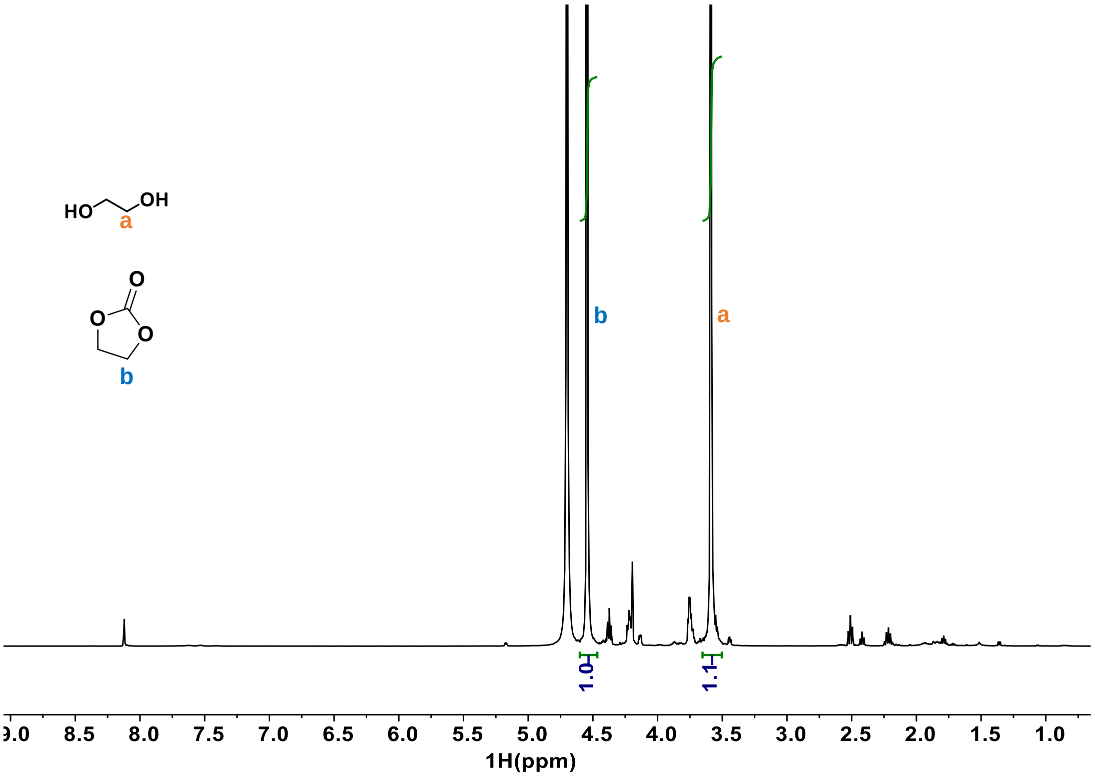

Figure S1. ^{1}H NMR spectrum (D_2O, 500 MHz, 298 K) of the reaction mixture obtained from the polymerisation process conducted as per the method described in Table S1, entry 1.Ethylene carbonate (0.6725 mmol) was used as an internal standard.

The ethylene glycol protons integrate to 1.1 compared to the ethylene carbonate protons normalised at 1. Since 0.6725 mmol of ethylene carbonate (internal standard) was used, the mmoles of ethylene glycol present in the reaction mixture can be calculated:

$$mmoles\ of\ ethylene\ glycol = mmoles\ of\ ethylene\ carbonate * 1.1$$

$$mmoles\ of\ ethylene\ glycol = 0.6725 * 1.1$$

$$mmoles\ of\ ethylene\ glycol = 0.7398\ mmol$$

Since 2 mmol of ethylene glycol was used at the start, the percentage of ethylene glycol left after the reaction can be calculated by using the following equation:

$$\%\ Ethylene\ glycol\ left = \frac{mmoles\ of\ ethylene\ glycol\ left\ after\ the\ reaction}{mmoles\ of\ ethylene\ glycol\ used\ at\ the\ start} \times 100\%$$

$$\%\ Ethylene\ glycol\ left = \frac{0.7398}{2} \times 100\%$$

$$\%\ Ethylene\ glycol\ left = 36.98\%$$

$$\%\ conversion\ = 63.01\%$$

1.4 Method to calculate the ester:ether ratio

As shown in the following figure (that corresponds to Table S1, entry 1), the signals at 4.0-4.4 ppm were assigned to ester (CH_2) and the signal at δ3.8 ppm was assigned to ether (CH_2). The ratio was estimated by the integration

of these two signals. As the ester repeating units have 2 protons while ether repeating units have 4 protons, so the ester integration value is multiplied by 2 to encounter for the value of extra ester moieties.

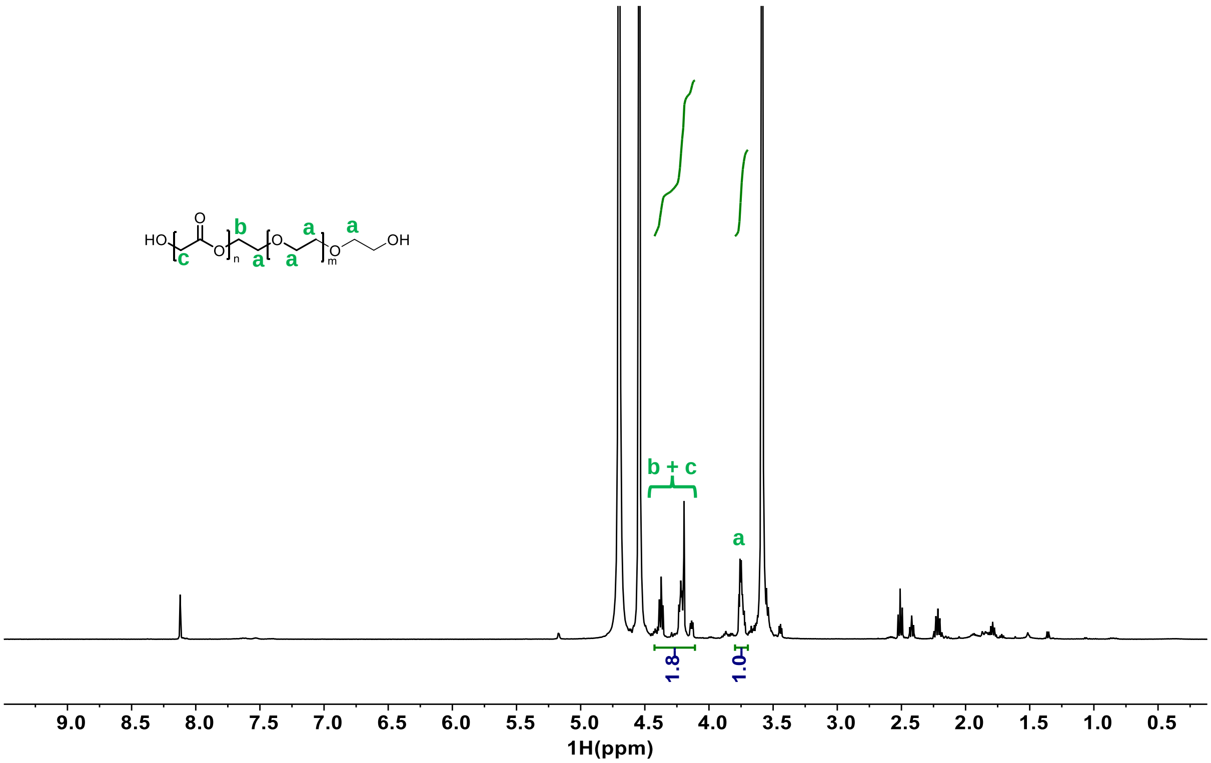

Figure S2. ^{1}H NMR spectrum (D_2O, 500 MHz, 298 K) of the reaction mixture obtained from the polymerisation process conducted as per the method described in Table S1, entry 1.

The ratio of ester and ether moieties present in the polyesterether = {(1.8) X 2}:1=3.6:1.

1.5 Method for product isolation

The reaction mixture was cooled to room temperature and the solvent was removed by rotary evaporator. Water (10 mL) was then added to the residue, followed by vacuum filtration to remove any solid as the desired product is soluble in water. The aqueous solution containing the product was concentrated using a rotary evaporator to remove the water. It was also purified by column chromatography using THF and ethyl acetate solution in a ratio of 1:9 to remove the traces of ethylene glycol left. Finally, the concentrated polymer was dried under vacuum on a Schlenk line for at least 2 hours, yielding the final product.

1.6 Characterisation of Ethylene Glycol

^{1}H NMR (500 MHz, D_2O): δH 3.5 (br, -O-CH_2-**CH_2**-O-).

^{13}C{^{1}H} NMR (126 MHz, D_2O): δC 62.5 (-O-CH_2-**CH_2**-O-).

IR (ATR-FTIR, cm^{-1}): ν 3300 brm (O-H), 2929w (C-H), 1035m(C-O).

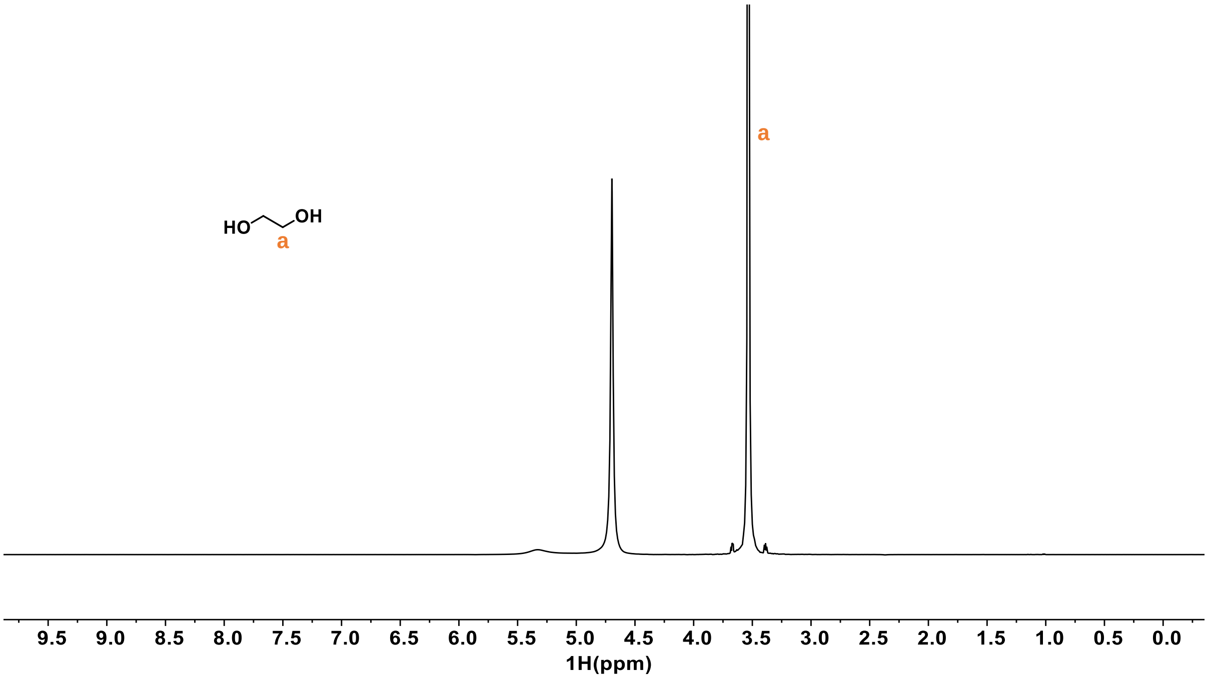

Figure S3. ^{1}H NMR (500 MHz, D_2O) spectrum of ethylene glycol.

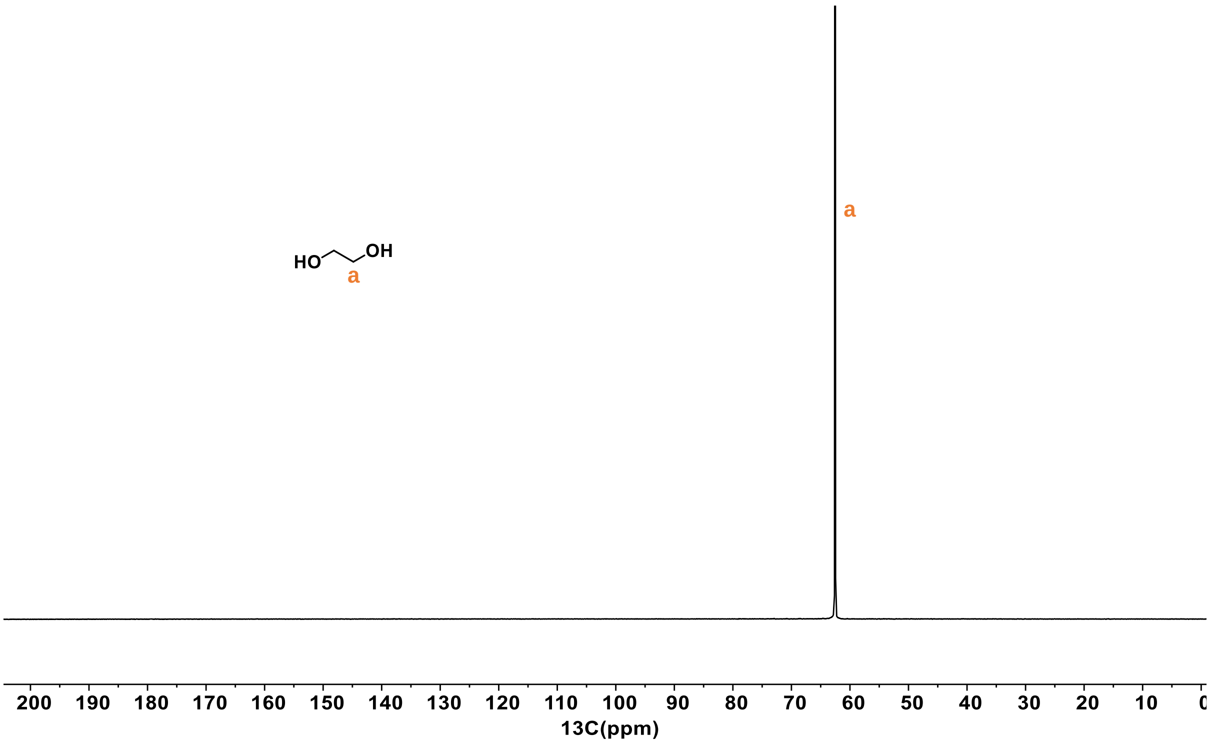

Figure S4. $^{13}C\{^{1}H\}$ NMR (126 MHz, D_2O) spectrum of ethylene glycol.

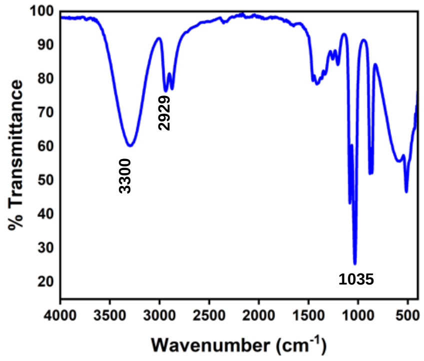

Figure S5. ATR-FT-IR spectrum of ethylene glycol.

1.7 GC-TCD analysis of the reaction product

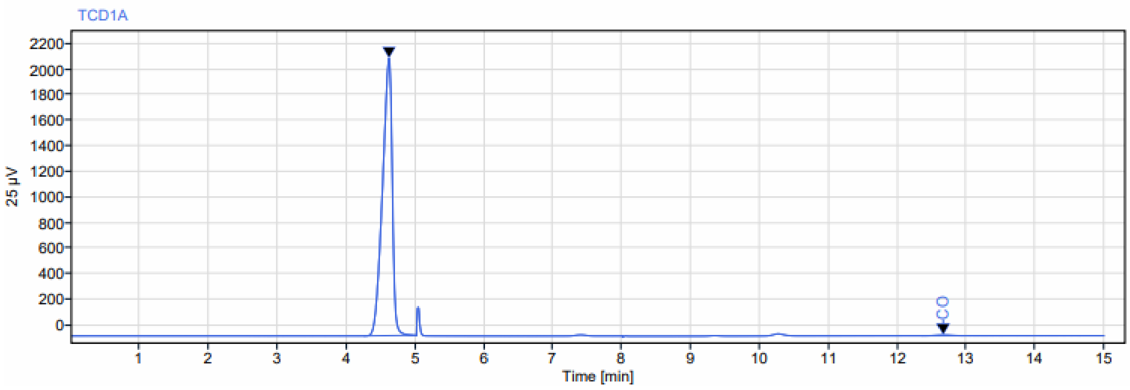

Signal: TCD1A

RT [min]	Type	Width [min]	Area	Height	Area%	Name
4.613	BM m	0.73	21350.61	2163.37	99.61	H_2
12.657	BV	0.60	83.01	5.25	0.39	CO
		**Sum**	**21433.62**			

Figure S6. GC-TCD analysis of the gas released from the reaction conducted as per the method described in Table S1, entry 13.

H_2 released= 99.6%

CO released= 0.4%

1.8 Characterisation of isolated product

The following data were obtained for the product isolated from the reaction conducted as per the conditions described in Table S1, entry 13.

Yield of the polymer= 99.8 mg (80.4%)

Ester: Ether = 4.8:1

^{1}H NMR (500 MHz, D_2O): δH 4.4 (br,O-**CH_2**-CO-), 4.2-4.0 (br,CO-O-**CH_2**-CH_2), 3.8 (br, CH_2-**CH_2**-O-).

^{13}C{^{1}H} NMR (126 MHz, D_2O): δC 176.3, 174.3, 164.1(-CH_2-**CO**-O), 72.2, 70.6,69.2 68.6,67.7, 66.6, 60.7 (-O-**CH_2**-CO-, CO-O-**CH_2**-CH_2), 61.8, 60.3,59.4 (-CH_2-**CH_2**-O-).

IR (ATR-FTIR, cm^{-1}): ν 3377 brm (O-H), 2980w (C-H), 1768s (C=O), 1201w, 1087m (C-O).

GPC: Mn =48,940 gmol^{-1} (Đ =1.4).

TGA Profile*:* Td= 250 °C.

DSC trace*:* Tg= -55.6 °C, Tm= 166.6 °C

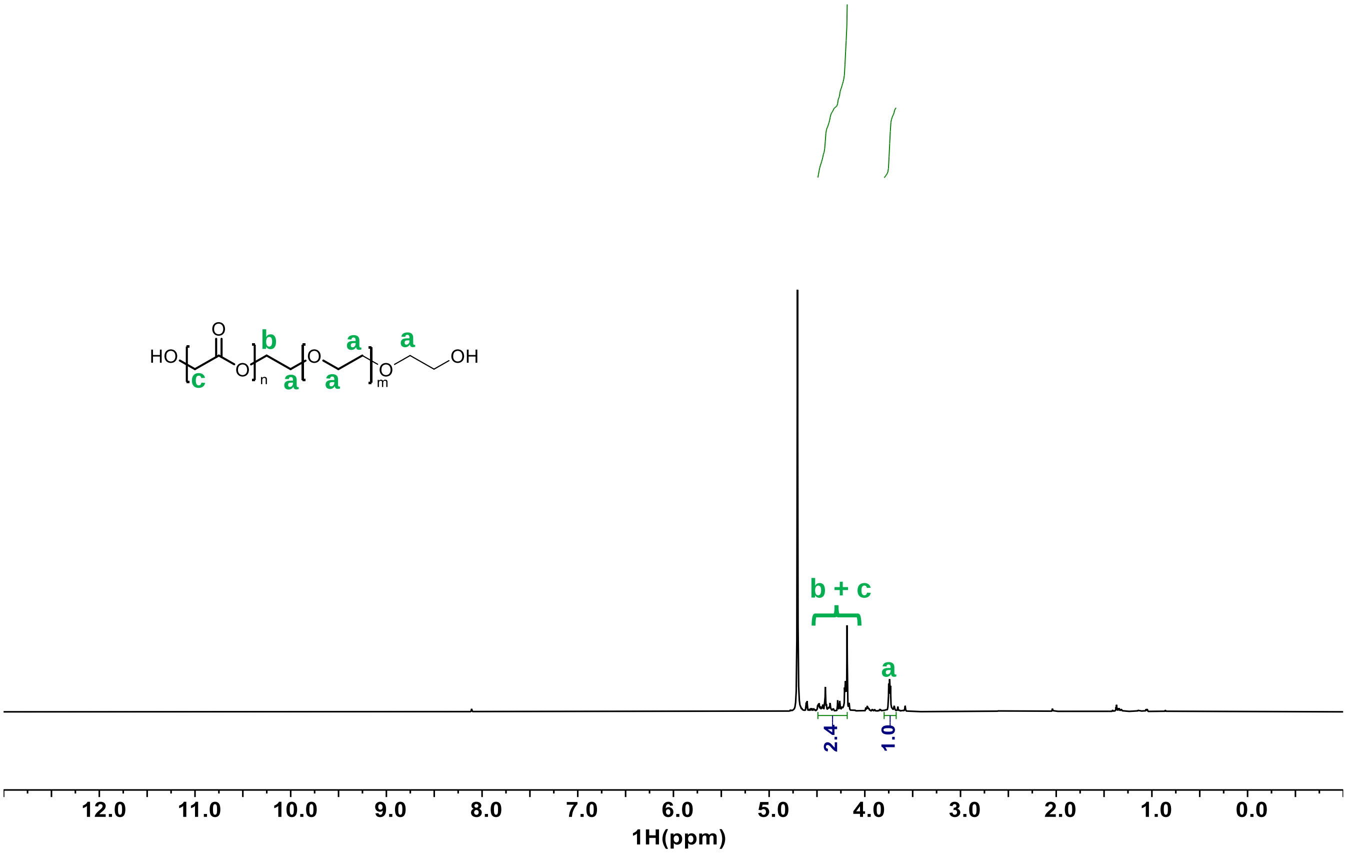

Figure S7. ^{1}H NMR (500 MHz, D_2O) spectrum of the isolated product from the polymerisation process conducted as per the method described in Table S1, entry 13.

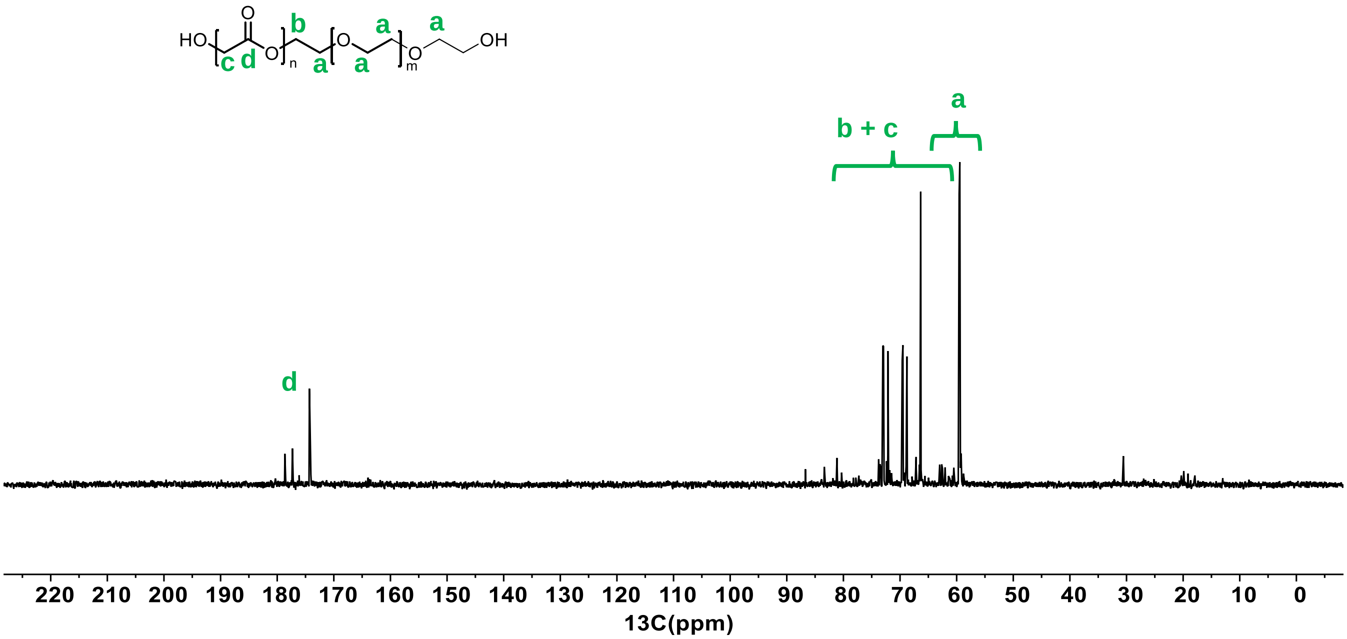

Figure S8. $^{13}C\{^{1}H\}$ NMR (126 MHz, D_2O) spectrum of the isolated product from the polymerisation process conducted as per the method described in Table S1, entry 13.

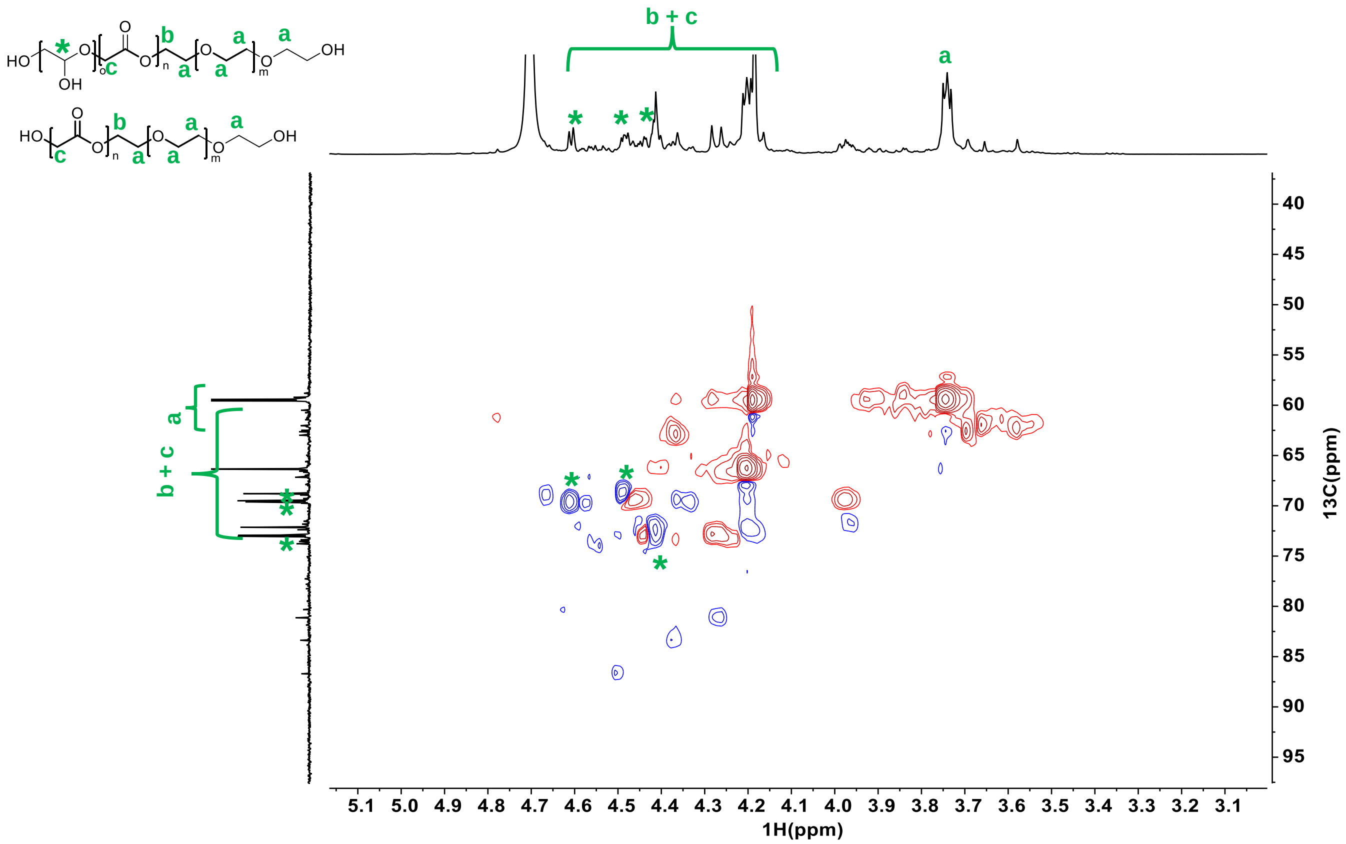

Figure S9. ^{1}H, ^{13}C- HSQC NMR (D_2O) spectrum of isolated product from the polymerisation process conducted as per the method described in Table S1, entry 13.

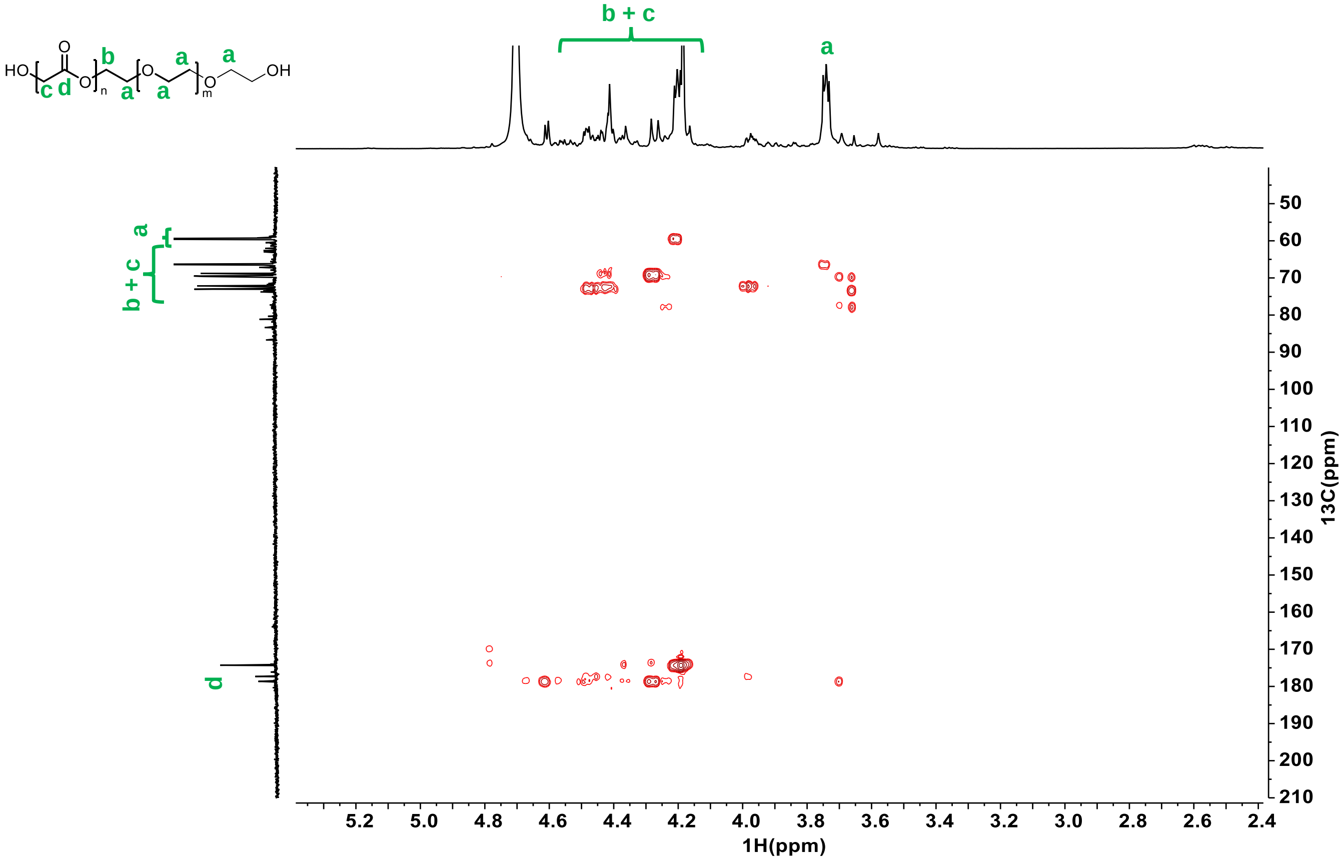

Figure S10. ^{1}H, ^{13}C- HMBC NMR (D_2O) spectrum of the isolated product from the polymerisation process conducted as per the method described in Table S1, entry 13.

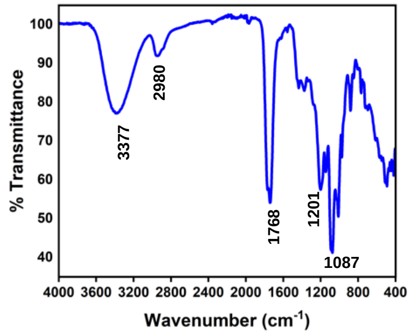

Figure S11. ATR-FTIR spectrum of the isolated product from the polymerisation process conducted as per the method described in Table S1, entry 13.

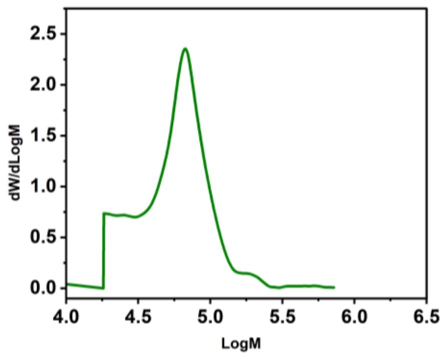

Figure S12. GPC data of the isolated product from the polymerisation process conducted as per the method described in Table S1, entry 13.

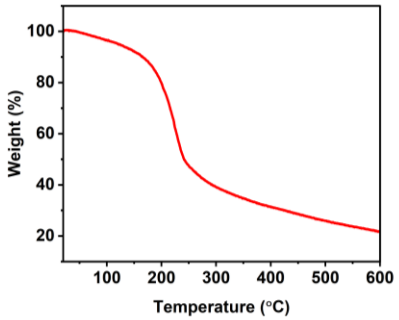

Figure S13. TGA Profile of the isolated product from the polymerisation process conducted as per the method described in Table S1, entry 13.

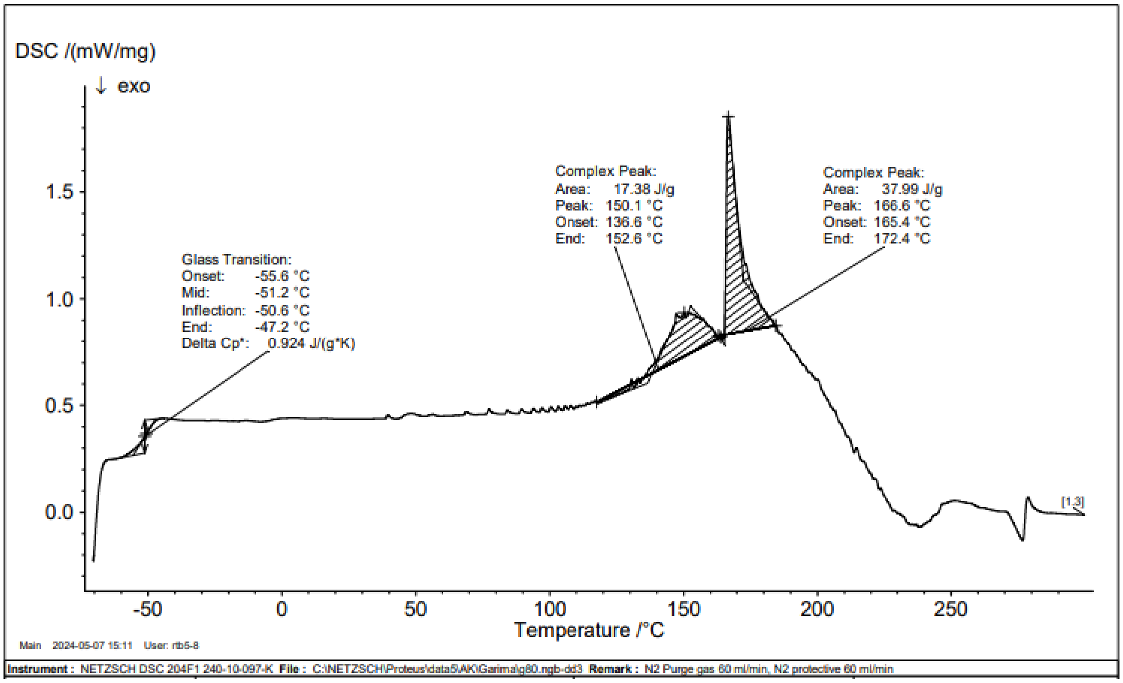

Figure S14. DSC trace of the isolated product from the polymerisation process conducted as per the method described in Table S1, entry 13.

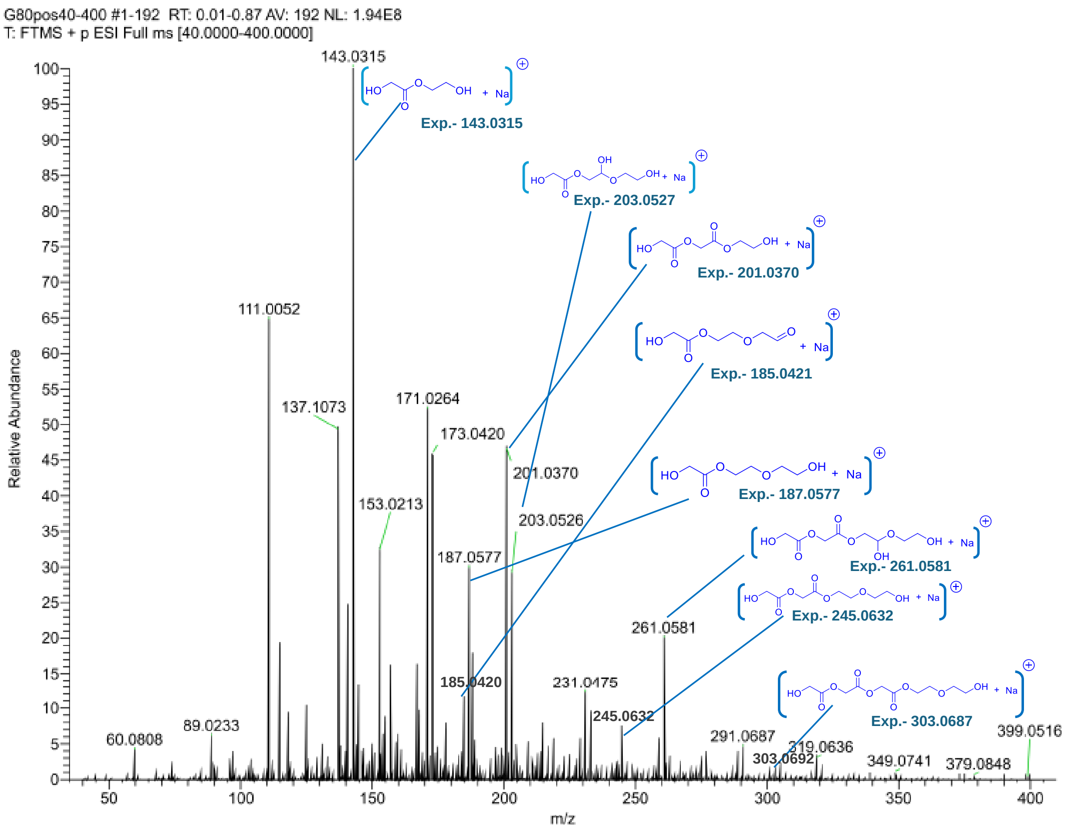

Figure S15. ESI- MS of the isolated product from the polymerisation process conducted as per the method described in Table S1, entry 13.

1.9 Optimisation of Catalytic Conditions

Table S1. Optimisation of catalytic conditions for the polymerization ethylene glycol.[a]

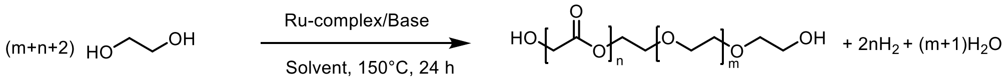

Entry no	Pre-catalyst	Base	Solvent	Time (h)	Gas released (mL)[b]	Conversion (%)[c]	Molar mass, Mn	Đ	Ester:Ether[d]
1	**Ru-1**	KO^{t}Bu	THF	24	24	63	38,020	1.3	3.6:1
2	**Ru-2**	KO^{t}Bu	THF	24	19	37	34,640	1.3	4.2:1
3	**Ru-3**	KO^{t}Bu	THF	24	3	14	-	-	1.8:1
4	**Ru-4**	KO^{t}Bu	THF	24	10	22	-	-	-
5	**Ru-5**	KO^{t}Bu	THF	24	49	89	34,450	1.3	4.6:1
6[e]	**Ru-5**	KO^{t}Bu	THF	24	10	20	-	-	-
7[f]	**Ru-5**	KO^{t}Bu	THF	24	22	47	15,640	1.5	3.8:1
8	**Ru-5**	KO^{t}Bu	H_2O	24	4	10	-	-	-
9	**Ru-5**	KO^{t}Bu	Diglyme	24	45	83	31,200	1.3	4.2:1
10	**Ru-5**	KO^{t}Bu	DME	24	56	91	11,770	2.6	5.6:1
11	**Ru-5**	KO^{t}Bu	DME	72	66	95	24,320	1.5	4.8:1
12[g]	**Ru-5**	KO^{t}Bu	Diglyme	24	-	81	32,680	1.4	4.2:1
13	**Ru-5**	KO^{t}Bu	THF	48	60	94	48,940	1.4	4.2:1
14[h]	**Ru-5**	KO^{t}Bu	THF	24	43	79	24,010	1.6	3.6:1
15	-	KO^{t}Bu	THF	24	1	1	-	-	-
16	**Ru-5**	K_2CO_3	THF	24	39	87	29,130	1.5	3.0:1
17	**Ru-5**	KOH	THF	24	34	84	33,790	1.4	2.8:1
18	**Ru-5**	KBH_4	THF	24	21	55	17,370	1.6	3.4:1
19	**Ru-5**	-	THF	24	3	7	-	-	-
20.[i]	**Ru-5**	KO^{t}Bu	THF	48	46	83	55,330	1.2	5.6:1
21.	**Ru-5**	KO^{t}Bu	-	24	1	2	-	-	-
22.[j]	**Ru-5**	KO^{t}Bu	THF	48	181	95	36,280	1.3	5.0:1
23.[k]	**Ru-5**	KO^{t}Bu	THF	24	37	78	33,005	1.4	3.6:1

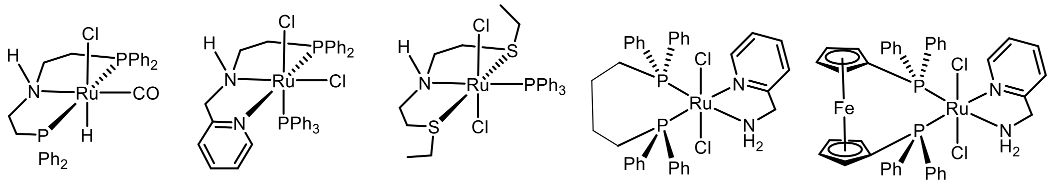

Ru-1 **Ru-2** **Ru-3** **Ru-4** **Ru-5**

[a]Reaction Conditions: Ethylene Glycol: 2 mmol, solvent: 2 mL, catalyst (1 mol%), base (2 mol%). [b]In all cases released gas was identified to be mainly H_2 with ≤1% of CO using GC-TCD analysis. [c]Conversion was determined by the ^1H NMR spectroscopy of the crude reaction mixture using ethylene carbonate as an internal standard and D_2O as solvent. [d]Ester, ether ratio were estimated by ^1H NMR spectroscopy (see ESI for more details). [e]Reaction conducted at 100 °C. [f]0.5 mol% catalyst is used. [g]Reaction performed under open flow of argon. [h]10 mol% KO^{t}Bu was used. [i]Reaction with non - anhydrous ethylene glycol. [j]Reaction at large scale- Ethylene Glycol: 5 mmol, solvent: 5 mL, catalyst (2.5 mol%), base (5 mol%). [k] Reaction done in the presence of molecular sieves.

Table S1; Entry 1:

^{1}H NMR (500 MHz, D_2O): δH 8.3 (O-H), 4.4-4.0 (br,O-**CH_2**-CO-,CO-O-**CH_2**-CH_2), 3.8 (br, CH_2-**CH_2**-O-).

$^{13}C\{^1H\}$ NMR (126 MHz, D_2O): δC 176.3, 174.3, 164.1(-CH_2-**CO**-O), 72.2, 70.6,69.2 68.6,67.7, 66.6, 60.7 (-O-**CH_2**-CO-, CO-O-**CH_2**-CH_2), 62.1, 60.2,59.4 (-CH_2-**CH_2**-O-).

GPC: Mn =38,020 $gmol^{-1}$ (Đ=1.3).

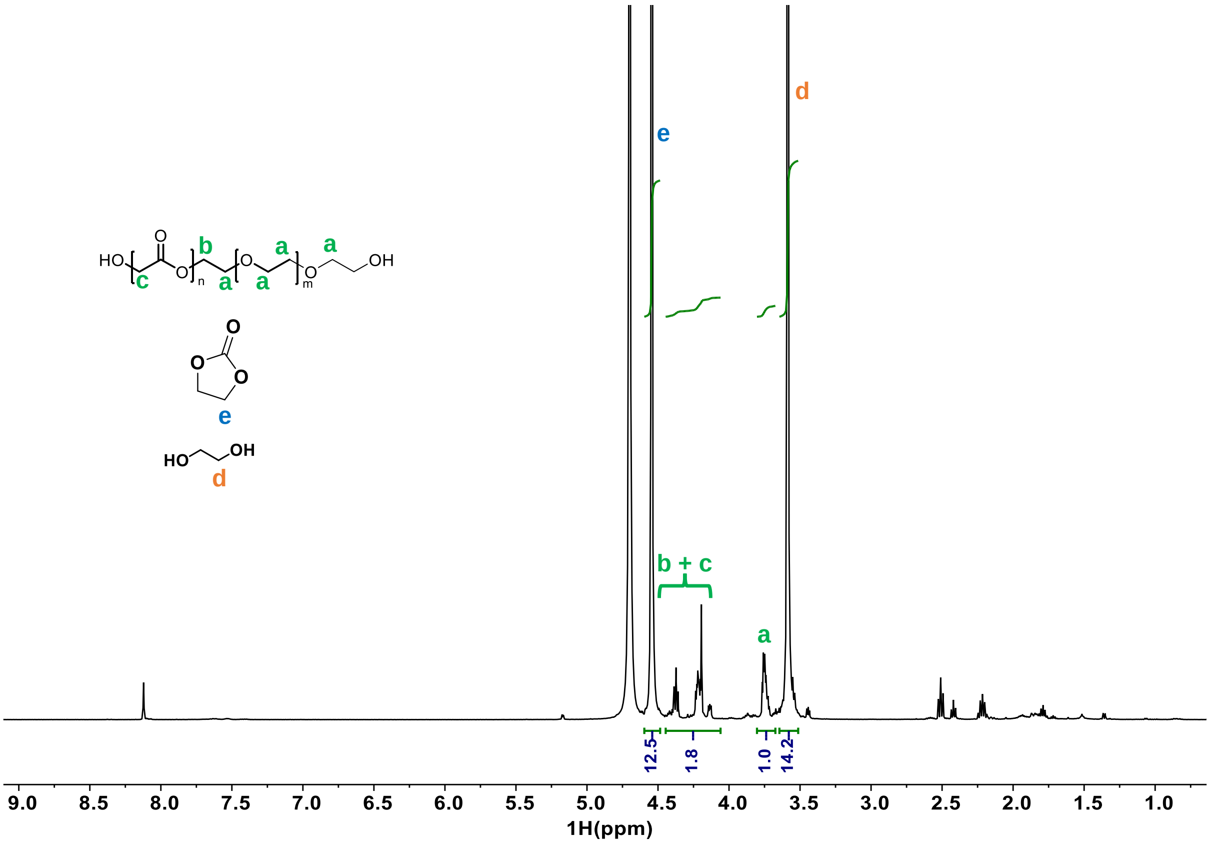

Figure S16. ^{1}H NMR (500 MHz, D_2O) spectrum of the reaction mixture resulting from the reaction corresponding to Table S1; Entry 1. Ethylene Carbonate (0.6 mmol) is used as an internal standard.

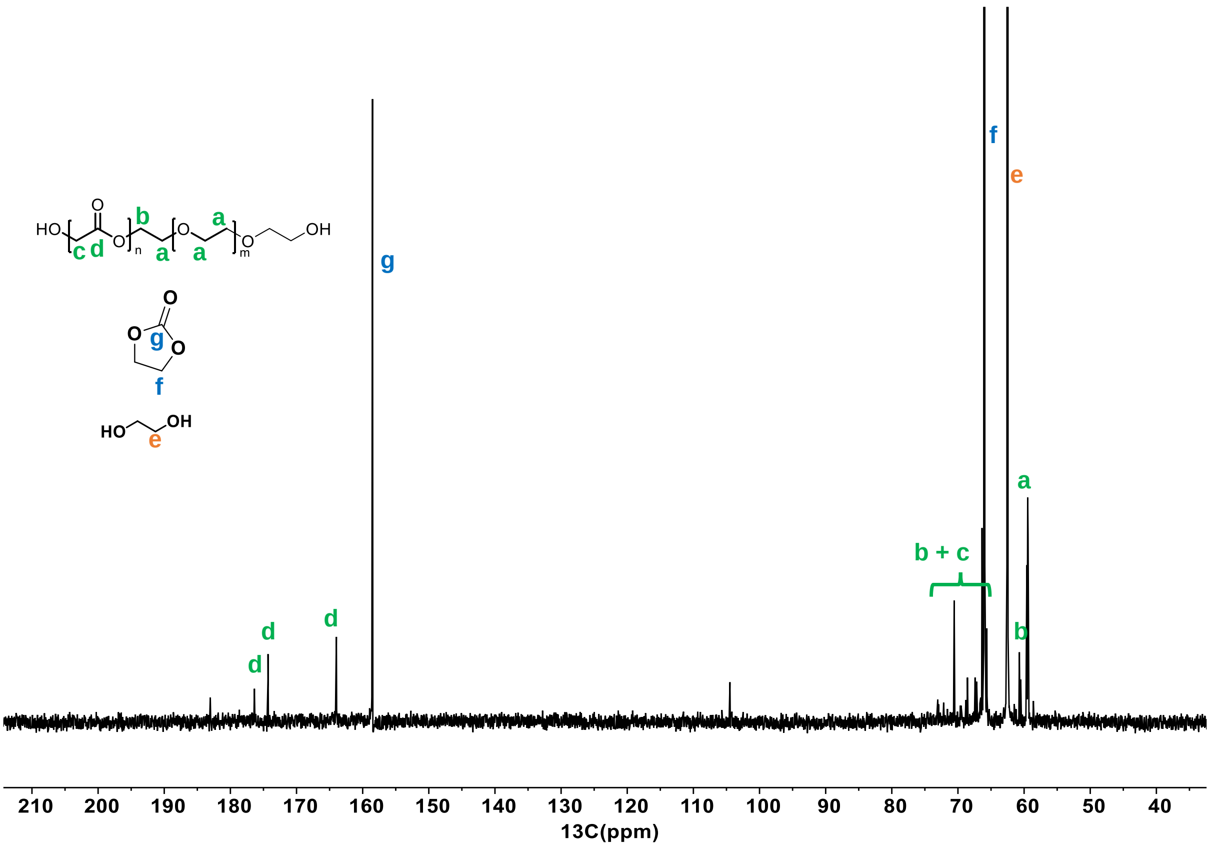

Figure S17. $^{13}C\{^{1}H\}$ NMR (126 MHz, D_2O) spectrum of the reaction mixture resulting from the reaction corresponding to Table S1; Entry 1. Ethylene Carbonate is used as an internal standard.

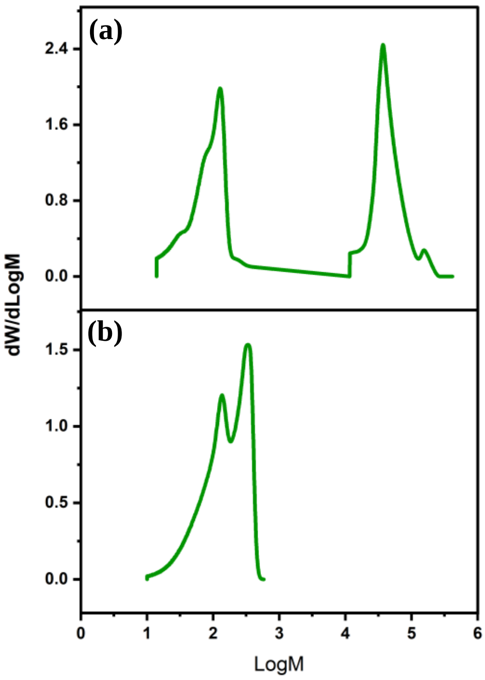

Figure S18. GPC data of the reaction mixture showing peaks of ethylene glycol left and the polymer formed in the reaction corresponding to Table S1; Entry 1(a) and GPC data of pure ethylene glycol(b).

Table S1; Entry 2:

^{1}H NMR (500 MHz, DMSO- d_6): δH 9.9(-**CHO**), 4.3-4.0 (br,O-**CH_2**-CO-,CO-O-**CH_2**-CH_2), 3.7 (br, CH_2-**CH_2**-O-).

^{13}C{^{1}H} NMR (126 MHz, DMSO- d_6): δC 206.7 (-**CHO**),173.1(-CH_2-**CO**-O),70.6,69.2 66.2,64.9, 60.0 (-O-**CH_2**-CO-, CO-O-**CH_2**-CH_2), 61.5, 60.8, 59.4, 55.3 (-CH_2-**CH_2**-O-).

GPC: Mn =34,640 gmo^{-1} (Đ =1.3).

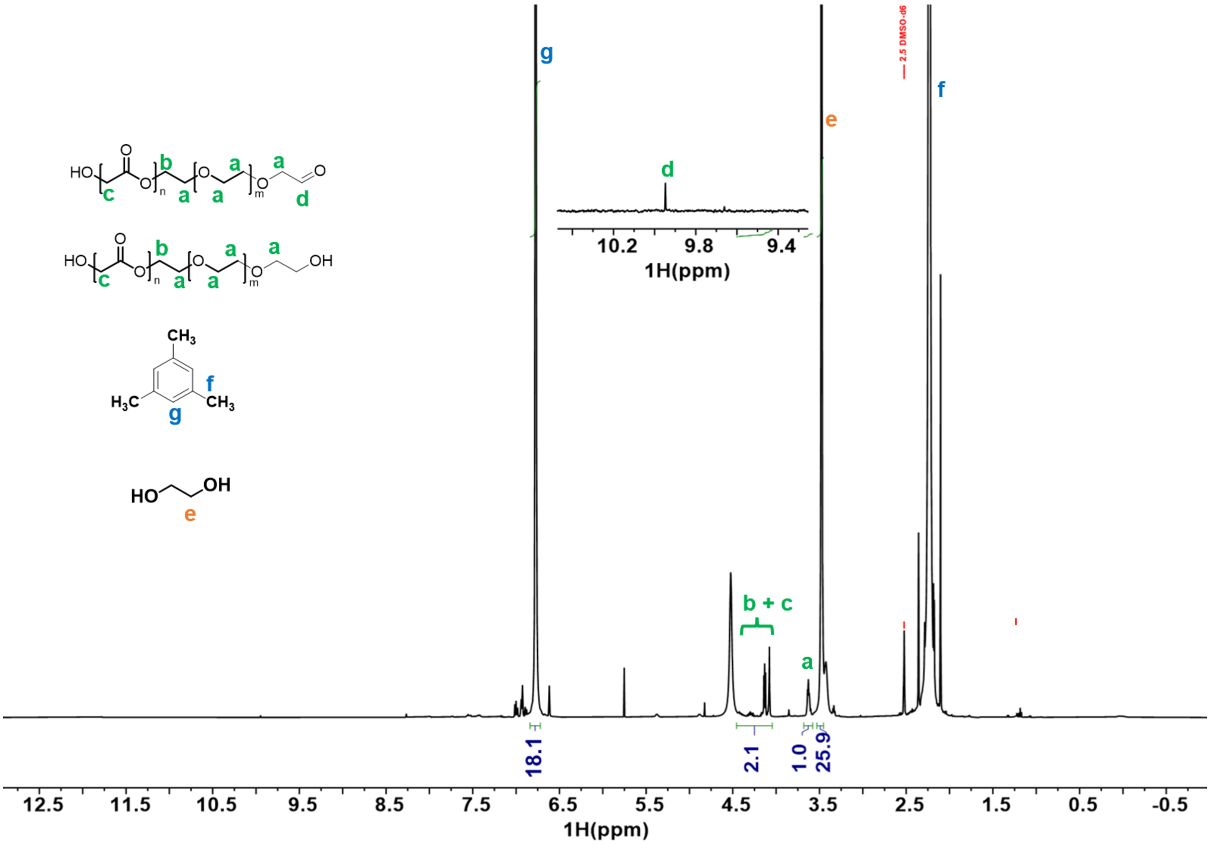

Figure S19. ^{1}H NMR (500 MHz, DMSO-d_6) spectrum of reaction mixture resulting from the reaction corresponding to Table S1; Entry 2. Mesitylene (1.1 mmol) is used as an internal standard.

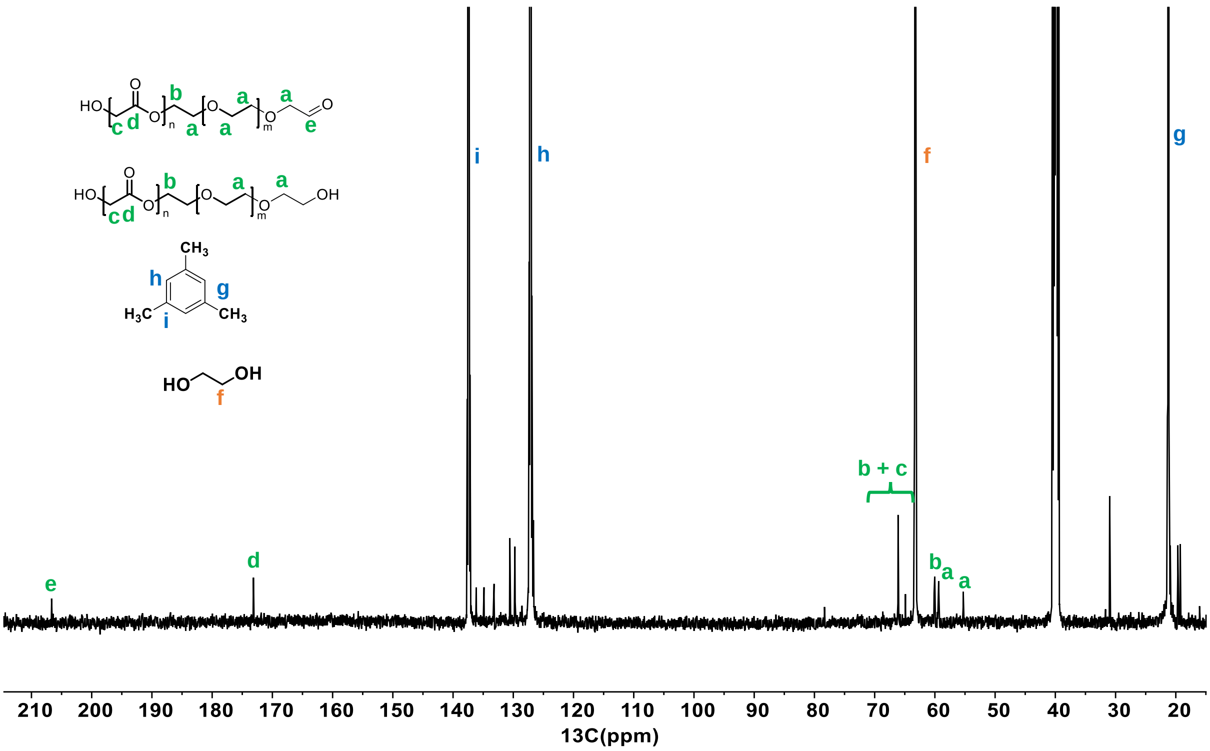

Figure S20. $^{13}C\{^1H\}$ NMR (126 MHz, DMSO-d_6) spectrum of reaction mixture resulting from the reaction corresponding to Table S1; Entry 2. Mesitylene is used as an internal standard.

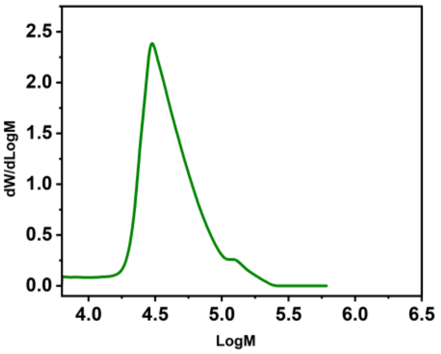

Figure S21. GPC data of the reaction mixture resulting from the reaction corresponding to Table S1; Entry 2.

Table S1; Entry 3:

^{1}H NMR (500 MHz, DMSO- d_6): δH 9.9(-**CHO**), 4.3-4.0 (br,O-**CH$_2$**-CO-,CO-O-**CH$_2$**-CH$_2$), 3.6 (br, CH$_2$-**CH$_2$**-O-).

$^{13}C\{^1H\}$ NMR (126 MHz, DMSO- d_6): δC 175(-CH$_2$-**CO**-O), 69.3, 66.1,64.7, 60.7 (-O-**CH$_2$**-CO-, CO-O-**CH$_2$**-CH$_2$), 61.7, 6 59.7 (-CH$_2$-**CH$_2$**-O-).

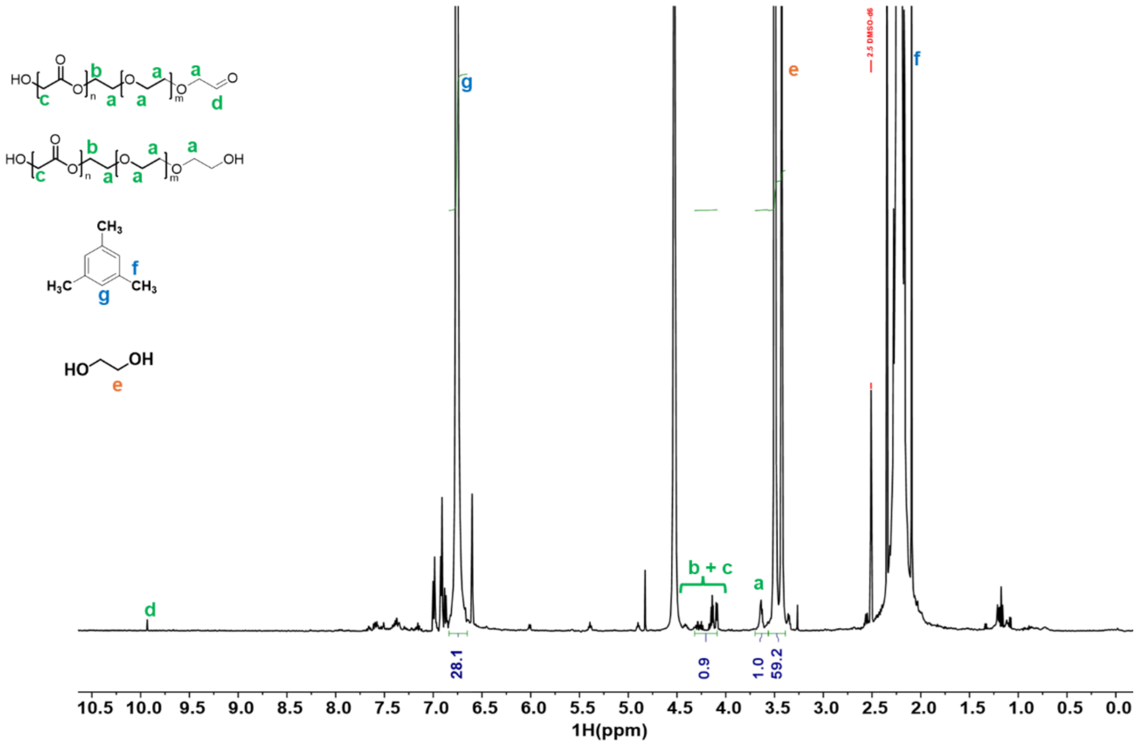

Figure S22. ^{1}H NMR (500 MHz, DMSO-d$_6$) spectrum of reaction mixture resulting from the reaction corresponding to Table S1; Entry 3. Mesitylene (1 mmol) is used as an internal standard.

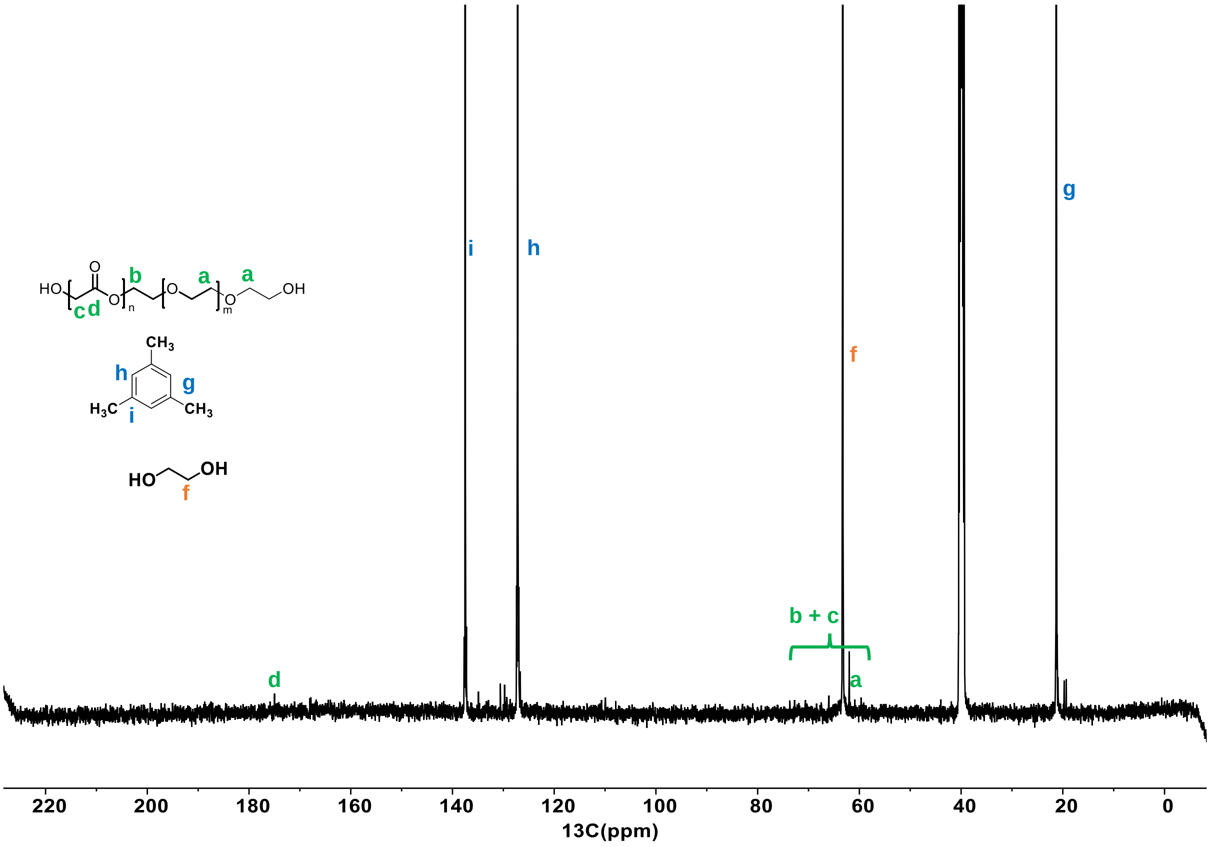

Figure S23. ^{13}C{^{1}H} NMR (126 MHz, DMSO-d$_6$) spectrum of reaction mixture resulting from the reaction corresponding to Table S1; Entry 3. Mesitylene is used as an internal standard.

Table S1; Entry 4

^{1}H NMR (500 MHz, D_2O): δH 4.4-4.0 (br,O-**CH_2**-CO-,CO-O-**CH_2**-CH_2), 3.6 (br, CH_2-**CH_2**-O-).

^{13}C{^{1}H} NMR (126 MHz, D_2O): δC 161.1(-CH_2-**CO**-O), 76.9, 68.5,65.5,64.8 (-O-**CH_2**-CO-, CO-O-**CH_2**-CH_2), 61.9, 61.0 (-CH_2-**CH_2**-O-).

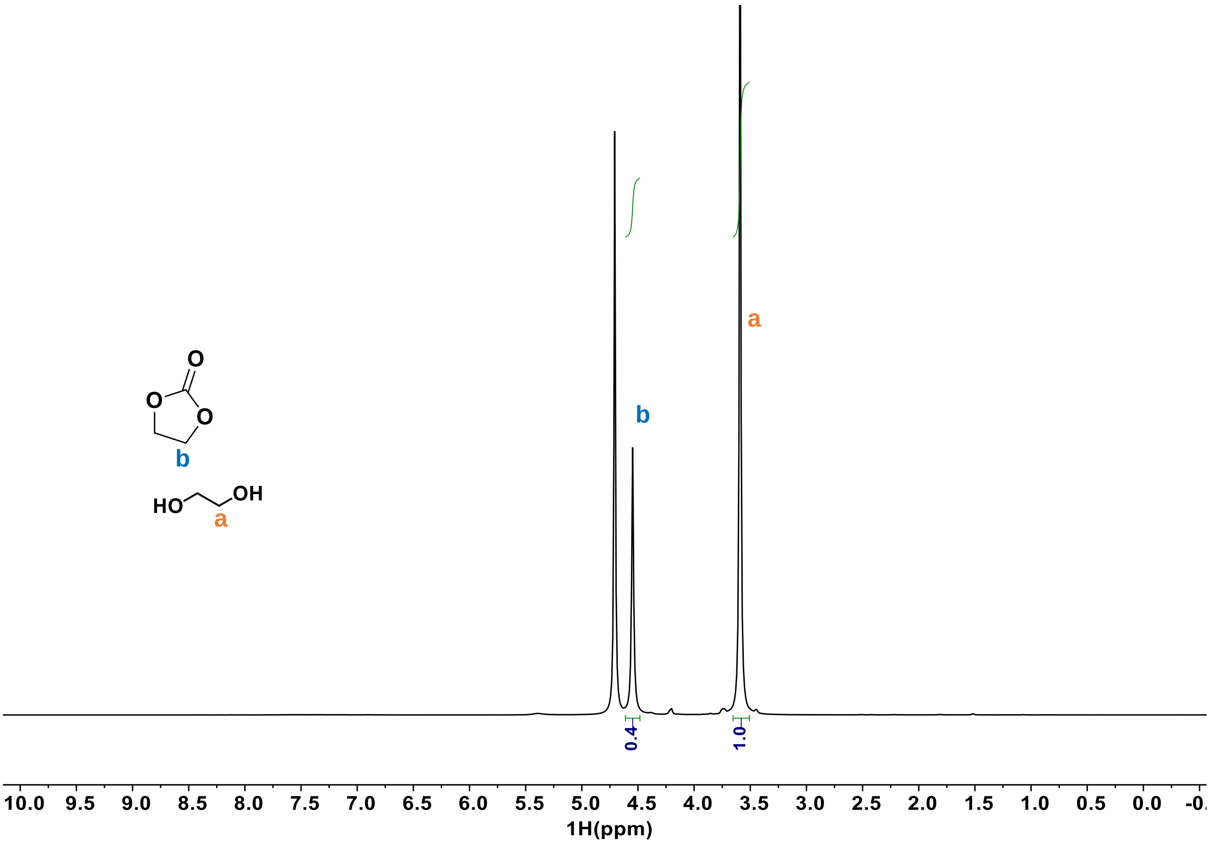

Figure S24. ^{1}H NMR (500 MHz, D_2O) spectrum of reaction mixture resulting from the reaction corresponding to Table S1; Entry 4. Ethylene Carbonate (0.6 mmol) is used as an internal standard.

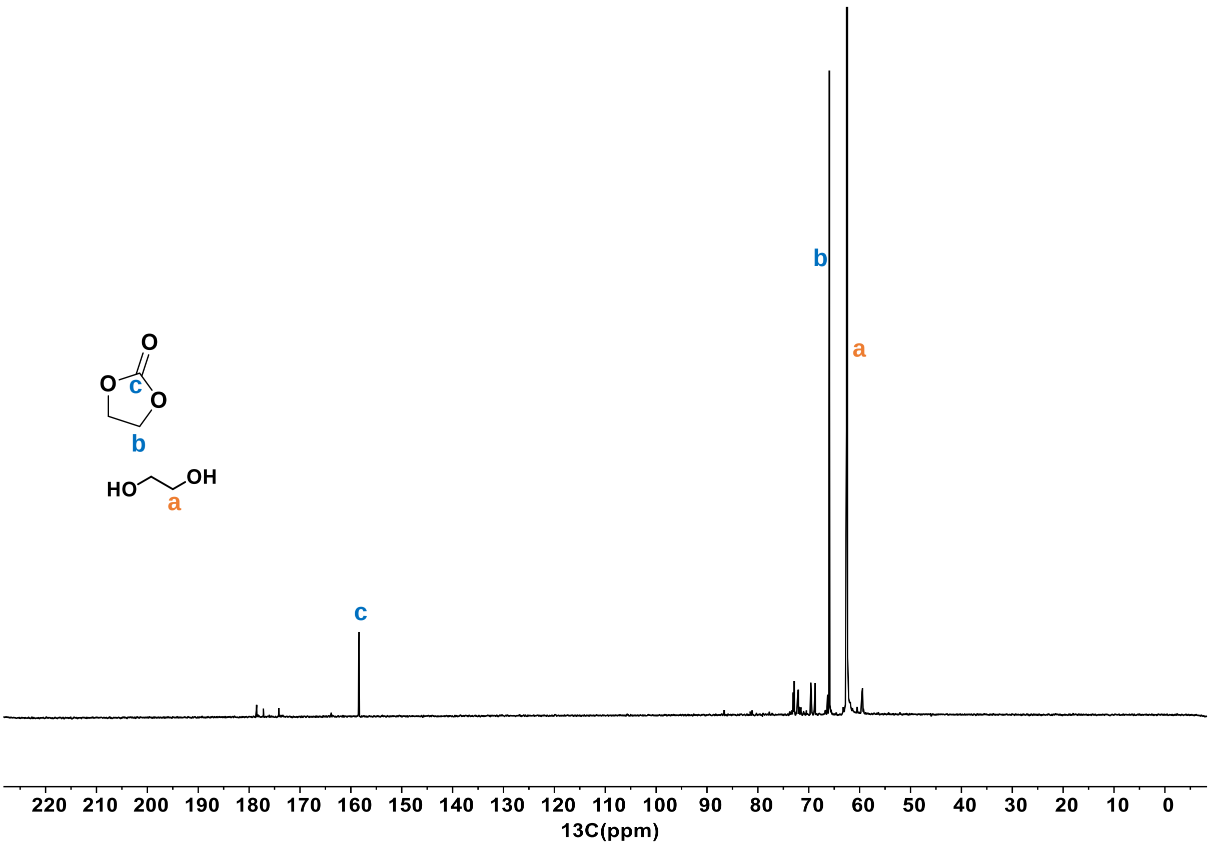

Figure S25. ^{13}C{^{1}H} NMR (126 MHz, D_2O) spectrum of reaction mixture resulting from the reaction corresponding to Table S1; Entry 4. Ethylene Carbonate is used as an internal standard.

Table S1; Entry 5

^{1}H NMR (500 MHz, D_2O): δH 4.4-4.0 (br,O-**CH_2**-CO-,CO-O-**CH_2**-CH_2), 3.6 (br, CH_2-**CH_2**-O-).

^{13}C{^{1}H} NMR (126 MHz, D_2O): δC 178.5,176.9,174.1(-CH_2-**CO**-O), 76.9, 68.5,66.4,65.5 (-O-**CH_2**-CO-, CO-O-**CH_2**-CH_2), 62.1, 61.0 (-CH_2-**CH_2**-O-).

GPC: Mn =34,450 $gmol^{-1}$ (Đ=1.3).

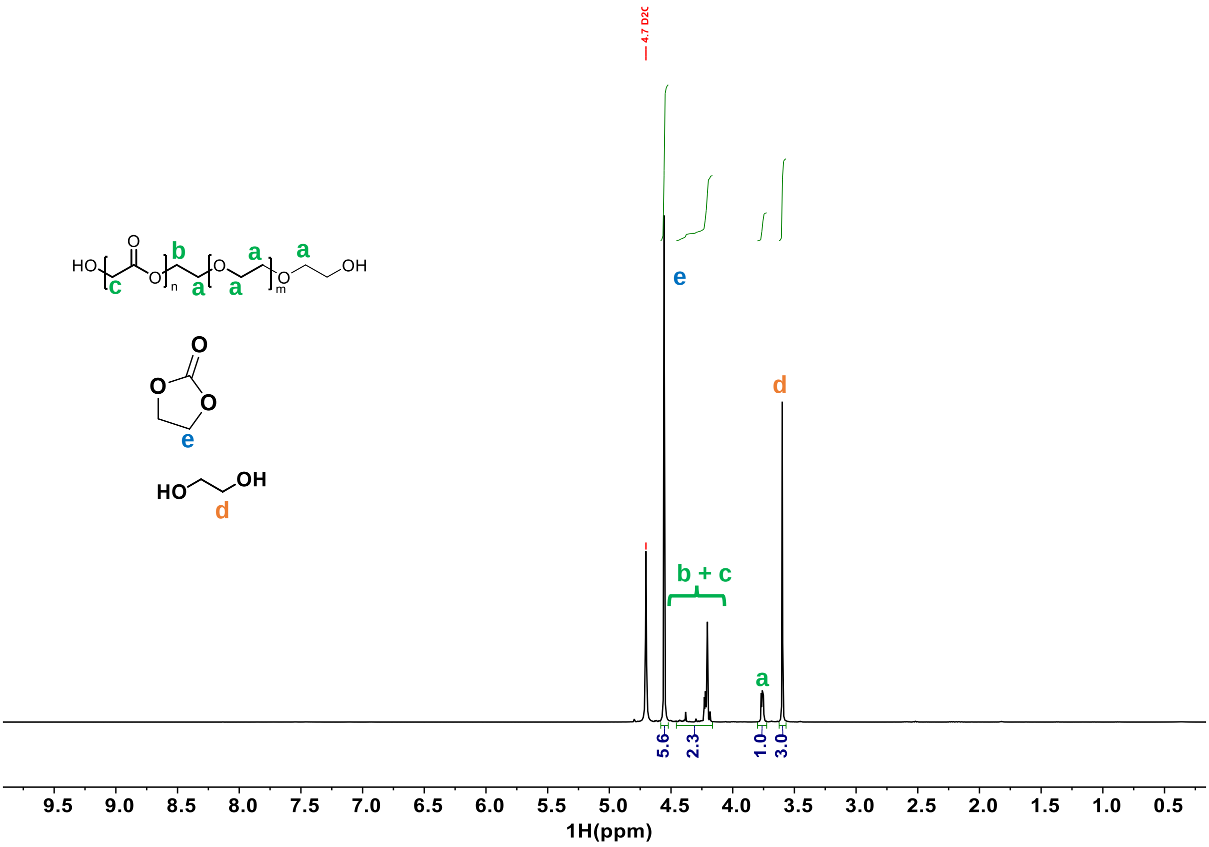

Figure S26. ^{1}H NMR (500 MHz, D_2O) spectrum of the reaction mixture resulting from the reaction corresponding to Table S1; Entry 5. Ethylene Carbonate (0.4 mmol) is used as an internal standard.

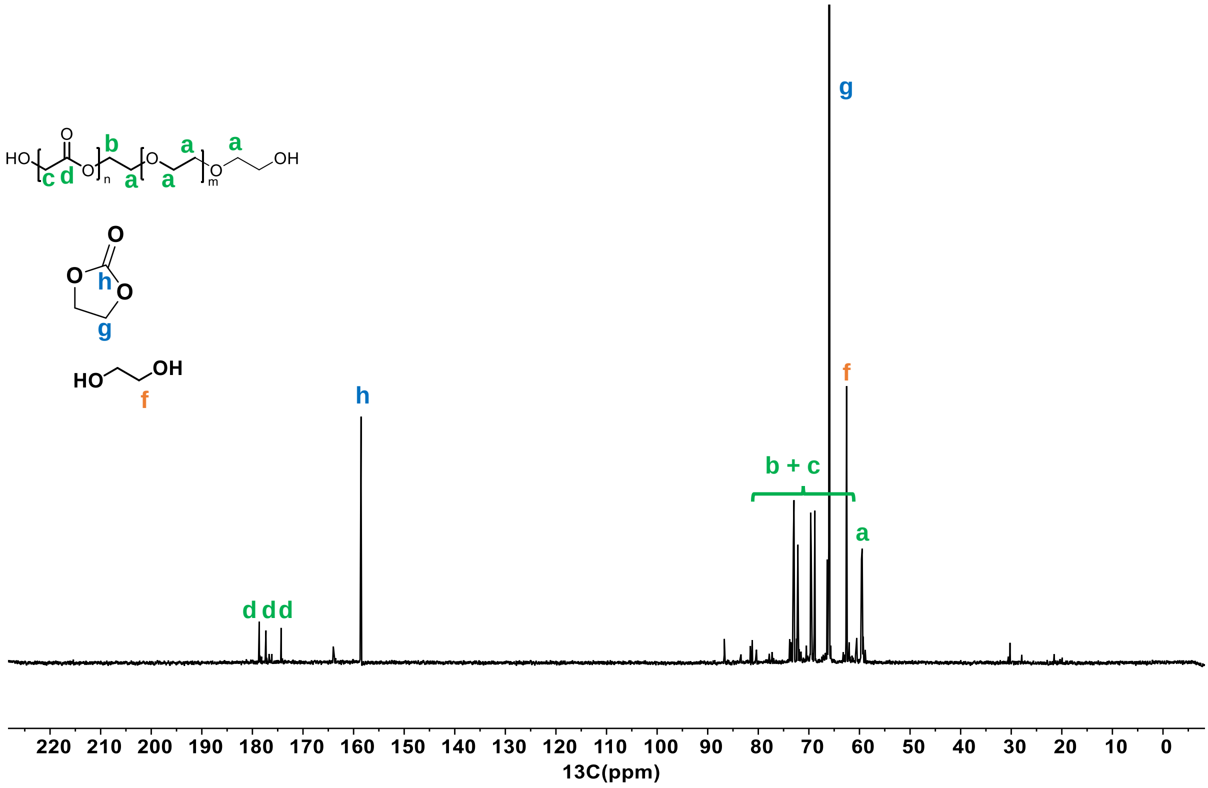

Figure S27. $^{13}C\{^{1}H\}$ NMR (126 MHz, D_2O) spectrum of the reaction mixture resulting from the reaction corresponding to Table S1; Entry 5. Ethylene Carbonate is used as an internal standard.

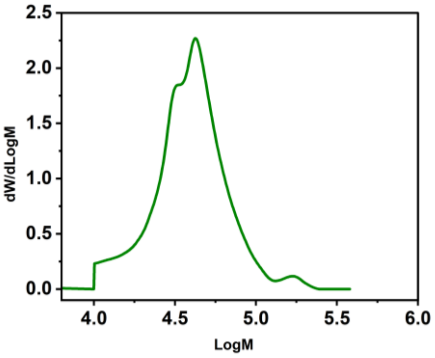

Figure S28. GPC data of the resulting mixture reaction mixture resulting from the reaction corresponding to Table S1; Entry 5.

Table S1; Entry 6

^{1}H NMR (500 MHz, D_2O): δH 4.4-4.0 (br,O-**CH_2**-CO-,CO-O-**CH_2**-CH_2), 3.6 (br, CH_2-**CH_2**-O-).

^{13}C{^{1}H} NMR (126 MHz, D_2O): δC 161.1(-CH_2-**CO**-O), 76.8, 68.1,66.4,65.8 (-O-**CH_2**-CO-, CO-O-**CH_2**-CH_2), 61.8, 61.1 (-CH_2-**CH_2**-O-).

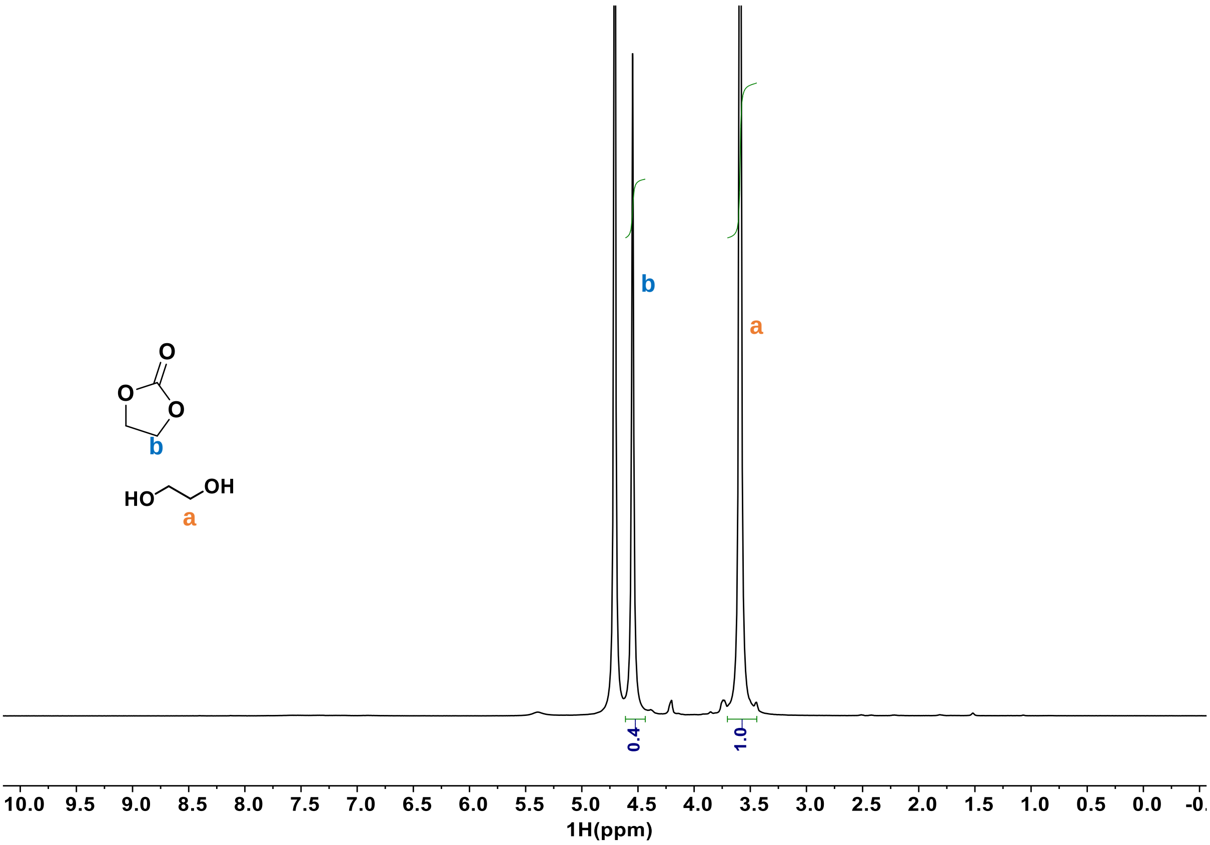

Figure S29. ^{1}H NMR (500 MHz, D_2O) spectrum of the reaction mixture resulting from the reaction corresponding to Table S1; Entry 6. Ethylene Carbonate (0.6 mmol) is used as an internal standard.

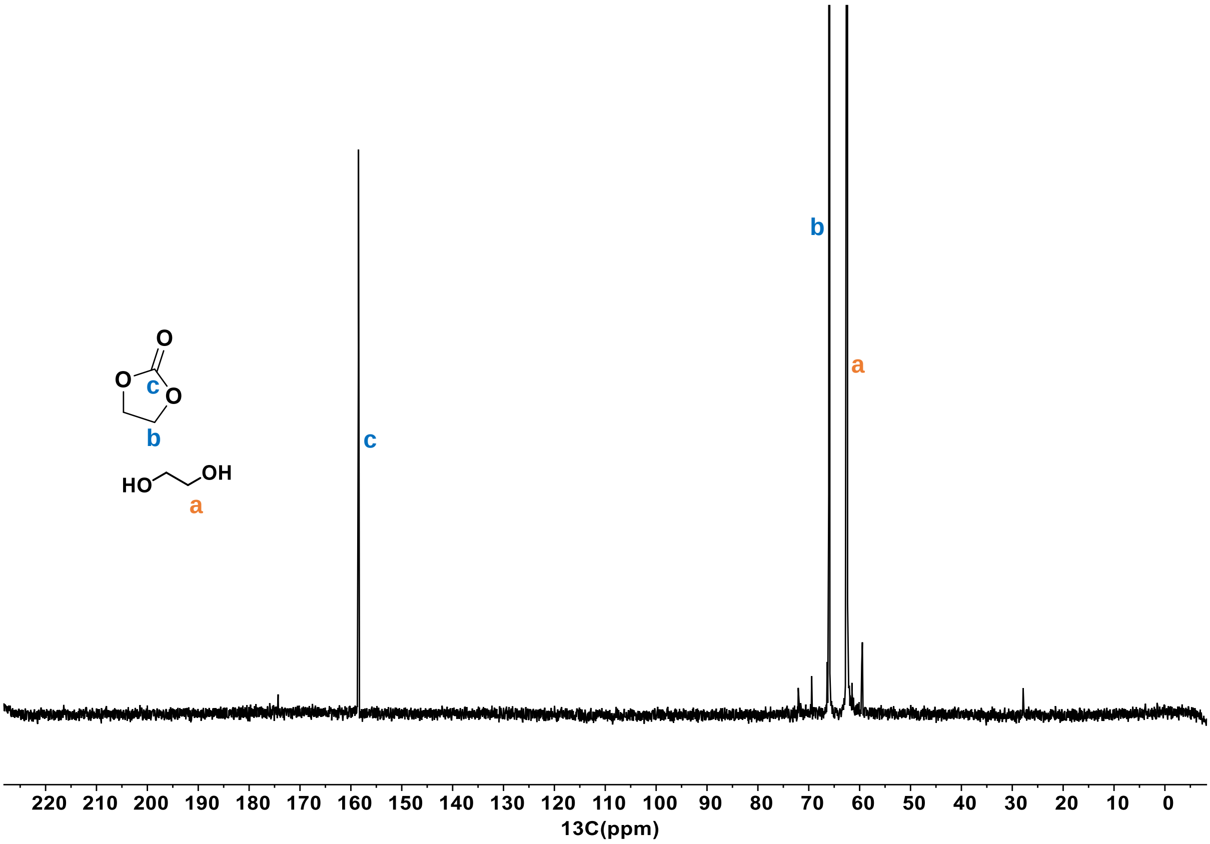

Figure S30. $^{13}C\{^1H\}$ NMR (126 MHz, D_2O) spectrum of the reaction mixture resulting from the reaction corresponding to Table S1; Entry 6. Ethylene Carbonate is used as an internal standard.

<u>Table S1; Entry 7:</u>

^{1}H NMR (500 MHz, D_2O): δH 8.3 (O-H), 4.4-4.0 (br,O-**CH_2**-CO-,CO-O-**CH_2**-CH_2), 3.6 (br, CH_2-**CH_2**-O-).

$^{13}C\{^1H\}$ NMR (126 MHz, D_2O): δC 174.3, 172.0, 164.0(-CH_2-**CO**-O), 72.9, 72.0,69.5 68.8,67.4 (-O-**CH_2**-CO-, CO-O-**CH_2**-CH_2), 61.7, 60.2,59.4 (-CH_2-**CH_2**-O-).

GPC: Mn =15,640 gmol^{-1} (Đ =1.5).

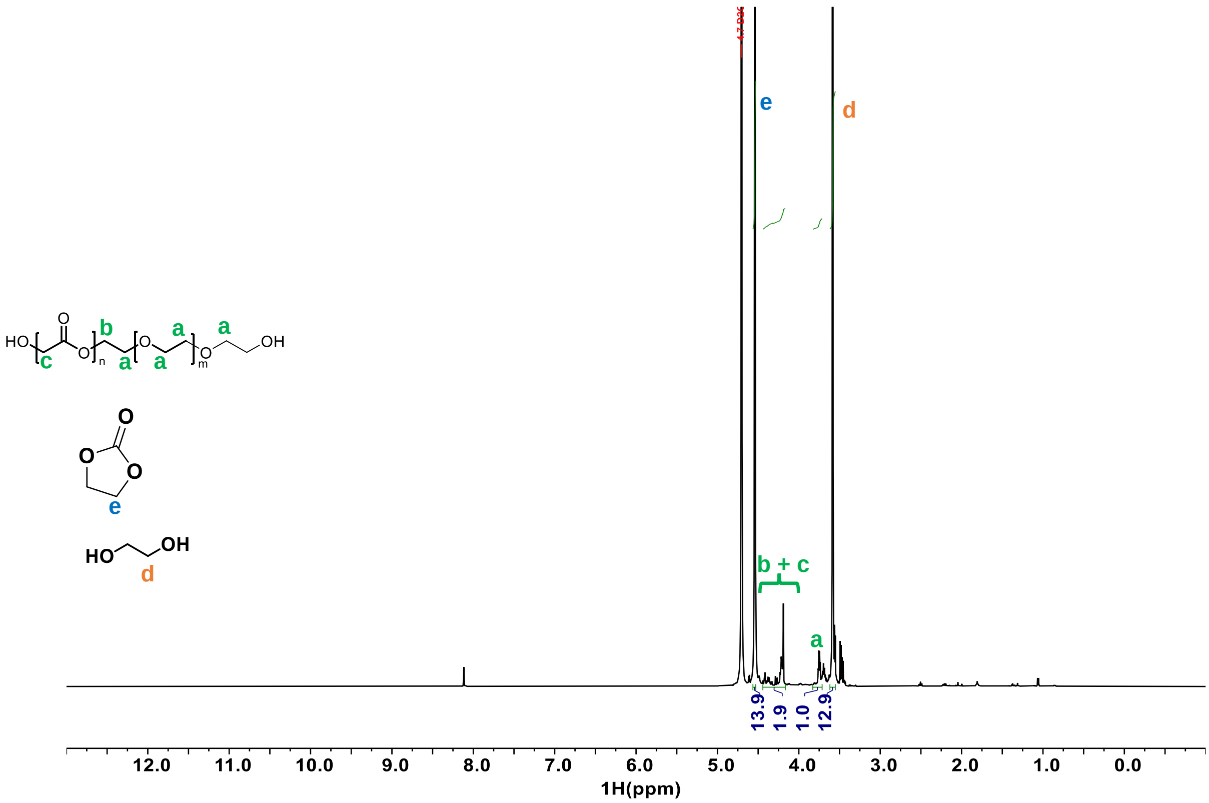

Figure S31. ^{1}H NMR (500 MHz, D_2O) spectrum of the reaction mixture resulting from the reaction corresponding to Table S1; Entry 7. Ethylene Carbonate (1.1 mmol) is used as an internal standard.

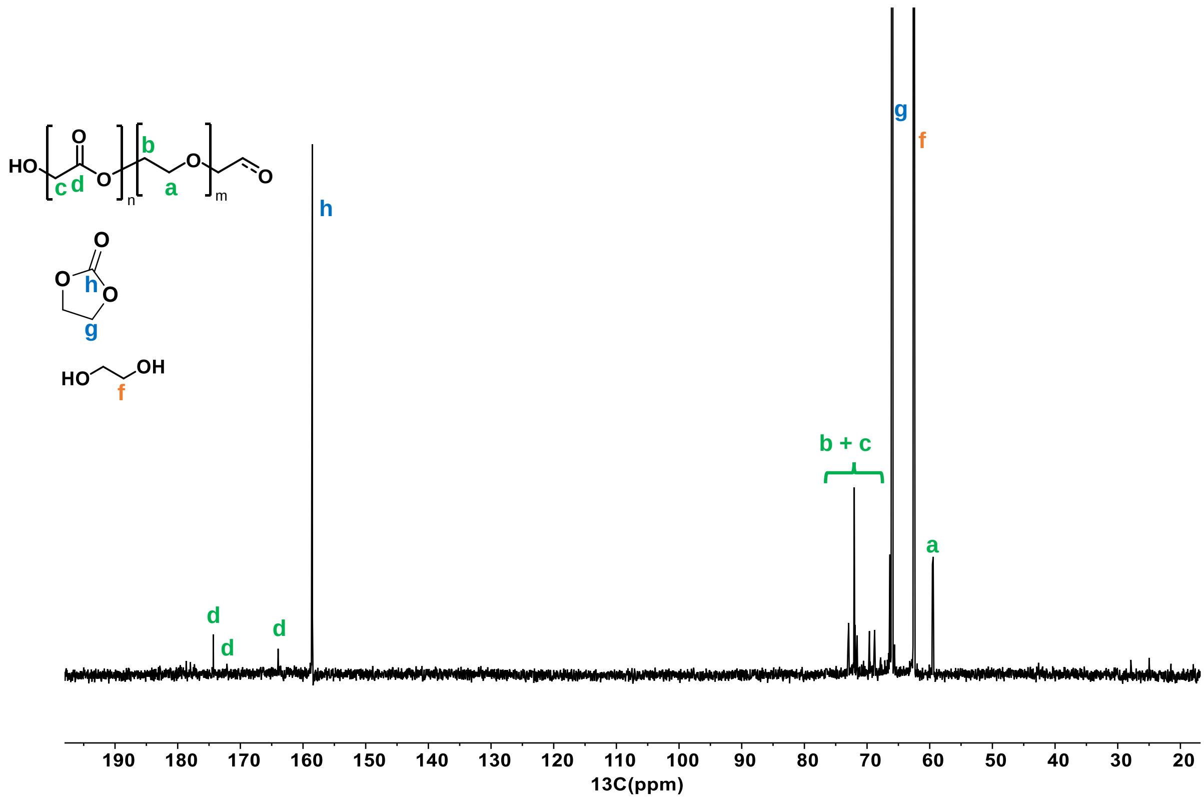

Figure S32. $^{13}C\{^{1}H\}$ NMR (126 MHz, D_2O) spectrum of the reaction mixture resulting from the reaction corresponding to Table S1; Entry 7. Ethylene Carbonate is used as an internal standard.

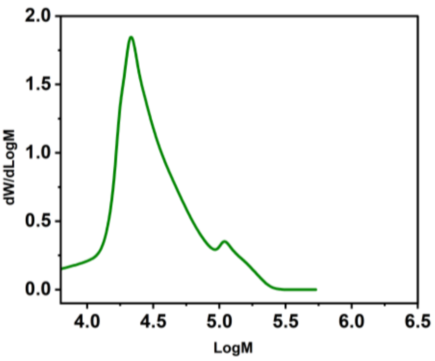

Figure S33. GPC data of the reaction mixture resulting from the reaction corresponding to Table S1; Entry 7.

<u>Table S1; Entry 8:</u>

^{1}H NMR (500 MHz, D_2O): δH 4.4-4.0 (br,O-**CH_2**-CO-,CO-O-**CH_2**-CH_2), 3.6 (br, CH_2-**CH_2**-O-).

^{13}C{^{1}H} NMR (126 MHz, D_2O): δC 216.3 (-**CHO**), 69.4 (-O-**CH_2**-CO-, CO-O-**CH_2**-CH_2), 59.5 (-CH_2-**CH_2**-O-).

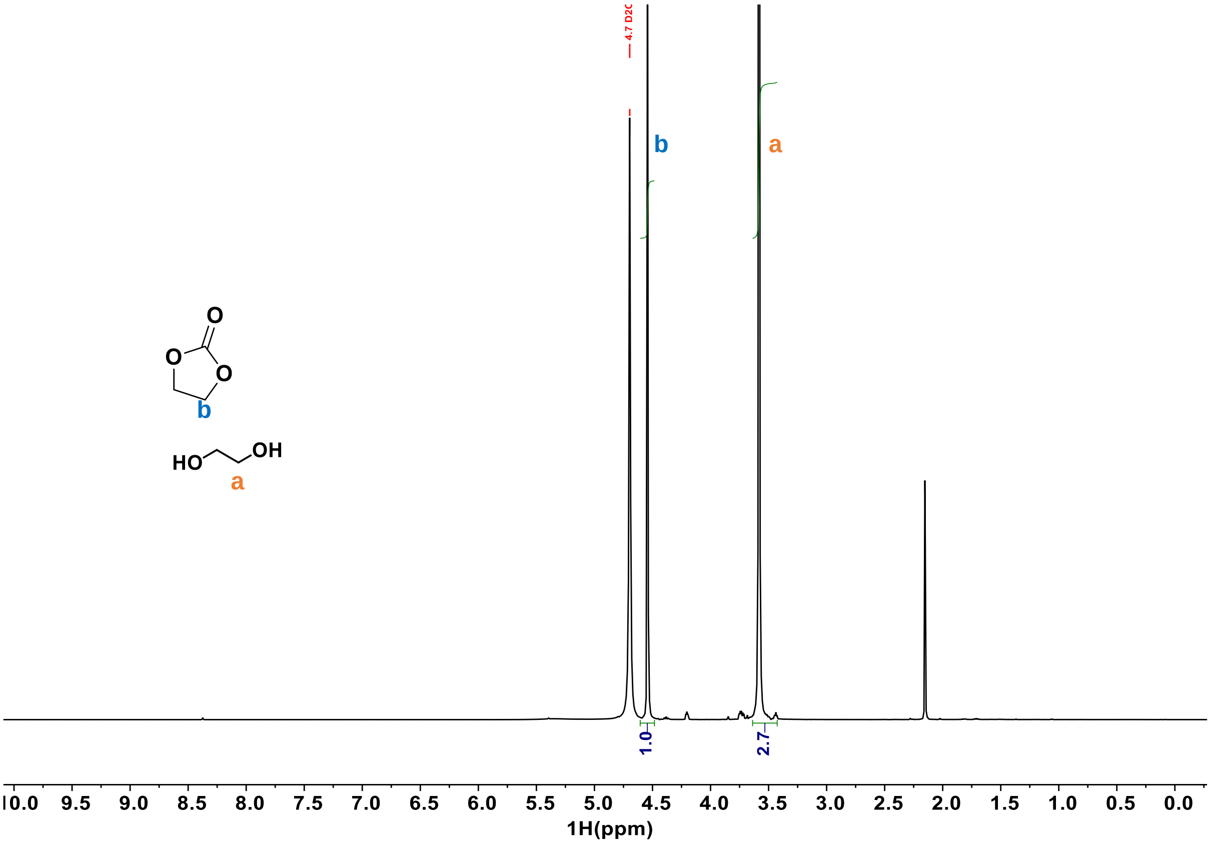

Figure S34. ^{1}H NMR (500 MHz, D_2O) spectrum of the reaction mixture resulting from the reaction corresponding to Table S1; Entry 8. Ethylene Carbonate (0.6 mmol) is used as an internal standard.

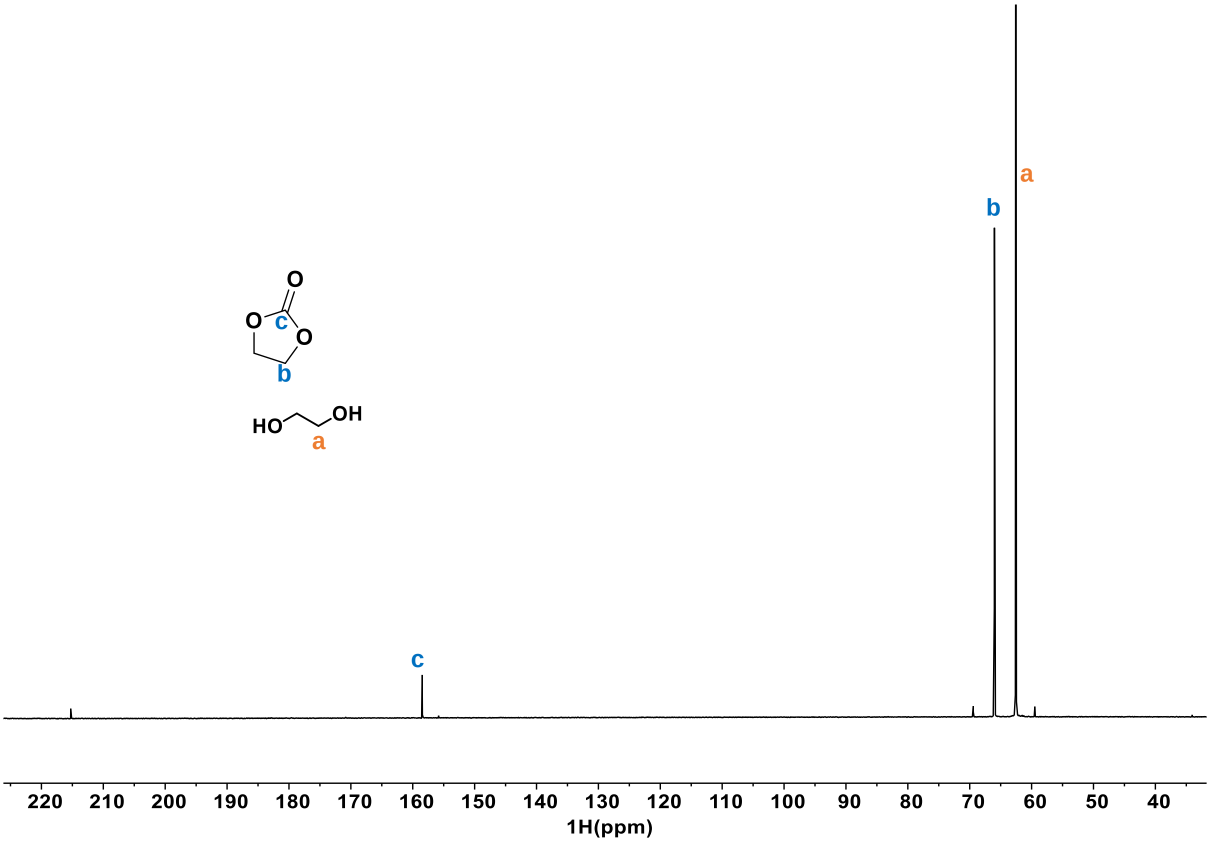

Figure S35. $^{13}C\{^{1}H\}$ NMR (126 MHz, D_2O) spectrum of the reaction mixture resulting from the reaction corresponding to Table S1; Entry 8. Ethylene Carbonate is used as an internal standard.

Table S1; Entry 9:

^{1}H NMR (500 MHz, D_2O): δH 8.3 (O-H), 4.4-4.3 (br,O-**CH_2**-CO-), 4.2-4.0 (br,CO-O-**CH_2**-CH_2), 3.7 (br, CH_2-**CH_2**-O-).

$^{13}C\{^{1}H\}$ NMR (126 MHz, D_2O): δC 172.9, 172.2, 163.9(-CH_2-**CO**-O), 73.2, 71.7, 71.1, 69.4, 68.8, 67.7,64.0, 60.4 (-O-**CH_2**-CO-, CO-O-**CH_2**-CH_2), 61.6, 60.5,59.4 (-CH_2-**CH_2**-O-).

GPC: Mn =31,200 $gmol^{-1}$ (Đ =1.3).

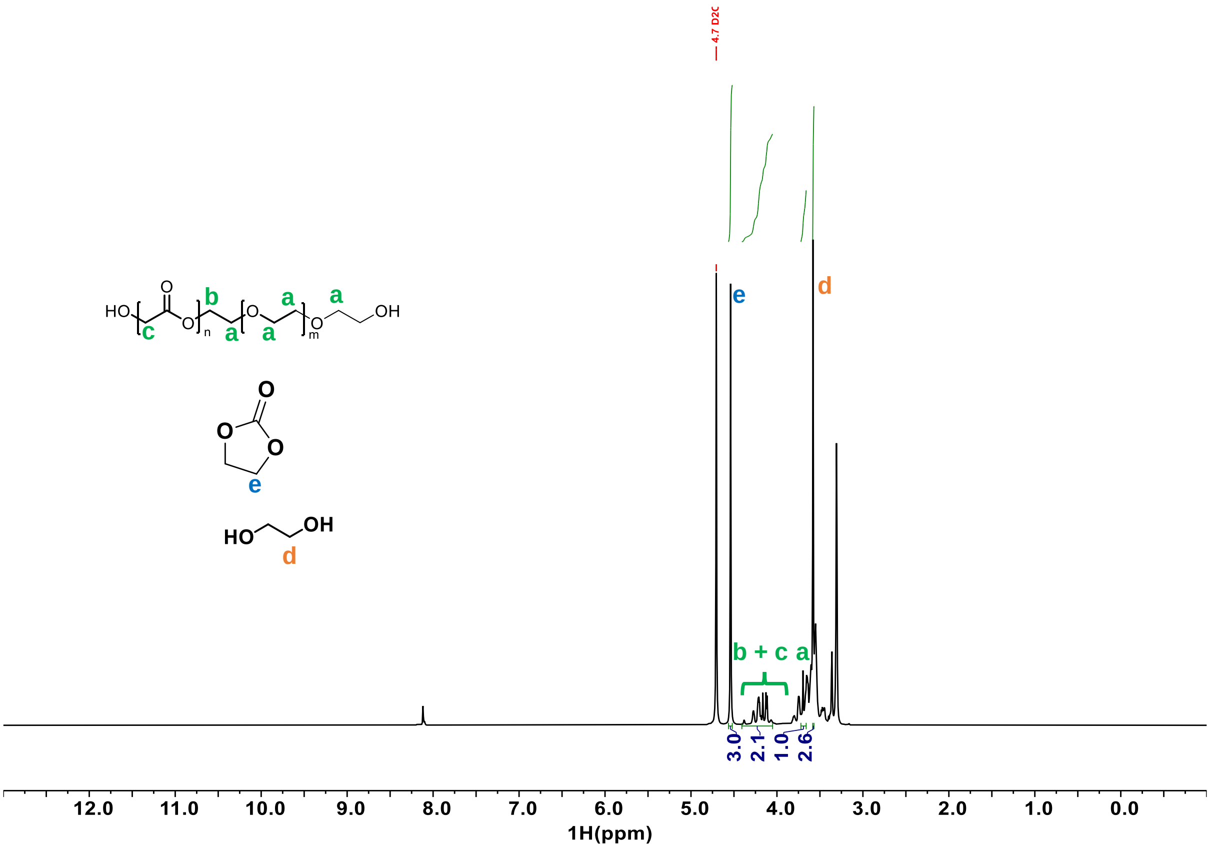

Figure 36. ^{1}H NMR (500 MHz, D_2O) spectrum of the reaction mixture resulting from the reaction corresponding to Table S1; Entry 9. Ethylene Carbonate (0.4 mmol) is used as an internal standard.

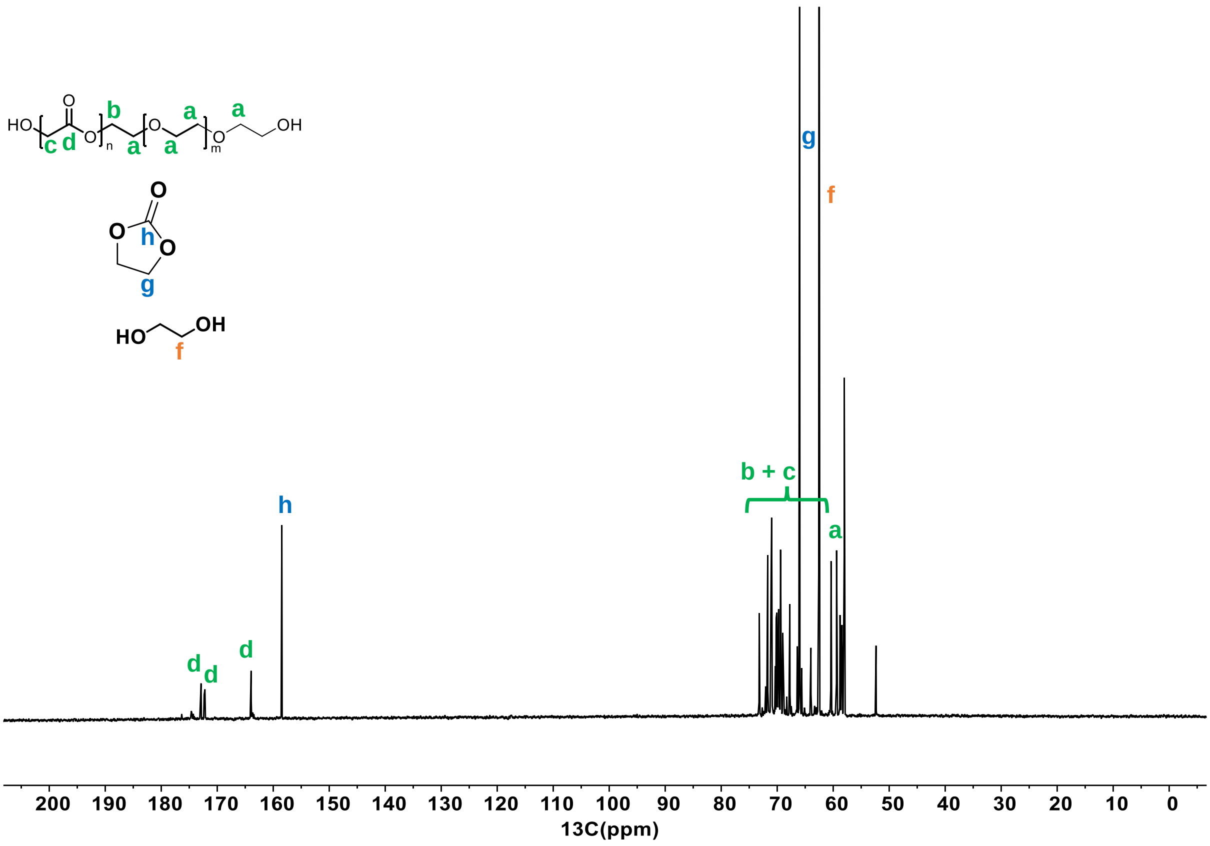

Figure S37. $^{13}C\{^1H\}$ NMR (126 MHz, D_2O) spectrum of the reaction mixture resulting from the reaction corresponding to Table S1; Entry 9. Ethylene Carbonate is used as an internal standard.

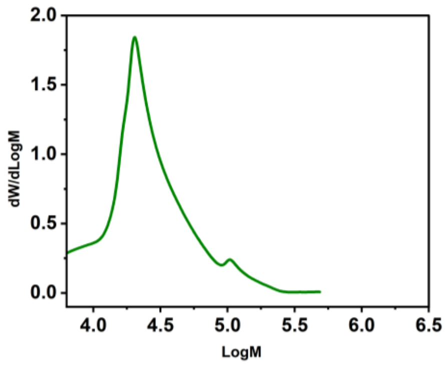

Figure S38. GPC data of the reaction mixture resulting from the reaction corresponding to Table S1; Entry 9.

Table S1; Entry 10:

^{1}H NMR (500 MHz, D_2O): δH 4.4-4.0 (br,O-**CH$_2$**-CO-,CO-O-**CH$_2$**-CH$_2$), 3.6 (br, CH$_2$-**CH$_2$**-O-).

$^{13}C\{^1H\}$ NMR (126 MHz, D_2O): δC 178.7, 177.3, 174.3, 164.0(-CH$_2$-**CO**-O), 73.1, 73.0, 72.2, 69.7, 68.8, 67.7, 60.9(-O-**CH$_2$**-CO-, CO-O-**CH$_2$**-CH$_2$), 61.4, 60.9,59.5 (-CH$_2$-**CH$_2$**-O-).

GPC: Mn =11,770 gmol^{-1} (Đ =2.6).

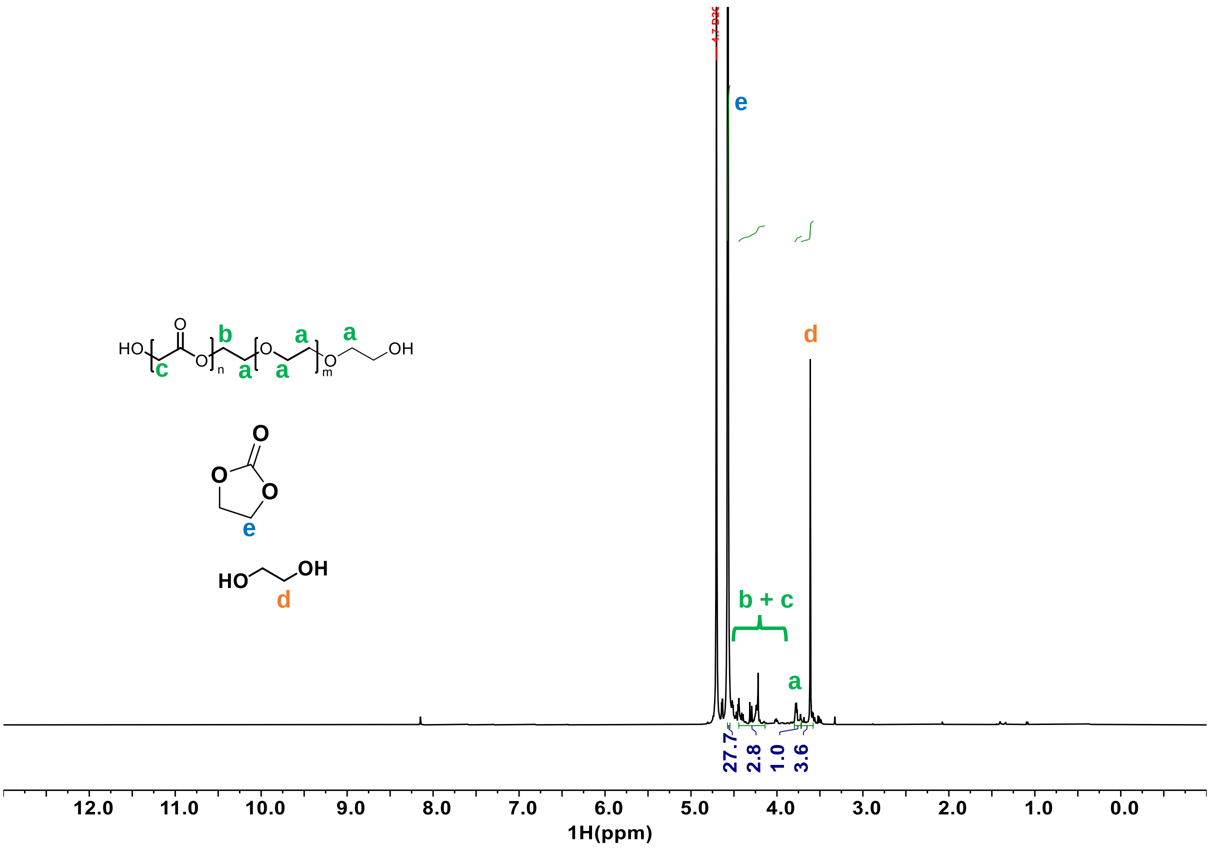

Figure S39. ^{1}H NMR (500 MHz, D_2O) spectrum of the reaction mixture resulting from the reaction corresponding to Table S1; Entry 10. Ethylene Carbonate (1.1 mmol) is used as an internal standard.

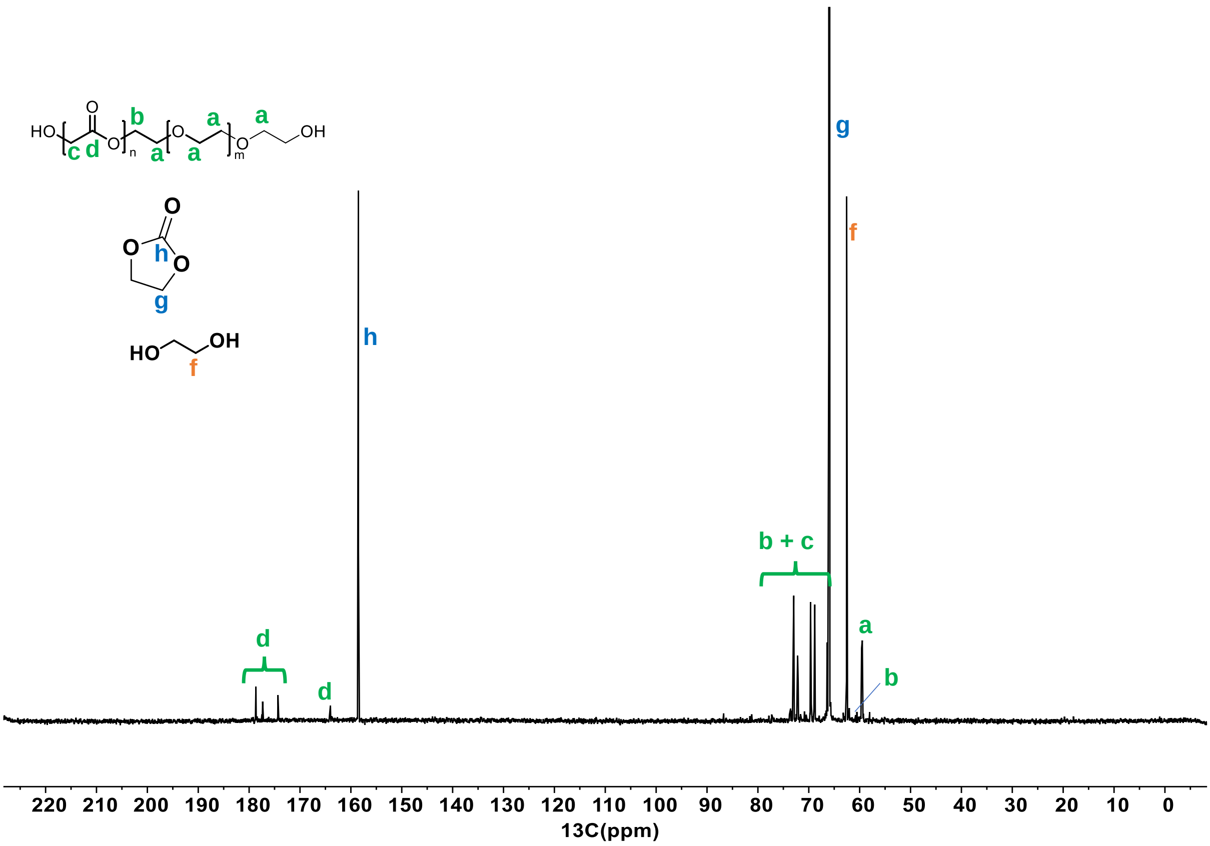

Figure S40. $^{13}C\{^{1}H\}$ NMR (126 MHz, D_2O) spectrum of the reaction mixture resulting from the reaction corresponding to Table S1; Entry 10. Ethylene Carbonate is used as an internal standard.

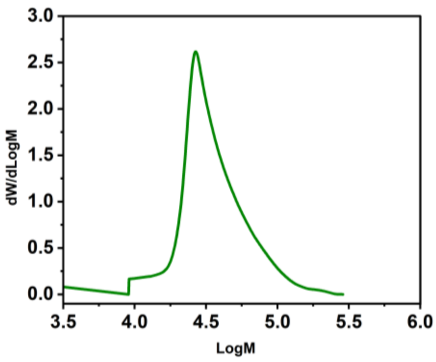

Figure S41. GPC data of the reaction mixture resulting from the reaction corresponding to Table S1, Entry 10.

<u>Table S1; Entry 11:</u>

^{1}H NMR (500 MHz, D_2O): δH 4.4-4.0 (br,O-**CH$_2$**-CO-,CO-O-**CH$_2$**-CH$_2$), 3.6 (br, CH$_2$-**CH$_2$**-O-).

$^{13}C\{^{1}H\}$ NMR (126 MHz, D_2O): δC 176.5, 174.9, 174.2 (-CH$_2$-**CO**-O), 73.2, 69.4, 68.8, 64.0, 61.2, 60.2 (-O-**CH$_2$**-CO-, CO-O-**CH$_2$**-CH$_2$), 61.8,59.6 (-CH$_2$-**CH$_2$**-O-).

GPC: Mn =24,320 gmol^{-1} (Đ =1.5).

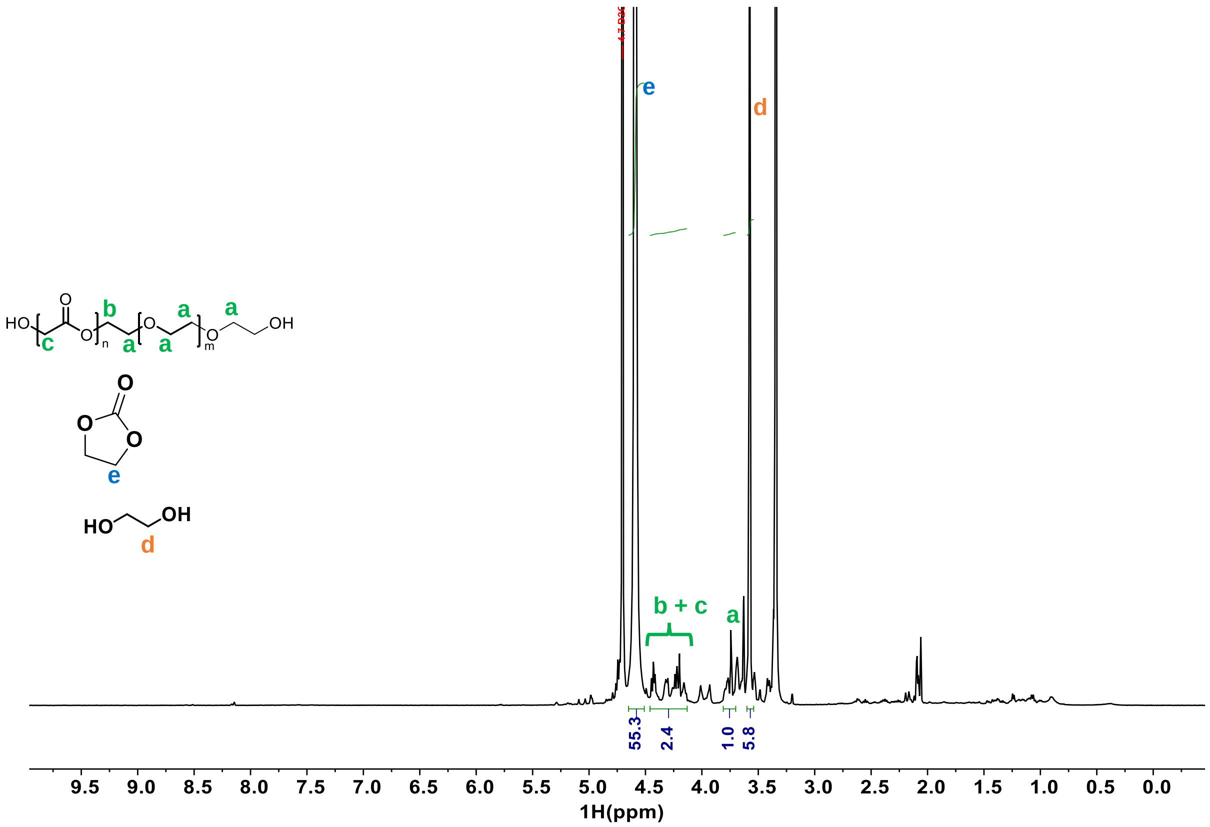

Figure S42. ^{1}H NMR (500 MHz, D_2O) spectrum of the reaction mixture resulting from the reaction corresponding to Table S1; Entry 11. Ethylene Carbonate (0.7 mmol) is used as an internal standard.

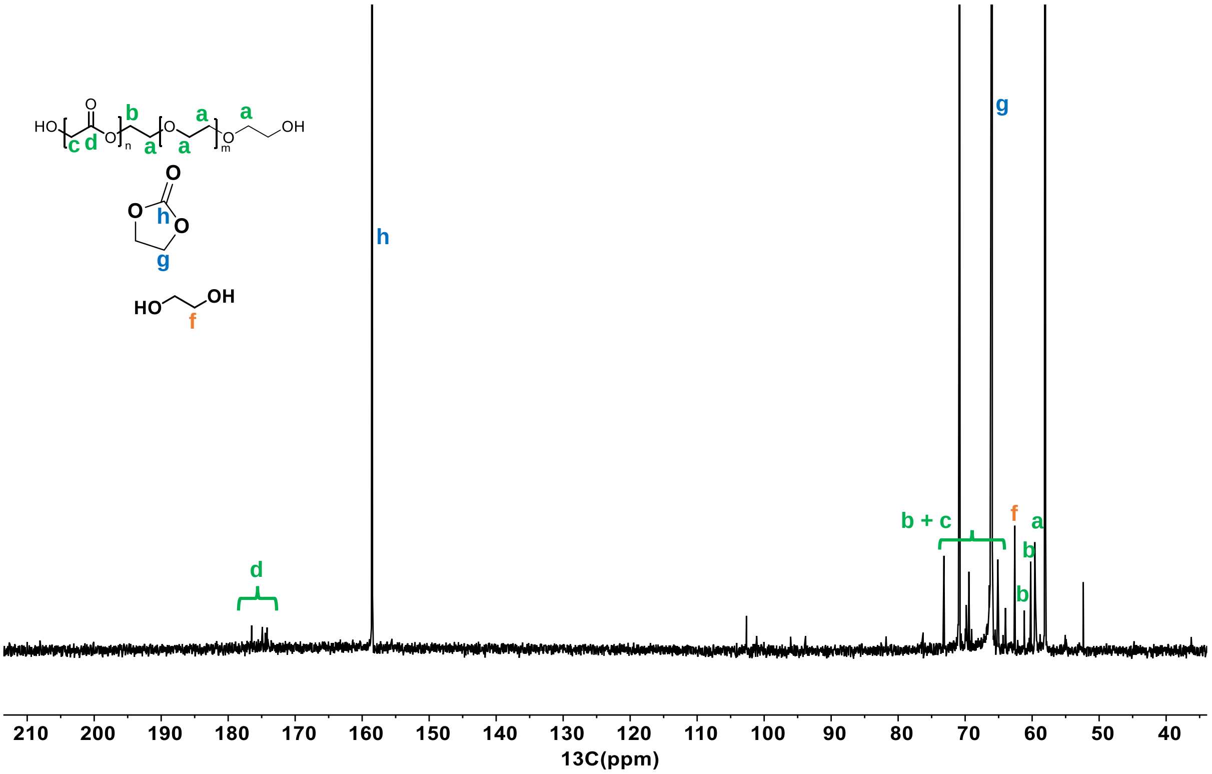

Figure S43. $^{13}C\{^{1}H\}$ NMR (126 MHz, D_2O) spectrum of the reaction mixture resulting from the reaction corresponding to Table S1; Entry 11. Ethylene Carbonate is used as an internal standard.

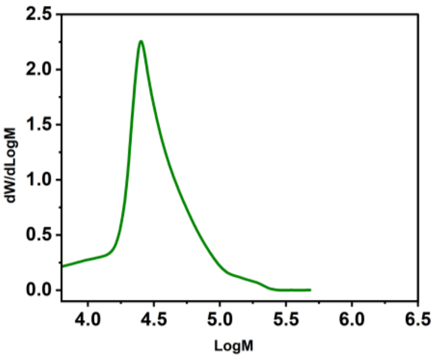

Figure S44. GPC data of the reaction mixture resulting from the reaction corresponding to Table S1; Entry 11.

Table S1; Entry 12:

^{1}H NMR (500 MHz, D_2O): δH 4.4-4.0 (br,O-**CH_2**-CO-,CO-O-**CH_2**-CH_2), 3.6 (br, CH_2-**CH_2**-O-).

^{13}C{^{1}H} NMR (126 MHz, D_2O): δC 174.3, 172.3, 164.0 (-CH_2-**CO**-O), 73.2, 72.1, 69.4, 67.8, 64.0, 61.1, 60.4 (-O-**CH_2**-CO-, CO-O-**CH_2**-CH_2), 61.8, 60.8, 59.4 (-CH_2-**CH_2**-O-).

GPC: Mn =32,680 $gmol^{-1}$ (Đ =1.4).

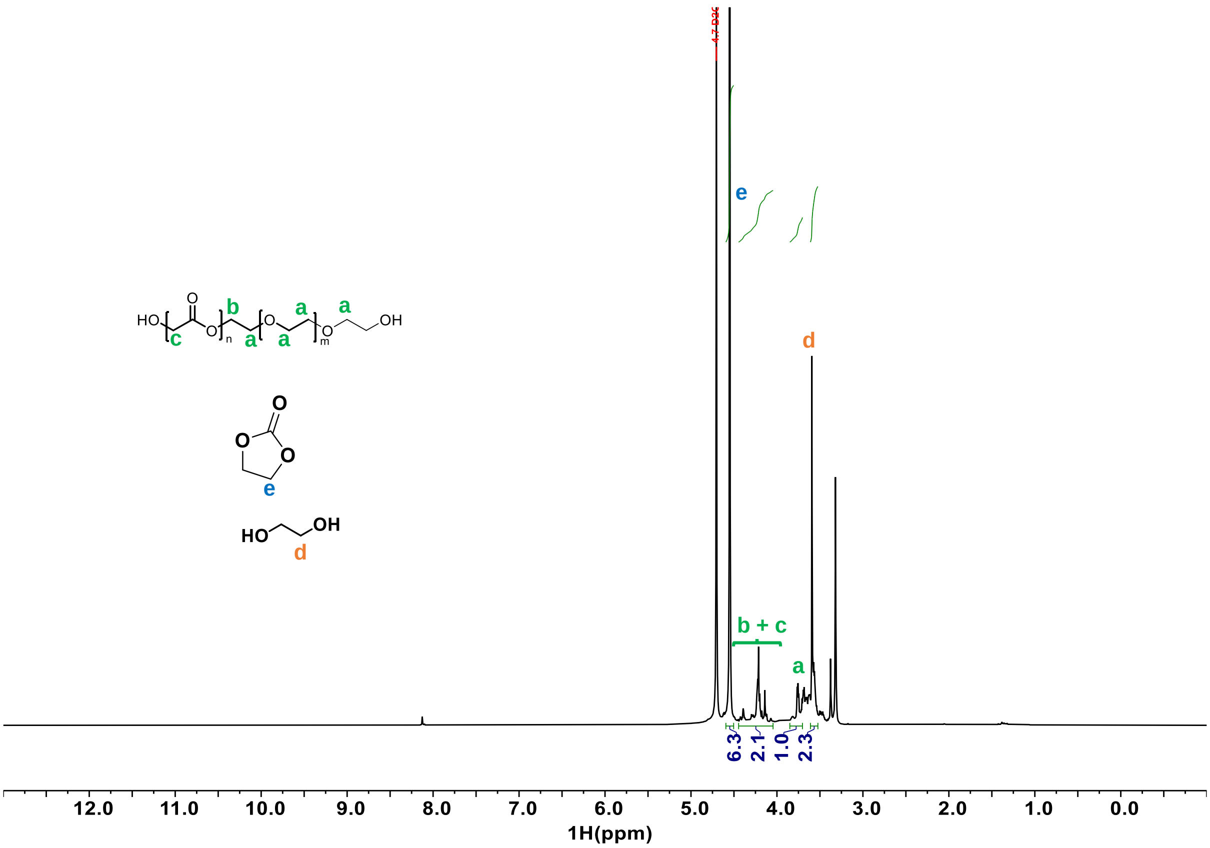

Figure S45. ^{1}H NMR (500 MHz, D_2O) spectrum of the reaction mixture resulting from the reaction corresponding to Table S1; Entry 12. Ethylene Carbonate (1 mmol) is used as an internal standard.

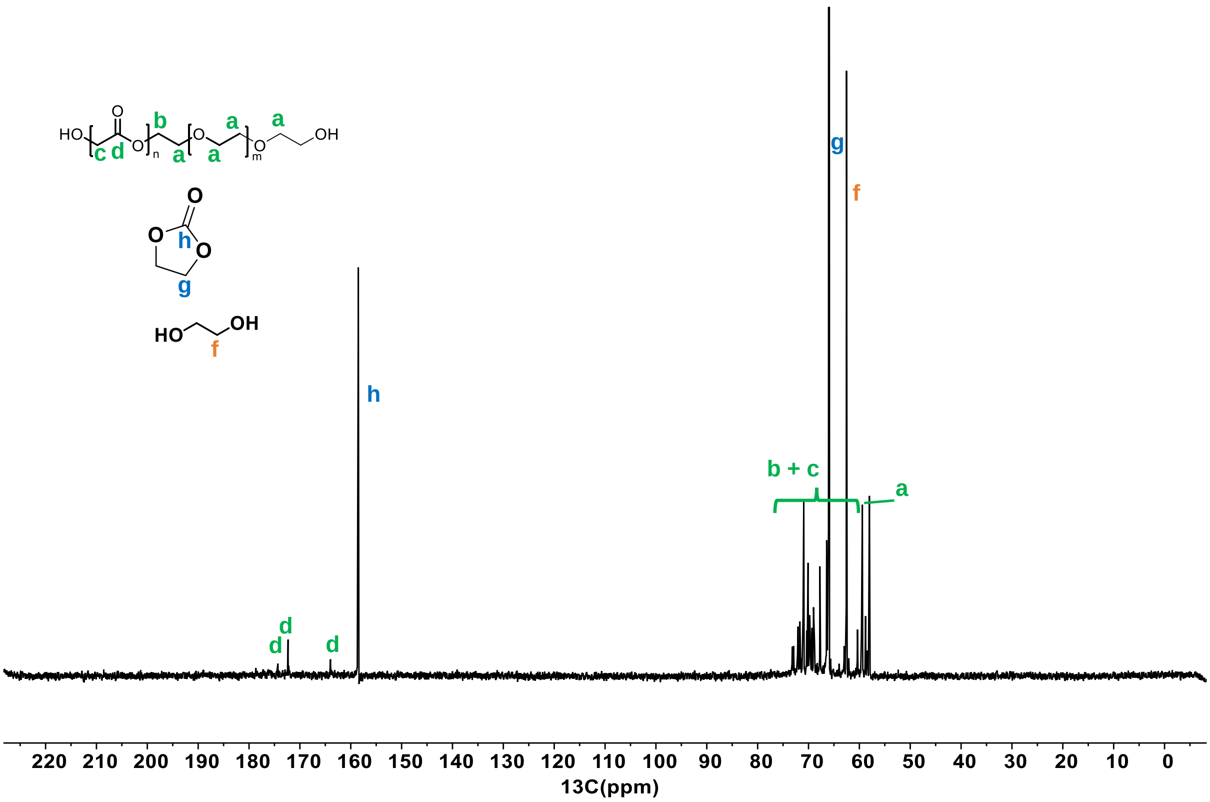

Figure S46. $^{13}C\{^{1}H\}$ NMR (126 MHz, D_2O) spectrum of reaction mixture resulting from the reaction corresponding to Table S1; Entry 12. Ethylene Carbonate is used as an internal standard.

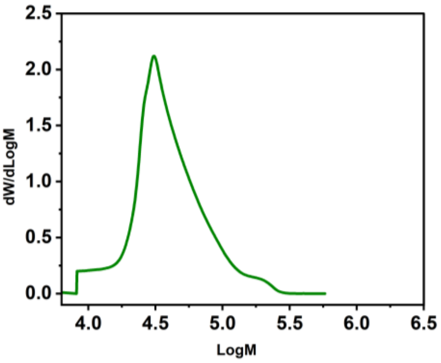

Figure S47. GPC data of the reaction mixture resulting from the reaction corresponding to Table S1, Entry 12.

Table S1; Entry 13:

^{1}H NMR (500 MHz, D_2O): δH 4.4-4.0 (br,O-**CH$_2$**-CO-,CO-O-**CH$_2$**-CH$_2$), 3.7 (br, CH$_2$-**CH$_2$**-O-).

^{13}C{^{1}H} NMR (126 MHz, D_2O): δC 178.7, 177.3, 174.3(-CH$_2$-**CO**-O), 72.9, 72.2, 69.6, 68.8, 66.8, 61.2 (-O-**CH$_2$**-CO-, CO-O-**CH$_2$**-CH$_2$), 61.8, 60.3, 59.5 (-CH$_2$-**CH$_2$**-O-).

GPC: Mn =48,940 gmol^{-1} (Đ =1.4).

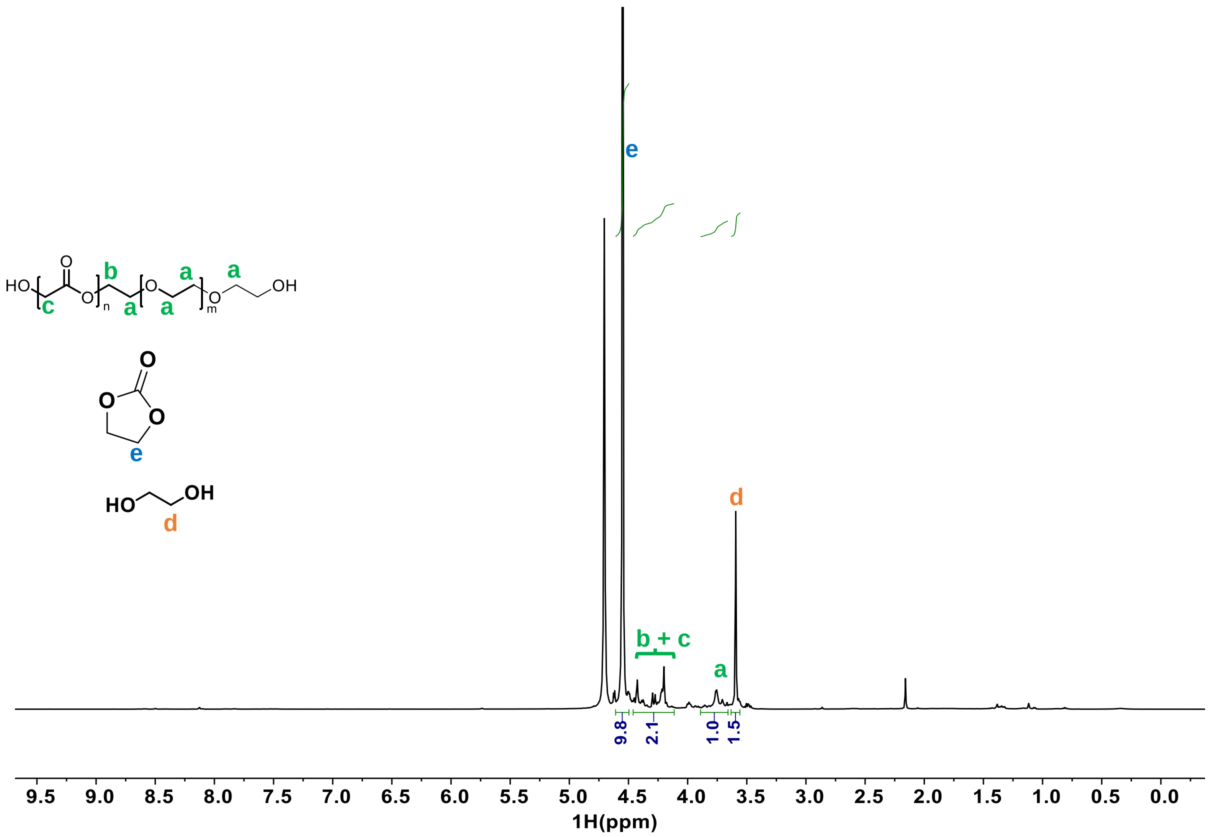

Figure S48. ^{1}H NMR (500 MHz, D_2O) spectrum of the reaction mixture resulting from the reaction corresponding to Table S1; Entry 13. Ethylene Carbonate (0.7 mmol) is used as an internal standard.

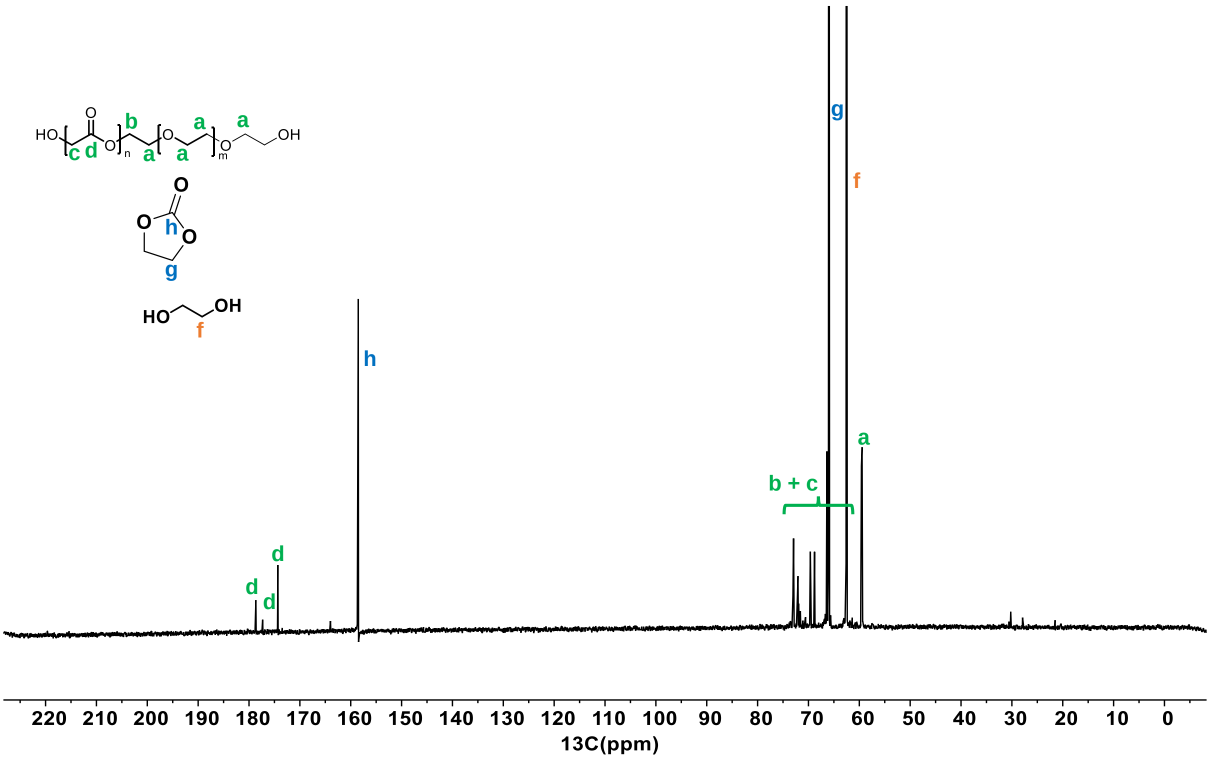

Figure S49. $^{13}C\{^{1}H\}$ NMR (126 MHz, D_2O) spectrum of the reaction mixture resulting from the reaction corresponding to Table S1; Entry 13. Ethylene Carbonate is used as an internal standard.

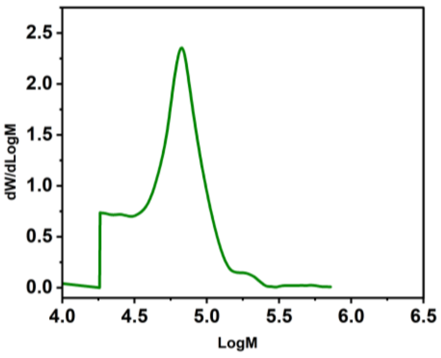

Figure S50. GPC data of the reaction mixture resulting from the reaction corresponding to Table S1; Entry 13.

<u>Table S1; Entry 14:</u>

^{1}H NMR (500 MHz, D_2O): δH 4.4-4.0 (br,O-**CH_2**-CO-,CO-O-**CH_2**-CH_2), 3.6 (br, CH_2-**CH_2**-O-).

^{13}C{^{1}H} NMR (126 MHz, D_2O): δC 179.4, 174.3 (-CH_2-**CO**-O), 72.9, 72.1, 71.9, 71.6, 69.6, 66.4, 66.8, 61.4 (-O-**CH_2**-CO-, CO-O-**CH_2**-CH_2), 61.6, 61.0, 59.5 (-CH_2-**CH_2**-O-).

GPC: Mn =24,010 $gmol^{-1}$ (Đ =1.6).

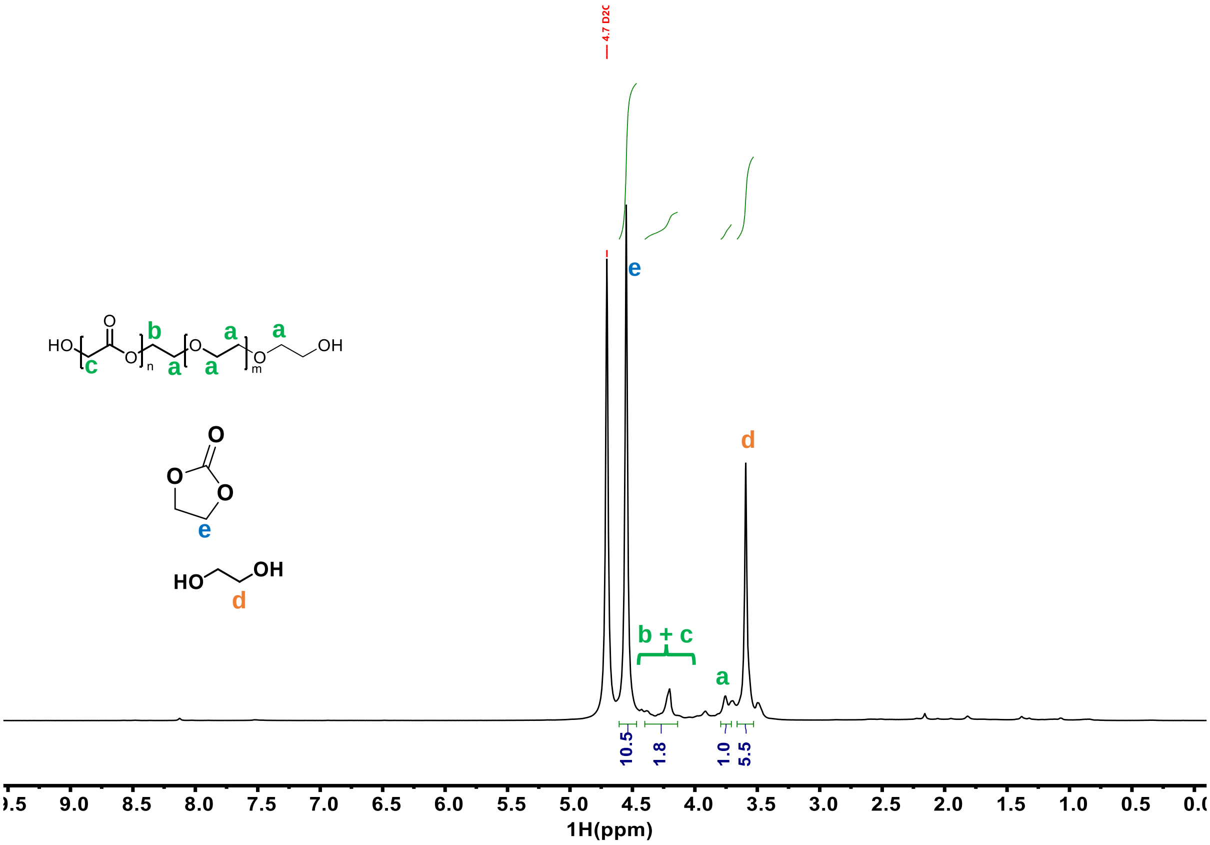

Figure S51. ^{1}H NMR (500 MHz, D_2O) spectrum of the reaction mixture resulting from the reaction corresponding to Table S1; Entry 14. Ethylene Carbonate (0.6 mmol) is used as an internal standard.

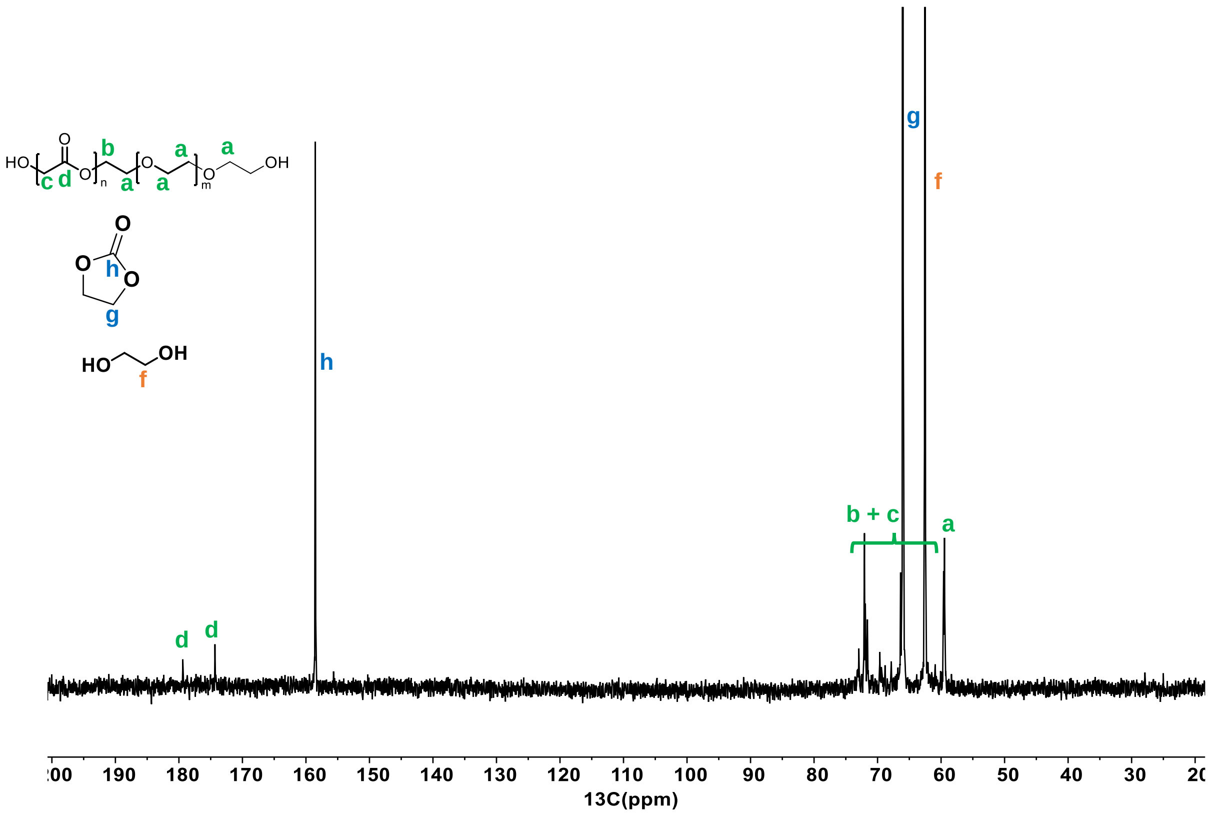

Figure S52. $^{13}C\{^{1}H\}$ NMR (126 MHz, D_2O) spectrum of the reaction mixture resulting from the reaction corresponding to Table S1; Entry 14. Ethylene Carbonate is used as an internal standard.

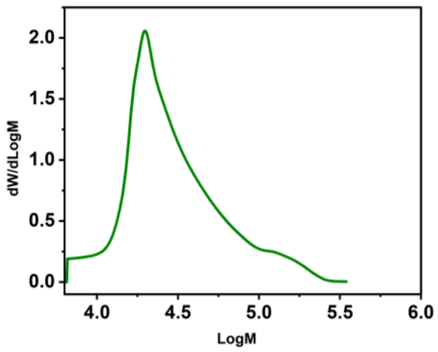

Figure S53. GPC data of the reaction mixture resulting from the reaction corresponding to Table S1; Entry 14.

Table S1; Entry 15:

No reaction

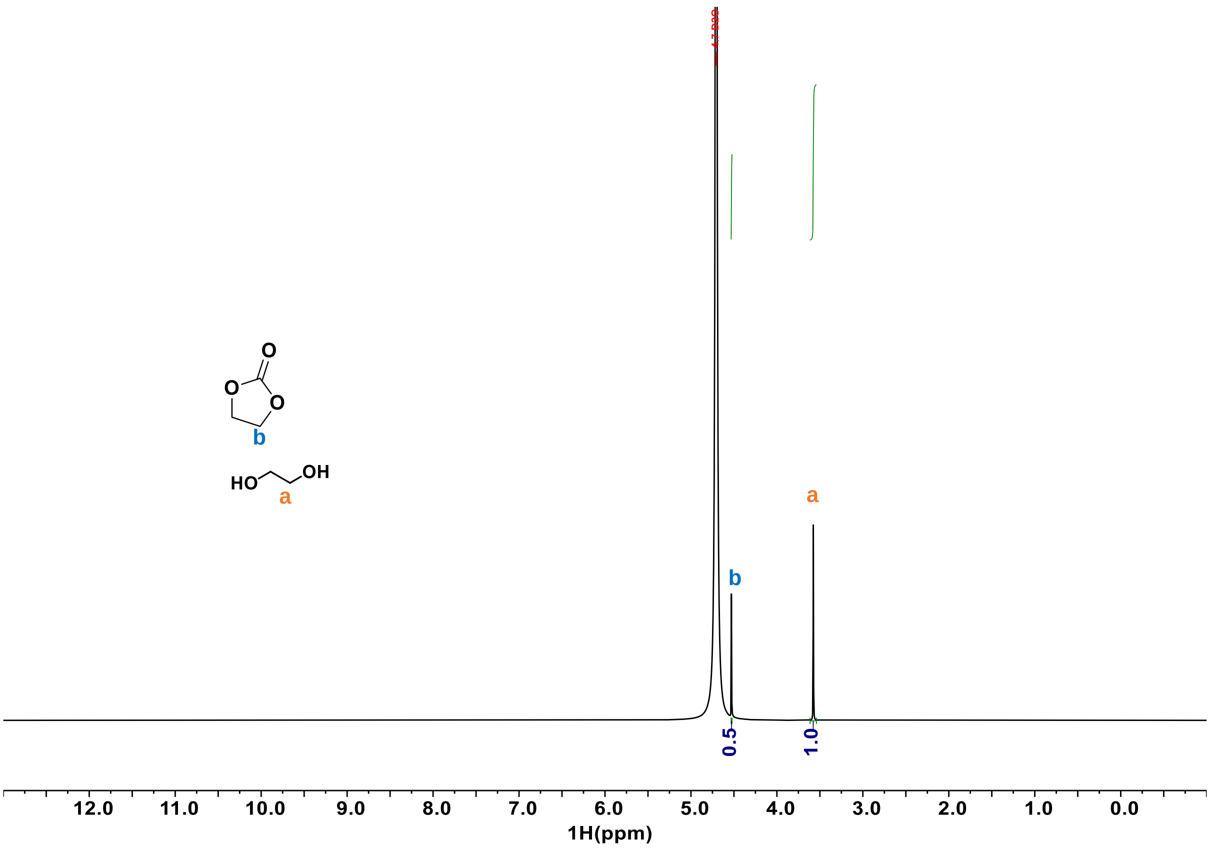

Figure S54. ^{1}H NMR (500 MHz, D_2O) spectrum of the reaction mixture resulting from the reaction corresponding to Table S1; Entry 15. Ethylene Carbonate (1mmol) is used as an internal standard.

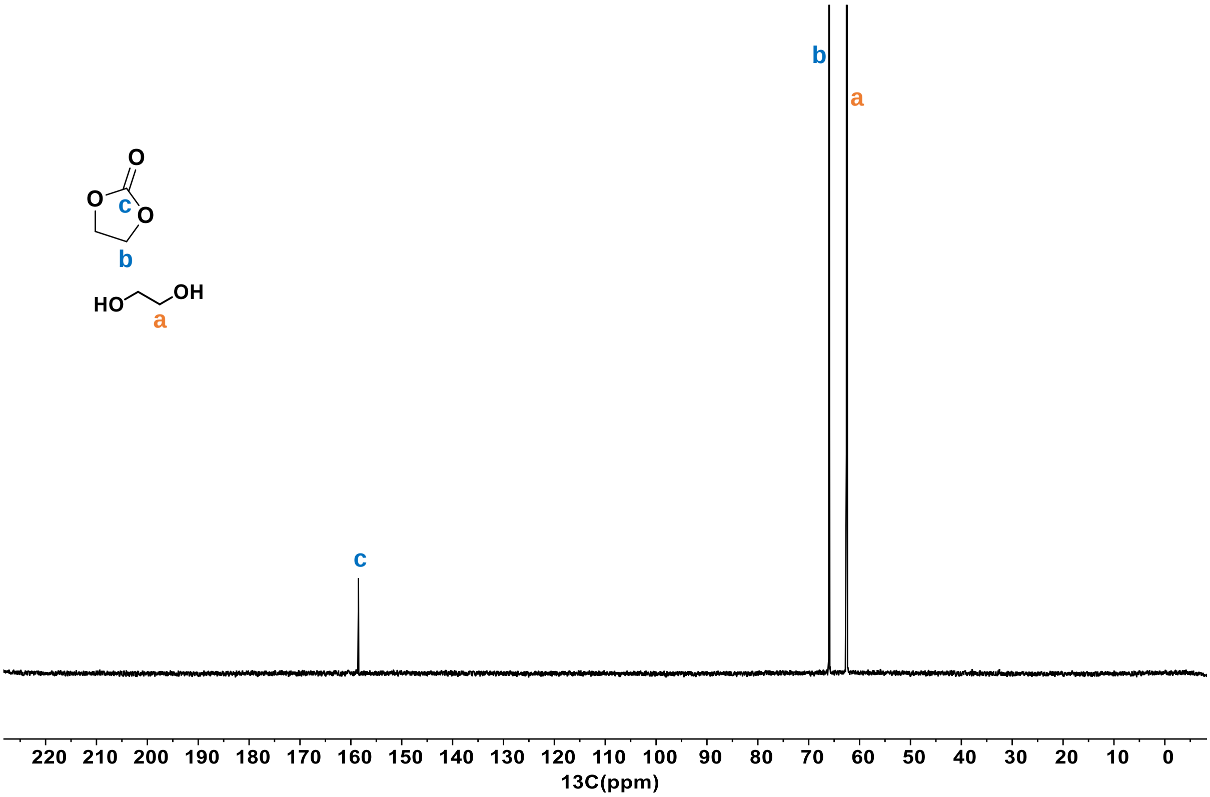

Figure S55. $^{13}C\{^{1}H\}$ NMR (126 MHz, D_2O) spectrum of the reaction mixture resulting from the reaction corresponding to Table S1; Entry 15. Ethylene Carbonate is used as an internal standard.

<u>Table S1; Entry 16:</u>

^{1}H NMR (500 MHz, D_2O): δH 4.4-4.0 (br,O-**CH_2**-CO-,CO-O-**CH_2**-CH_2), 3.6 (br, CH_2-**CH_2**-O-).

^{13}C{^{1}H} NMR (126 MHz, D_2O): δC 178.3, 177.3, 174.3 (-CH_2-**CO**-O), 73.1, 73.0, 72.1, 69.8, 68.8, 66.4 (-O-**CH_2**-CO-, CO-O-**CH_2**-CH_2), 61.7, 60.3, 59.5 (-CH_2-**CH_2**-O-).

GPC: Mn =29,130 gmol^{-1} (Đ =1.5).

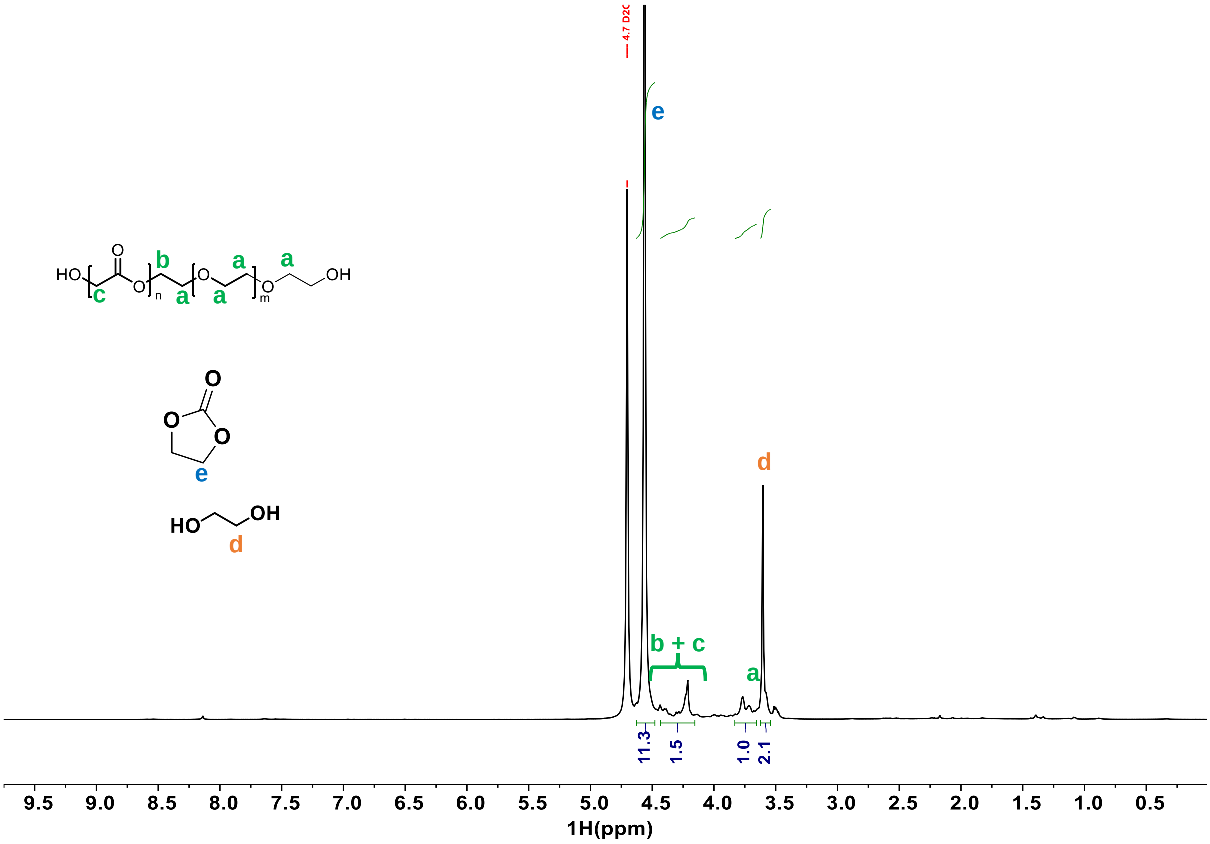

Figure S56. ^{1}H NMR (500 MHz, D_2O) spectrum of the reaction mixture resulting from the reaction corresponding to Table S1; Entry 16. Ethylene Carbonate (1 mmol) is used as an internal standard.

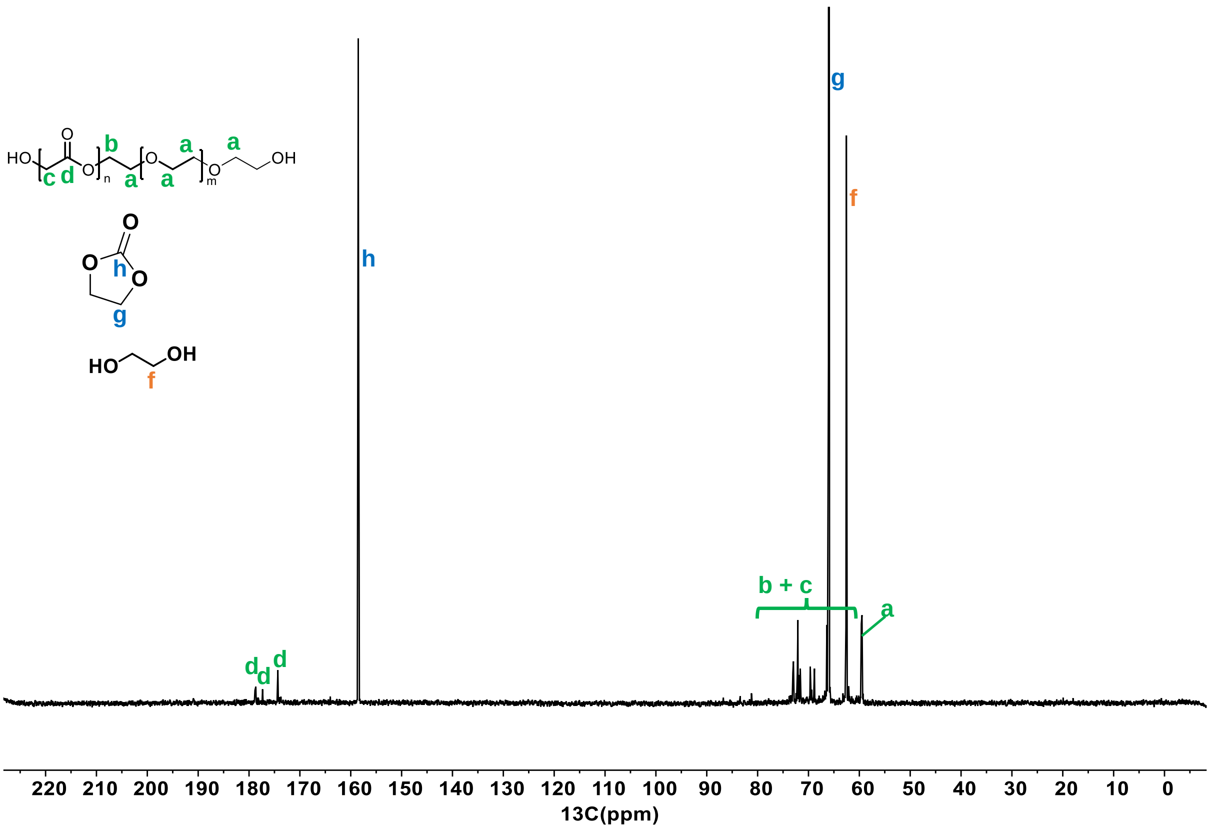

Figure S57. $^{13}C\{^1H\}$ NMR (126 MHz, D_2O) spectrum of the reaction mixture resulting from the reaction corresponding to Table S1; Entry 16. Ethylene Carbonate is used as an internal standard.

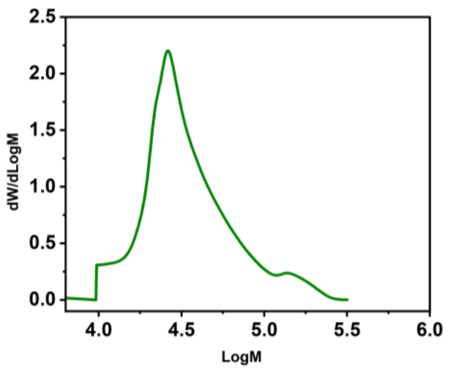

Figure S58. GPC data of the reaction mixture resulting from the reaction corresponding to Table S1; Entry 16. Ethylene Carbonate is used as an internal standard.

<u>Table S1; Entry 17:</u>

^{1}H NMR (500 MHz, D_2O): δH 8.3 (O-H), 4.4-4.0 (br,O-**CH_2**-CO-,CO-O-**CH_2**-CH_2), 3.6 (br, CH_2-**CH_2**-O-).

$^{13}C\{^1H\}$ NMR (126 MHz, D_2O): δC 179.5, 174.3 (-CH_2-**CO**-O), 72.1, 71.9, 71.6, 69.9 66.4(-O-**CH_2**-CO-, CO-O-**CH_2**-CH_2), 61.8, 59.5 (-CH_2-**CH_2**-O-).

GPC: Mn =33,790 $gmol^{-1}$ (Đ =1.4)

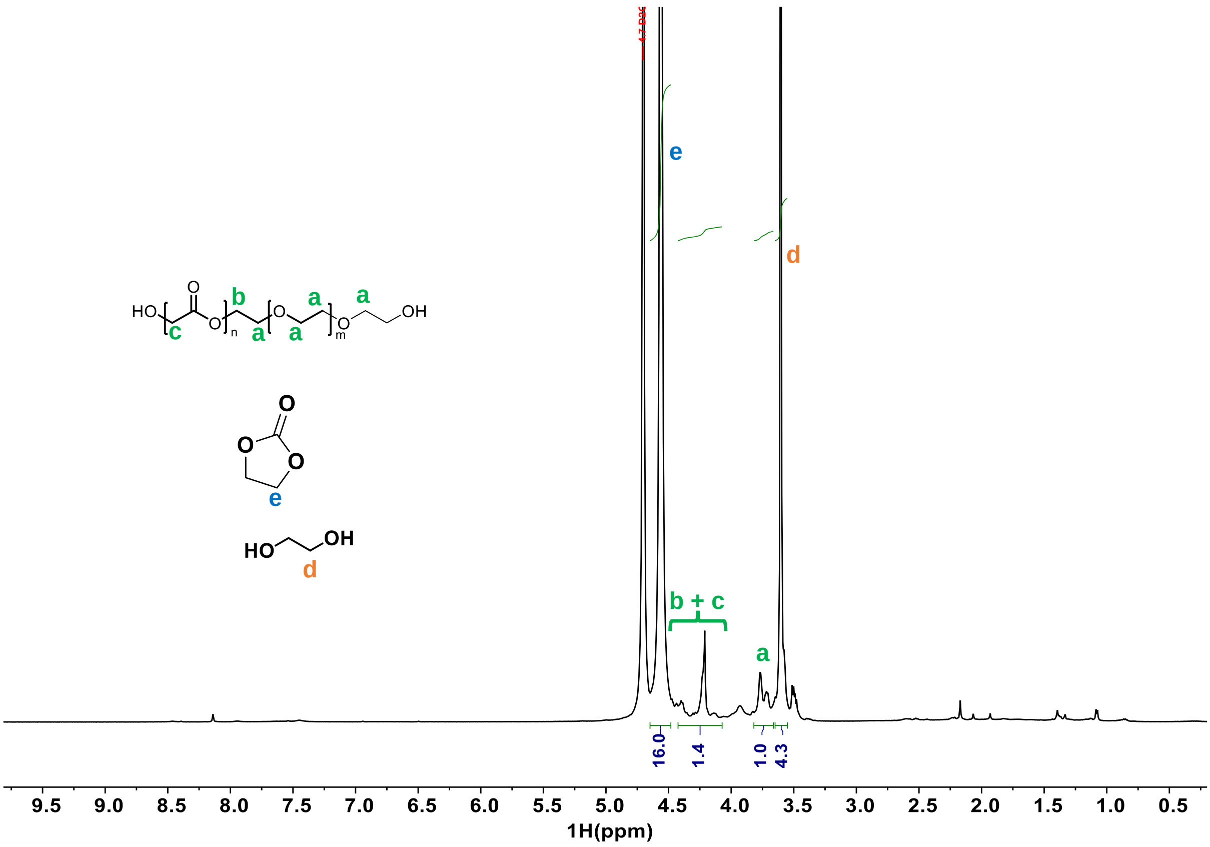

Figure S59. ^{1}H NMR (500 MHz, D_2O) spectrum of the reaction mixture resulting from the reaction corresponding to Table S1; Entry 17. Ethylene Carbonate (1 mmol) is used as an internal standard.

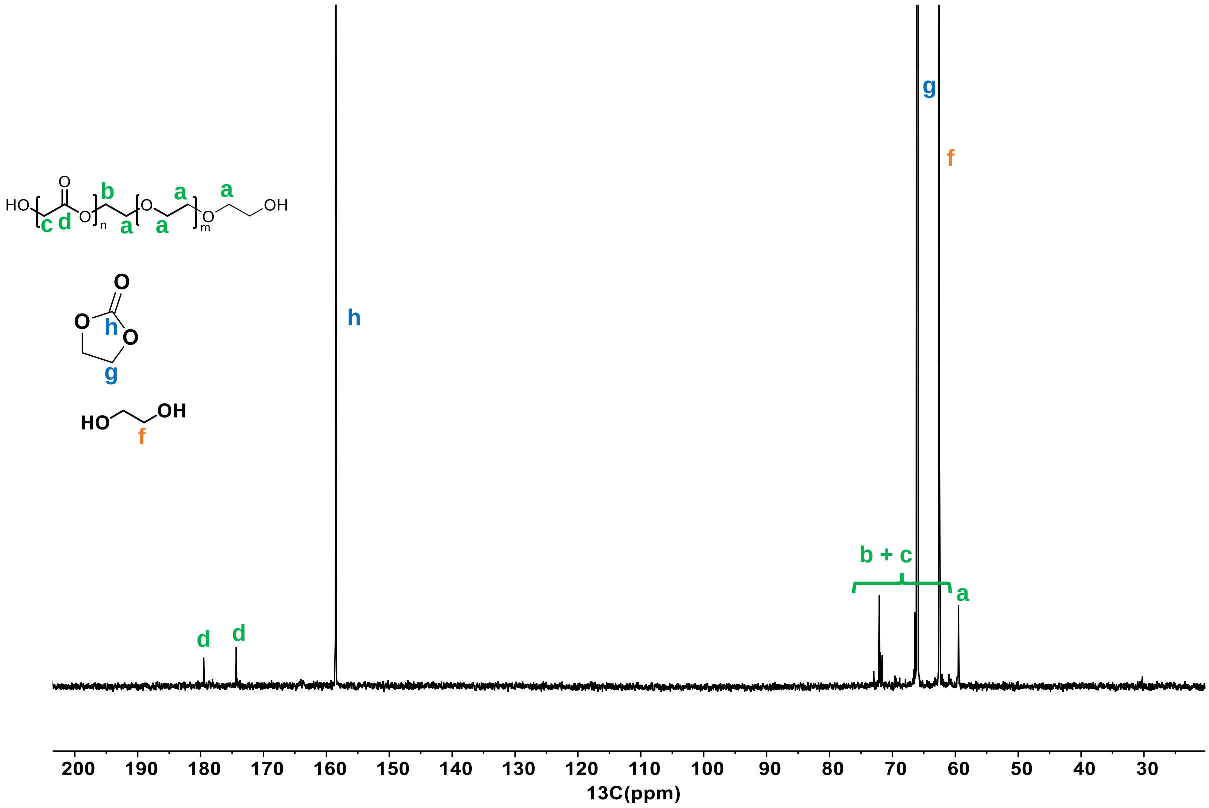

Figure S60. $^{13}C\{^{1}H\}$ NMR (126 MHz, D_2O) spectrum of the reaction mixture resulting from the reaction corresponding to Table S1; Entry 17. Ethylene Carbonate is used as an internal standard.

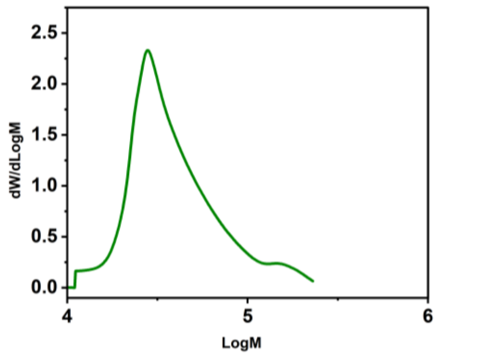

Figure S61. GPC data of the reaction mixture resulting from the reaction corresponding to Table S1; Entry 17.

Table S1; Entry 18:

^{1}H NMR (500 MHz, D_2O): δH 4.4-4.0 (br,O-**CH_2**-CO-,CO-O-**CH_2**-CH_2), 3.6 (br, CH_2-**CH_2**-O-).

^{13}C{^{1}H} NMR (126 MHz, D_2O): δC 178.7, 177.3, 174.5, (-CH_2-**CO**-O), 72.7, 72.1, 69.6, 68.6, 66.4 (-O-**CH_2**-CO-, CO-O-**CH_2**-CH_2), 61.9, 59.5 (-CH_2-**CH_2**-O-).

GPC: Mn =17,370 $gmol^{-1}$ (Đ =1.6).

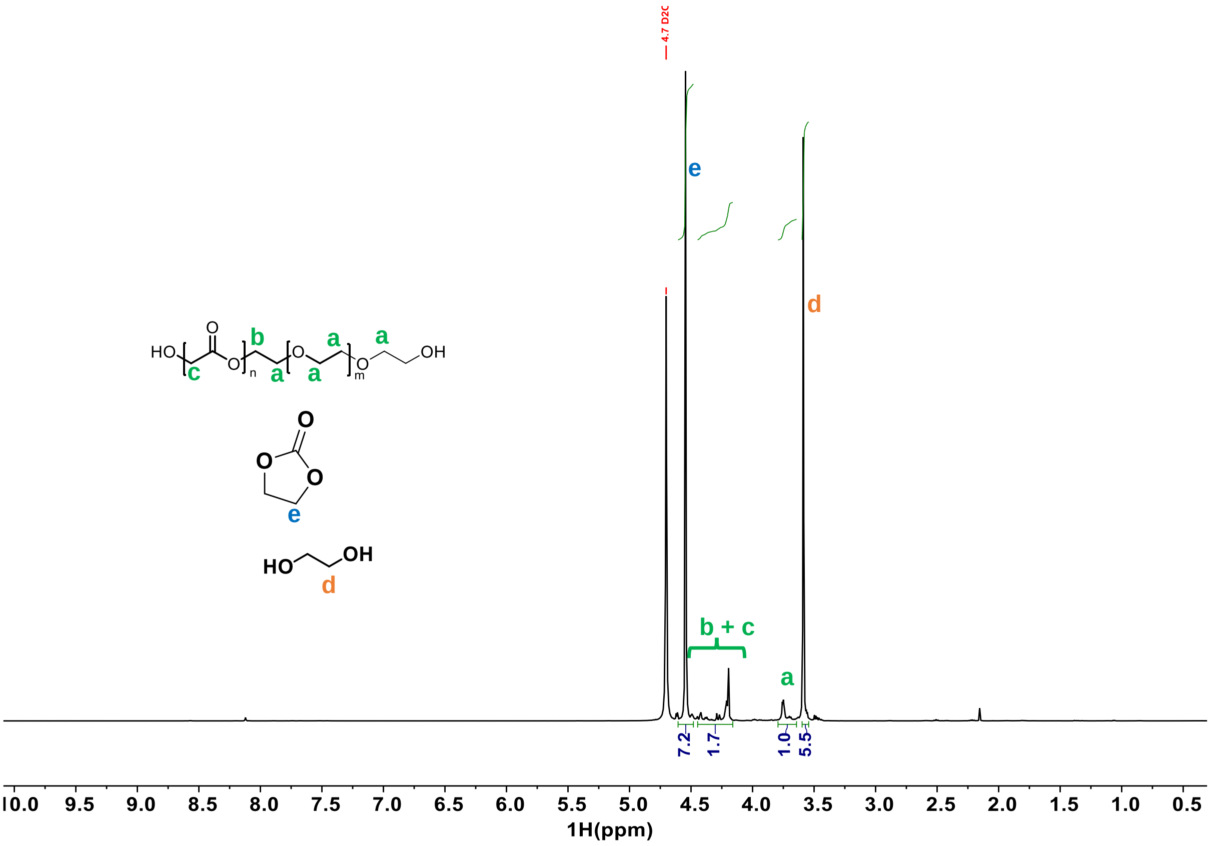

Figure S62. ^{1}H NMR (500 MHz, D_2O) spectrum of the reaction mixture resulting from the reaction corresponding to Table S1; Entry 18. Ethylene Carbonate (1.1 mmol) is used as an internal standard.

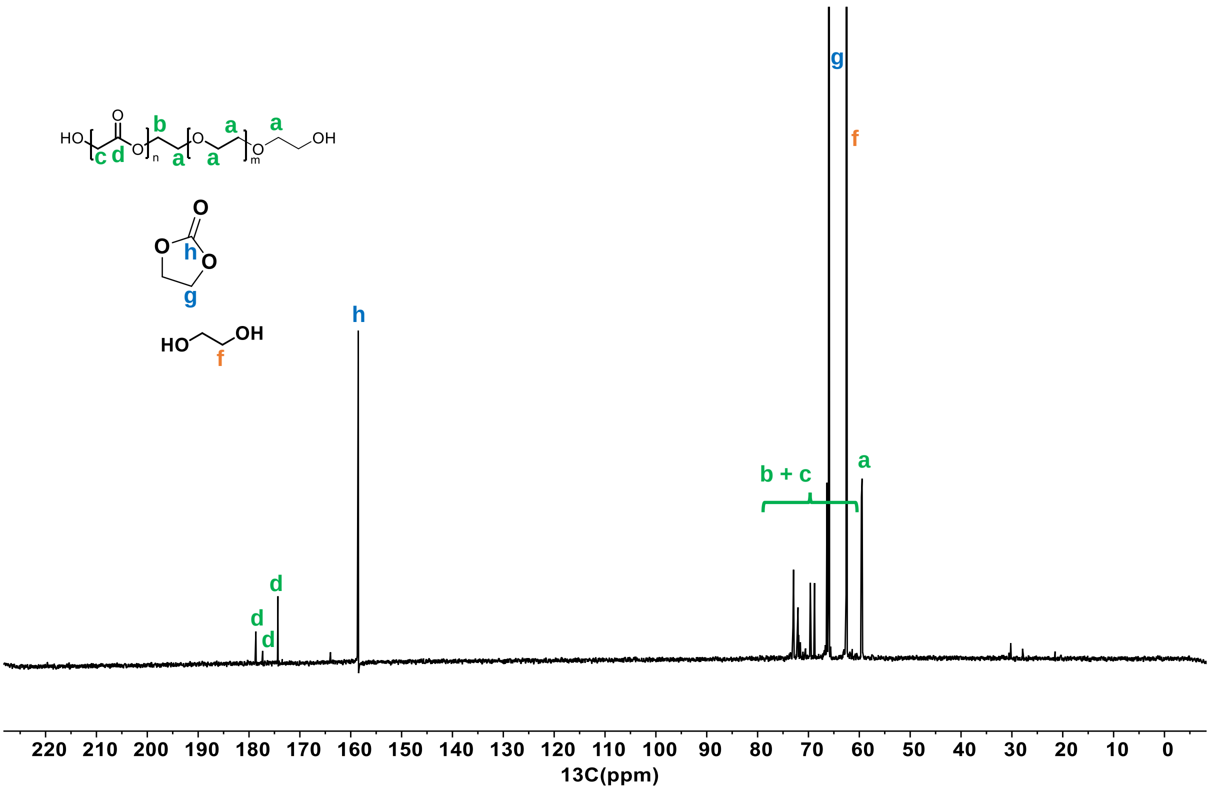

Figure S63. $^{13}C\{^1H\}$ NMR (126 MHz, D_2O) spectrum of the reaction mixture resulting from the reaction corresponding to Table S1; Entry 18. Ethylene Carbonate is used as an internal standard.

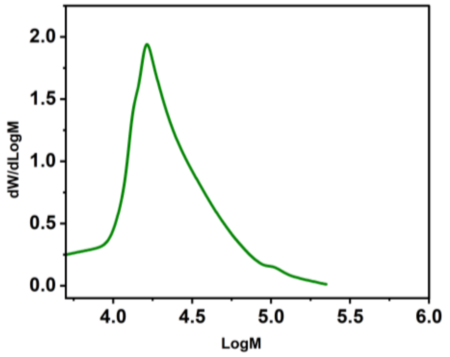

Figure S64. GPC data of the reaction mixture resulting from the reaction corresponding to Table S1; Entry 18.

Table S1; Entry 19:

^{1}H NMR (500 MHz, D_2O): δH 4.4-4.0 (br,O-**CH_2**-CO-,CO-O-**CH_2**-CH_2), 3.6 (br, CH_2-**CH_2**-O-).

$^{13}C\{^1H\}$ NMR (126 MHz, D_2O): δC 178.6, 174.3, (-CH_2-**CO**-O), 72.9, 72.2, 69.6, 68.8, 66.4 (-O-**CH_2**-CO-, CO-O-**CH_2**-CH_2), 60.8, 59.4 (-CH_2-**CH_2**-O-).

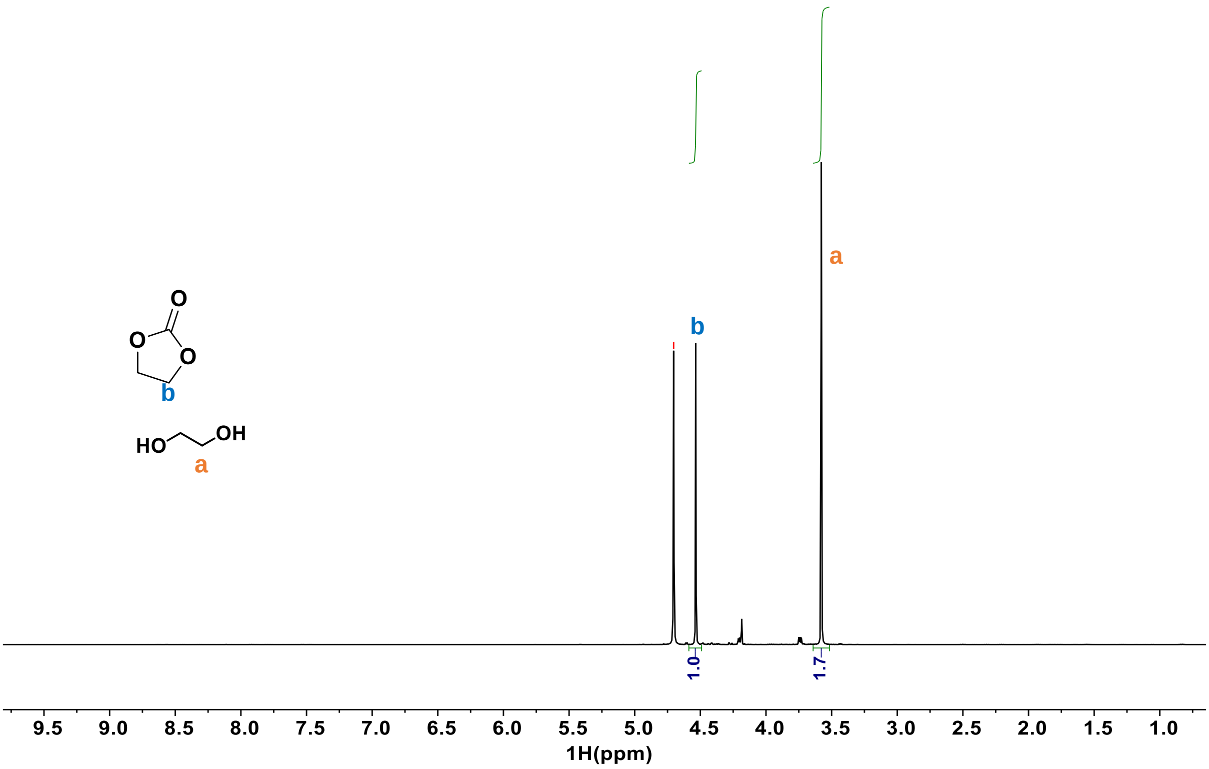

Figure S65. ^{1}H NMR (500 MHz, D_2O) spectrum of the reaction mixture resulting from the reaction corresponding to Table S1; Entry 19. Ethylene Carbonate (1.1 mmol) is used as an internal standard.

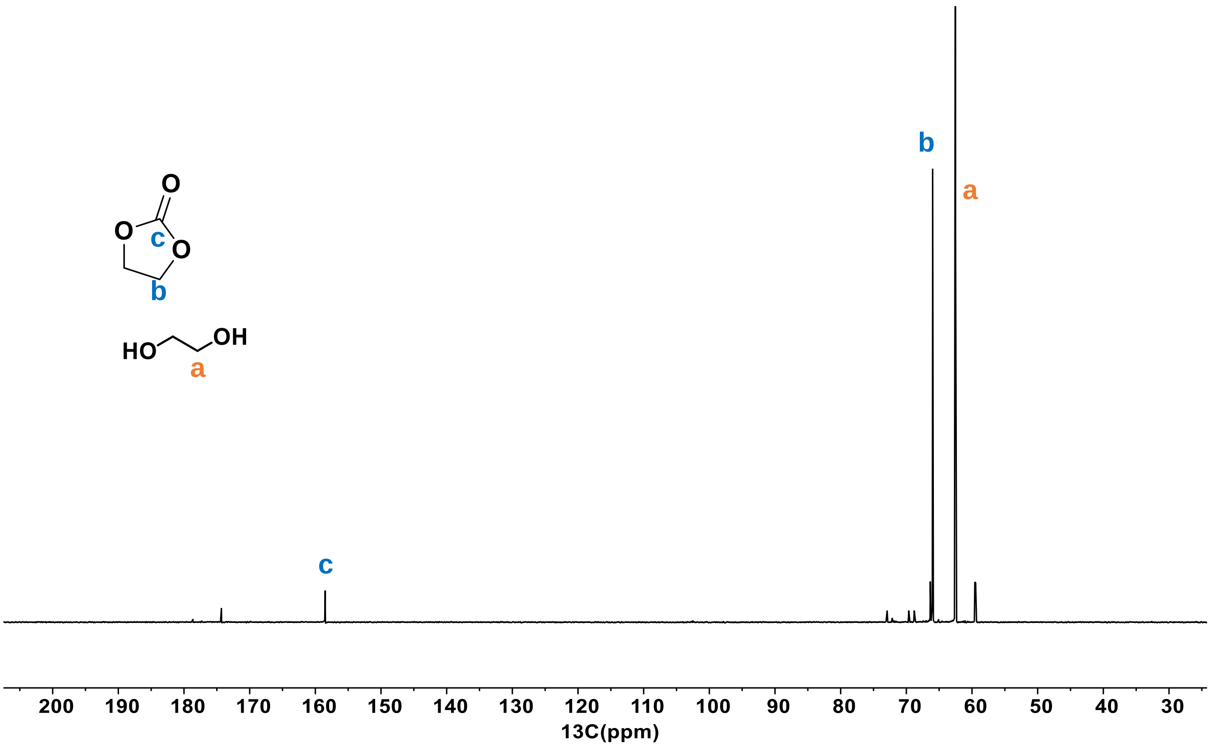

Figure S66. $^{13}C\{^{1}H\}$ NMR (126 MHz, D_2O) spectrum of the reaction mixture resulting from the reaction corresponding to Table S1; Entry 19. Ethylene Carbonate is used as an internal standard.

Table S1; Entry 20:

^{1}H NMR (500 MHz, D_2O): δH 4.4-4.0 (br,O-**CH_2**-CO-,CO-O-**CH_2**-CH_2), 3.7 (br, CH_2-**CH_2**-O-).

^{13}C{^{1}H} NMR (126 MHz, D_2O): δC 178.4,177.3,174.5(-CH_2-**CO**-O), 76.8, 69.5,66.4,65.4 (-O-**CH_2**-CO-, CO-O-**CH_2**-CH_2), 61.4, 60.3, 59.3(-CH_2-**CH_2**-O-).

GPC: Mn =55,330 gmol^{-1} (Đ =1.2).

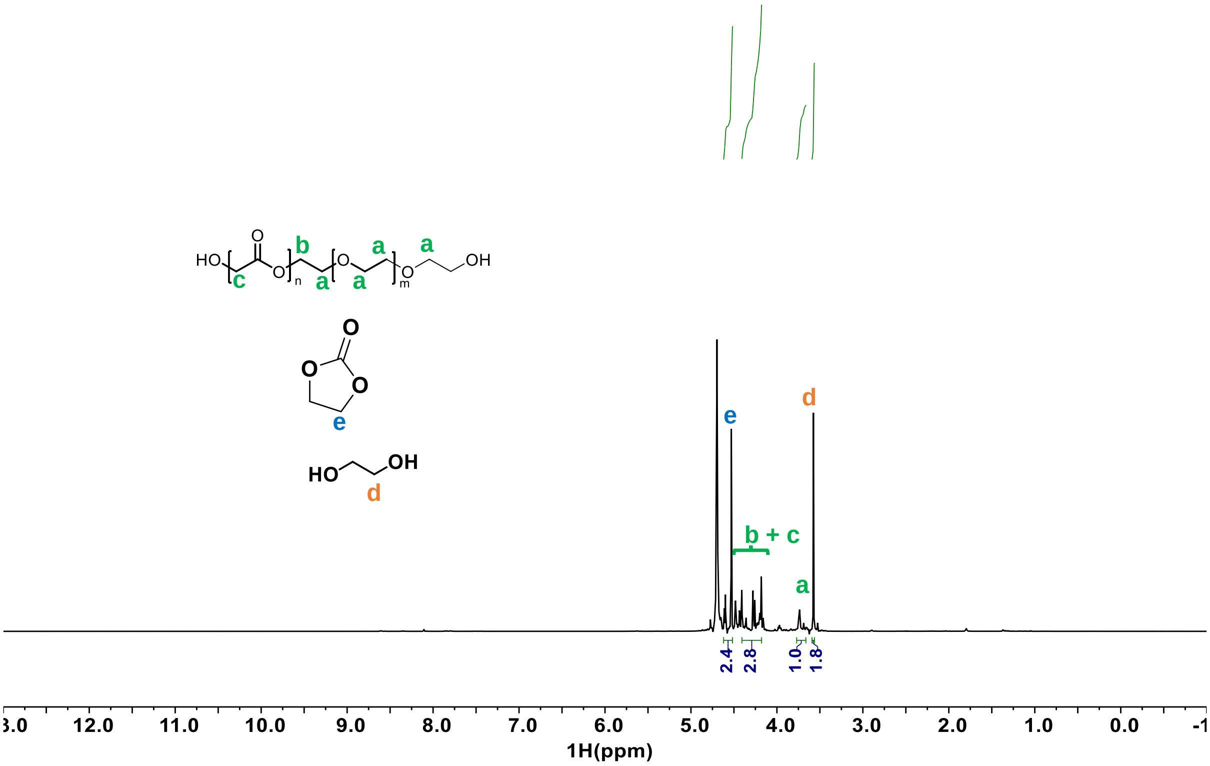

Figure S67. ^{1}H NMR (500 MHz, D_2O) spectrum of resulting mixture from reaction corresponding to Table S1; Entry 20. Ethylene carbonate (0.5 mmol) is used as an internal standard.

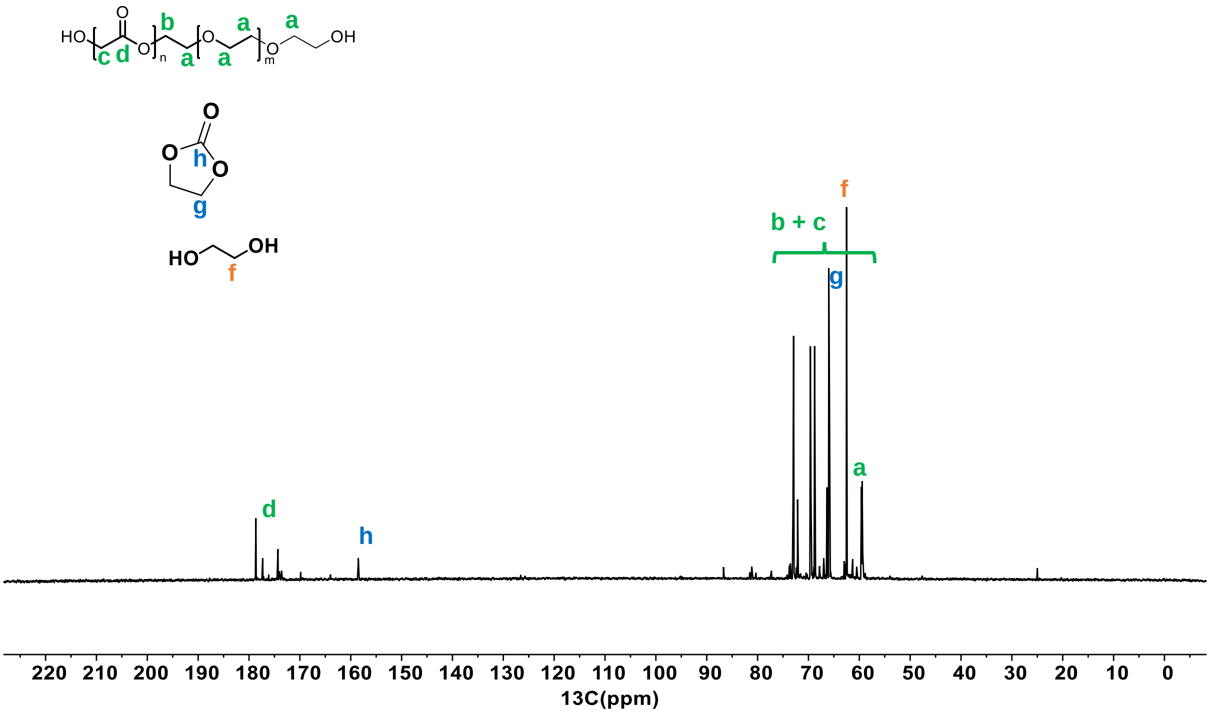

Figure S68. $^{13}C\{^{1}H\}$ NMR (126 MHz, D_2O) spectrum of the resulting mixture corresponding to Table S1; Entry 20. Ethylene Carbonate is used as an internal standard.

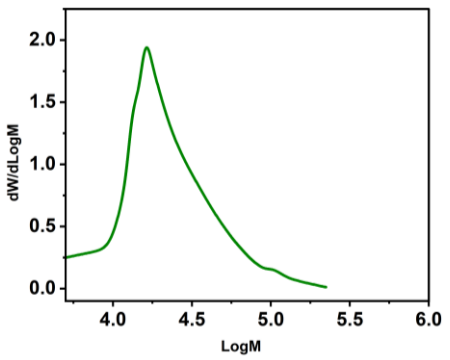

Figure S69. GPC data of the reaction mixture resulting from the reaction corresponding to Table S1; Entry 20.

<u>Table S1; Entry 21:</u>

No reaction

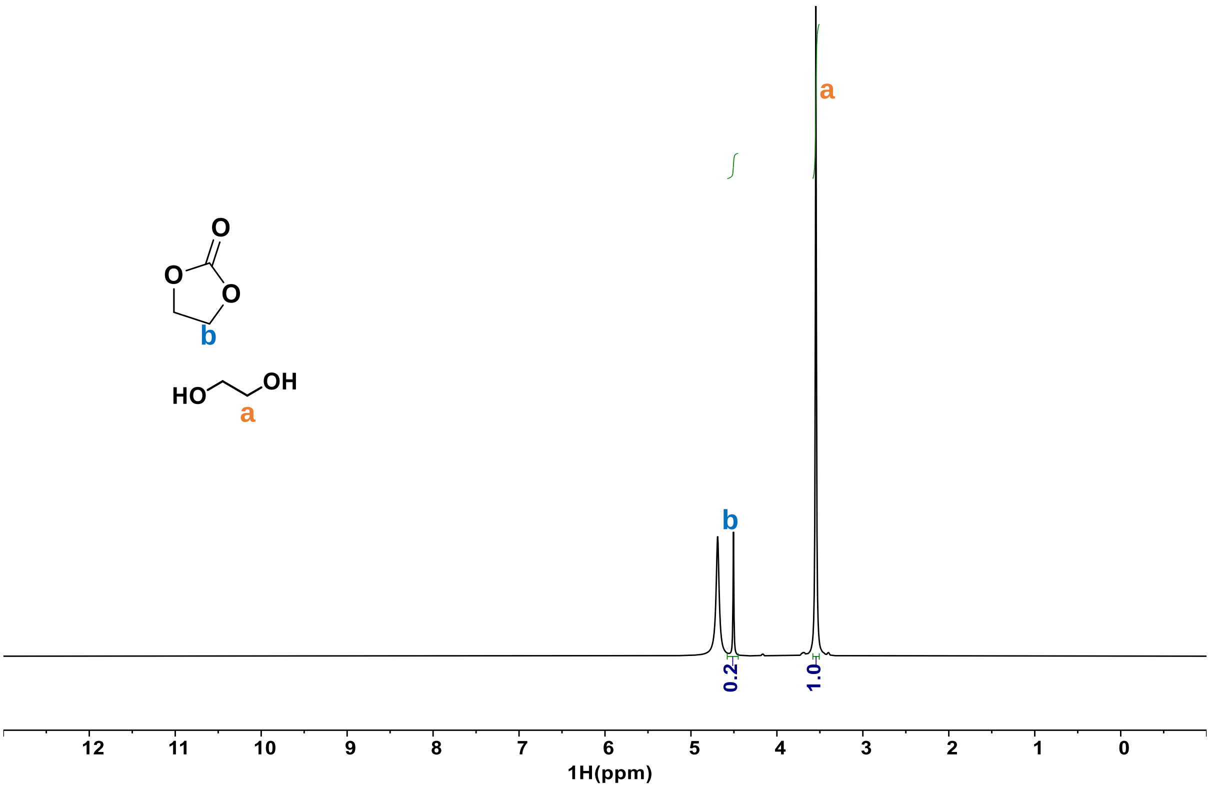

Figure S70. ^{1}H NMR (500 MHz, D_2O) spectrum of the reaction mixture resulting from the reaction corresponding to Table S1; Entry 21. Ethylene Carbonate (0.4 mmol) is used as an internal standard.

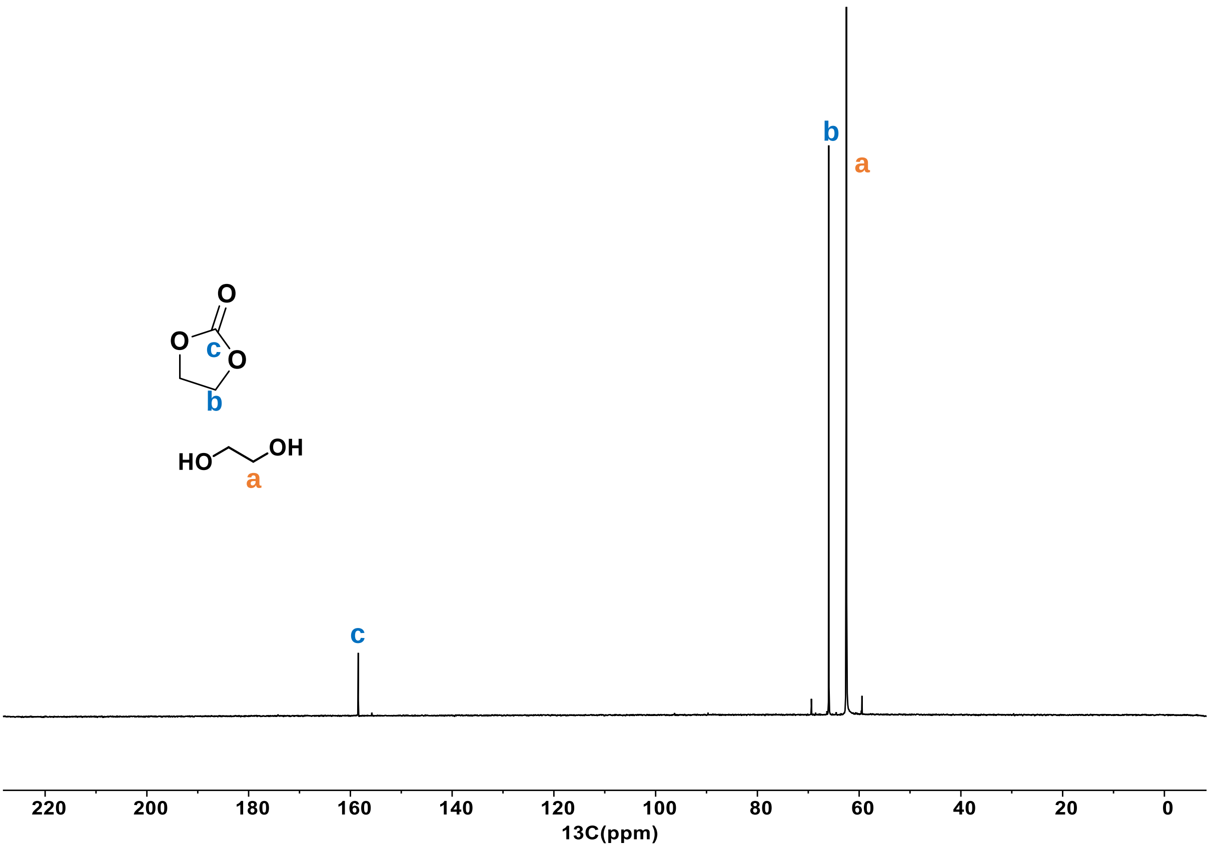

Figure S71. $^{13}C\{^1H\}$ NMR (126 MHz, D_2O) spectrum of the reaction mixture resulting from the reaction corresponding to Table S1; Entry 21. Ethylene Carbonate is used as an internal standard.

Table S1; Entry 22:

^{1}H NMR (500 MHz, D_2O): δH 4.4 (br,O-**CH**$_2$-CO-), 4.2-4.0 (br,CO-O-**CH**$_2$-CH$_2$), 3.8 (br, CH$_2$-**CH**$_2$-O-).

^{13}C{^{1}H} NMR (126 MHz, D_2O): δC 178.7, 177.3, 174.3, 163.8 (-CH$_2$-**CO**-O), 86.7, 81.6, 72.9, 72.2, 69.6, 68.8, 66.4, 59.7 (-O-**CH**$_2$-CO-, CO-O-**CH**$_2$-CH$_2$), 62.1, 60.3, 59.5 (-CH$_2$-**CH**$_2$-O-).

GPC: Mn =36,280 gmol^{-1} (Đ =1.3).

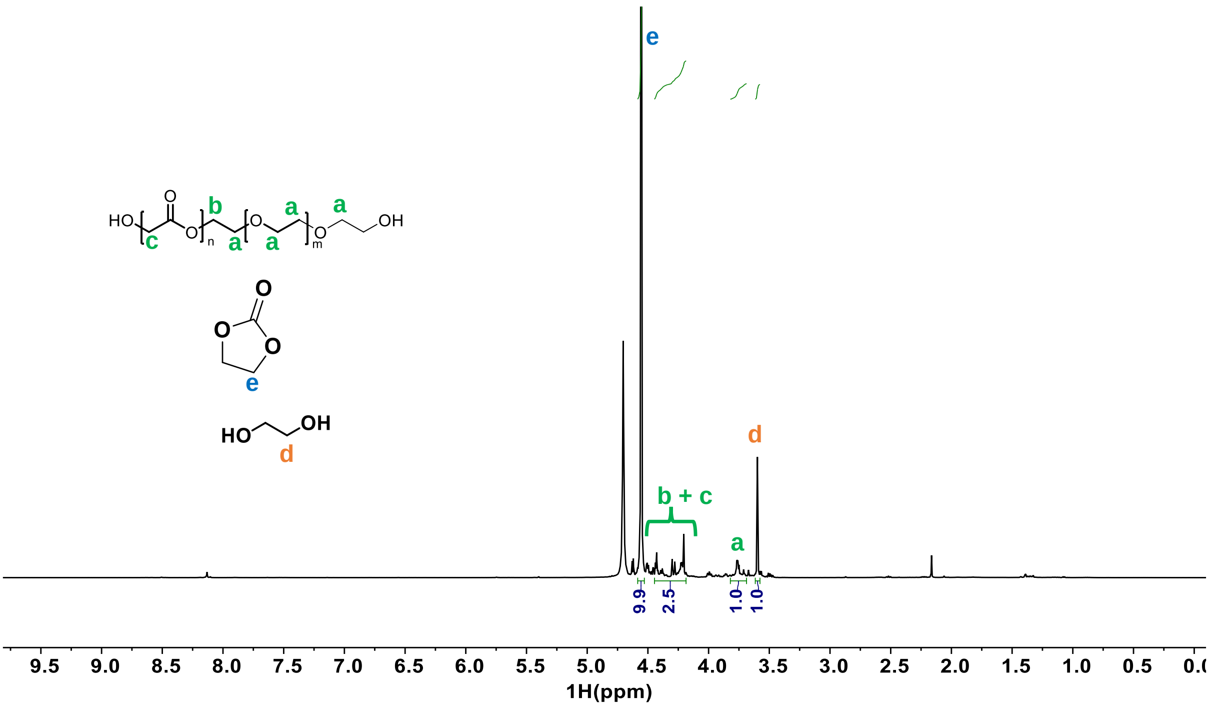

Figure S72. ^{1}H NMR (500 MHz, D_2O) spectrum of resulting mixture resulting from the reaction corresponding to Table S1; Entry 22. Ethylene carbonate (2.5 mmol) is used as an internal standard.

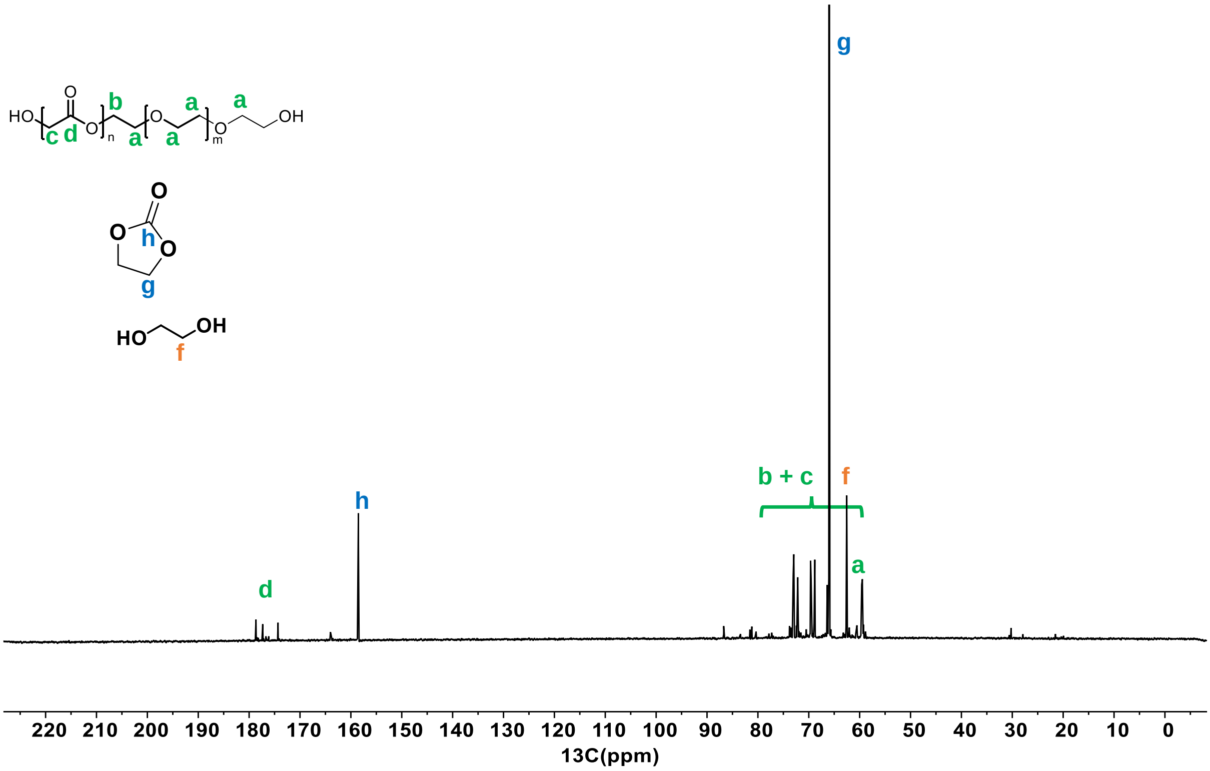

Figure S73. $^{13}C\{^{1}H\}$ NMR (126 MHz, D_2O) spectrum of the resulting mixture corresponding to Table S1; Entry 22. Ethylene Carbonate is used as an internal standard.

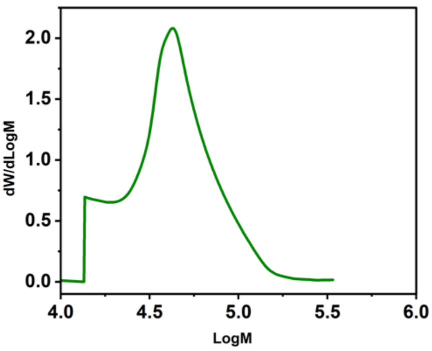

Figure S74 GPC data of the reaction mixture resulting from the reaction corresponding to Table S1; Entry 22.

Table S1; Entry 23:

The amount of ester has decreased in proportion to ether. It has changed from 4.6:1 (entry 5, table1) to 3.6:1 (figure shown below). It can be concluded from here that the amount of water present in the reaction significantly affects the degree of ether formation.

^{1}H NMR (500 MHz, D_2O): δH 4.4-4.0 (br,O-**CH$_2$**-CO-,CO-O-**CH$_2$**-CH$_2$), 3.8 (br, CH$_2$-**CH$_2$**-O-).

^{13}C{^{1}H} NMR (126 MHz, D_2O): δC 174.3 (-CH$_2$-**CO**-O), 73.1, 72.9, 71.9,69.6,68.8,66.4,64.8 (-O-**CH$_2$**-CO-, CO-O-**CH$_2$**-CH$_2$), 61.1, 59.1 (-CH$_2$-**CH$_2$**-O-).

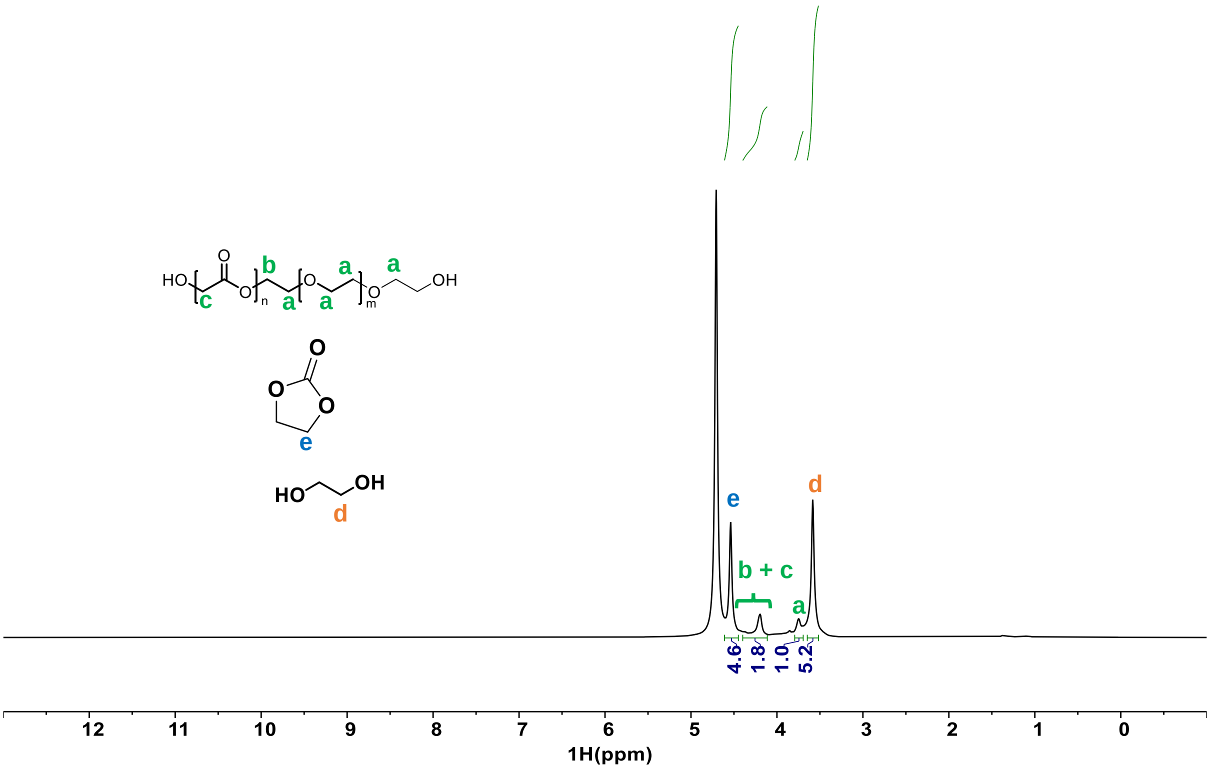

Figure S75. ^{1}H NMR (500 MHz, D_2O) spectrum of the reaction mixture resulting from the reaction corresponding to Table S1; Entry 23. Ethylene Carbonate (0.4 mmol) is used as an internal standard.

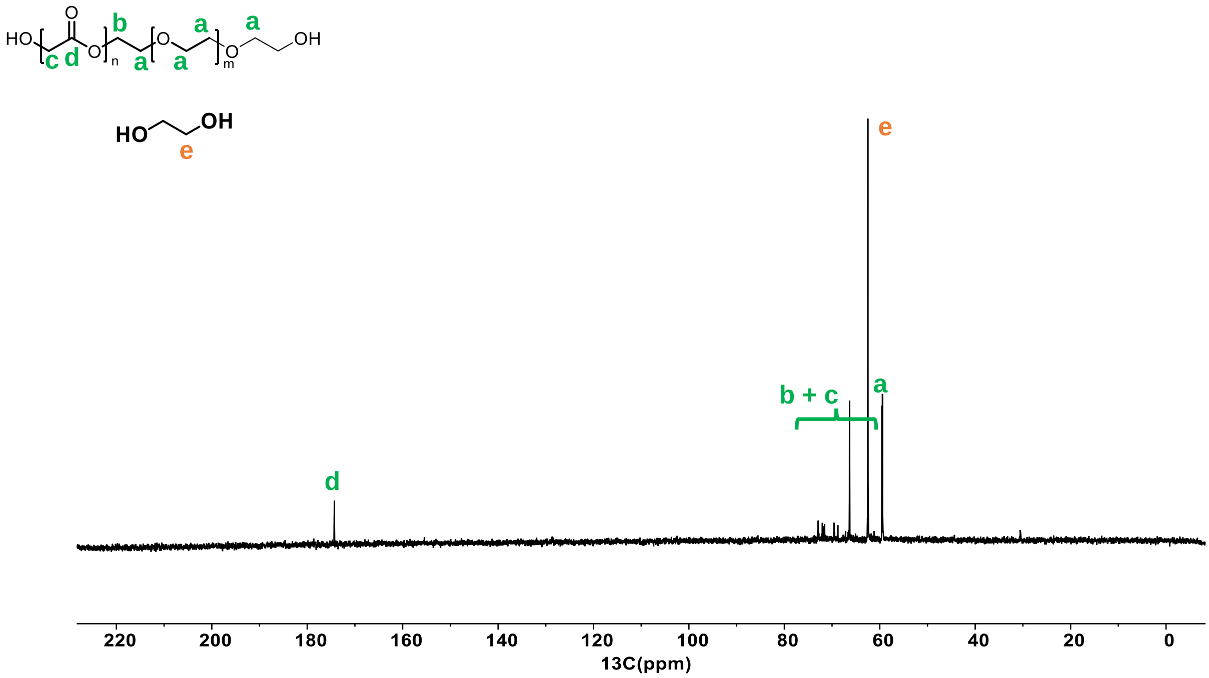

Figure S76. $^{13}C\{^{1}H\}$ NMR (126 MHz, D_2O) spectrum of the reaction mixture resulting from the reaction corresponding to Table S1; Entry 23.

1.10 Reaction with n-Propanol and n-Hexanol

Reaction with n-Propanol

^{1}H NMR (500 MHz, $CDCl_3$): δH 4.0 (br,-COO-**CH_2**-CH_2-CH_3), 2.3 (br,CH_3-**CH_2**-COO-), 1.8 (br,-COO-CH_2-**CH_2**-CH_3), 1.2(br,**CH_3**-CH_2-COO-), 1.0 (br,-COO-CH_2-CH_2-**CH_3**).

$^{13}C\{^{1}H\}$ NMR (126 MHz, $CDCl_3$): δC 174.4 (CH_3-CH_2-**CO**-O-), 64.6 (COO-**CH_2**-CH_2-CH_3), 30.1 (CH_3-**CH_2**-CO-O-), 22.0 COO-CH_2-**CH_2**-CH_3), 10.3 (**CH_3**-CH_2-CO-O-**,**COO-CH_2-CH_2-**CH_3**),

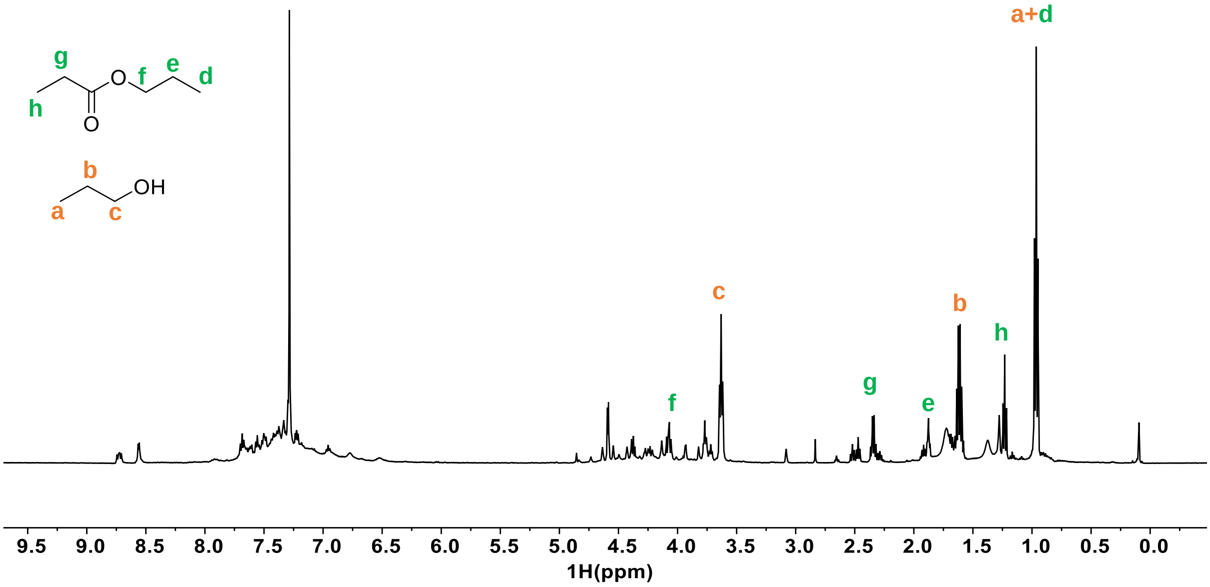

Figure S77. ^{1}H NMR (500 MHz, $CDCl_3$) spectrum of the reaction mixture resulting from the reaction with n-Propanol.

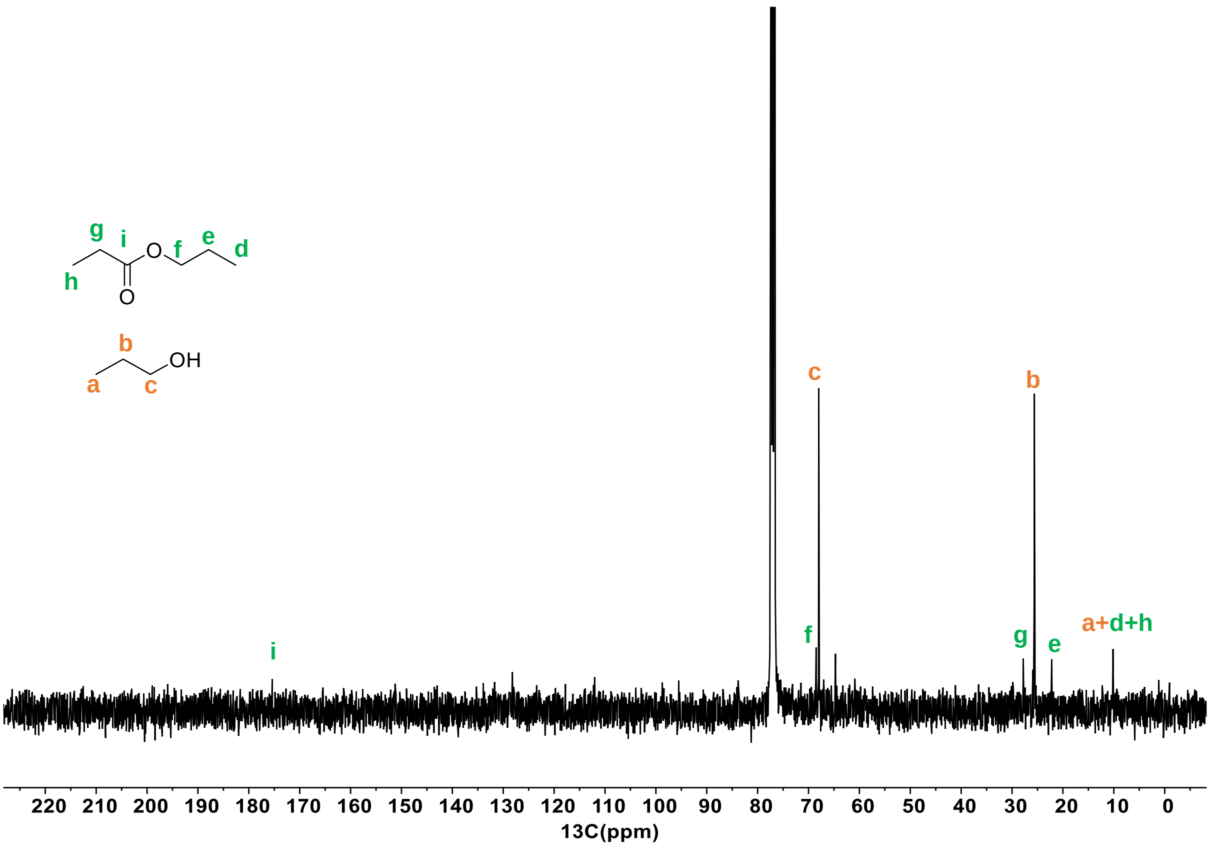

Figure S78. $^{13}C\{^{1}H\}$ NMR (126 MHz, $CDCl_3$) spectrum of the reaction mixture resulting from the reaction with n-Propanol.

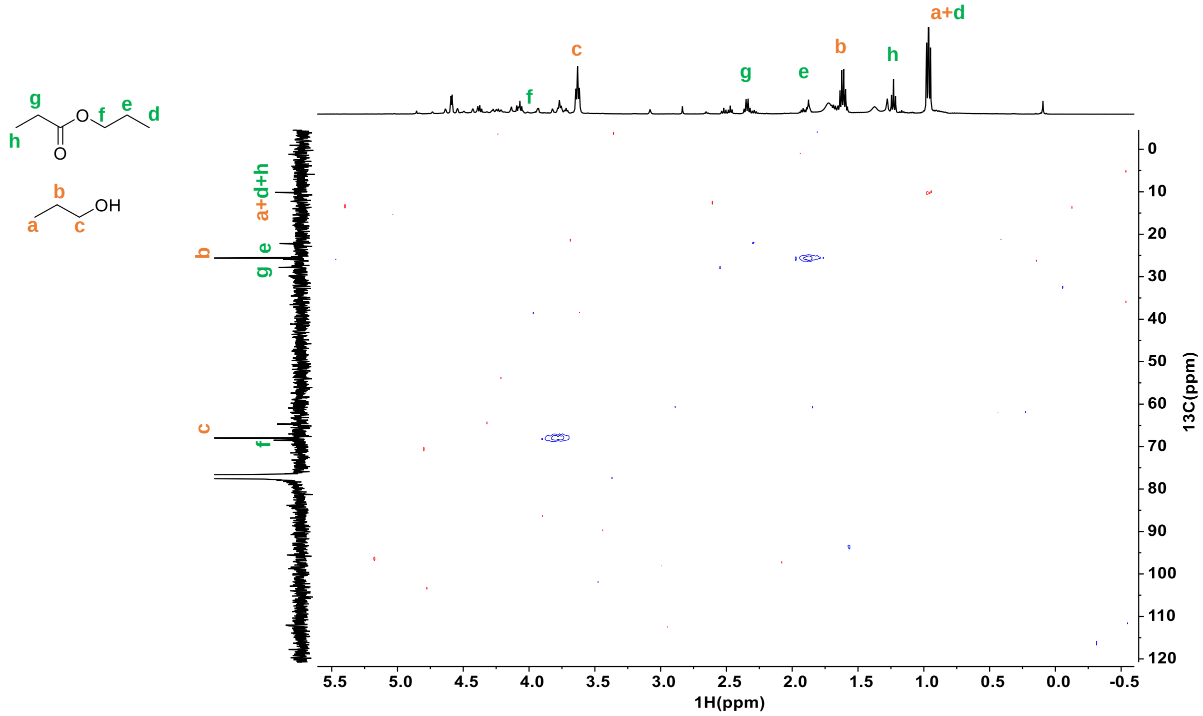

Figure S79. ^{1}H, ^{13}C- HSQC NMR (500-126 MHz, $CDCl_3$) spectrum of the reaction mixture resulting from the reaction with n-Propanol.

<u>Reaction with n-Hexanol</u>

^{1}H NMR (500 MHz, $CDCl_3$): δH 4.4-4.0 (br,-COO-**CH_2**-CH_2-CH_2-), 2.3 (br,-CH_2-**CH_2**-COO-), 1.6 (br,-COO-CH_2-**CH_2**-CH_2-,-CH_2-**CH_2**-CH_2-COO-), 1.3-1.0(br, CH_3- **CH_2- CH_2**-CH_2-CH_2-COO-**,**-COO-CH_2-CH_2- **CH_2-CH_2-CH_2**-CH_3),0.8 (br, **CH_3**- CH_2- CH_2-CH_2-CH_2-COO-**,**-COO-CH_2-CH_2- CH_2-CH_2-CH_2-**CH_3**).

^{13}C{^{1}H} NMR (126 MHz, $CDCl_3$): δC 173.7 (-CH_2-CH_2-**COO-**), 63.9 (COO-**CH_2**-CH_2-CH_2-), 33.8-30.9,29.5,25.6-22.9 (-CH_2-**CH_2**-COO-**,**-CH_2-**CH_2**-CH_2-COO-,CH_3-**CH_2**-CO-O-,-CH_3- **CH_2- CH_2**-CH_2-CH_2-COO-**,**-COO-CH_2-CH_2- **CH_2-CH_2-CH_2**-CH_3), 14.4 **CH_3**- CH_2- CH_2-CH_2-CH_2-COO-**,**-COO-CH_2-CH_2- CH_2-CH_2-CH_2-**CH_3**).

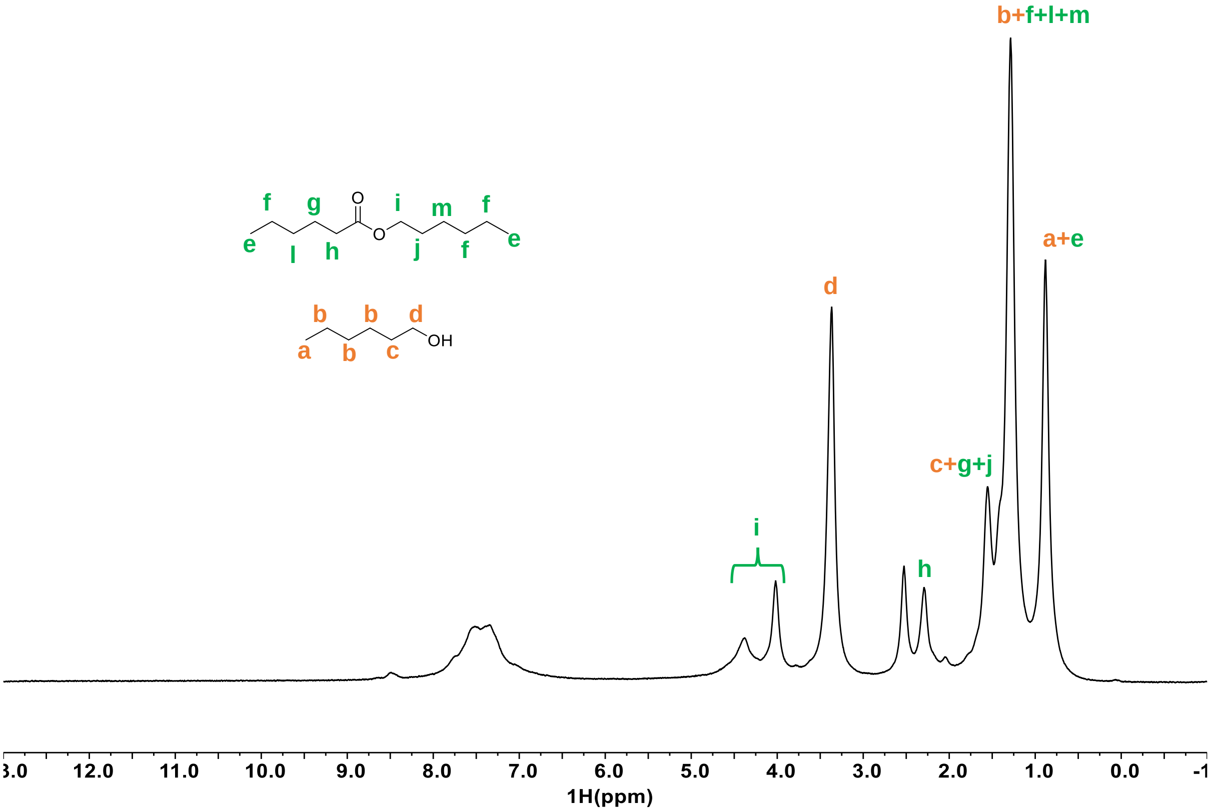

Figure S80. ^{1}H NMR (500 MHz,DMSO-d_6) spectrum of the reaction mixture resulting from the reaction with n-Hexanol.

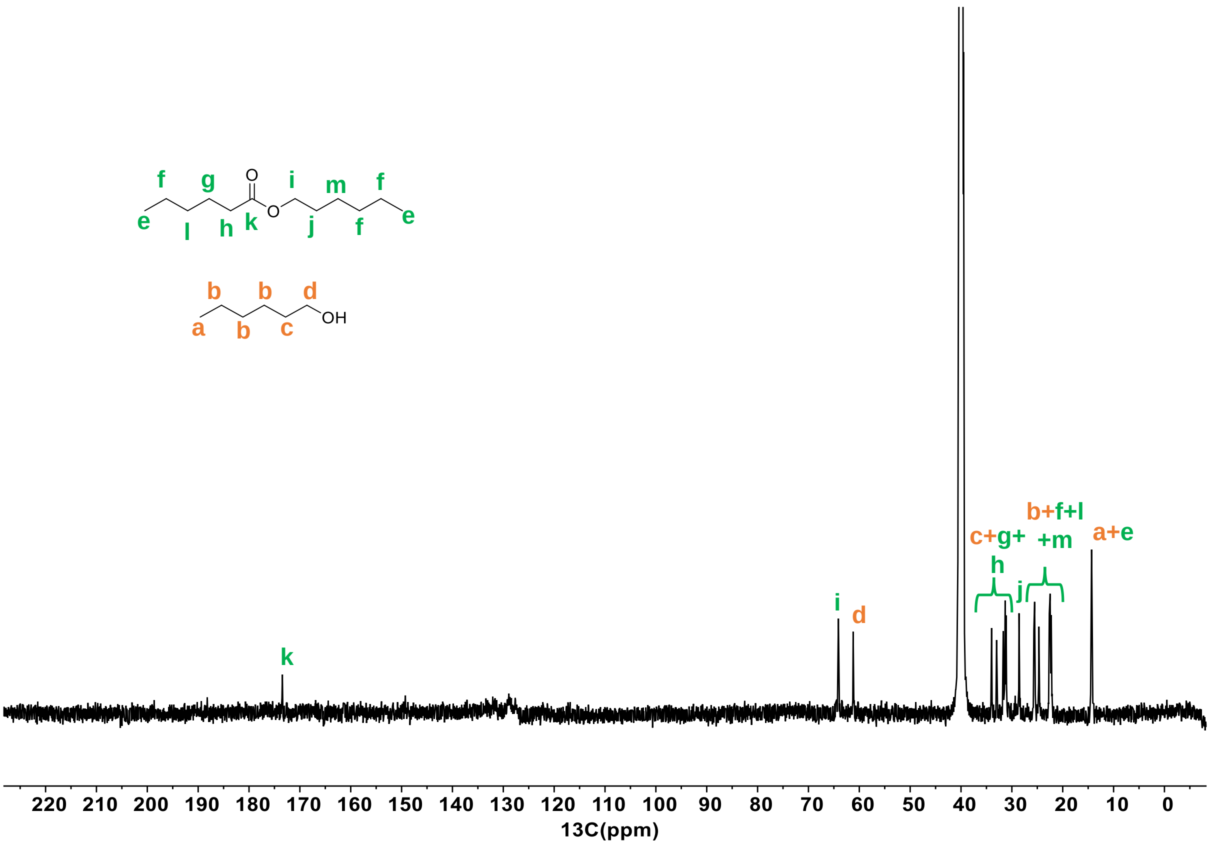

Figure S81. $^{13}C\{^{1}H\}$ NMR (126 MHz, DMSO-d_6) spectrum of the reaction mixture resulting from the reaction with n-Hexanol.

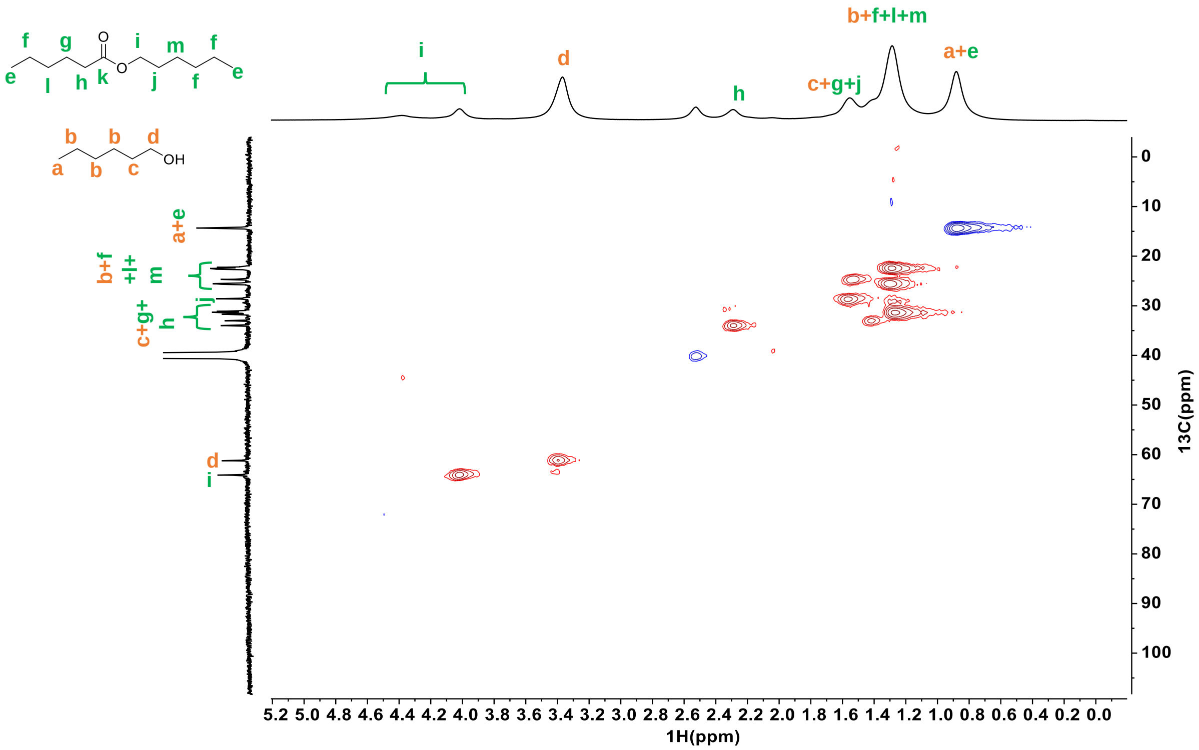

Figure S82. ^{1}H, ^{13}C- HSQC NMR (500-126 MHz, DMSO-d_6) spectrum of the reaction mixture resulting from the reaction with n-Hexanol.

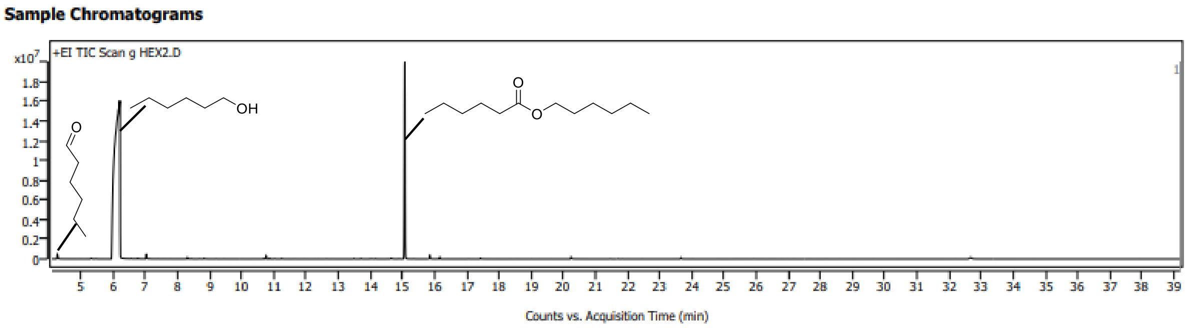

Figure S83. EI- TIC Scan of the reaction mixture resulting from the reaction with n-Hexanol.

1.11 Substrate scope

Table S2. Polymerisation of different diols

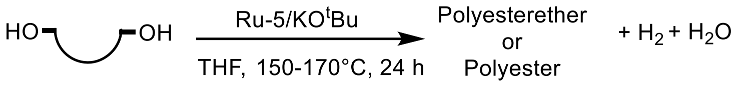

Entry no	Diol	Gas released (mL)[b]	Conversion (%)[c]	Molar mass, Mn	Đ	Ester:Ether
1	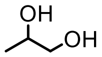	25	78	33,930	1.4	0.8:1
2[d]	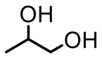	45	86	10,110	1.8	0.9:1
3	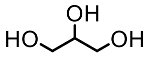	8	-	37,350	1.4	-
4	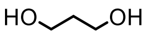	27	92	34,870	1.3	1.8:1
5	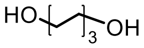	14	30	37,700	1.2	-
6	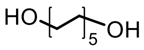	1	33	-	-	-
7	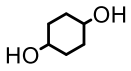	24	12	-	-	-

[a]Reaction Conditions: Diol: 2 mmol, THF: 2 mL, **Ru-5** (1 mol%), KO^{t}Bu (2 mol%), 150 °C, 24 h. [b]In all cases released gas was identified to be mainly H_2 with ≤1% of CO using GC-TCD analysis. [c]Conversion was determined by the ^{1}H NMR spectroscopy of the crude reaction mixture using ethylene carbonate as an internal standard and D_2O as solvent. [d]Reaction conducted at 170°C.

<u>Table S2; Entry 1:</u>

Product formed: Polyesterether

^{1}H NMR (500 MHz, D_2O): δH 4.1 (br,-O-**CH**(CH$_3$)-CO-,-COO-**CH$_2$**-CH(CH$_3$)-O-), 3.5-3.3 (br,-CH$_2$-**CH**(CH$_3$)-O-,-O-**CH$_2$**-CO-CH$_3$), 1.5-1.3 (br, -CH$_2$-**CH$_3$**).

^{13}C{^{1}H} NMR (126 MHz, D_2O): δC 182.6, 181.5, 176.3 (-CH(CH$_3$)-**CO**-O-), 80.3, 78.9, 77.7, 76.4, 73.2 (-O-**CH**(CH$_3$)-CO-,-COO-**CH$_2$**-CH(CH$_3$)-O-), 70.6, 69.8, 62.5 (-CH$_2$-**CH**(CH$_3$)-O-,-O-**CH$_2$**-CH(CH$_3$)-O-), 27.7 (-CH$_2$-CO-**CH$_3$**), 21.5, 20.5, 16.5, 13.2 (-CH$_2$-**CH$_3$**).

GPC: Mn =33,930 gmol^{-1} (Đ =1.4).

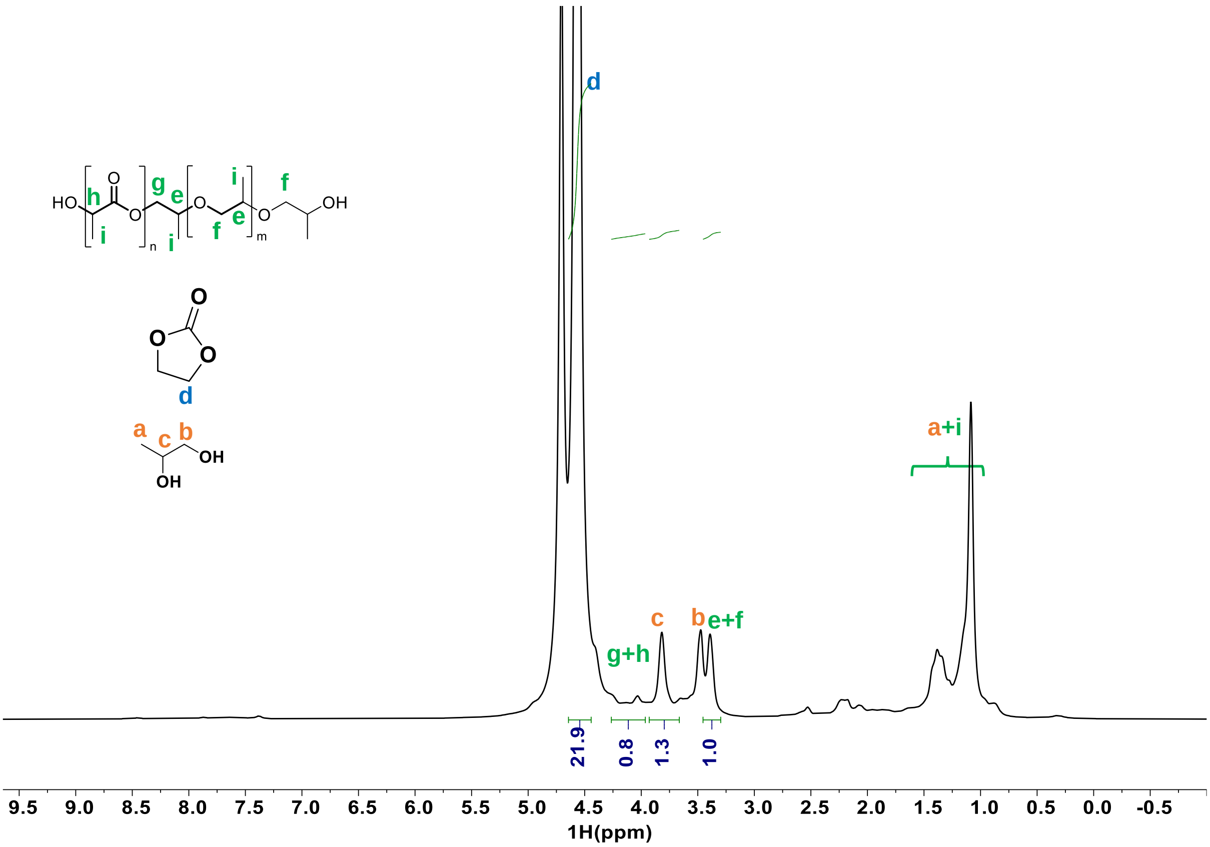

Figure S84. ^{1}H NMR (500 MHz, D_2O) spectrum of the reaction mixture resulting from the reaction corresponding to Table S2; Entry 1. Ethylene Carbonate (1.8 mmol) is used as an internal standard.

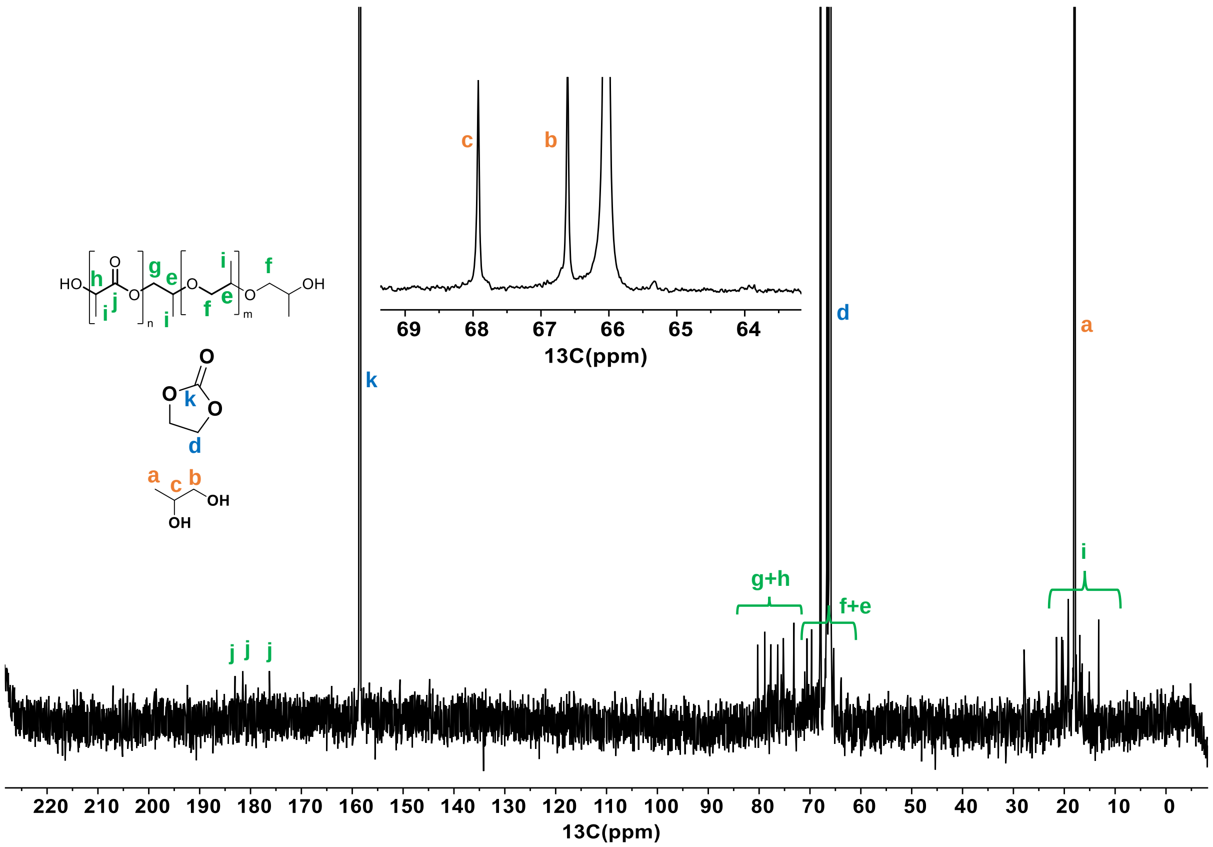

Figure S85. $^{13}C\{^{1}H\}$ NMR (126 MHz, D_2O) spectrum of the reaction mixture resulting from the reaction corresponding to Table S2; Entry 1. Ethylene Carbonate is used as an internal standard.

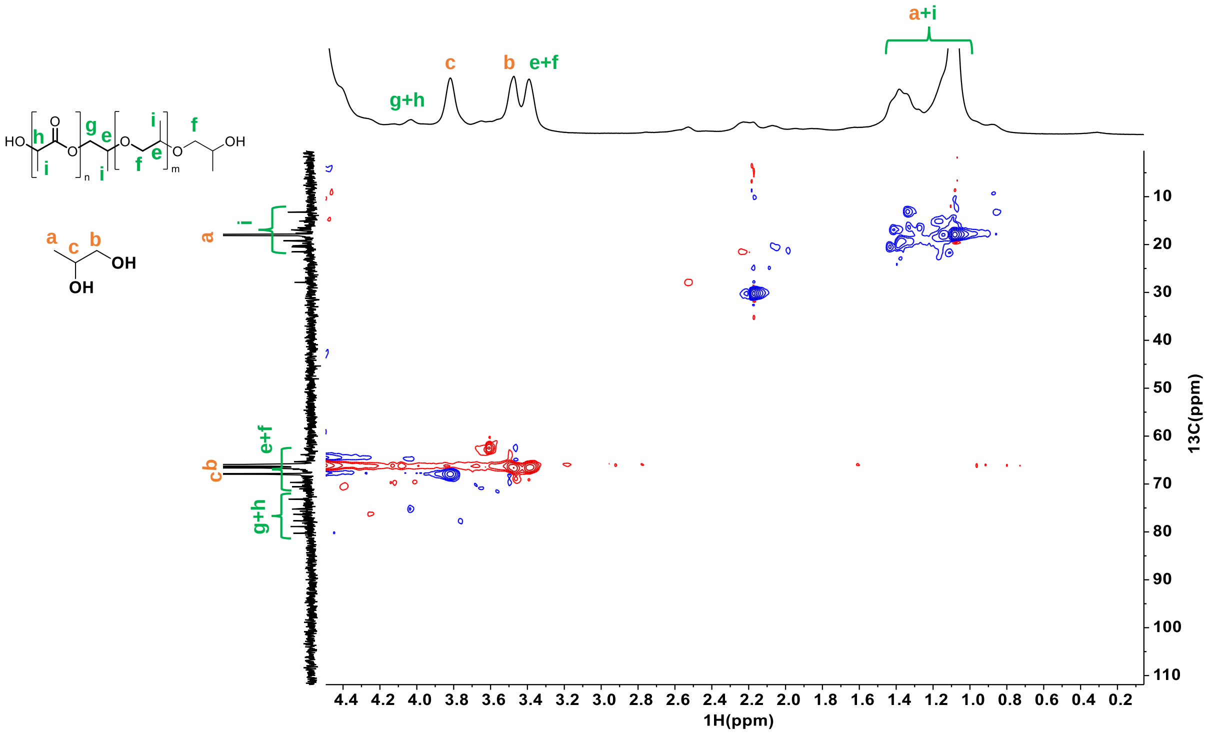

Figure S86. ^{1}H, ^{13}C- HSQC NMR (500-126 MHz, D_2O) spectrum of the reaction mixture resulting from the reaction corresponding to Table S2; Entry 1. Ethylene Carbonate is used as an internal standard.

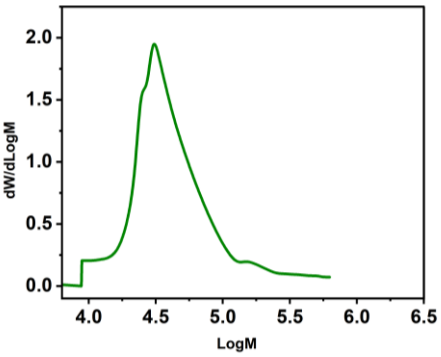

Figure S87. GPC data of the reaction mixture resulting from the reaction corresponding to Table S2; Entry 1.

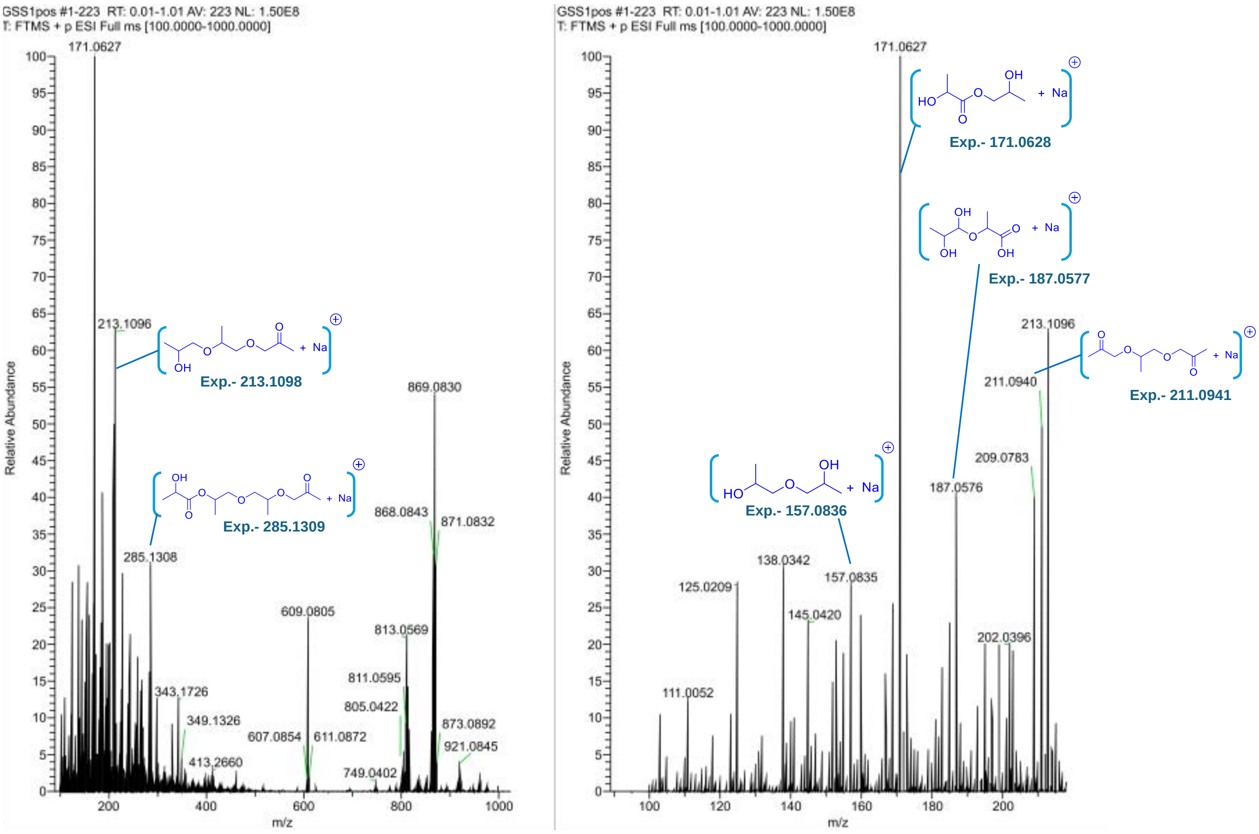

Figure S88. ESI- MS the reaction mixture resulting from the reaction corresponding to Table S2.

Table S2; Entry 2:

Product formed: Polyesterether

^{1}H NMR (500 MHz, $CDCl_3$): δH 4.1 (br,-O-**CH**(CH$_3$)-CO-,-COO-**CH$_2$**-CH(CH$_3$)-O-), 3.-3.3 (br,-CH$_2$-**CH**(CH$_3$)-O-,-O-**CH$_2$**-CO-CH$_3$), 2.3 (br,-O-CH$_2$-CO-**CH$_3$**), 1.5-1.3 (br, -CH$_2$-**CH$_3$**).

^{13}C{^{1}H} NMR (126 MHz, $CDCl_3$): δC 192.2 (-**CO**-CH$_3$), 182.6, 181.5, 176.3 (-CH(CH$_3$)-**CO**-O-), 80.3, 78.9, 77.7, 76.4, 73.2 (-O-**CH**(CH$_3$)-CO-,-COO-**CH$_2$**-CH(CH$_3$)-O-), 70.6, 69.8, 62.5 (-CH$_2$-**CH**(CH$_3$)-O-,-O-**CH$_2$**-CH(CH$_3$)-O-), 27.7 (-CH$_2$-CO-**CH$_3$**), 21.5, 20.5, 16.5, 13.2 (-CH$_2$-**CH$_3$**).

GPC: Mn =10,110 gmol^{-1} (Đ =1.8).

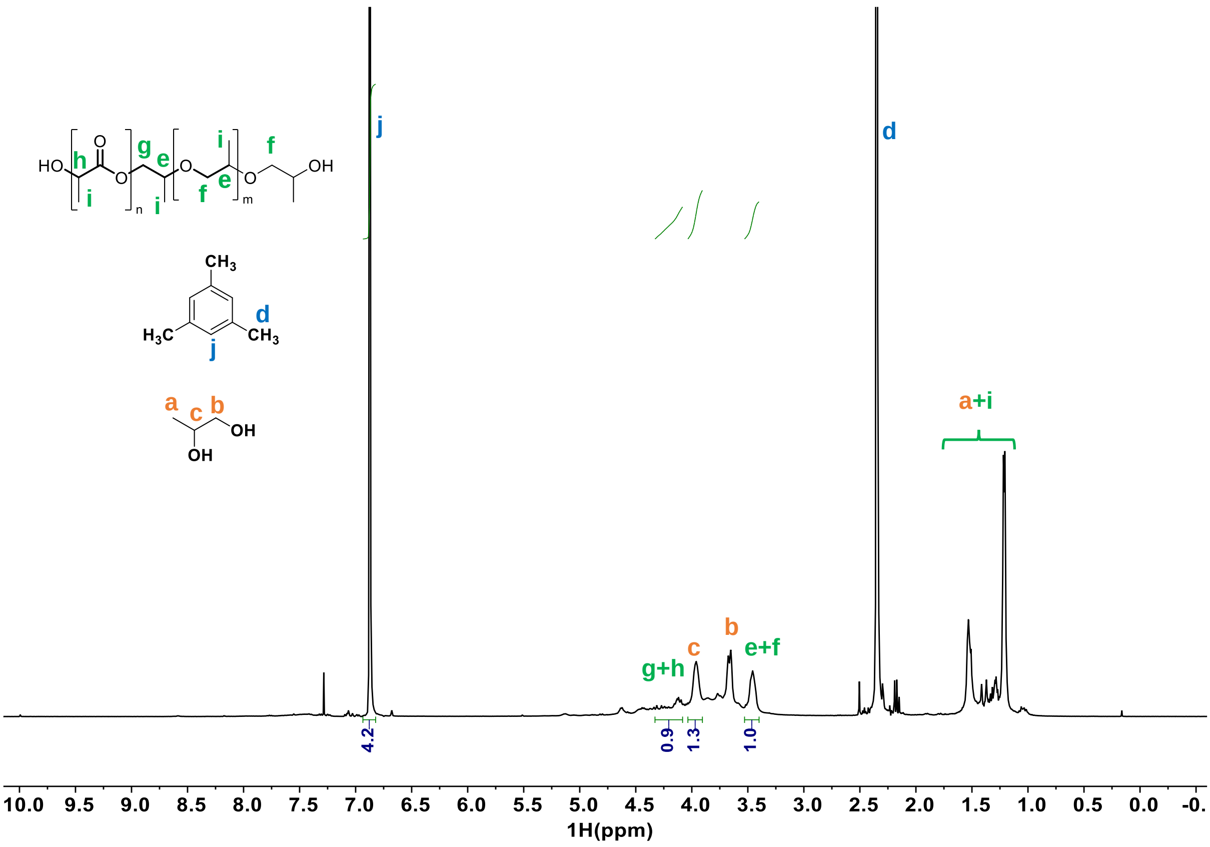

Figure S89. ^{1}H NMR (500 MHz, $CDCl_3$) spectrum of the reaction mixture resulting from the reaction corresponding to Table S2; Entry 2. Mesitylene (0.3 mmol) is used as an internal standard.

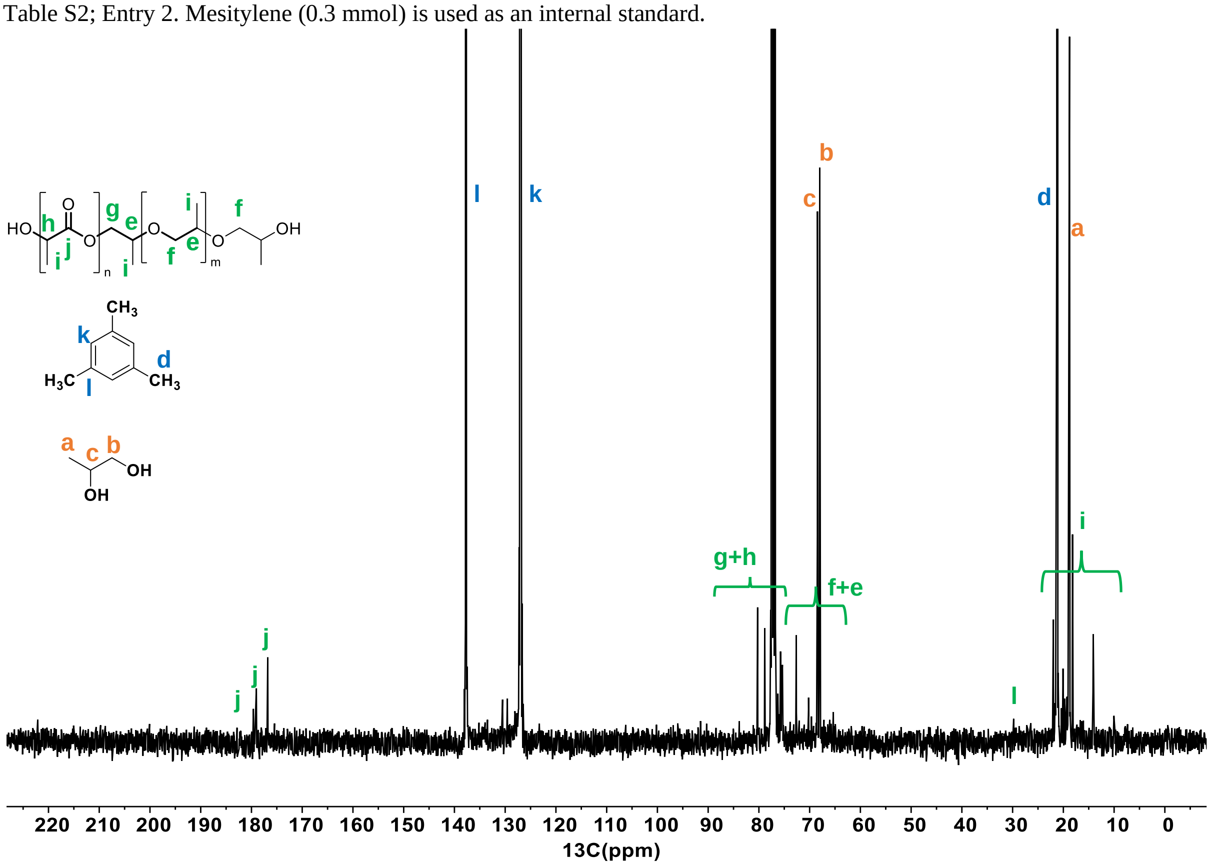

Figure S90. $^{13}C\{^{1}H\}$ NMR (126 MHz, $CDCl_3$) spectrum of the reaction mixture resulting from the reaction corresponding to Table S2; Entry 2. Mesitylene is used as an internal standard.

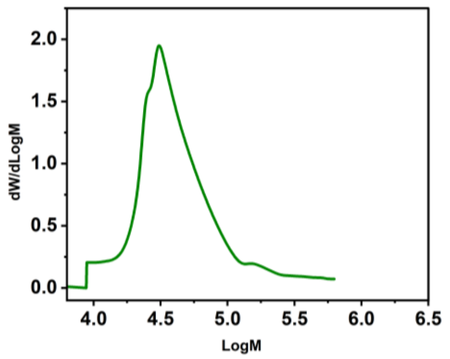

Figure S91. GPC data of the reaction mixture resulting from the reaction corresponding to Table S2; Entry 2.

Table S2; Entry 3:

Product formed: Polyesterether

^{1}H NMR (500 MHz, D_2O): δH 4.2-3.9 (br,-O-**CH$_2$**(**CH**-OH)-CO-,-COO-**CH$_2$**(CH-OH)-CO-), 3.5-3.3 (br,-O-**CH$_2$**(CH-OH)-CH$_2$-O-,-O-CH$_2$(**CH**-OH)-CH$_2$-O-,-O-CH$_2$(CH-OH)-**CH$_2$**-O-).

^{13}C{^{1}H} NMR (126 MHz, D_2O): δC 183.0, 176.2, 174.1 (-CH$_2$(CH-OH)-**CO**-O-), 86.7, 81.5, 80.4, 77.8, 73.0, 71.6(,-O-**CH$_2$**(**CH**-OH)-CO-,-COO-**CH$_2$**(CH-OH)-CO-), 68.8, 67.9, 66.6, 65.1, 60.1, 59.7, 59.3(-O-**CH$_2$**(**CH**-OH)-**CH$_2$**-O-)

GPC: Mn =37,350 gmol^{-1} (Đ =1.4).

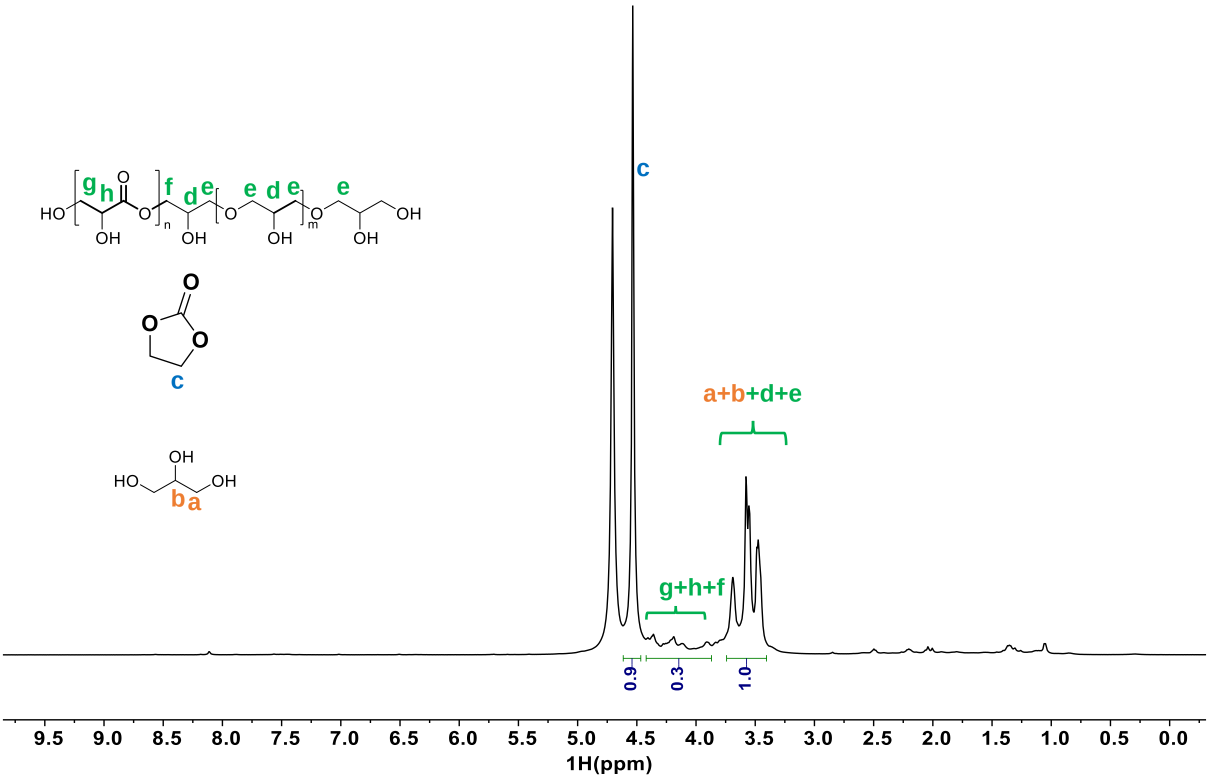

Figure S92. ^{1}H NMR (500 MHz, D_2O) spectrum of the reaction mixture resulting from the reaction corresponding to Table S2; Entry 3. Ethylene Carbonate(0.6 mmol) is used as an internal standard.(Substrate conversion and ester : ether cannot be calculated in this case due to overlapping of substrate and product signals).

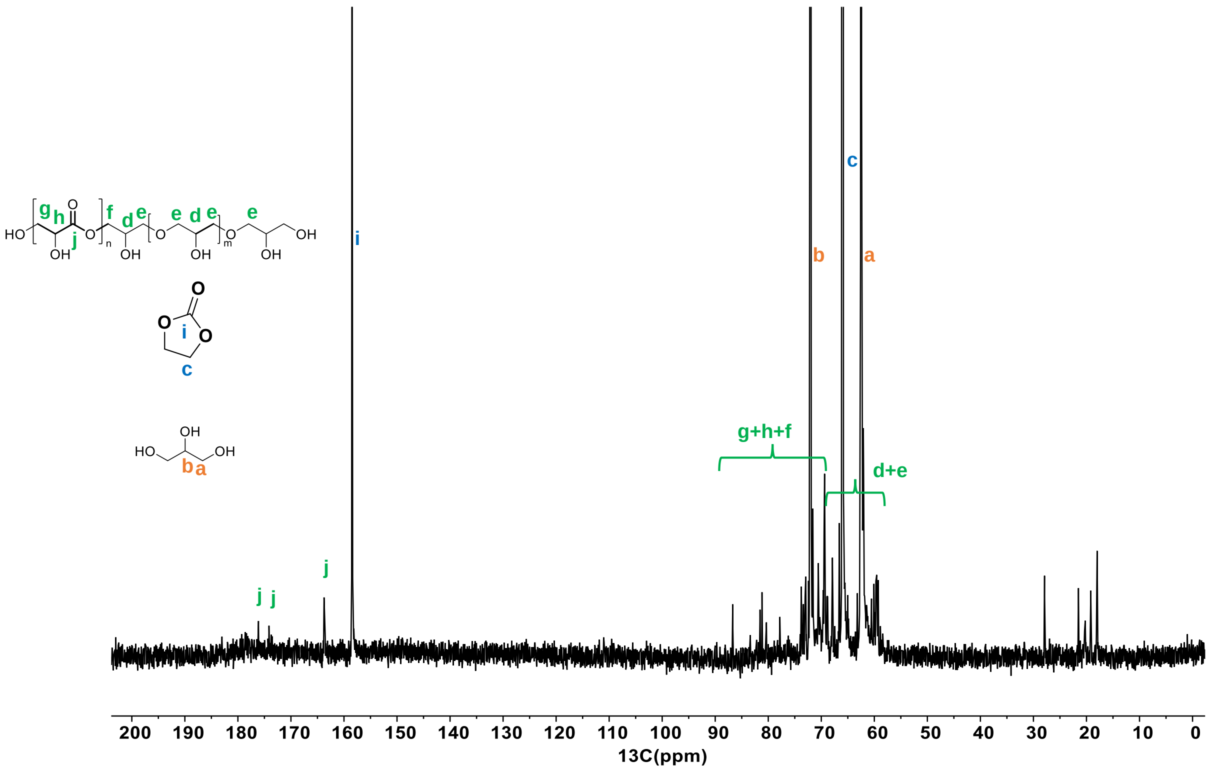

Figure S93. $^{13}C\{^{1}H\}$ NMR (126 MHz, D_2O) spectrum of the reaction mixture resulting from the reaction corresponding to Table S2; Entry 3. Ethylene Carbonate is used as an internal standard.

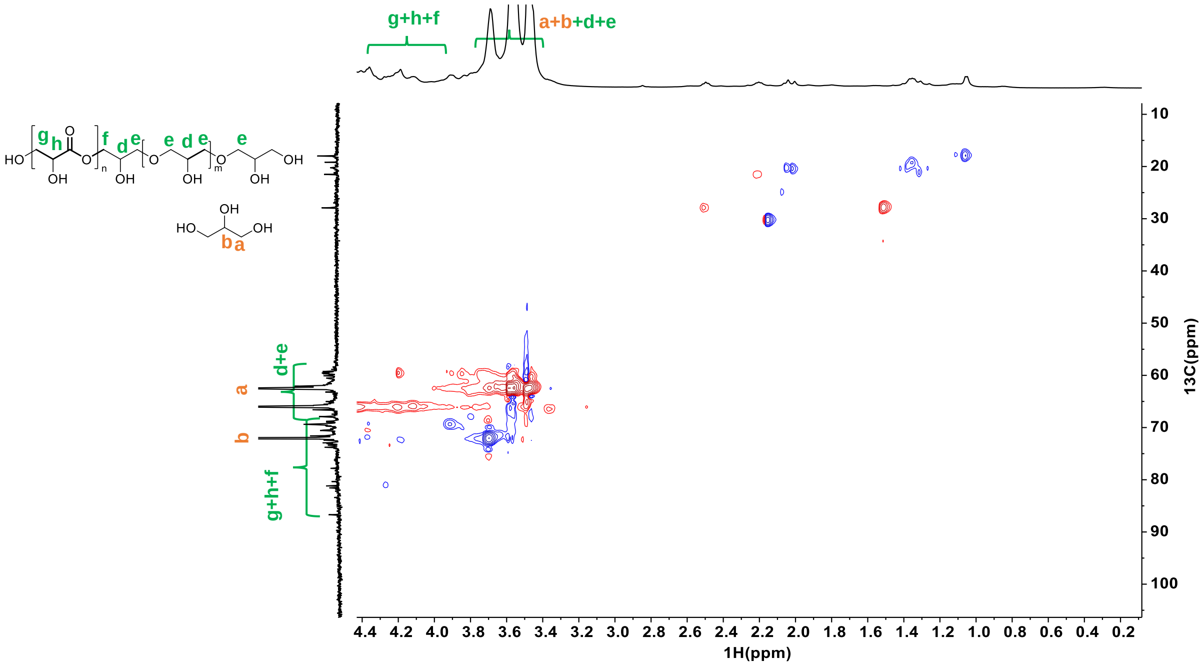

Figure S94. ^{1}H, ^{13}C- HSQC NMR (500-126 MHz, D_2O) spectrum of the reaction mixture resulting from the reaction corresponding to Table S2; Entry 3. Ethylene Carbonate is used as an internal standard.

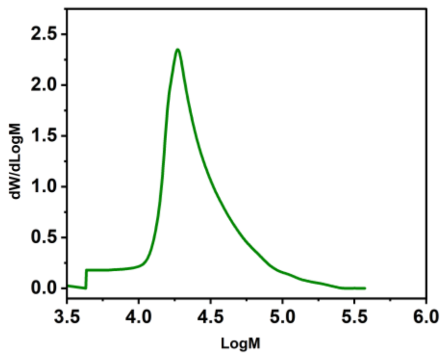

Figure S95. GPC data of the reaction mixture resulting from the reaction corresponding to Table S2; Entry 3.

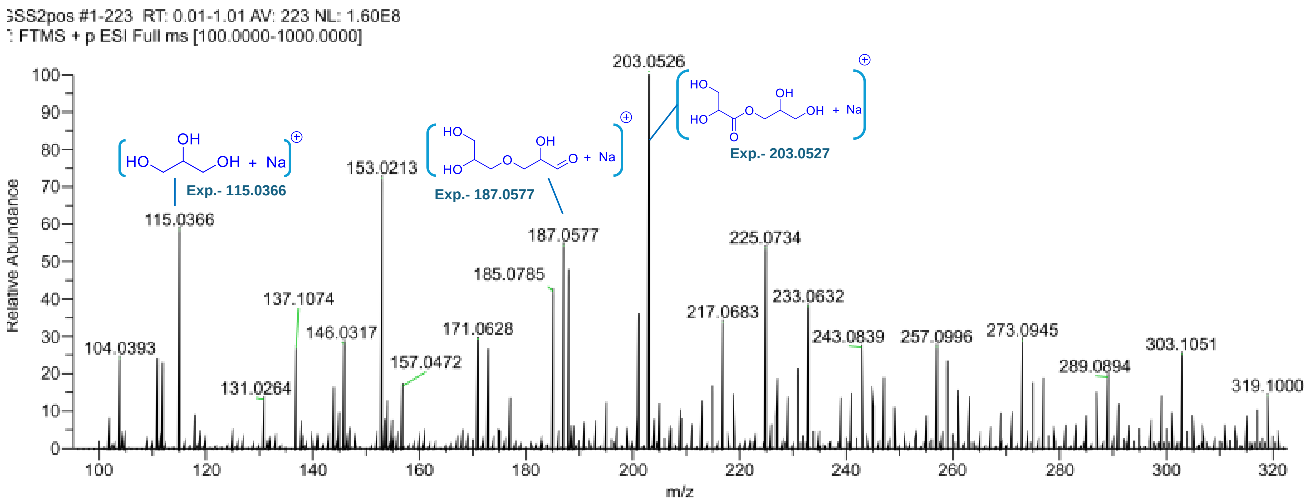

Figure S96. ESI- MS the reaction mixture resulting from the reaction corresponding to Table S2; Entry 3.

Table S2; Entry 4:

Product formed: Polyesterether

^{1}H NMR (500 MHz, D_2O): δH 4.2 (br,-COO-**CH$_2$**-CH$_2$-CO-), 3.8 (br,-O-**CH$_2$**-CH$_2$-CO-), 3.5 (br,-O-CH$_2$-CH$_2$-**CH$_2$**-O-,-O-**CH$_2$**-CH$_2$-CH$_2$-O-), 2.6-2.4 (br,-O-CH$_2$-**CH$_2$**-CO-), 1.8 (br,-O-CH$_2$-**CH$_2$**-CH$_2$-O-)

^{13}C{^{1}H} NMR (126 MHz, D_2O): δC 179.6, 174.5 (-O-CH$_2$-CH$_2$-**CO** -), 88.8, 71.2, 69.8, 67.5, 67.2(-O-**CH$_2$**-CH$_2$-CH$_2$-O-,-O-CH$_2$-CH$_2$-**CH$_2$**-O-), 62.4, 61.5,61.2,57.7(-O-**CH$_2$**-CH$_2$-CO-,-COO-**CH$_2$**-CH$_2$-CO-), 39.3, 38.9, 37.0,33.6, 30.1, 29.3, 27.9, 26.9,23.5, 21.5(-O-CH$_2$-**CH$_2$**-CO-,-O-CH$_2$-**CH$_2$**-CH$_2$-O-)

GPC: Mn = 34,870 gmol^{-1} (Đ =1.3).

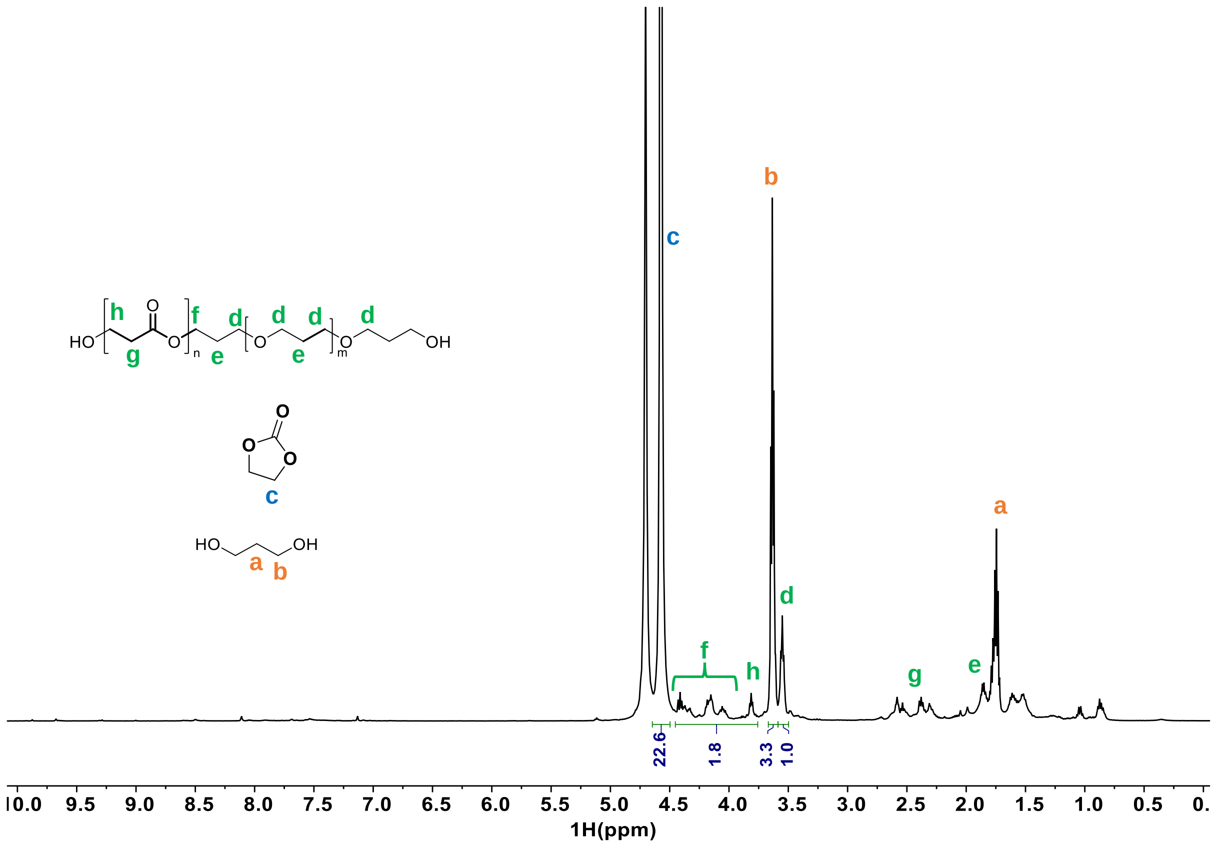

Figure S97. ^{1}H NMR (500 MHz, D_2O) spectrum of the reaction mixture resulting from the reaction corresponding to Table S2; Entry 4. Ethylene Carbonate (1 mmol) is used as an internal standard.

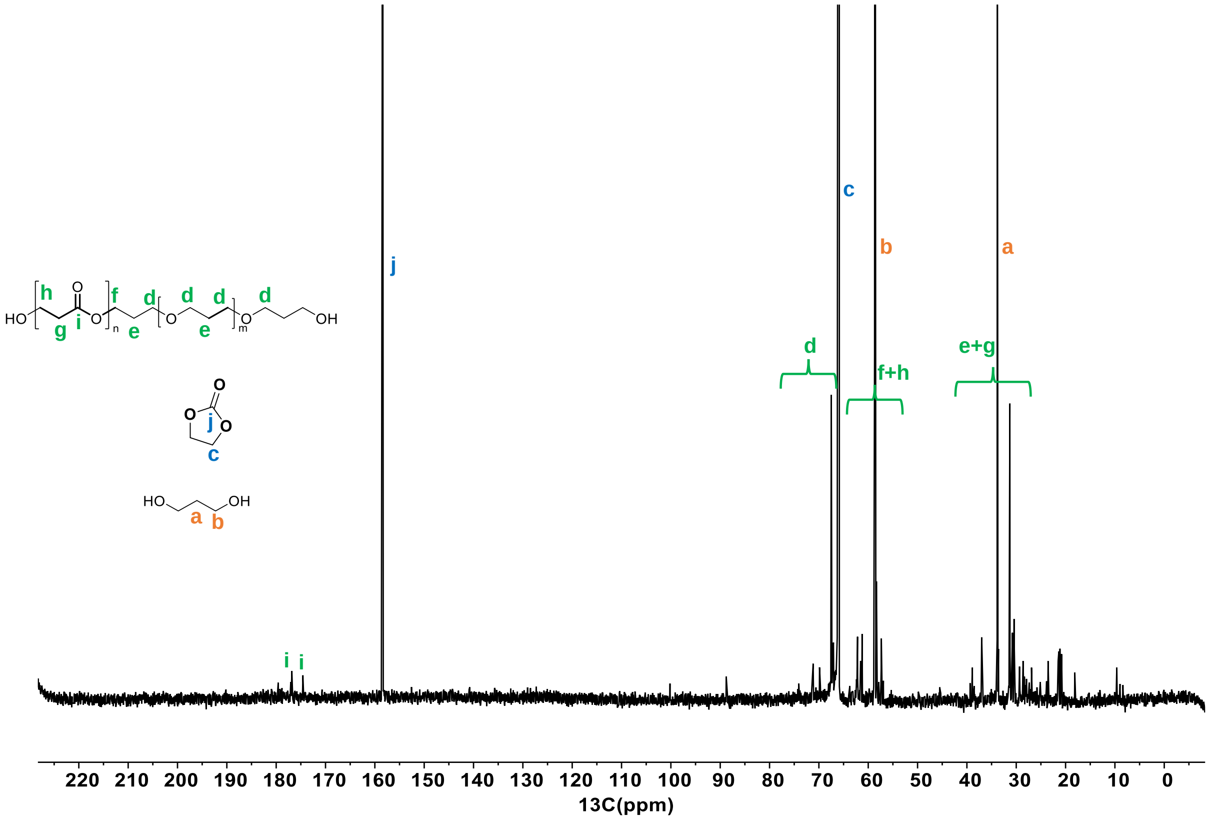

Figure S98. $^{13}C\{^{1}H\}$ NMR (126 MHz, D_2O) spectrum of the reaction mixture resulting from the reaction corresponding to Table S2; Entry 4. Ethylene Carbonate is used as an internal standard.

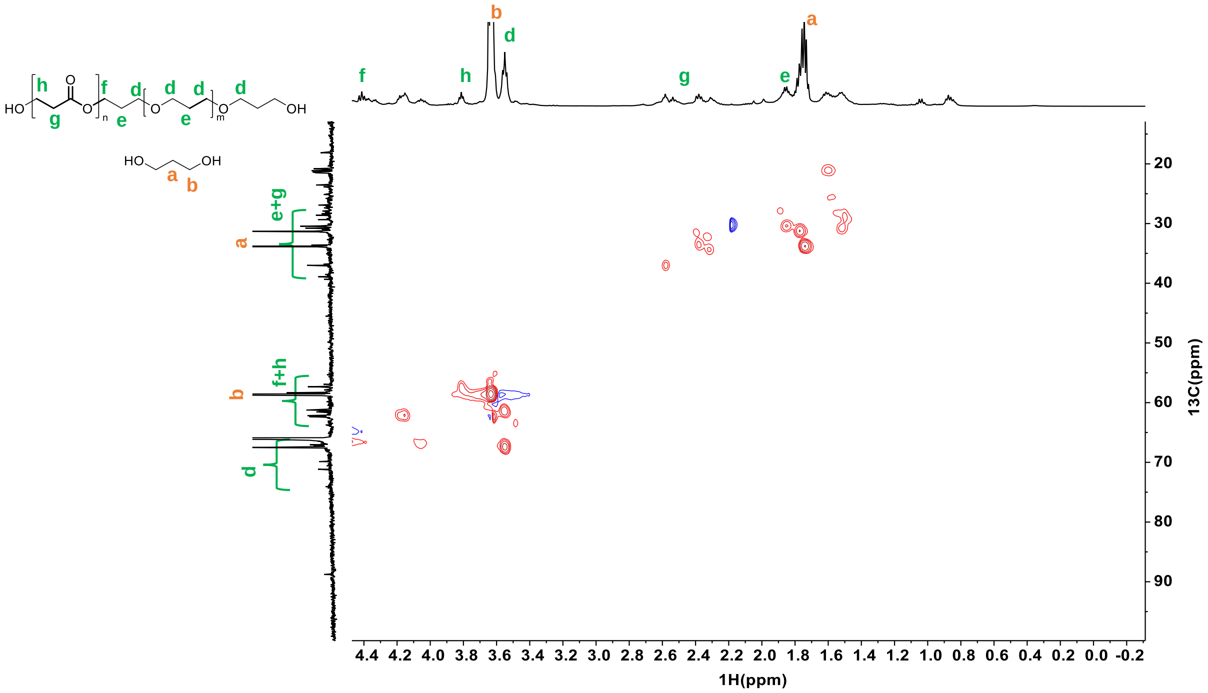

Figure S99. ^{1}H, ^{13}C- HSQC NMR (500-126 MHz, D_2O) spectrum of the reaction mixture resulting from the reaction corresponding to Table S2; Entry 4. Ethylene Carbonate is used as an internal standard.

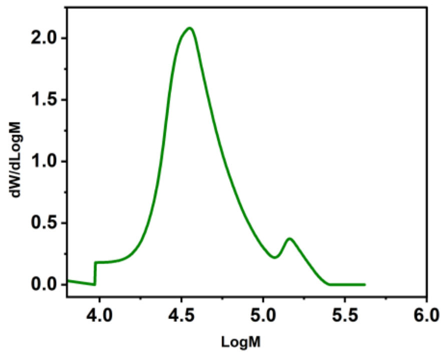

Figure S100. GPC data of the reaction mixture resulting from the reaction corresponding to Table S2; Entry 4.

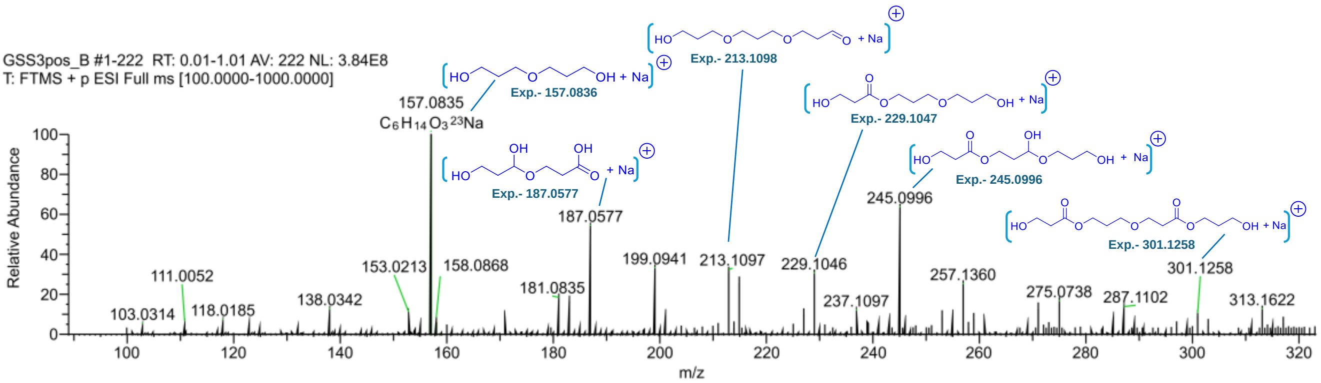

Figure S101. ESI- MS the reaction mixture resulting from the reaction corresponding to Table S2; Entry 4.

Table S2; Entry 5:

Product formed: Polyester

^{1}H NMR (500 MHz, D_2O): δH 4.1 (br,-O-**CH_2**-CH_2- CH_2-CH_2-CH_2-CO-), 2.3 (br,-O-CH_2-CH_2- CH_2-CH_2-**CH_2**-CO-), 1.8-1.2 (br,-O-CH_2-**CH_2**- **CH_2-CH_2**-CH_2-CO-).

^{13}C{^{1}H} NMR (126 MHz, D_2O): δC 176.9 (-O-CH_2-CH_2- CH_2-CH_2-CH_2-**CO**-), 65.4 (-O-**CH_2**-CH_2- CH_2-CH_2-CH_2-CO- 34.1(-O-CH_2-**CH_2**- CH_2-CH_2-**CH_2**-CO-), 27.9 (,-O-CH_2-CH_2- **CH_2-CH_2**-CH_2-CO-).

GPC: Mn = 37,700 gmol^{-1} (Đ =1.2).

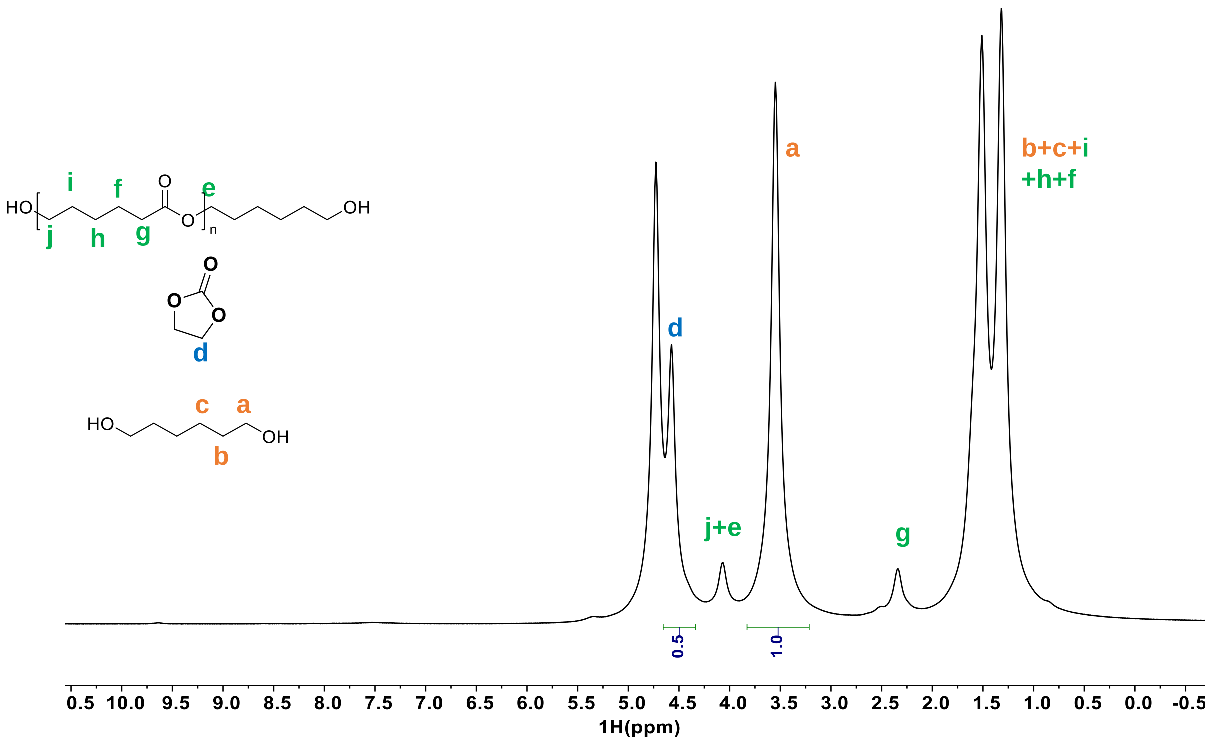

Figure S102. ^{1}H NMR (500 MHz, D_2O) spectrum of the reaction mixture resulting from the reaction corresponding to Table S2; Entry 5. Ethylene Carbonate (0.7 mmol) is used as an internal standard.

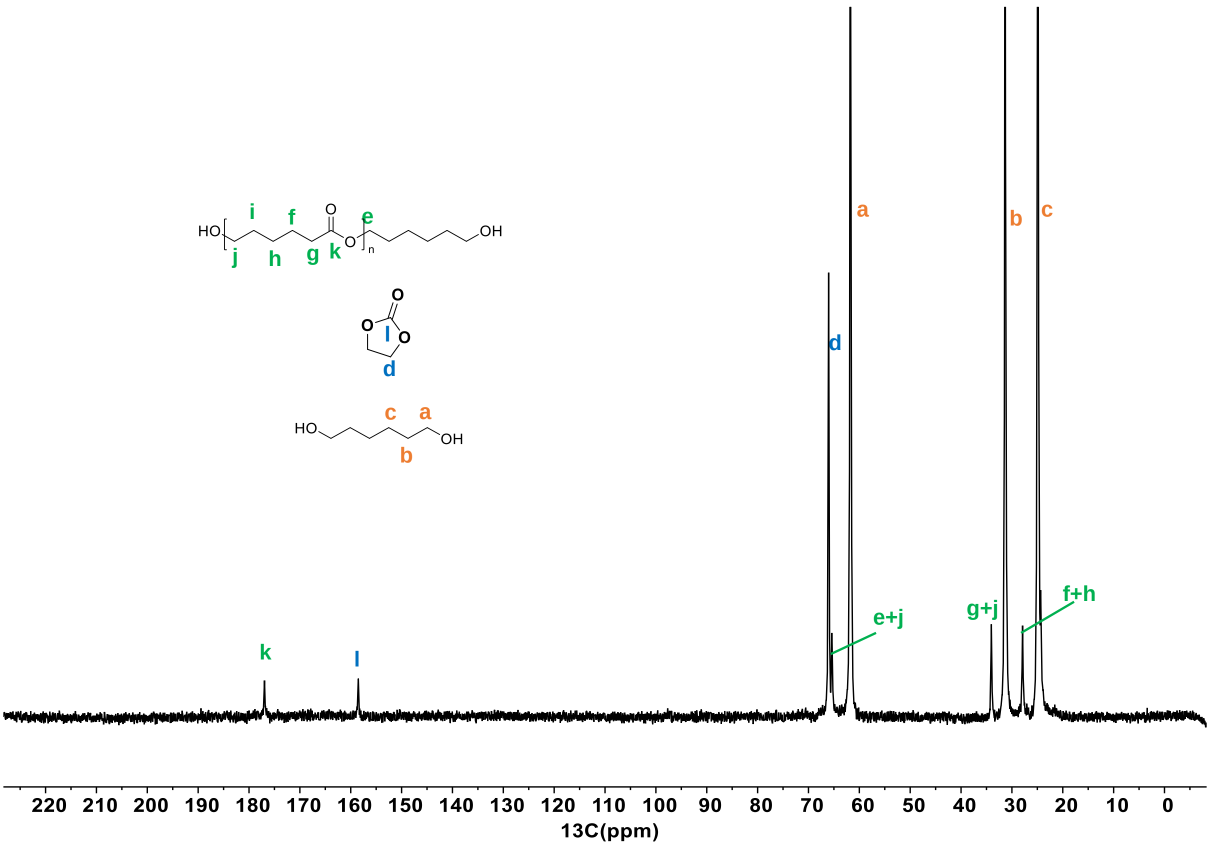

Figure S103. $^{13}C\{^{1}H\}$ NMR (126 MHz, D_2O) spectrum of the reaction mixture resulting from the reaction corresponding to Table S2; Entry 5. Ethylene Carbonate is used as an internal standard.

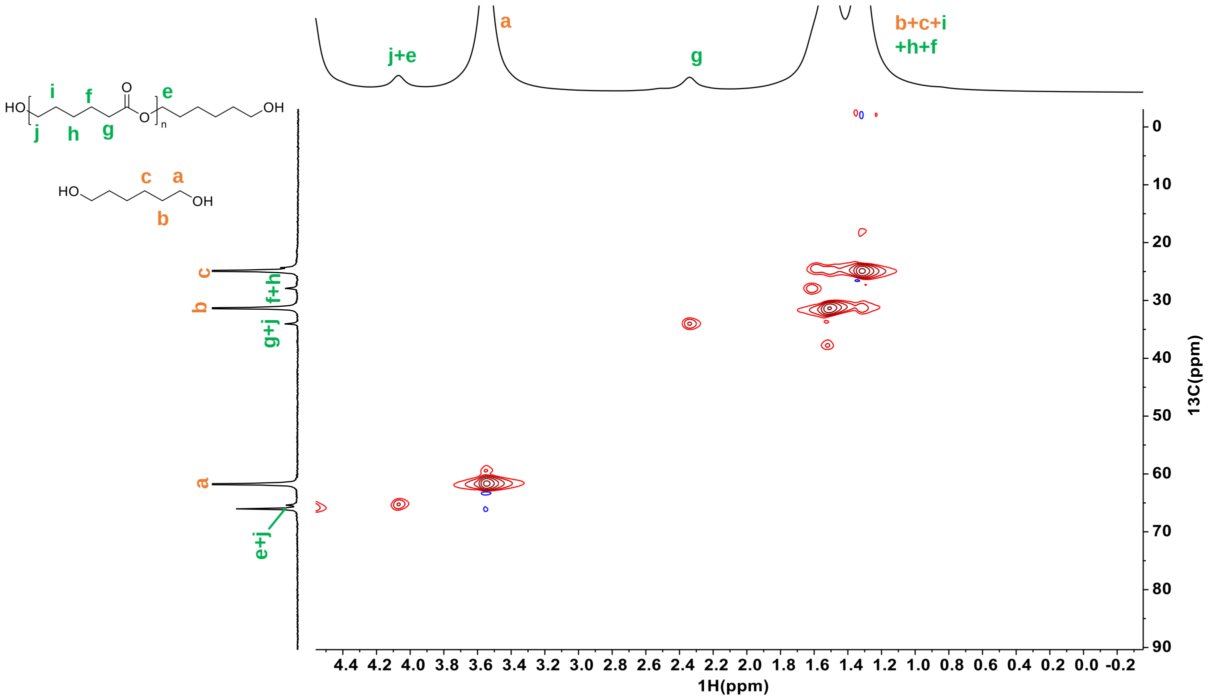

Figure S104. ^{1}H, ^{13}C- HSQC NMR (500-126 MHz, D_2O) spectrum of the reaction mixture resulting from the reaction corresponding to Table S2; Entry 5. Ethylene Carbonate is used as an internal standard.

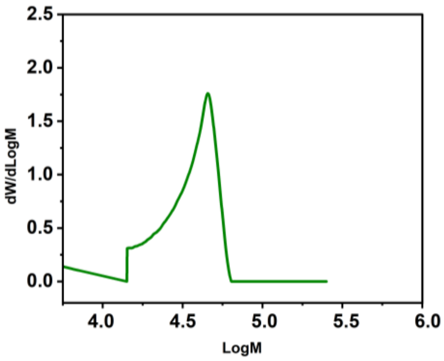

Figure S105. GPC data of the reaction mixture resulting from the reaction corresponding to Table S2; Entry 5.

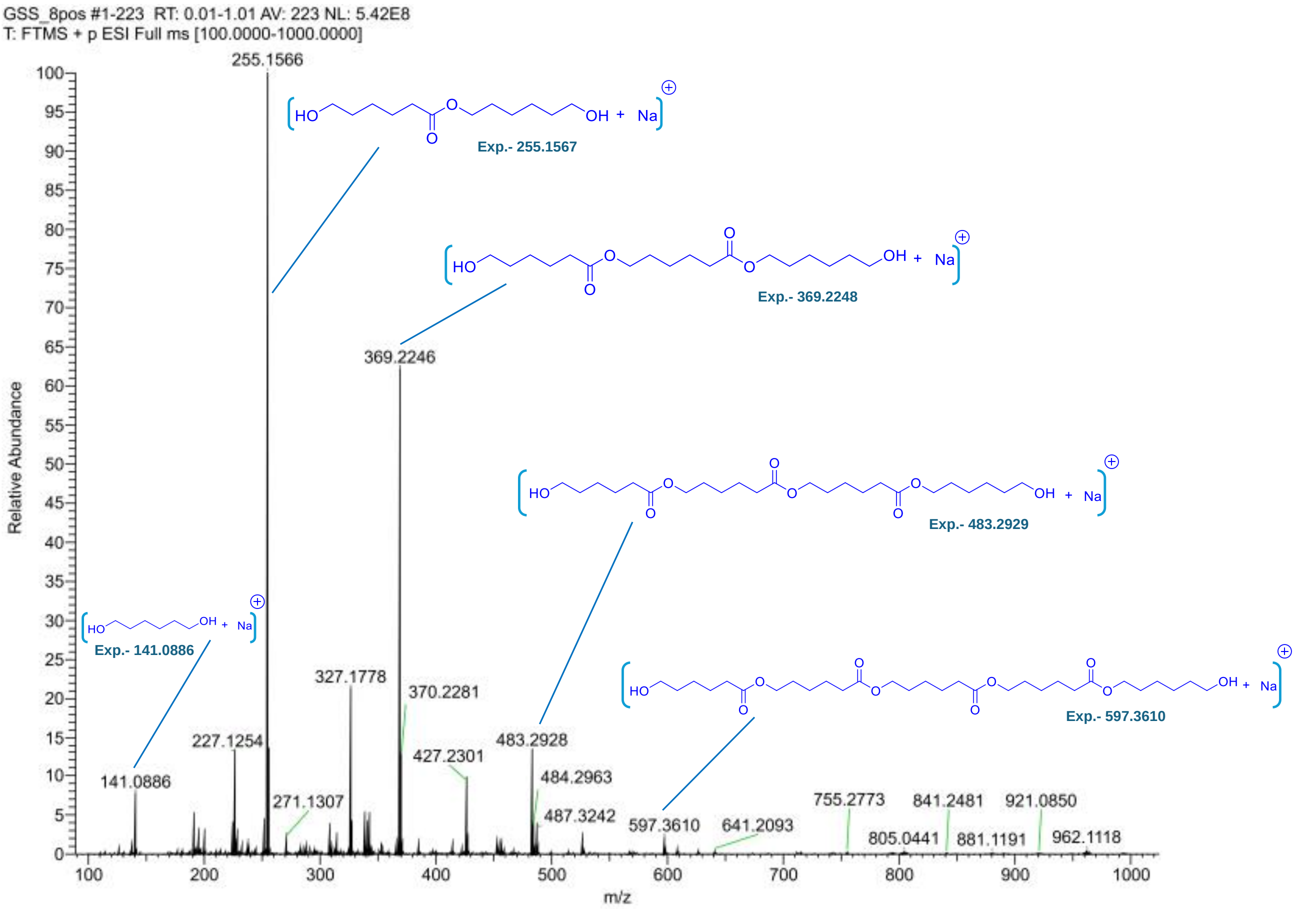

Figure S106. ESI- MS of the reaction mixture resulting from the reaction corresponding to Table S2; Entry 5.

<u>Table S2; Entry 6:</u>

Product formed: Polyester

^{1}H NMR (500 MHz, DMSO-d$_6$): δH 4.1 (br,-O-**CH$_2$**-CH$_2$-CH$_2$-(CH$_2$- CH$_2$-)$_2$-CH$_2$-CH$_2$-CO-), 2.3 (br,-O-CH$_2$-CH$_2$-CH$_2$-(CH$_2$- CH$_2$-)$_2$-CH$_2$-**CH$_2$**-CO-),1.5 (br,-O-CH$_2$-**CH$_2$**-CH$_2$-(CH$_2$- CH$_2$-)$_2$-**CH$_2$**-CH$_2$-CO-),1.4-1.2 (br,-O-CH$_2$-CH$_2$-**CH$_2$-(CH$_2$- CH$_2$-**)$_2$-CH$_2$-CH$_2$-CO-).

^{13}C{^{1}H} NMR (126 MHz DMSO-d$_6$): δC 206.9 (**-CHO**), 173.4 (-O-CH$_2$-CH$_2$-CH$_2$-(CH$_2$- CH$_2$-)$_2$-CH$_2$-CH$_2$-**CO**-), 64.1 (-O-**CH$_2$**-CH$_2$-CH$_2$-(CH$_2$- CH$_2$-)$_2$-CH$_2$-CH$_2$-CO-), 30.9(-O-CH$_2$-**CH$_2$**-CH$_2$-(CH$_2$- CH$_2$-)$_2$-CH$_2$-**CH$_2$**-CO-), 29.5-28.6(-O-CH$_2$-CH$_2$-CH$_2$-(**CH$_2$- CH$_2$**-)$_2$-CH$_2$-CH$_2$-CO-), 24.9 (-O-CH$_2$-CH$_2$-**CH$_2$**-(CH$_2$- CH$_2$-)$_2$-**CH$_2$**-CH$_2$-CO-).

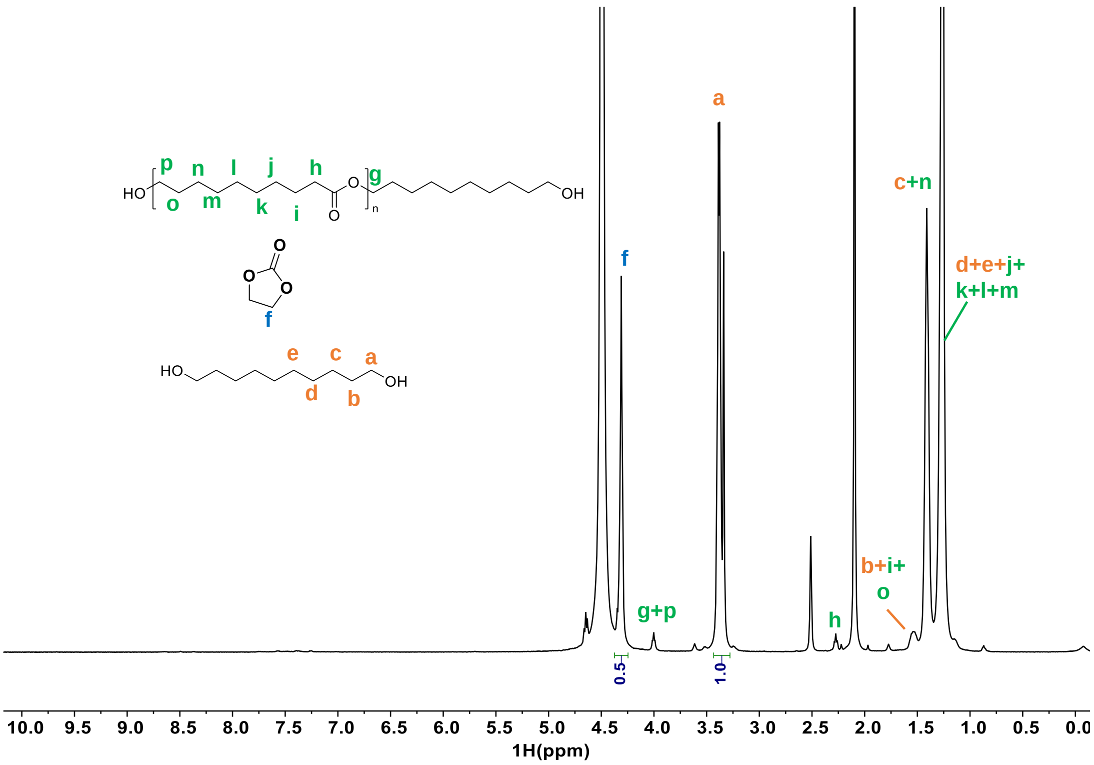

Figure S107. ^{1}H NMR (500 MHz, DMSO-d_6) spectrum of the reaction mixture resulting from the reaction corresponding to Table S2; Entry 6. Ethylene Carbonate (0.6 mmol) is used as an internal standard.

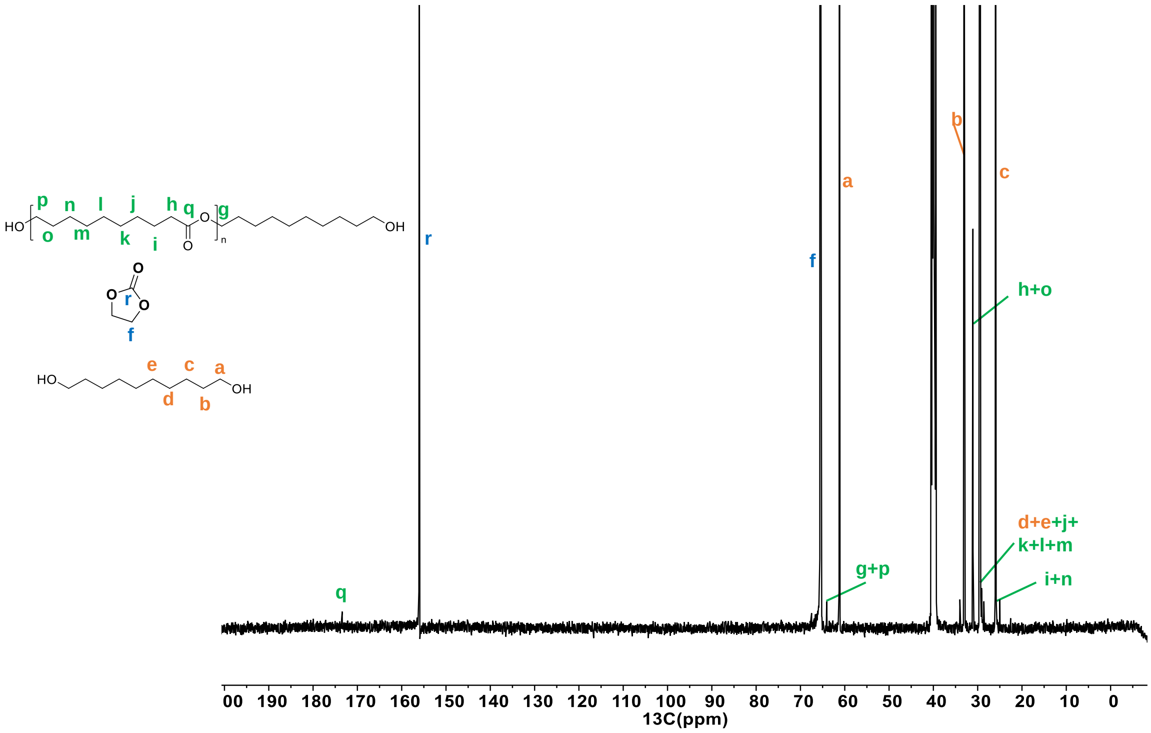

Figure S108. $^{13}C\{^{1}H\}$ NMR (126 MHz, DMSO-d_6) spectrum of the reaction mixture resulting from the reaction corresponding to Table S2; Entry 6. Ethylene Carbonate is used as an internal standard.

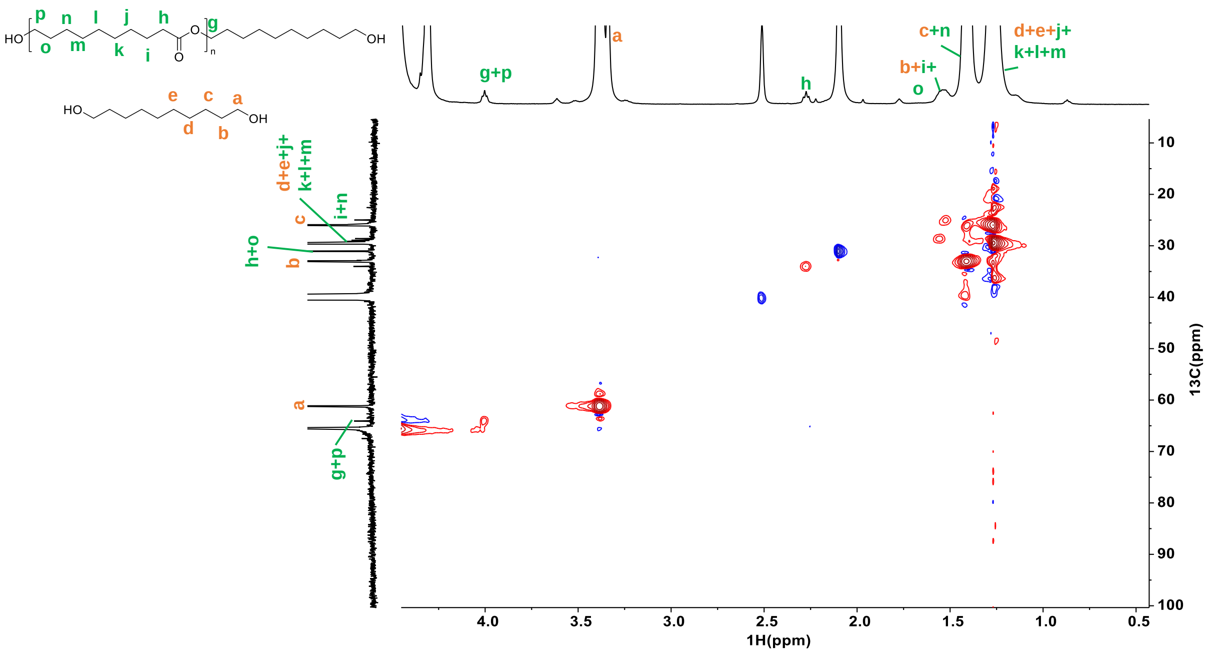

Figure S109. ^{1}H, ^{13}C- HSQC NMR (500-126 MHz, DMSO-d_6) spectrum of the reaction mixture resulting from the reaction corresponding to Table S2; Entry 6. Ethylene Carbonate is used as an internal standard.

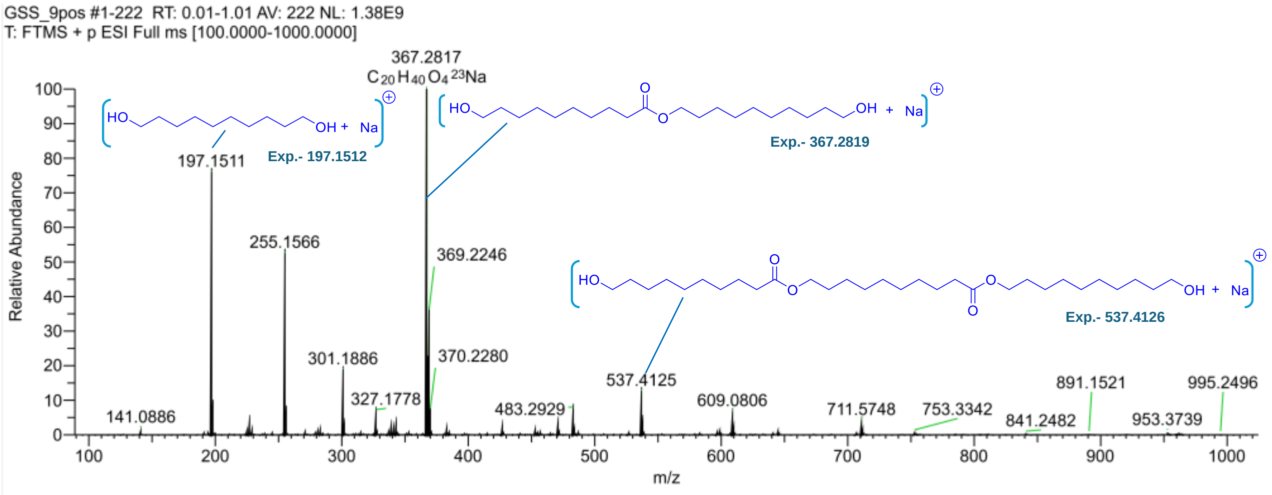

Figure S110. ESI- MS of the reaction mixture resulting from the reaction corresponding to Table S2; Entry 6.

<u>Table S2; Entry 7:</u>

^{1}H NMR (500 MHz, D_2O): δH 4.1 (br,-CO-CH$_2$- CH$_2$-**CH**-OH), 2.3 (br,-CO-**CH$_2$**- CH$_2$-CH-OH), 2.0 (br,-CO-CH$_2$- **CH$_2$**-CH-OH).

^{13}C{^{1}H} NMR (126 MHz, D_2O): δC 218.0 (-**CO**-CH$_2$- CH$_2$-CH-OH -), 66.1(-CO-CH$_2$- CH$_2$-**CH**-OH), 36.9(-CO-**CH$_2$**- CH$_2$-CH-OH), 31.7(-CO-CH$_2$- **CH$_2$**-CH-OH).

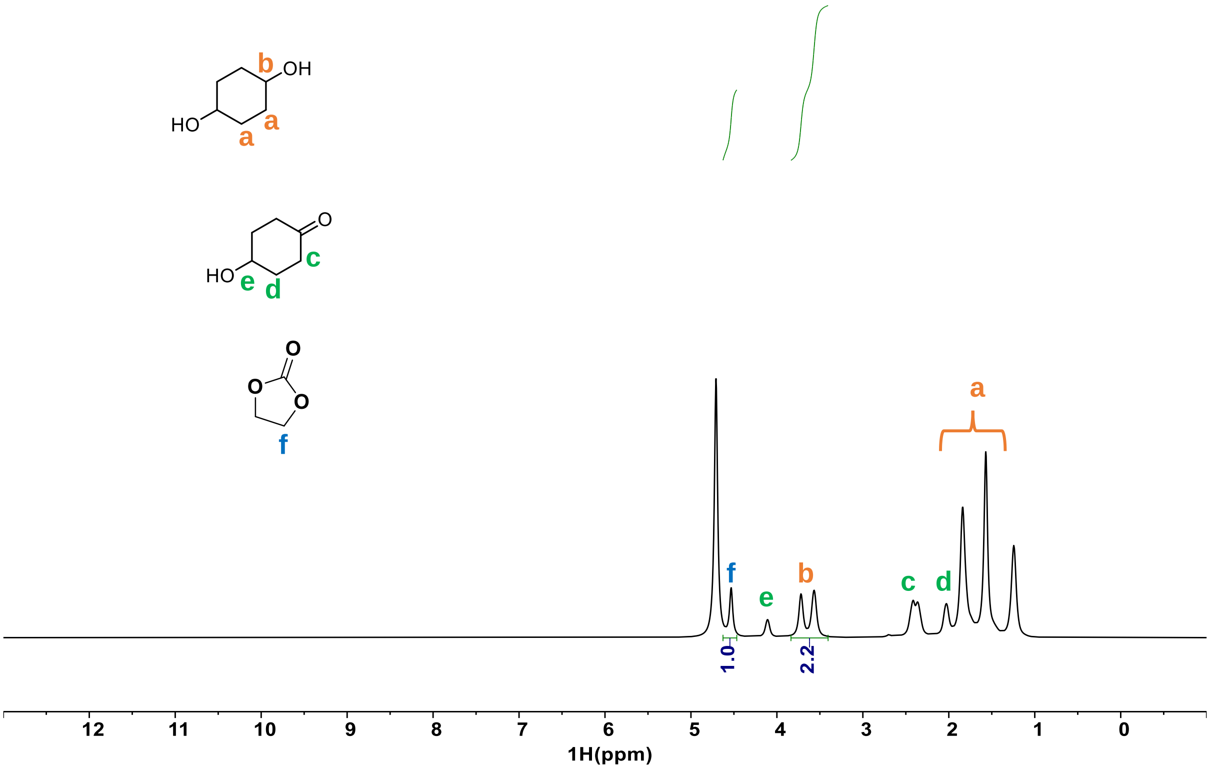

Figure S111. ^1H NMR (500 MHz, D_2O) spectrum of the reaction mixture resulting from the reaction corresponding to Table S2; Entry 7. Ethylene Carbonate (0.4 mmol) is used as an internal standard.

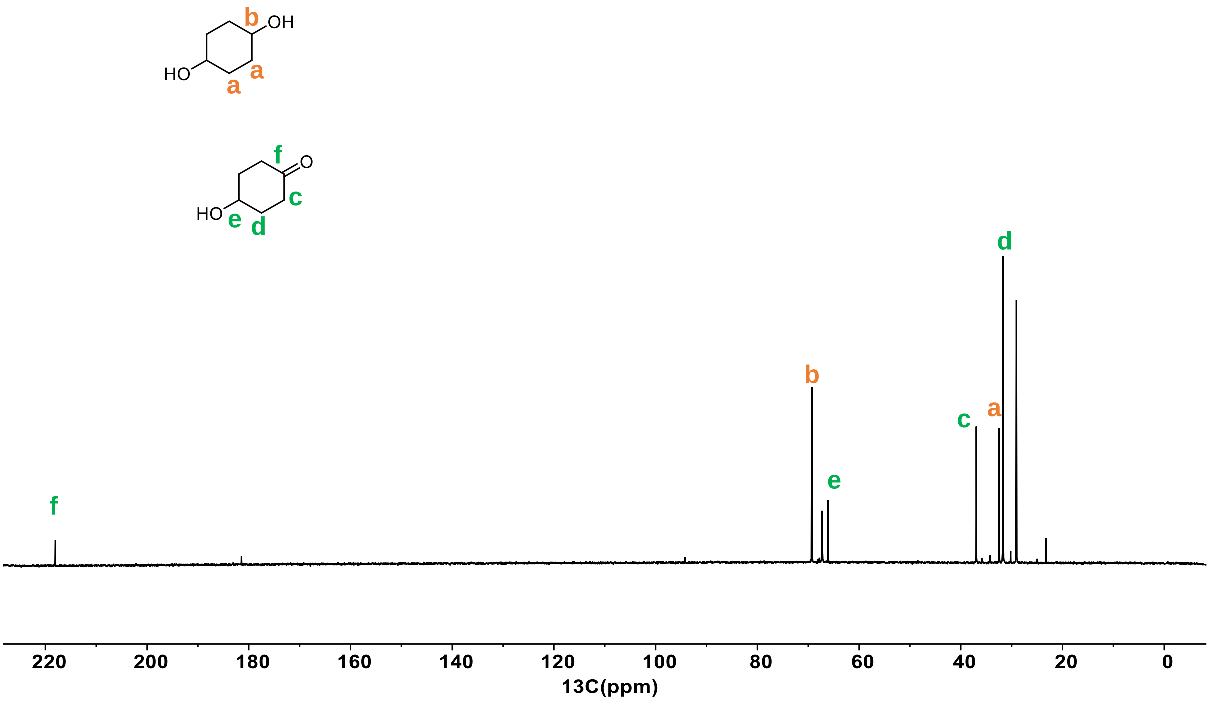

Figure S112. $^{13}C\{^1H\}$ NMR (126 MHz, D_2O) spectrum of the reaction mixture resulting from the reaction corresponding to Table S2; Entry 7.

1.12 Reaction with Alkenols

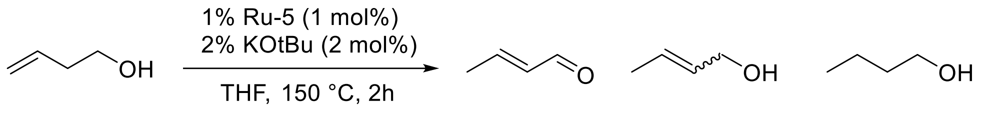

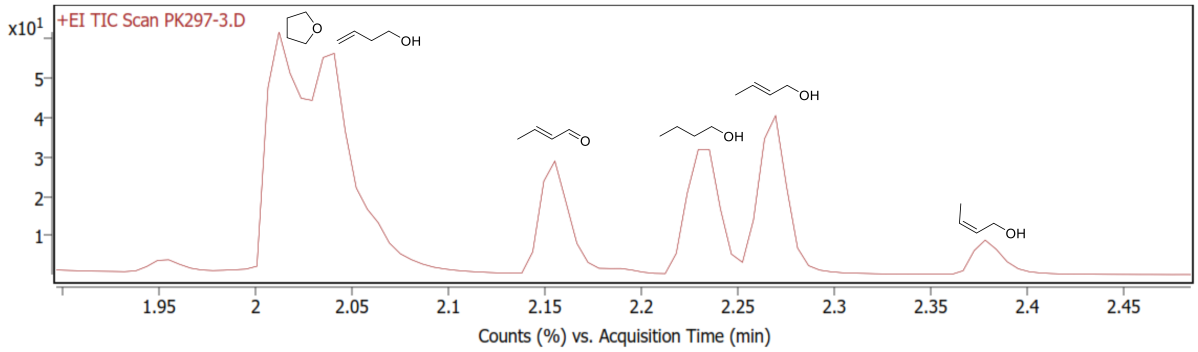

Figure S113. EI- TIC Scan corresponding to reaction with 3-Buten-1-ol.

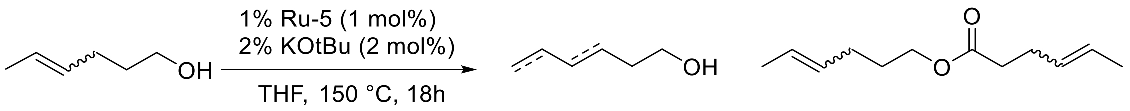

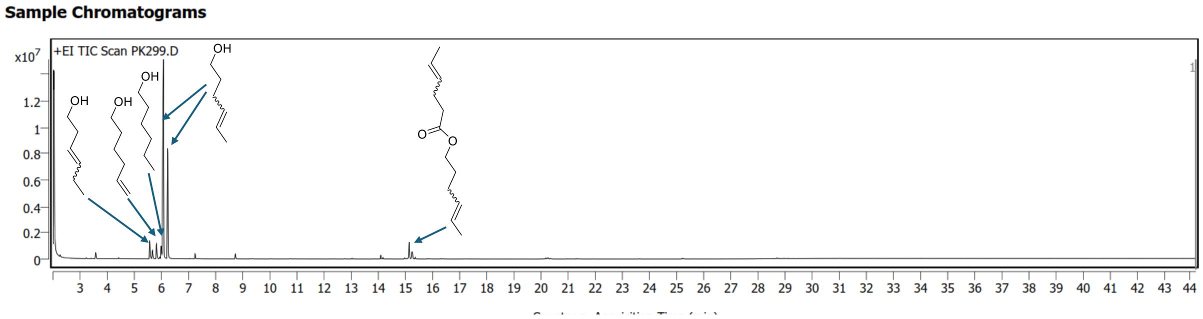

Figure S114. EI- TIC Scan corresponding to reaction with 3-Hexen-1-ol.

1.13 Reaction of Ethylene Glycol and Hexanal

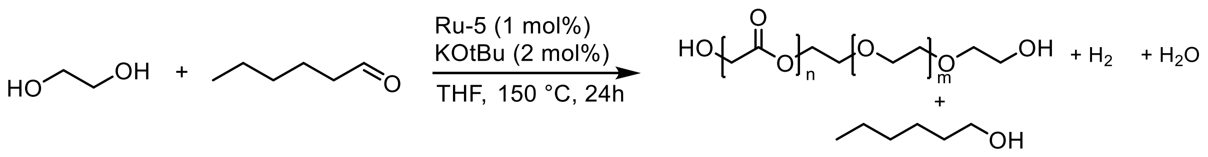

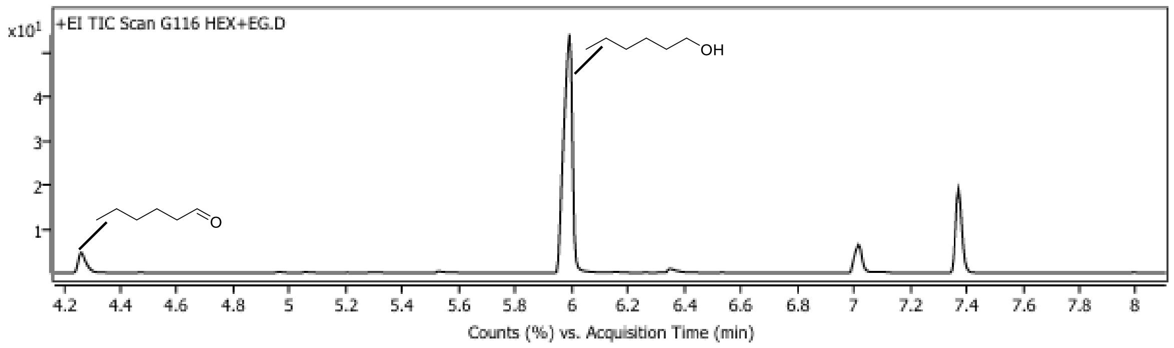

Figure S115. EI- TIC Scan corresponding to reaction with Ethylene glycol and Hexanal.

1.14 Catalyst stability study during reaction

Study with catalyst and base:

Complex Ru-5 (0.04mmol), KO^{t}Bu (0.08 mmol) were added in 2 mL of THF. An aliquot of the mixture (0.5 mL) was then taken in Young's NMR tube under Ar atmosphere and immediately given for NMR at room temperature. The NMR tube was then heated at 60 °C and the reaction was monitored by ^{1}H and ^{31}P{^{1}H} NMRs over 24 h at different time intervals. Both ^{1}H and ^{31}P{^{1}H} NMRs show mixture of species with some species remaining consistent over time.

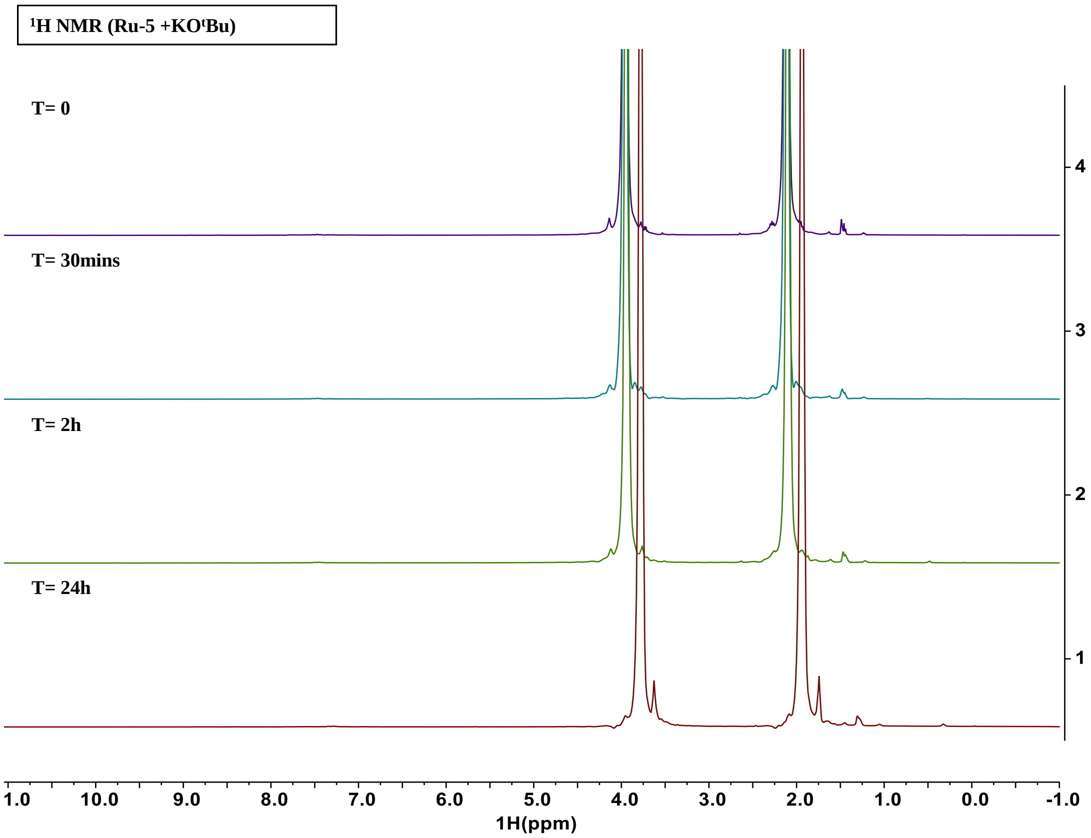

Figure S116. ^{1}H NMR (400 MHz, DMSO-d$_6$) spectrum of the reaction mixture resulting from the reaction with catalyst and base.

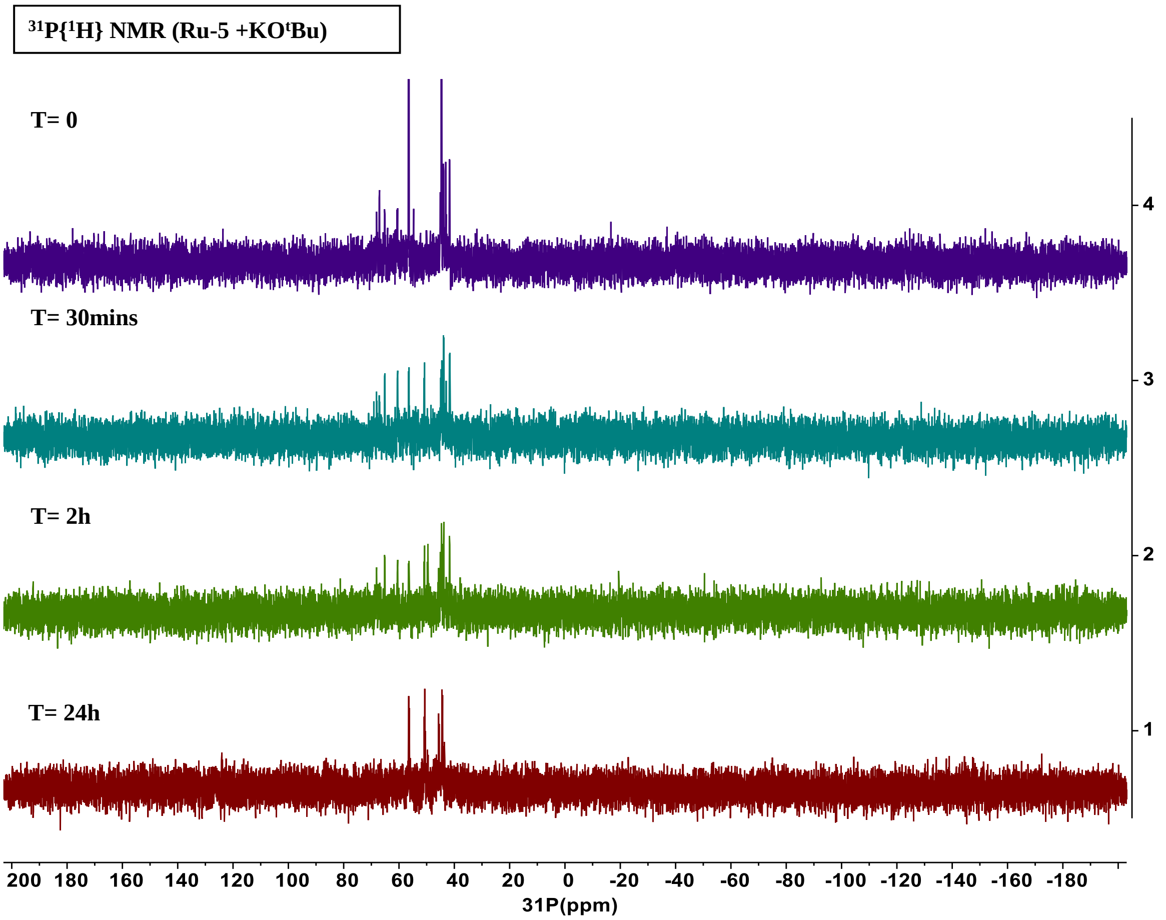

Figure S117. $^{31}P\{^{1}H\}$ NMR (DMSO-d_6) spectrum of the reaction mixture resulting from the reaction with catalyst and base.

Study with catalyst, base and ethylene glycol:

Complex Ru-5 (0.04mmol), KO^{t}Bu (0.08 mmol) and Ethylene Glycol (1 mmol) were added in 2 mL of THF. An aliquot of the mixture (0.5 mL) was then taken in Young's NMR tube under Ar atmosphere and immediately given for NMR at room temperature. The NMR tube was then heated at 60 °C and the reaction was monitored by ^{1}H and $^{31}P\{^{1}H\}$ NMRs over 24 h at different time intervals. Both ^{1}H and $^{31}P\{^{1}H\}$NMRs show mixture of species with some species remaining consistent over time.

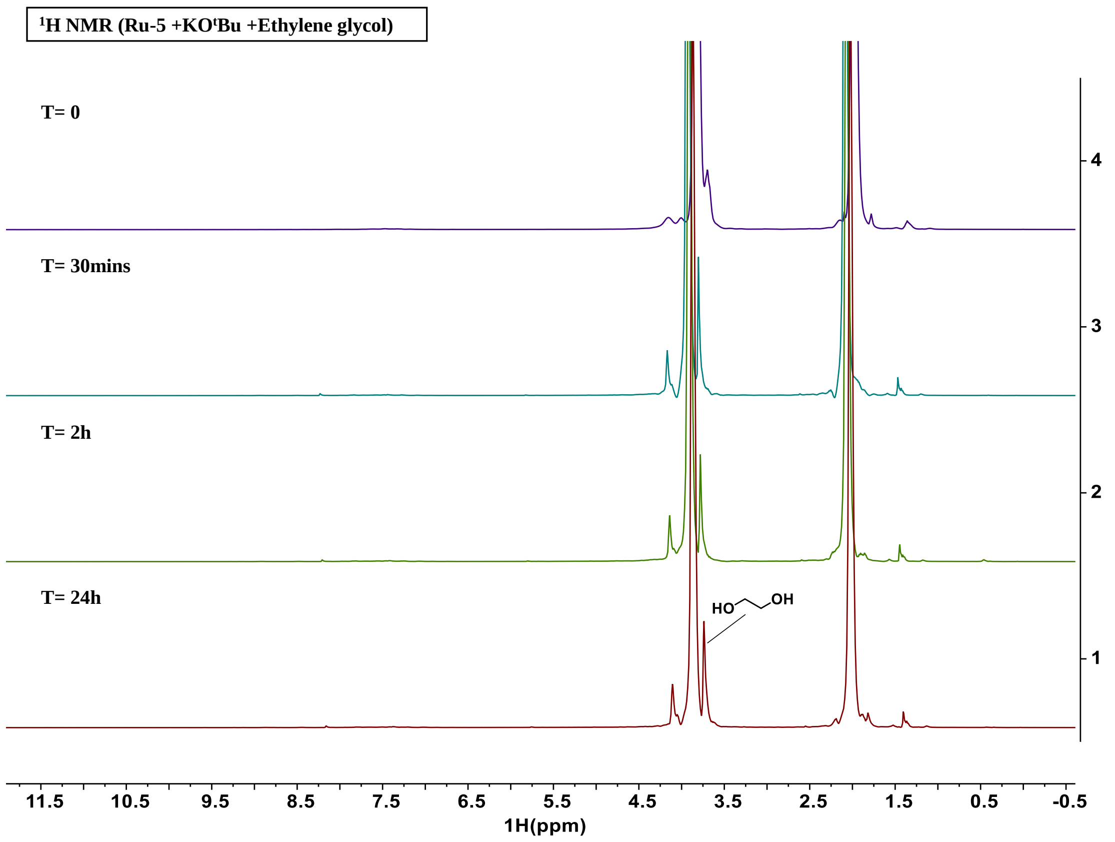

Figure S118. ^{1}H NMR (400 MHz, DMSO-d$_6$) spectrum of the reaction mixture resulting from the reaction with catalyst, base and Ethylene glycol.

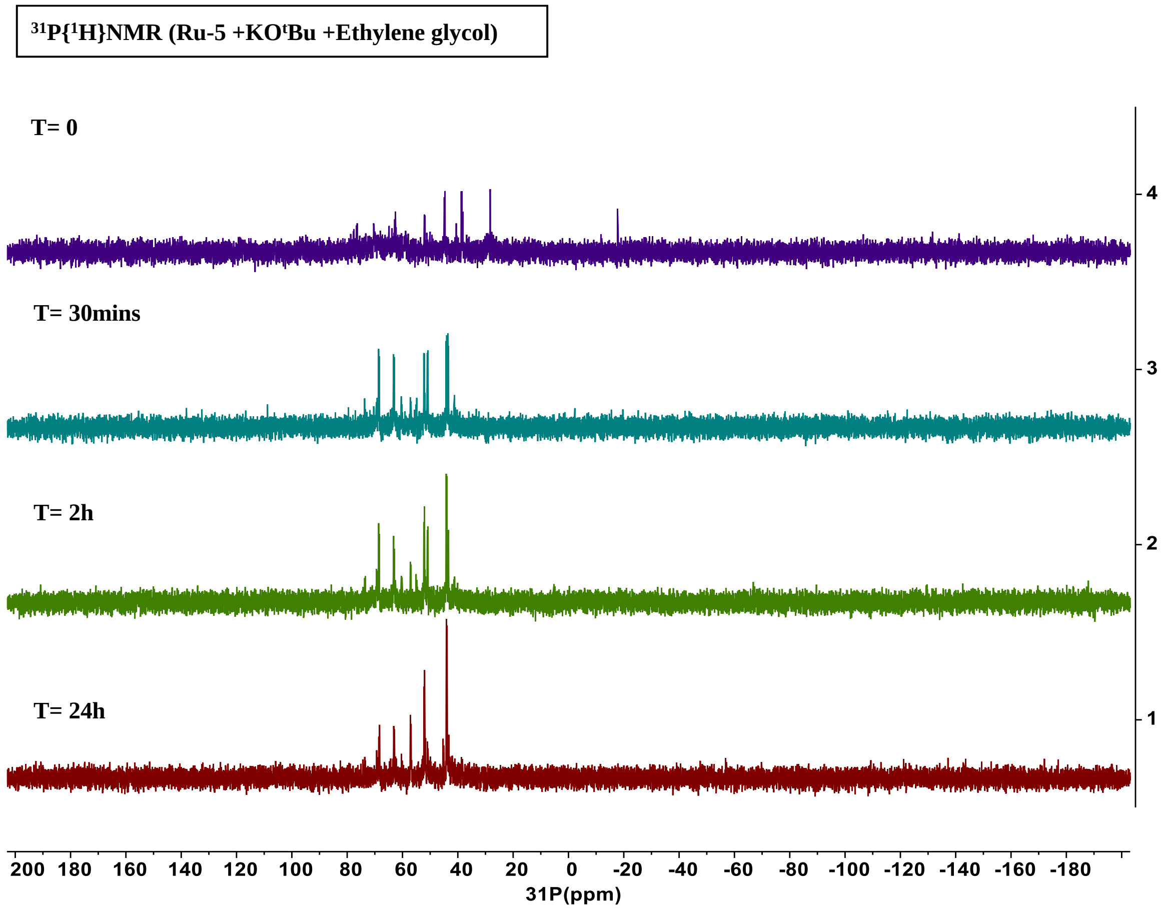

Figure S119. $^{31}P\{^1H\}$ NMR (DMSO-d_6) spectrum of the reaction mixture resulting from the reaction with catalyst, base and ethylene glycol.

1.15 Study of organometallic species during catalysis

A 100 mL ampoule equipped with a J-Young's valve was charged with **Ru-5** (33.2 mg, 0.04 mmol, 4 mol%, 1 eq.) and KO^{t}Bu (8.9 mg, 0.08 mmol, 8 mol%, 2 eq.). THF (2 mL) and ethylene glycol (0.11 mL, 2.0 mmol, 50 eq.) were added, and the flask was sealed under an argon atmosphere before heating to 150 °C for 1 hour with stirring. After this period, the reaction vessel was allowed to cool to room temperature. An aliquot of the mixture (0.5 mL) was then taken in a Young's NMR tube with C_6D_6 capillary in it under Ar atmosphere and immediately analysed by NMR spectroscopy (^1H and $^{31}P\{^1H\}$) and ESI-MS. The reaction was then left for further 24 h at room temperature and again analysed by NMR spectroscopy (^1H and $^{31}P\{^1H\}$) and ESI-MS to look for any potential changes over time.

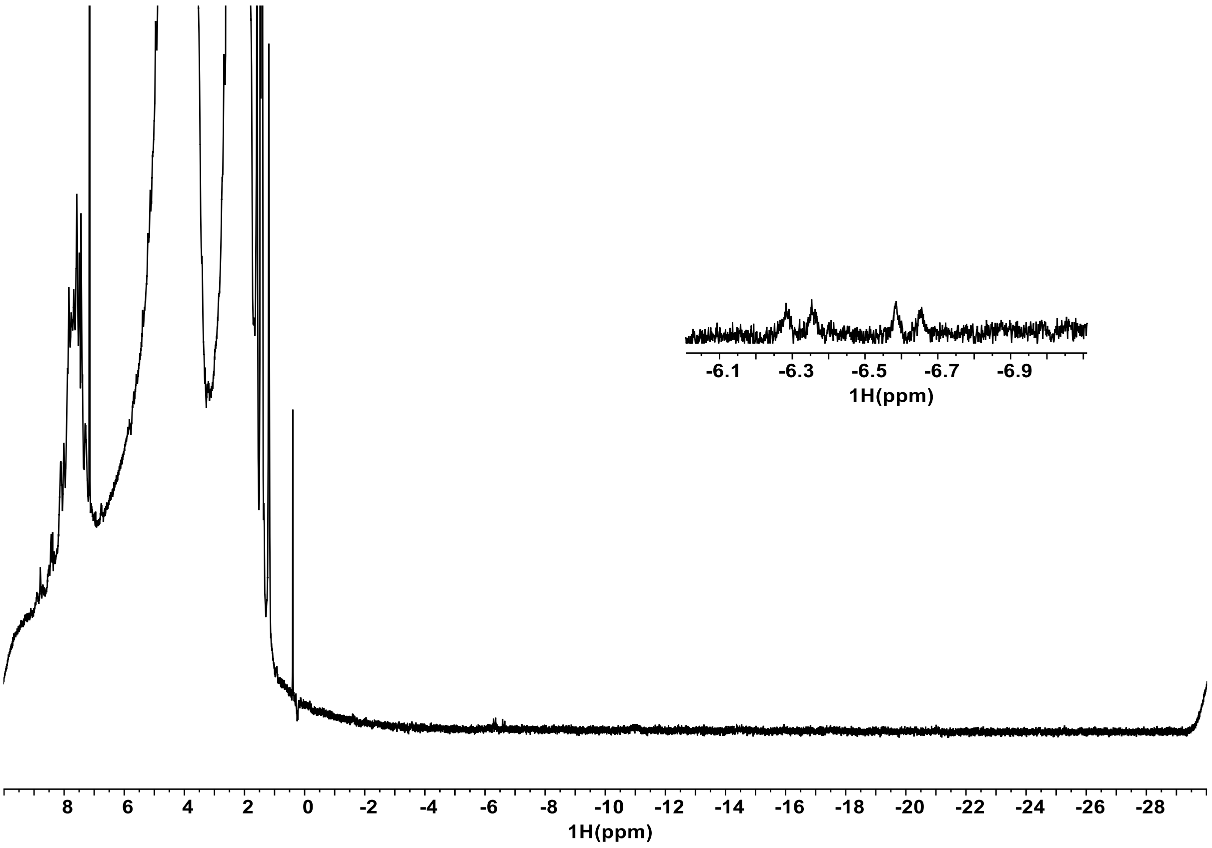

Figure S120. ^1H NMR (400 MHz, C_6D_6) spectrum of the reaction mixture resulting from the reaction of precatalyst, base and ethylene glycol heated at 150 °C for 1h. NMR was taken immediately after 1 h of the reaction.

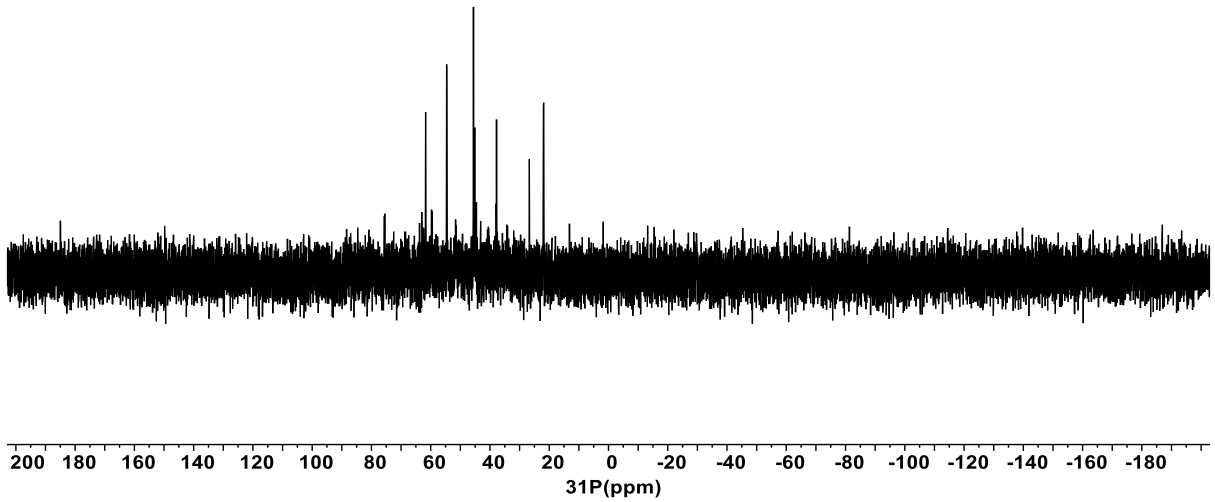

Figure S121. $^{31}P\{^1H\}$ NMR (C_6D_6) spectrum of the reaction mixture resulting from the reaction of precatalyst, base and ethylene glycol heated at 150 °C for 1h. NMR was taken immediately after completing the reaction.

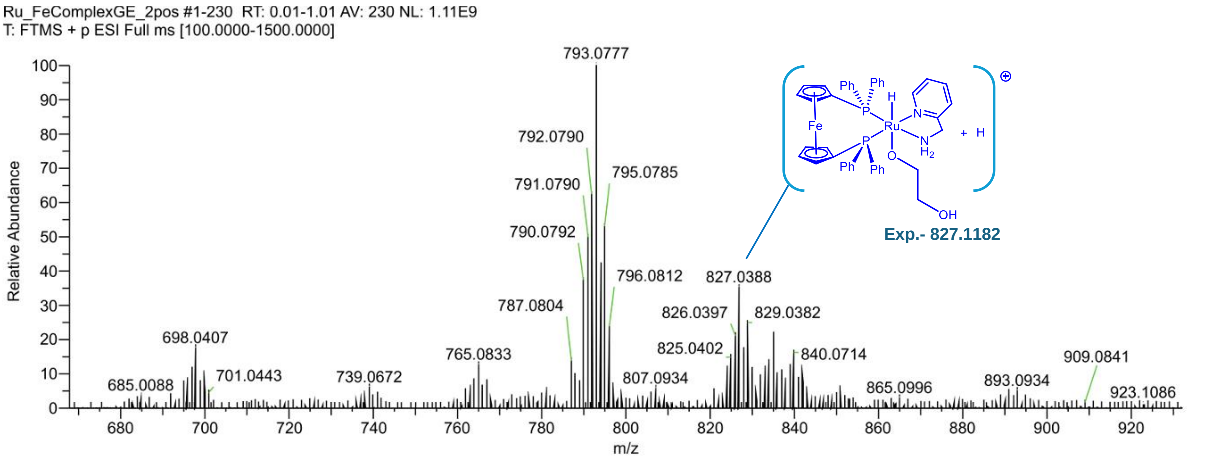

Figure S122. ESI- MS of the of the reaction mixture resulting from the reaction of precatalyst, base and ethylene glycol heated at 150 °C for 1h. ESI-MS was taken immediately after completing the reaction.

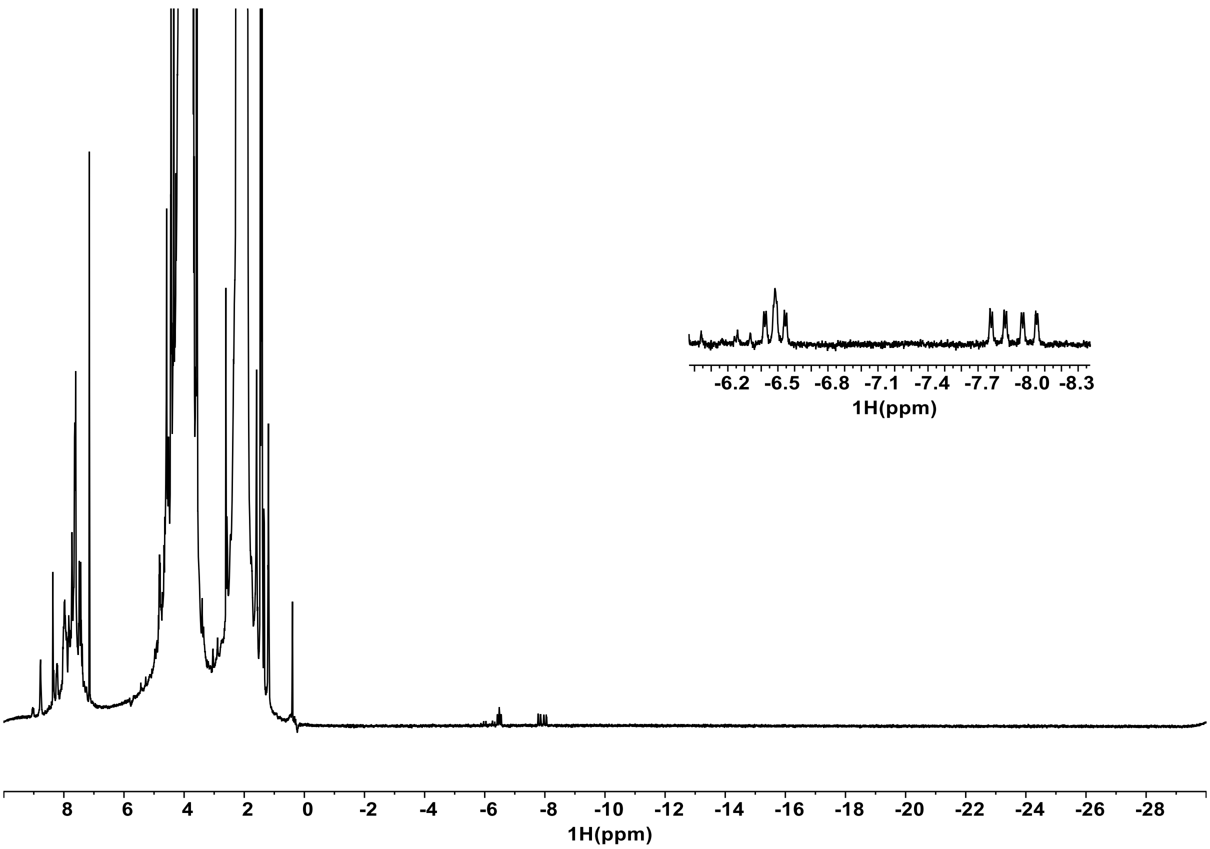

Figure S123. ^{1}H NMR (400 MHz, C_6D_6) spectrum of the reaction mixture resulting from the reaction of precatalyst, base and ethylene glycol heated at 150 °C for 1h. NMR was taken after keeping the reaction mixture at room temperature for 24 h.

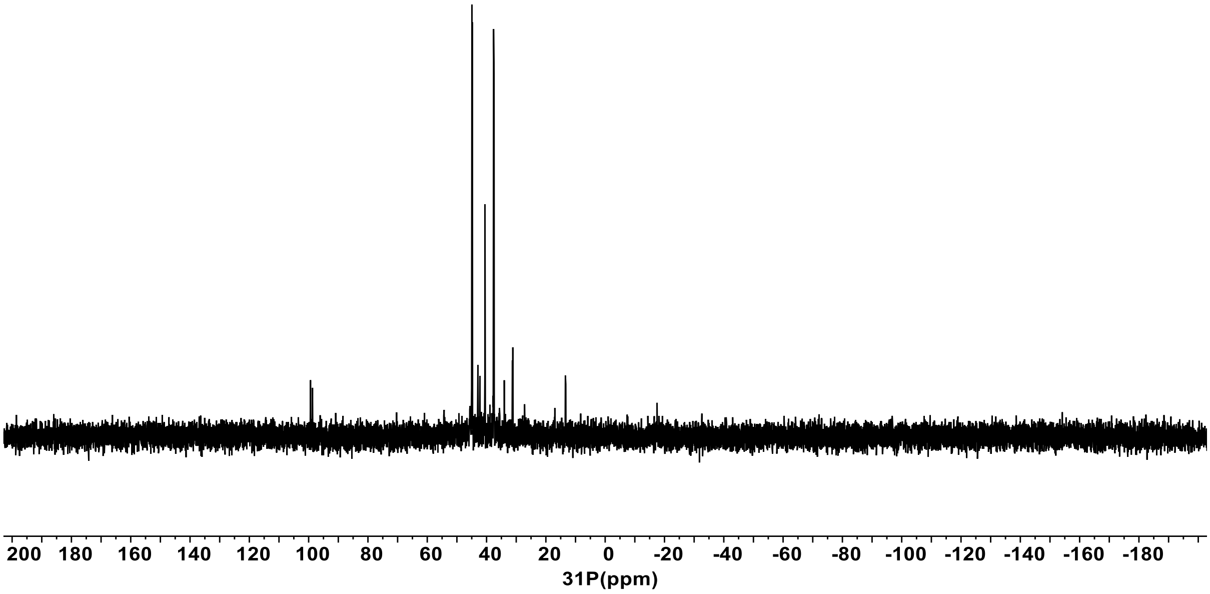

Figure S124. $^{31}P\{^{1}H\}$ NMR (C_6D_6) spectrum of the reaction mixture resulting from the reaction of the precatalyst, base and ethylene glycol heated at 150 °C for 1h. NMR was taken after keeping the reaction mixture at room temperature for 24 h.

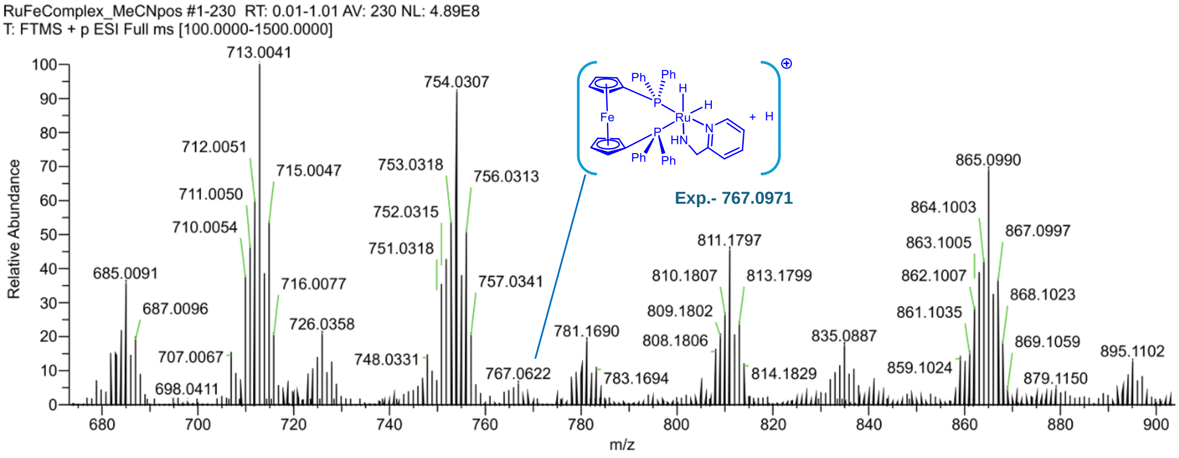

Figure S125. ESI- MS of the reaction mixture resulting from the reaction of the precatalyst, base and ethylene glycol heated at 150 °C for 1h. NMR was taken after keeping the reaction mixture at room temperature for 24 h.

1.16 DFT Studies

All computed structures can be found at the free online ioCHEM-BD repository under the following link:

https://iochem-bd.bsc.es/browse/review-collection/100/337436/827ba2018415452c7cad9775

All geometries were optimized using the M06-L functional,[1] the def2-SVP basis set[2] and W06 density fitting to increase computational efficiency,[3]as well as Grimme's empirical GD3 dispersion correction.[4] Frequency calculations at this level of theory confirmed stationary points and transition states and were used to compute thermodynamic properties at 298.15 K, if not stated otherwise. Single point energies of the optimized structures were computed using the range-separated hybrid meta-GGA exchange-correlation functional ωB97M -V,[5] including non-local correlation (VV10),[6,7] together with the triple-ζ def2-TZVPP basis set [2] and density fitting with the RIJCOSX formalism using the auxiliary basis sets def2/J [3] and def2-TZVPP/C[8] to speed up computational time. The choice of the ωB97M-V functional was rationalized given its excellent results in a recent benchmark study on transition metal reactions.[9] Gibbs Free Energies, unless otherwise stated, were computed by adding the Free Energy correction terms from the frequency calculations to the single point energies at the ωB97M-V/def2-TZVPP level of theory in

solution (SMD, Benzene) according to:

$$G_{SMD}^{\omega B97M-V} = E_{el/SMD}^{\omega B97M-V} + corr_{freq/T}^{M06L}$$

Where $corr_{freq/T}^{M06L}$ is the thermal correction to the Gibbs Free Energy from the frequency calculation at temperature T.[10] ΔG_M^0 is reported at 423.15 K. We did not apply the Martin, Hay and Pratt correction to the Gibbs free energy in solution[11], as it was found to not significantly increase computational

accuracy[12]. Instead, we only applied standard state corrections (1 atm to 1M), which was shown to give good results for dehydrogenative pathways[13], where the pressure of hydrogen was kept at 1 atm, while all other species are corrected to 1M, according to the following formula:

$$\Delta G_M^0 = \Delta G_{atm}^0 + R_1 T ln(R_2 T Q)$$

With $R_1 = 8.31447 \, {}^{J}/_{mol.K}$, $R_2 = 0.08206 \, {}^{L.atm}/_{mol.K}$, $T = temperature\ in\ K$ and $Q = reaction\ quotient$

Optimizations, frequency calculations and single point energies were done using the Gaussian 16 software suite in the B.01 revision.[14]

Table S3. Rel. energy calculation for different Ruthenium species

Compound	IntA	IntA_1	IntA_2
Rel. energy (kcal/mol)	0.0	19.7	16.5
Structure	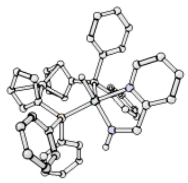	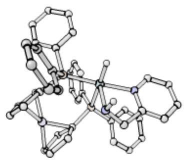	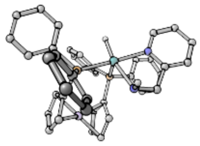

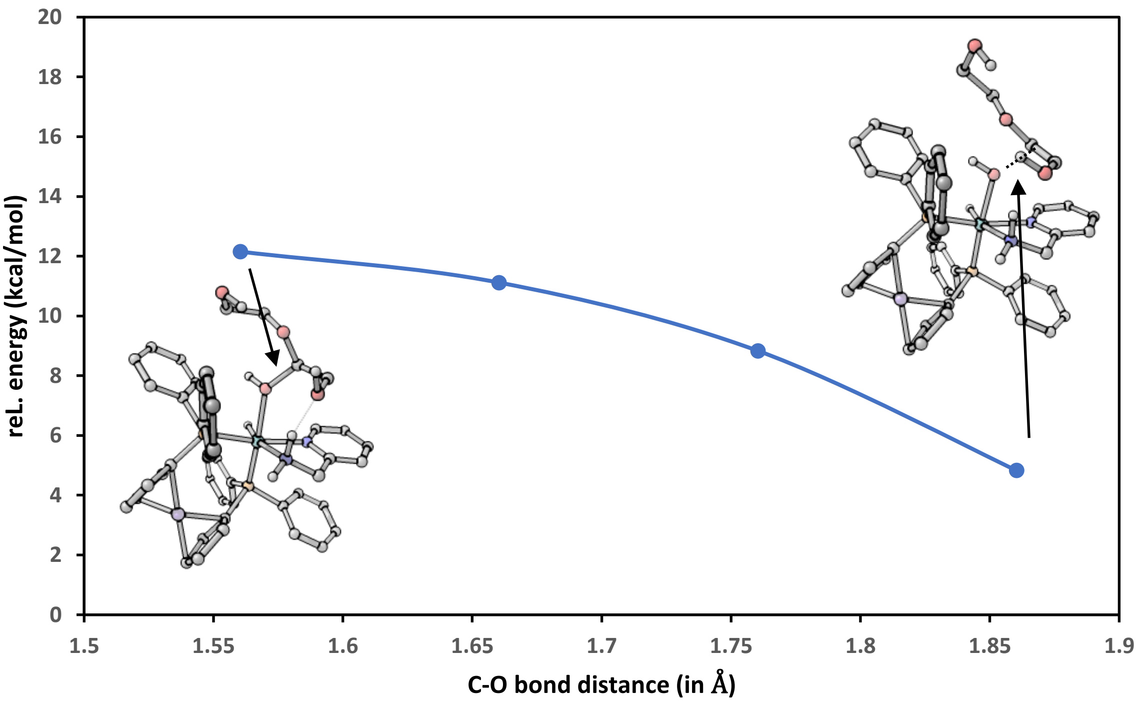

Figure S126. PES scan for the C-O bond dissociation of the hemiacetal coordinated to the Ru-center. No barrier is observed for the breaking of the C-O bond (water release).

1.17 Hydrolysis of Polyesterether

The hydrolysis of the polyesterether (M_n = 35,950 Da, Đ =1.2) was carried out using KOH as base and water as solvent at 150°C for 24h. After the completion of the reaction, the resulting reaction mixture was extracted with diethyl ether. Analysis of the reaction mixture showed a mixture of poly ethyleneglycol (confirmed by NMR spectroscopy) of Mn=1,550Da (Đ =2.1) and Mn=22,660Da (Đ =1.5). Additionally, ethylene glycol, and a higher molecular weight polyethylene glycol [Mn=55,580Da (Đ =1.2)] were also observed. We speculate that the high molecular weight of the polymer could be due to reaction of alcohol groups of polyethyleneglycol to the ester groups present in a polymer chain after hydrolysis.

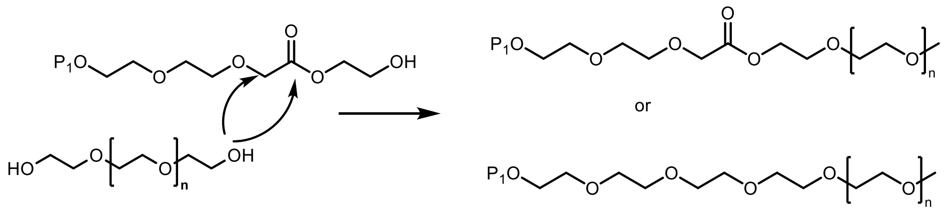

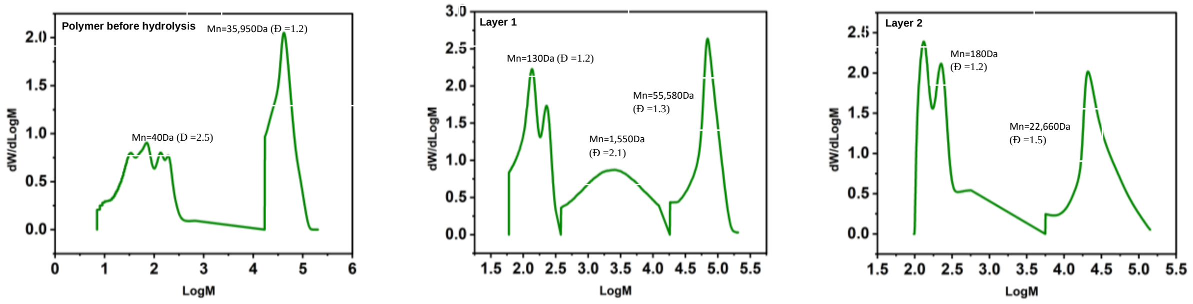

Figure S127. GPC data of the polymer before and after hydrolysis reaction.

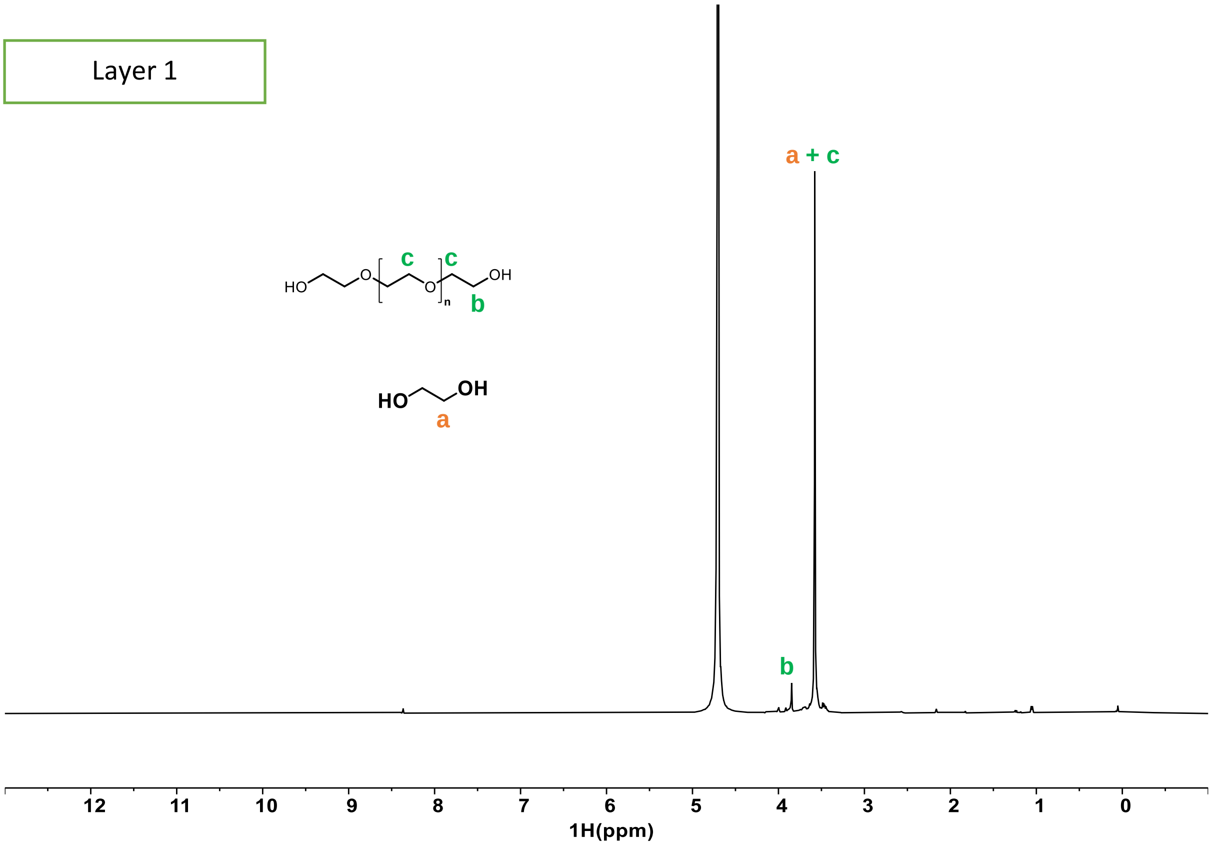

Figure S128. ^{1}H NMR (500 MHz, D_2O) spectrum of the reaction mixture resulting from the hydrolysis reaction.

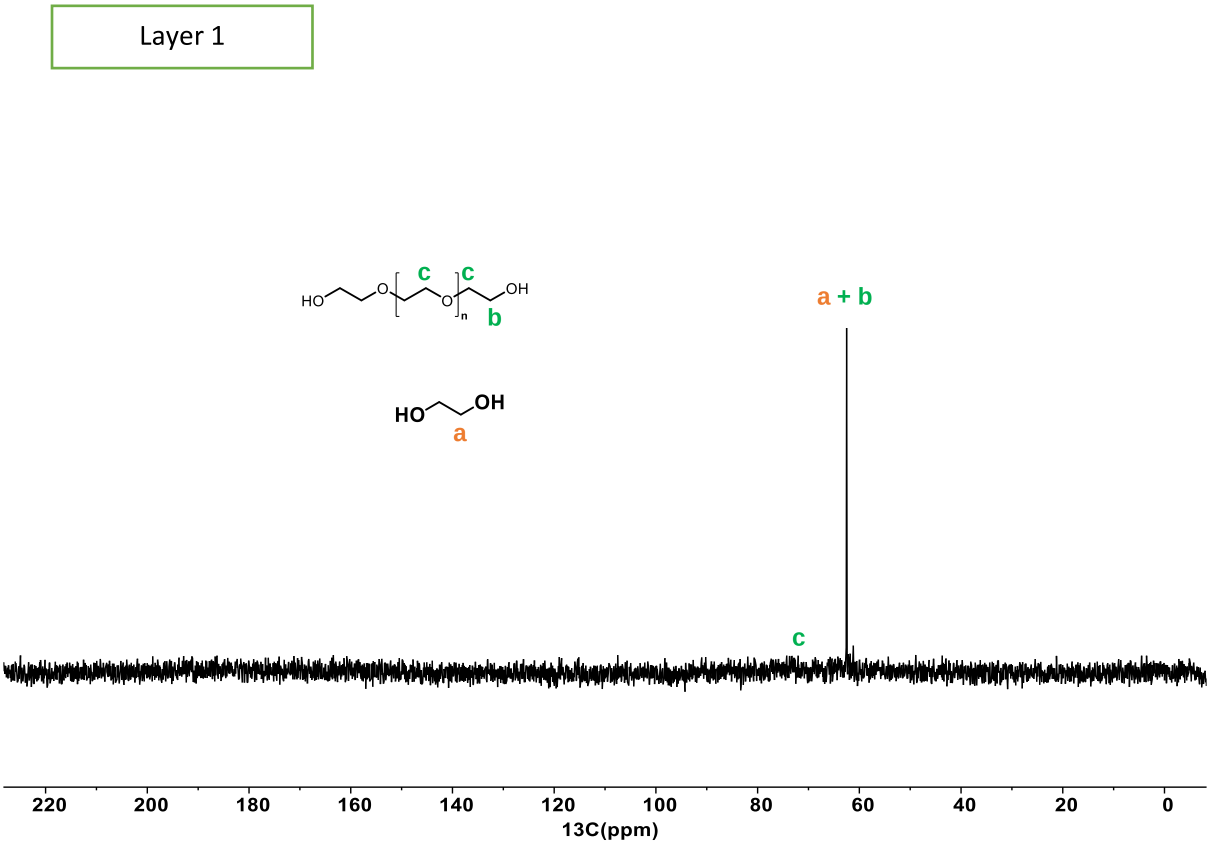

Figure S129. $^{13}C\{^{1}H\}$ NMR (126 MHz, D_2O) spectrum of the reaction mixture resulting from the hydrolysis reaction.

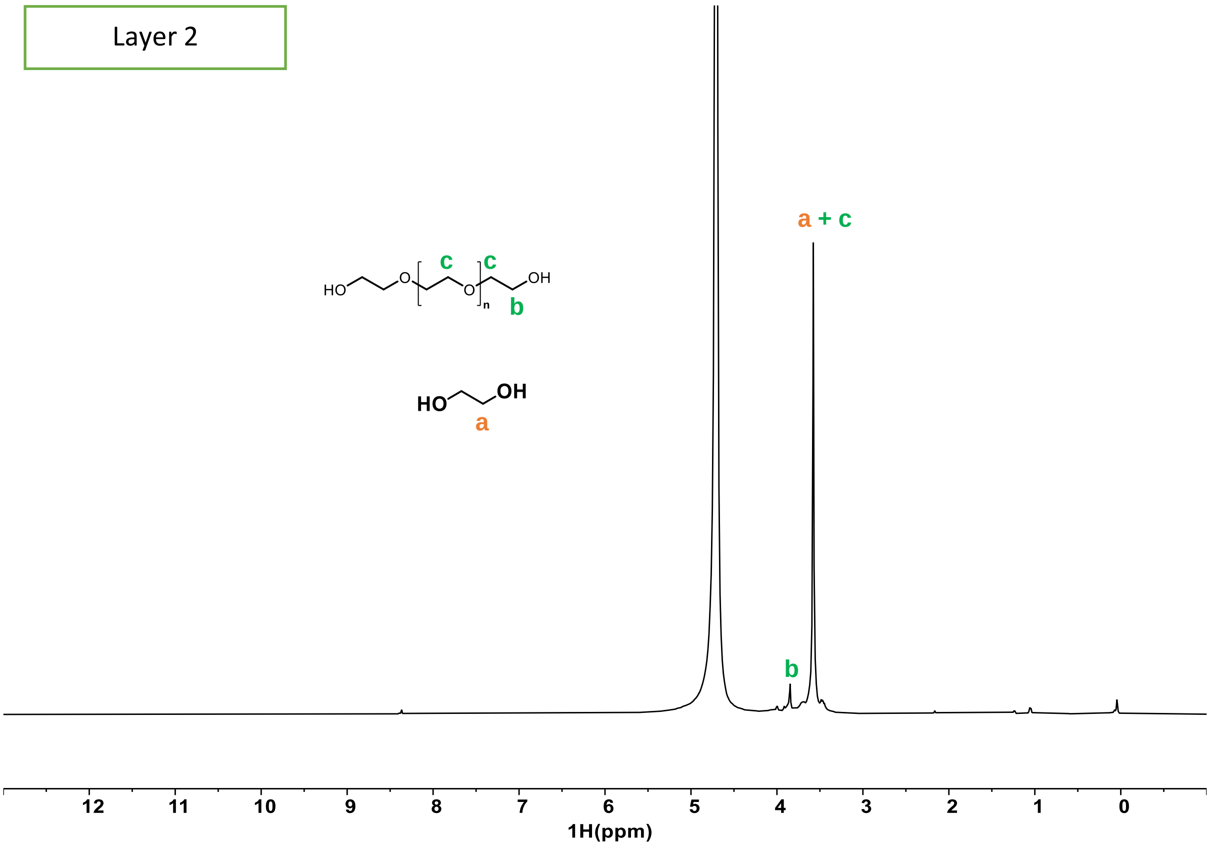

Figure S130. ^{1}H NMR (500 MHz, D_2O) spectrum of the reaction mixture resulting from the hydrolysis reaction.

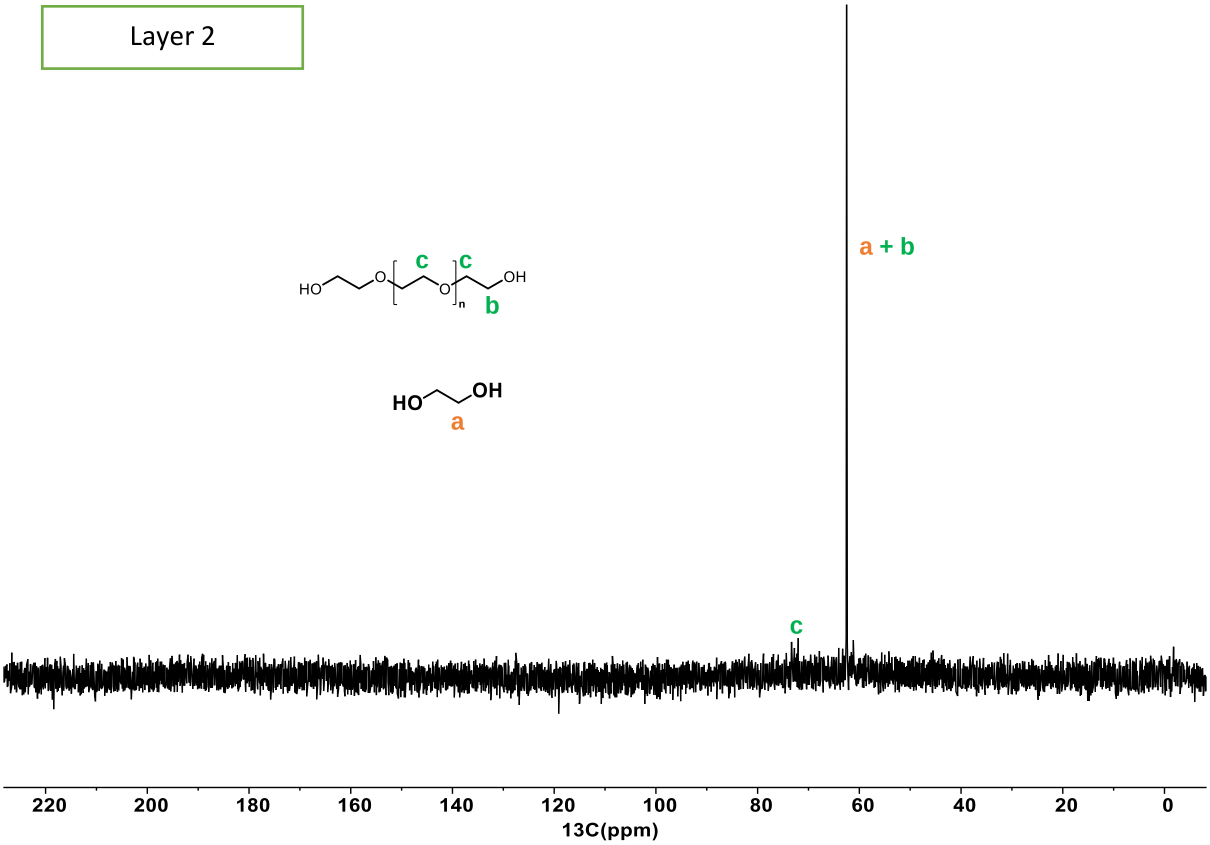

Figure S131 ^{13}C{^{1}H} NMR (126 MHz, D_2O) spectrum of the reaction mixture resulting from the hydrolysis reaction.

1.18 Reaction with 1-Phenylethanol

^{1}H NMR (500 MHz, D_2O): δH 7.9 (br,-CH-CH-**CH**- C-CO-CH_3), 7.6 (br,-**CH**-CH-CH- C-CO-CH_3), 7.4 (br,-CH-**CH**-CH- C-CO-CH_3), 2.6 (br,-CH-CH-CH- C-CO-**CH_3**).

^{13}C{^{1}H} NMR (126 MHz, D_2O): δC 202.2 (-CH-CH-CH- C-**CO**-CH_3), 136.4 (-CH-CH-CH- **C**-CO-CH_3), 134.1(-**CH**-CH-CH- C-CO-CH_3),128.5-125.6 (-CH-**CH-CH**- C-CO-CH_3), 26.2(-CH-CH-CH- C-CO-**CH_3**).

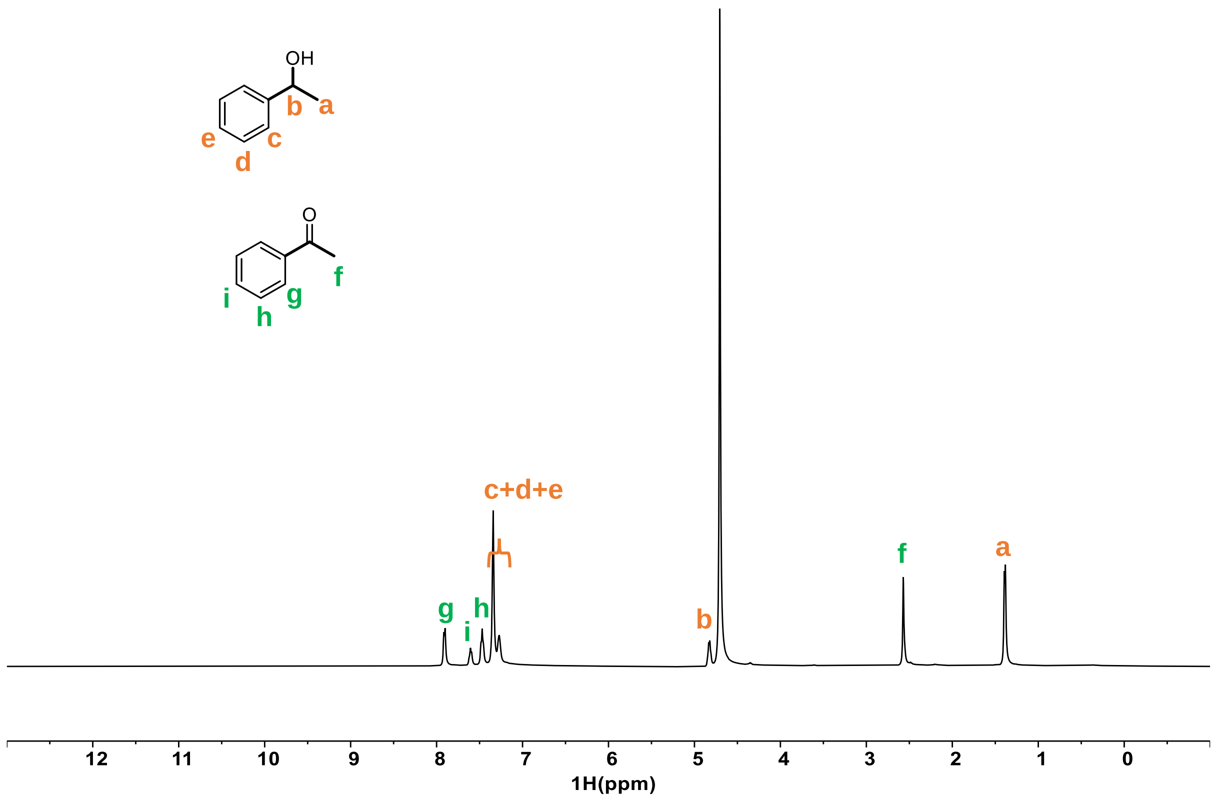

Figure S132. ^{1}H NMR (500 MHz, D_2O) spectrum of the reaction mixture resulting from the reaction done with 1-phenylethanol.

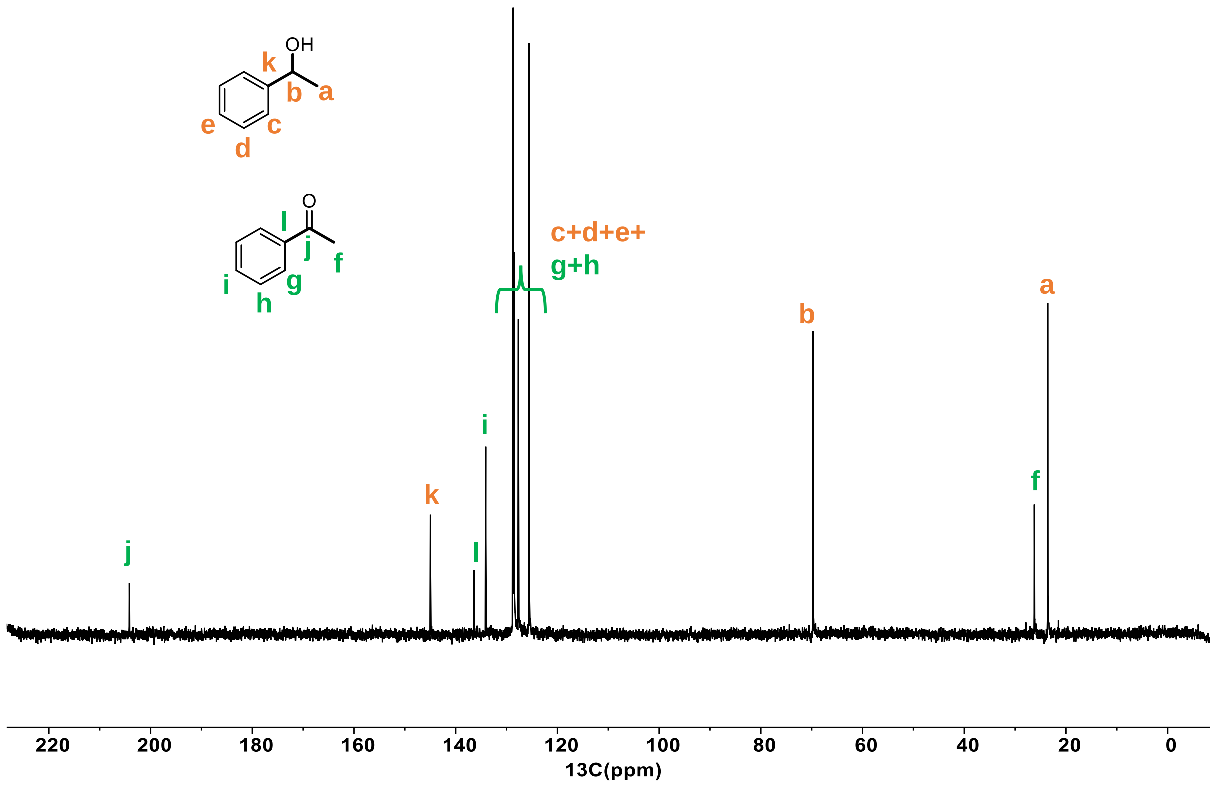

Figure S133. $^{13}C\{^{1}H\}$ NMR (126 MHz, D_2O) spectrum of the reaction mixture resulting from the reaction done with 1-phenylethanol.

1.19 References

(1) Zhao, Y.; Truhlar, D. G. A New Local Density Functional for Main-Group Thermochemistry, Transition Metal Bonding, Thermochemical Kinetics, and Noncovalent Interactions. *Journal of Chemical Physics* **2006**, *125* (19), 194101. https://doi.org/10.1063/1.2370993/566338.

(2) Weigend, F.; Ahlrichs, R. Balanced Basis Sets of Split Valence, Triple Zeta Valence and Quadruple Zeta Valence Quality for H to Rn: Design and Assessment of Accuracy. *Physical Chemistry Chemical Physics* **2005**, *7* (18), 3297–3305. https://doi.org/10.1039/B508541A.

(3) Weigend, F. Accurate Coulomb-Fitting Basis Sets for H to Rn. *Physical Chemistry Chemical Physics* **2006**, *8* (9), 1057–1065. https://doi.org/10.1039/B515623H.

(4) Grimme, S.; Antony, J.; Ehrlich, S.; Krieg, H. A Consistent and Accurate Ab Initio Parametrization of Density Functional Dispersion Correction (DFT-D) for the 94 Elements H-Pu. *Journal of Chemical Physics* **2010**, *132* (15), 154104. https://doi.org/10.1063/1.3382344/926936.

(5) Mardirossian, N.; Head-Gordon, M. ω B97M-V: A Combinatorially Optimized, Range-Separated Hybrid, Meta-GGA Density Functional with VV10 Nonlocal Correlation. *Journal of Chemical Physics* **2016**, *144* (21), 214110. https://doi.org/10.1063/1.4952647/15512462/214110_1_ACCEPTED_MANUSCRIPT.PDF.

(6) Vydrov, O. A.; Van Voorhis, T. Nonlocal van Der Waals Density Functional: The Simpler the Better. *Journal of Chemical Physics* **2010**, *133* (24), 244103. https://doi.org/10.1063/1.3521275/983192.

(7) Hujo, W.; Grimme, S. Performance of the van Der Waals Density Functional VV10 and (Hybrid)GGA Variants for Thermochemistry and Noncovalent Interactions. *J Chem Theory Comput* **2011**, *7* (12), 3866–3871. https://doi.org/10.1021/CT200644W/SUPPL_FILE/CT200644W_SI_001.PDF.

(8) Hellweg, A.; Hättig, C.; Höfener, S.; Klopper, W. Optimized Accurate Auxiliary Basis Sets for RI-MP2 and RI-CC2 Calculations for the Atoms Rb to Rn. *Theor Chem Acc* **2007**, *117* (4), 587–597. https://doi.org/10.1007/S00214-007-0250-5/METRICS.

(9) Iron, M. A.; Janes, T. Evaluating Transition Metal Barrier Heights with the Latest Density Functional Theory Exchange-Correlation Functionals: The MOBH35 Benchmark Database. *Journal of Physical Chemistry A* **2019**, *123* (17), 3761–3781. https://doi.org/10.1021/ACS.JPCA.9B01546/SUPPL_FILE/JP9B01546_SI_002.ZIP.

(10) Mondal, B.; Neese, F.; Ye, S. Control in the Rate-Determining Step Provides a Promising Strategy To Develop New Catalysts for CO2 Hydrogenation: A Local Pair Natural Orbital Coupled Cluster Theory Study. *Inorg Chem* **2015**, *54* (15), 7192–7198. https://doi.org/10.1021/ACS.INORGCHEM.5B00469/SUPPL_FILE/IC5B00469_SI_001.PDF.

(11) Martin, R. L.; Hay, P. J.; Pratt, L. R. Hydrolysis of Ferric Ion in Water and Conformational Equilibrium. *Journal of Physical Chemistry A* **1998**, *102* (20), 3565–3573. https://doi.org/10.1021/JP980229P/ASSET/IMAGES/MEDIUM/JP980229PE00016.GIF.

(12) Royle, C. G.; Sotorrios, L.; Gyton, M. R.; Brodie, C. N.; Burnage, A. L.; Furfari, S. K.; Marini, A.; Warren, M. R.; Macgregor, S. A.; Weller, A. S. Single-Crystal to Single-Crystal Addition of H 2

to [Ir(i Pr-PONOP)(Propene)][BAr F 4] and Comparison Between Solid-State and Solution Reactivity. https://doi.org/10.1021/acs.organomet.2c00274.

(13) Rauch, M.; Luo, J.; Avram, L.; Ben-David, Y.; Milstein, D. Mechanistic Investigations of Ruthenium Catalyzed Dehydrogenative Thioester Synthesis and Thioester Hydrogenation. *ACS Catal* **2021**, *11* (5), 2795–2807. https://doi.org/10.1021/ACSCATAL.1C00418/ASSET/IMAGES/LARGE/CS1C00418_0022.JPEG.

(14) *Citation | Gaussian.com*. https://gaussian.com/citation/ (accessed 2025-01-23).
